# Stabilizing Monoatomic Two-Coordinate Bismuth(I) and Bismuth(II) Using a Redox Noninnocent Bis(germylene) Ligand

**DOI:** 10.1021/jacs.3c13016

**Published:** 2024-02-26

**Authors:** Jian Xu, Sudip Pan, Shenglai Yao, Christian Lorent, Christian Teutloff, Zhaoyin Zhang, Jun Fan, Andrew Molino, Konstantin B. Krause, Johannes Schmidt, Robert Bittl, Christian Limberg, Lili Zhao, Gernot Frenking, Matthias Driess

**Affiliations:** † Metalorganic and Inorganic Materials, Department of Chemistry, Technische Universität Berlin, 10623 Berlin, Germany; ‡ Institute of Atomic and Molecular Physics, Jilin University, Changchun 130023, China; § Physical and Biophysical Chemistry, Department of Chemistry, Technische Universität Berlin, 10623 Berlin, Germany; ∥ Fachbereich Physik, Freie Universität Berlin, 14195 Berlin, Germany; ⊥ State Key Laboratory of Materials-Oriented Chemical Engineering, School of Chemistry and Molecular Engineering, Nanjing Tech University, Nanjing 211816, China; # Department of Chemistry and Physics, La Trobe Institute for Molecular Science, La Trobe University, Melbourne 3086 Victoria, Australia; ¶ Institut für Chemie, Humboldt-Universität zu Berlin, 12489 Berlin, Germany; ∇ Functional Materials, Department of Chemistry, Technische Universität Berlin, 10623 Berlin, Germany; ○ Fachbereich Chemie, Philipps-Universität Marburg, 35032 Marburg, Germany

## Abstract

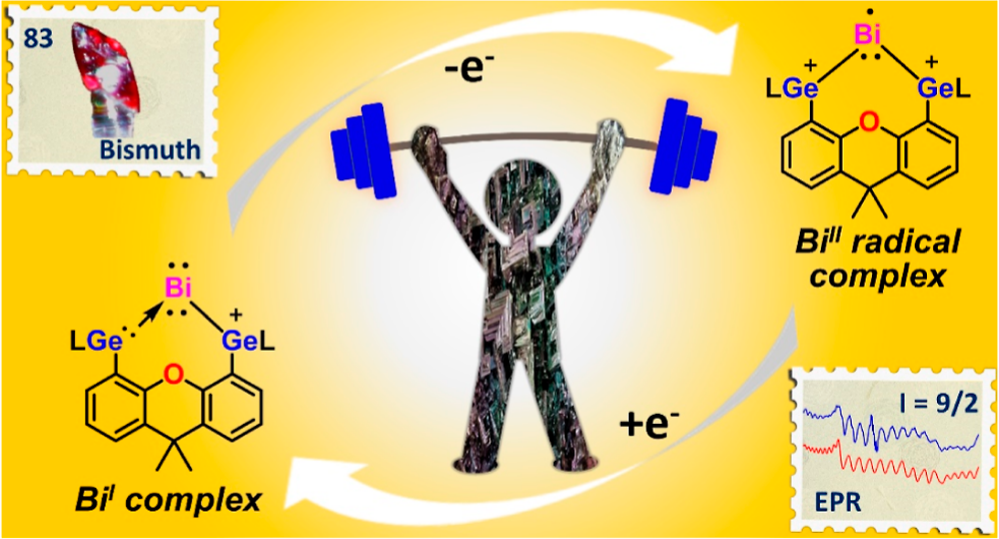

The formation of isolable monatomic Bi^I^ complexes and Bi^II^ radical species is challenging due to the pronounced reducing nature of metallic bismuth. Here, we report a convenient strategy to tame Bi^I^ and Bi^II^ atoms by taking advantage of the redox noninnocent character of a new chelating bis(germylene) ligand. The remarkably stable novel Bi^I^ cation complex **4**, supported by the new bis(iminophosphonamido-germylene)xanthene ligand [(P)Ge^II^(Xant)Ge^II^(P)] **1**, [(P)Ge^II^(Xant)Ge^II^(P) = Ph_2_P(N*t*Bu)_2_Ge^II^(Xant)Ge^II^(N*t*Bu)_2_PPh_2_, Xant = 9,9-dimethyl-xanthene-4,5-diyl], was synthesized by a two-electron reduction of the cationic Bi^III^I_2_ precursor complex **3** with cobaltocene (Cp_2_Co) in a molar ratio of 1:2. Notably, owing to the redox noninnocent character of the germylene moieties, the positive charge of Bi^I^ cation **4** migrates to one of the Ge atoms in the bis(germylene) ligand, giving rise to a germylium(germylene) Bi^I^ complex as suggested by DFT calculations and X-ray photoelectron spectroscopy (XPS). Likewise, migration of the positive charge of the Bi^III^I_2_ cation of **3** results in a bis(germylium)Bi^III^I_2_ complex. The delocalization of the positive charge in the ligand engenders a much higher stability of the Bi^I^ cation **4** in comparison to an isoelectronic two-coordinate Pb^0^ analogue (plumbylone; decomposition below −30 °C). Interestingly, **4**[BAr^F^] undergoes a reversible single-electron transfer (SET) reaction (oxidation) to afford the isolable Bi^II^ radical complex **5** in **5**[BAr^F^]_2_. According to electron paramagnetic resonance (EPR) spectroscopy, the unpaired electron predominantly resides at the Bi^II^ atom. Extending the redox reactivity of **4**[OTf] employing AgOTf and MeOTf affords Bi^III^(OTf)_2_ complex **7** and Bi^III^Me complex **8**, respectively, demonstrating the high nucleophilic character of Bi^I^ cation **4**.

## Introduction

Compounds containing heavy p-block group 14 and 15 elements E in uncommon low oxidation states are of paramount interest because they provide multiple new opportunities for the design of main-group element species mimicking transition-metal-like reactivity with respect to small molecule activation and catalysis.^1−5^ However, the synthesis of such isolable species is challenging and requires suitable ligation around the low-valent E atom to prevent E–E bond oligomerization and disproportionation. Recent developments in this direction have paved the way to zerovalent monatomic complexes of the group 14 elements named tetrylones with the general formula L:→E^0^←:L (E = C, Si, Ge, Sn, Pb).^6−15^ Utilizing the bis(silylene)xanthene,^16^ we achieved the whole series of heavier tetrylones previously.^7,11,13,14^ The central E^0^ atom is two-coordinated by two sufficient donor ligands and obeys the octet rule, retaining its four valence electrons as two lone pairs.^17,18^ The various tetrylones stabilized by sufficient Lewis bases show an unparalleled reactivity toward small molecules.^19−22^ Monovalent group 15 element complex cations of the type L_2_E^+^ (L = donor, E = N, P, As, Sb, Bi)^23−27^ are known isoelectronic species but particularly difficult to tame for the heaviest pnictogen, bismuth. Compared with the lighter congeners, low-valent bismuth compounds possess exceptional features such as strong spin–orbit coupling (SOC) due to relativistic effects,^28,29^ low redox potentials and transition-metal-like properties in redox catalysis.^30−37^ It should be noted here that the peculiar electronic features of low-valent Bi play also a decisive role in rare earth metal–bismuth coordination compounds that are single-molecule magnets.^38−41^

**Chart 1:**
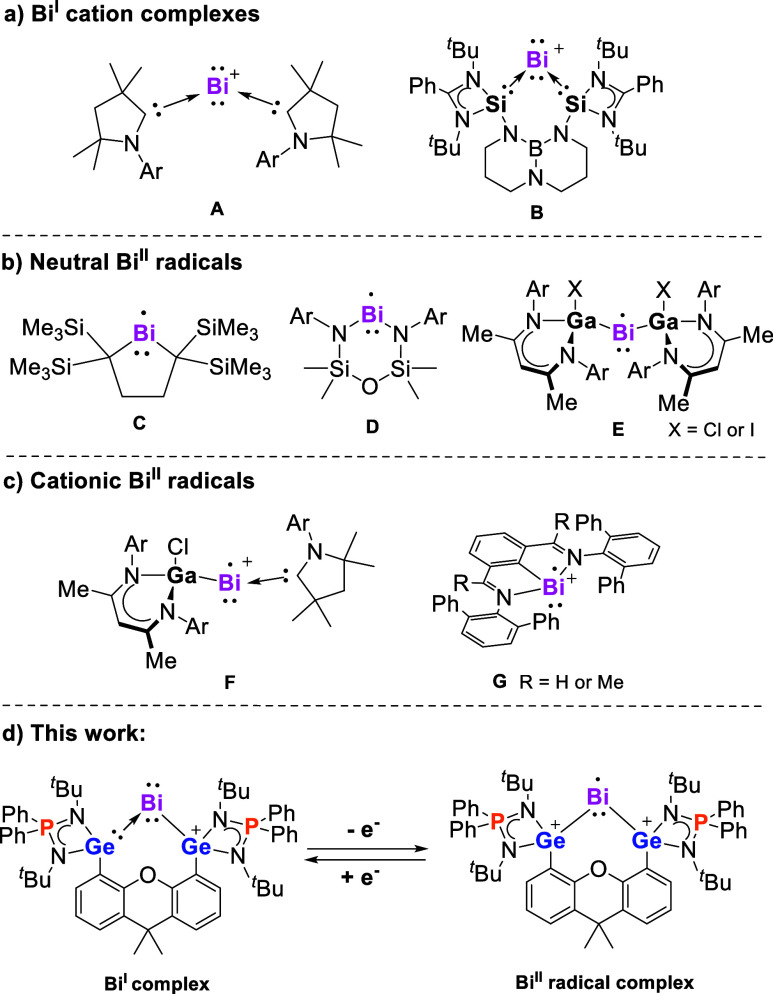
(a) Reported Examples of Bi^I^ Cation Complexes (A,B); (b) Neutral Bi^II^ Radical Complexes (C–E); (c) Cationic Bi^II^ Radical Complexes (F,G); (d) This Work: Bi^I^ and Bi^II^ Complexes with a Redox Non-innocent Bis(germylene) Liganda

Utilizing the same Lewis donor–acceptor stabilization strategy as previously applied for tetrylones, two examples of isolable two-coordinate Bi^I^ cation complexes have been synthesized, namely, cyclic (alkyl)(amino)carbene (cAAC)-supported Bi^I^ cation complex **A**(42) and bis(silylene)-supported Bi^I^ cation complex **B**^23^ (Chart 1a). **A** is only stable in ethereal solutions at relatively high concentrations under an inert atmosphere; dilution of the solution results in its decomposition. The intrinsic lability of Bi^I^ cation complexes may be attributed to the higher nucleophilicity and electropositive and redox character of the Bi^I^ site caused by notably larger, diffuse, and polarizable valence orbitals of Bi compared to those of its lighter nonmetallic congeners (N, P, As).^43,44^ These electronic features may enable Bi^I^ cation complexes to act as electron transfer reagents under the concomitant formation of Bi^II^ and Bi^III^ complexes, respectively. However, the redox reactivity of **A** and **B** and whether they are suitable precursors for Bi^II^ radical complex are currently unknown. Bi^II^ radical complexes, in turn, are also scarce.^45^ Their existence as reactive intermediates^37,46^ and transient species^47^ has been postulated in previous studies. The unequivocal identification of Bi^II^ radical complexes is challenging, in particular, the electron paramagnetic resonance (EPR) characterization due to the enormous (isotropic and anisotropic) hyperfine interactions and the large nuclear quadrupole moment of the ^209^Bi nucleus (*I* = 9/2; 100% natural abundance). Until now, only a few stable monatomic Bi^II^ radicals have been reported (Chart 1b,c).^48−53^ Among these, **C**^48^ is considered a redox radical Bi^II^/Bi^III^ couple and lacks observable EPR signals. Compounds **D**^49^ and **E**(50,51) are isolable neutral Bi^II^ radicals with the unpaired electron predominantly residing at the Bi atom. In addition, **F**^52^ and **G**^53^ represent known cationic Bi^II^ complexes.

a Ar = 2,6-Diisopropylphenyl.

In this work, we present the synthesis and characterization of unprecedented types of remarkably stable monatomic two-coordinate Bi^I^ and Bi^II^ complexes, which are stabilized by the novel chelating bis(iminophosphonamido-germylene)xanthene ligand [(P)Ge^II^(Xant)Ge^II^(P)] **1**, [(P)Ge^II^(Xant)Ge^II^(P) = Ph_2_P(N*t*Bu)_2_Ge^II^(Xant)Ge^II^(N*t*Bu)_2_PPh_2_, Xant = 9,9-dimethyl-xanthene-4,5-diyl]. The Bi^I^ complex **4** can be regarded as an isoelectronic analog of a two-coordinate Pb^0^ complex (plumbylone). In contrast to the thermolabile plumbylone analogue, however, which decomposes above −30 °C, **4** is stable even in boiling benzene most likely due to resonance stabilization: the positive charge of the Bi^I^ cation migrates to one of the germanium atoms in the ligand, giving rise to a (germylium)germylene Bi^I^ situation as suggested by density functional theory (DFT) calculations. Starting from the Bi^I^ complex **4**[BAr^F^], the single electron oxidation with ferrocenium BAr^F^ (Cp_2_FeBAr^F^, BAr^F^ = tetrakis[3,5-bis(trifluoromethyl)phenyl]borate) resulted in the formation of the isolable Bi^II^ radical complex **5**[BAr^F^]_2_ with the unpaired electron mainly located at the bismuth center, and each germanium atom features one positive charge. Oxidative reactions of **4**[OTf] with AgOTf and MeOTf (OTf = OSO_2_CF_3_) afford cationic Bi^III^ complexes, demonstrating the remarkably high nucleophilic character of Bi^I^ complex **4**.

## Results and Discussion

### Synthesis and Characterization of Monoatomic Bi^I^ Complexes 4[BAr^F^] and 4[OTf]

At first, we prepared the starting material for **1**, the *N*-heterocyclic iminophosphonamido-chlorogermylene, **(P)GeCl** [Ph_2_P(N*t*Bu)_2_GeCl], through salt metathesis reaction of iminophosphonamide with NEt_3_ and GeCl_2_·dioxane in THF at −78 °C (see the Supporting Information). The latter is characterized by NMR spectroscopy, electrospray ionization (ESI) mass spectrometry, and X-ray diffraction (XRD) analysis (see the Supporting Information). Compared to *N*-heterocyclic amidinato-chlorogermylene [PhC(N*t*Bu)_2_GeCl],^54^ the phosphorus atom in **(P)GeCl** increases the σ-donor ability of germylene due to the N^–^–P^+^ bond polarity.^55^ The latter is well supported by DFT calculations of **(P)GeCl** and related amidinato-chlorogermylene (see the Supporting Information, Figure S66).

**Scheme 1:**
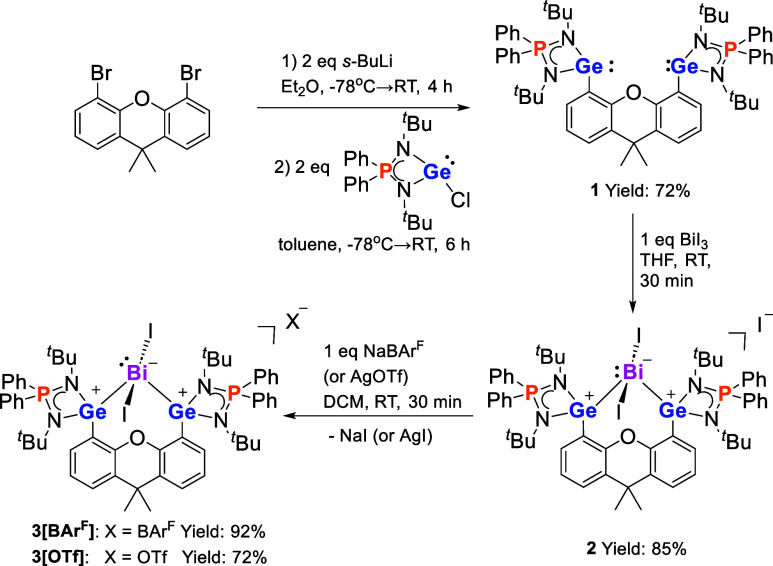
Synthesis of Bis(germylene) **1** and Bi^III^I_2_ Precursors **2** and **3**

**Figure 1:**
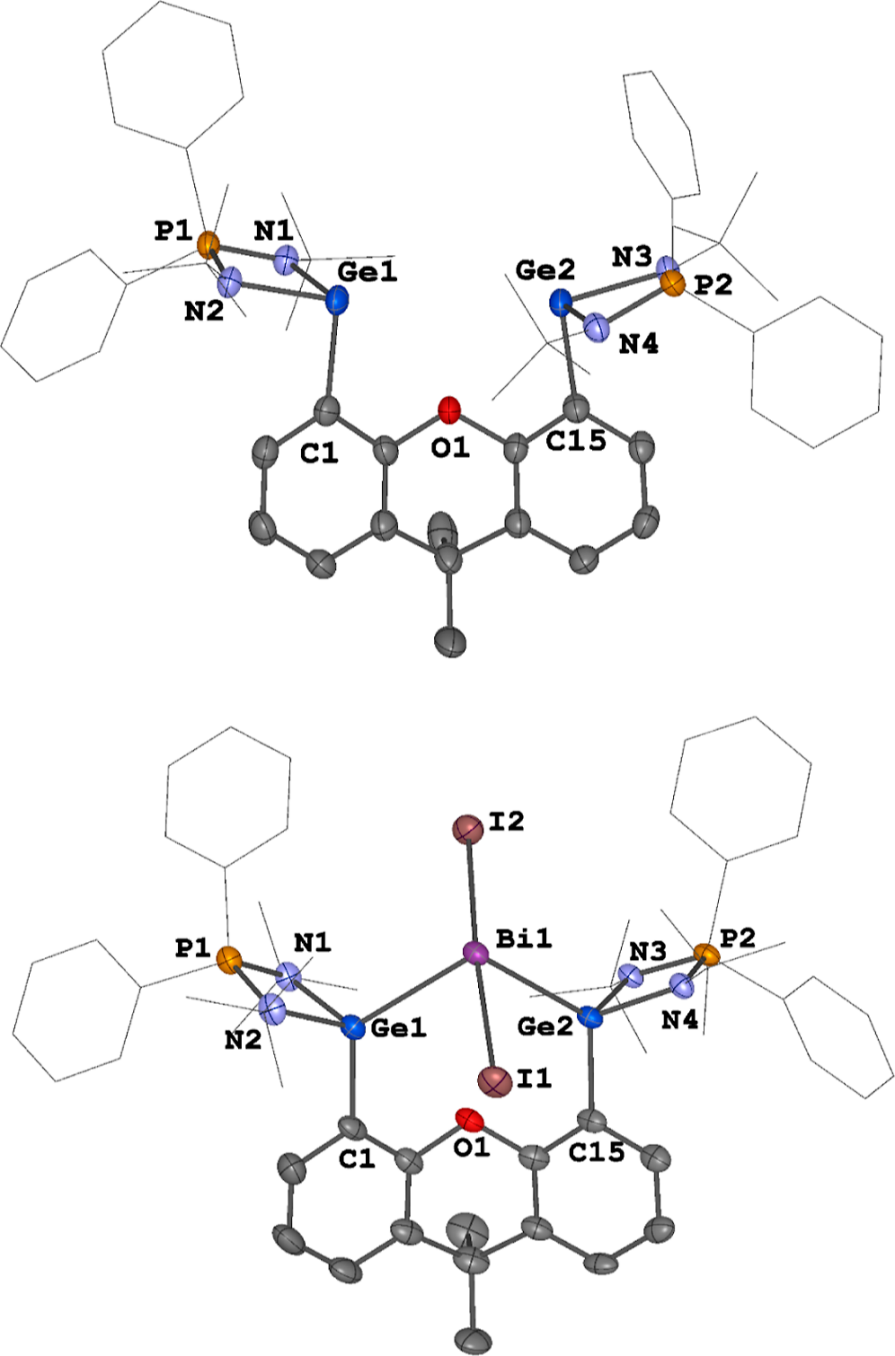
Molecular structures of **1** (top) and the cations in **2**, **3**[BAr^F^] and **3**[OTf] (bottom). Thermal ellipsoids are drawn at the 50% probability level. H atoms, anionic moieties and solvent molecules are omitted for clarity. Selected bond lengths (Å) and angles (deg): **1**: Ge1–C1 2.0607(15), Ge2–C15 2.0458(15), N1–Ge1–C1 99.11(5), N2–Ge1–C1 96.42(5), N3–Ge2–C15 99.21(5), N4–Ge2–C15 92.06(5). **2**: Bi1–Ge1 2.7578(6), Bi1–Ge2 2.7497(6), Bi1–I1 3.0921(5), Bi1–I2 2.9991(4), I1–Bi1–I2 169.779(15), Ge1–Bi1–Ge2 109.244(18). **3**[BAr^F^]: Bi1–Ge1 2.7737(5), Bi1–Ge2 2.7800(5), Bi1–I1 3.0406(3), Bi1–I2 3.0424(3), I1–Bi1–I2 172.326(10) Ge1–Bi1–Ge2 103.895(14). **3**[OTf]: Bi1–Ge1 2.8035(5), Bi1–Ge2 2.7813(5), Bi1–I1 3.0404(3), Bi1–I2 3.0679(3), I1–Bi1–I2 174.507(10), Ge1–Bi1–Ge2 106.995(16).

Starting from **(P)GeCl**, chelating ligand **1** is readily accessible in a one-pot synthesis (Scheme 1). Dilithiation of 4,5-dibromo-9,9-dimethylxanthene with 2 M equiv of *s*-BuLi in Et_2_O, followed by a salt metathesis reaction with **(P)GeCl**, afforded the desired bis(iminophosphonamido-germylene)xanthene **1** as a yellow powder in 72% yield. Its ^31^P{^1^H} NMR spectrum shows a singlet at δ 26.6 ppm. The molecular structure of **1** established by XRD analysis reveals a Ge···Ge distance of 4.071 Å, excluding attractive interaction between the two Ge atoms (Figure 1). Compared to bis(amidinato germylene)xanthene,^56^ compound **1** is a stronger chelating bis(germylene) ligand due to the P–N bond polarization mentioned above.

Treatment of **1** with 1 M equiv of BiI_3_ in THF at room temperature led to the formation of **2** as a brown powder in 85% yield. The iodide counteranion in **2** can be easily displaced by BAr^F^ and OTf anion upon mixing of **2** with 1 M equiv of NaBAr^F^ or AgOTf in dichloromethane (DCM), affording **3**[BAr^F^] (yield: 92%) and **3**[OTf] (yield: 72%) as orange powders, respectively (Scheme 1). As expected, the ^1^H NMR spectra of **2**, **3**[BAr^F^], and **3**[OTf] are practically identical. The ^11^B{^1^H} NMR spectrum of **3**[BAr^F^] shows a singlet at δ −6.6 pm, which is attributed to the BAr^F^ anion. The ^19^F{^1^H} NMR signal of **3**[OTf] (δ −78.8 ppm) indicates the presence of a “free” OTf anion.^24^ The ^31^P{^1^H} NMR spectra of **2**, **3**[BAr^F^], and **3**[OTf] exhibit a singlet at δ 54.6 ppm, which is significantly downfield shifted compared to that of **1** (δ 26.6 ppm). The molecular structures of **2**, **3**[BAr^F^], and **3**[OTf] were determined by XRD analysis (Figure 1; also see the Supporting Information). In each of these compounds, a stereoactive lone pair is present on the four-coordinate bismuth atom with a central Bi^III^ atom adopting a seesaw geometry with two iodine atoms located in axial positions (I1–Bi1–I2:169.779(15)-174.507(10)°). The Ge1–Bi1–Ge2 angles range from 103.895(14) to 109.244(18)°. It is worth noting that **2**, **3**[BAr^F^], and **3**[OTf] are the first examples of germylene Bi^III^ halide complexes.

**Scheme 2:**
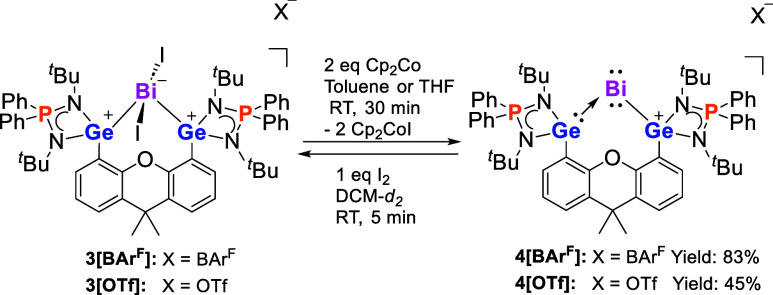
Synthesis of **4** and Reversible Interconversion of **3** and **4** with I_2_

With the cationic Bi^III^I_2_ precursors **2**, **3**[BAr^F^], and **3**[OTf] in hand, we envisioned that the monatomic Bi^I^ complex could be obtained through their reductive deiodination. The reaction of **2** with 2 M equivs of KC_8_ or {(ArNacnac)Mg^I^}_2_^57^ in THF led to decomposition of **2** most likely due to overreduction. To our delight, the reactions of **3**[BAr^F^] and **3**[OTf] with 2 M equivs of cobaltocene (Cp_2_Co) in toluene (**3**[BAr^F^]) and THF (**3**[OTf]) at room temperature gave red solutions, from which **4**[BAr^F^] and **4**[OTf] were isolated as red crystals in 83 and 45% yields, respectively (Scheme 2). Featuring the same cation moiety, the ^1^H NMR spectra of both products reveal a singlet at the same chemical shift (δ 1.08 ppm) for the *tert*-butyl groups, implying that cation **4** is symmetric and solvent is separated in solution. Accordingly, the ^31^P{^1^H} NMR spectra of **4**[BAr^F^] and **4**[OTf] exhibit a singlet at the same chemical shift of δ 44.5 ppm, significantly upfield shifted compared to those of **2** and **3** (δ 54.6 ppm), but downfield shifted compared to that of **1** (δ 26.6 ppm), respectively. The ^11^B{^1^H} NMR spectrum of **4**[BAr^F^] shows a sharp singlet at δ −6.6 ppm, corresponding to the weakly coordinating BAr^F^ anion, and the ^19^F{^1^H} NMR spectrum of **4**[OTf] shows a signal at δ −78.9 ppm for the “free” OTf anion.^24^ Notably, a two-electron oxidation of **4** with I_2_ in DCM-*d*_2_ after 5 min at room temperature restored compound **3** in quantitative yield.

**Figure 2:**
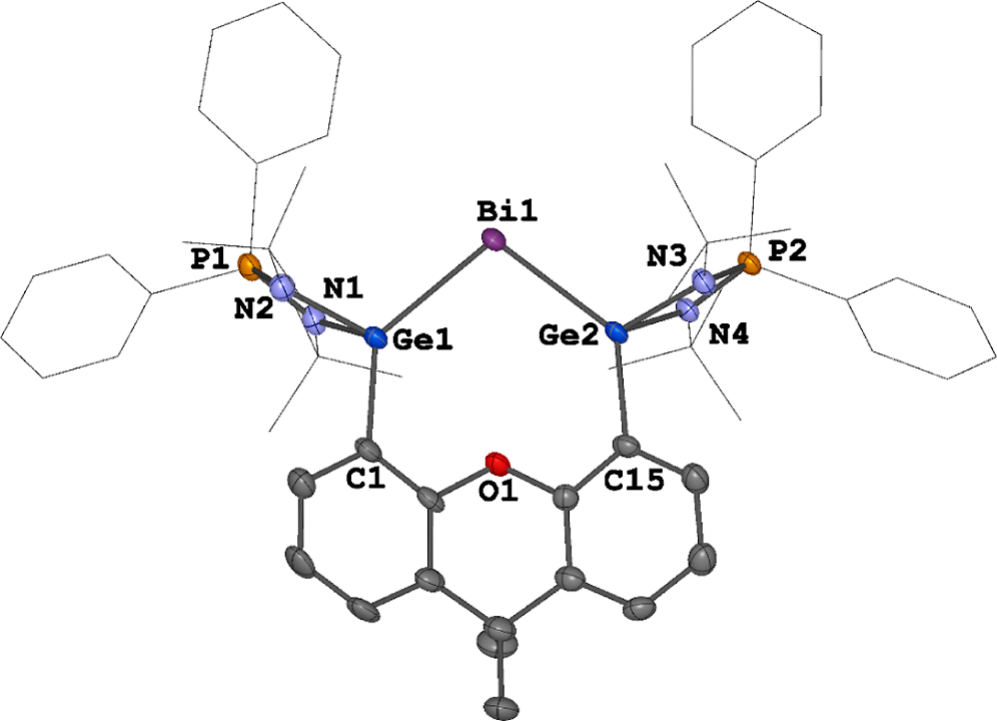
Molecular structures of the cation **4** in **4**[BAr^F^] and **4**[OTf]. Thermal ellipsoids are set at the 50% probability. Hydrogen atoms, counteranions and solvent molecules are omitted for clarity. Selected distances (Å) and angles (deg): **4**[BAr^F^]: Bi1–Ge1 2.6672(4), Bi1–Ge2 2.6627(4), Ge1–Bi1–Ge2 103.981(12). **4**[OTf]: Bi1–Ge1 2.6693(9), Bi1–Ge2 2.6712(9), Ge1–Bi1–Ge2 104.67(3).

**4**[BAr^F^] crystallized in the triclinic space group *P*1̅, while **4**[OTf] crystallized in the orthorhombic space group *Pbca*. Both structures were elucidated by XRD analysis and exhibit separated ion-pair structures with an almost identical Bi^I^ complex (Figure 2). The central Bi^I^ site is bonded to the two Ge atoms with Ge–Bi distances ranging from 2.6627(4) to 2.6712(9) Å, the latter are significantly shorter than those observed in **3** [2.7737(5)–2.8035(5) Å], which is consistent with the decrease of the coordination number of the Bi atom in **4** compared to its precursor **3**. The Ge1–Bi1–Ge2 angles of 103.981(12)° (**4**[BAr^F^]) and 104.67(3)° (**4**[OTf]) are quite similar to those of **3**[BAr^F^] (103.895(14)°) but slightly smaller than those of **2** (109.244(18)°) and **3**[OTf] (106.995(10)°), respectively. However, the Ge1–Bi1–Ge2 angles are significantly larger than the Si1–Bi1–Si2 angle of the Bi^I^ cation complex **B** (82.10(3)°) due to the larger ring size.^23^

### Single-Electron Transfer Reactions

**Scheme 3:**
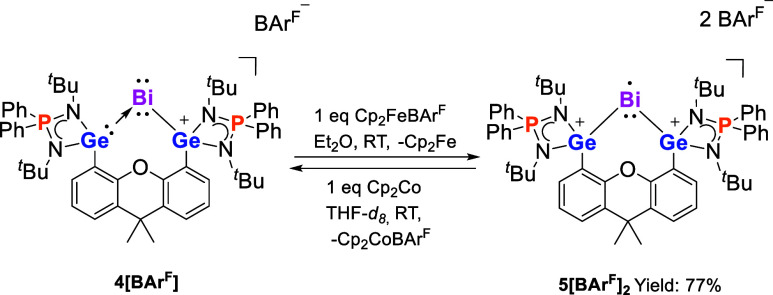
Reversible Interconversion of **4**[BAr^F^] and **5**[BAr^F^]_2_

Owing to the presence of two lone pairs of electrons on the Bi^I^ center in **4**, we envisaged that **4** is a suitable precursor for the synthesis of Bi^II^ and Bi^0^ radical complexes via single-electron oxidation/reduction reactions. This is supported by the cyclic voltammetry (CV) analysis of compound **4**[BAr^F^]. The CV exhibits two quasi-reversible oxidation processes at *E*_1/2_ ≈ −0.54 V and −0.09 V, followed by a third irreversible oxidation wave at *E*_p,a_ = 0.66 V vs Fc/Fc^+^ and an irreversible reduction wave at *E*_p,c_ = −1.53 V vs Fc/Fc^+^ (see the Supporting Information, Figure S29). To examine this hypothesis, we carried out single-electron oxidation reactions of **4**[BAr^F^]. The latter was reacted with 1 M equiv of Cp_2_Fe[BAr^F^] in Et_2_O, furnishing the desired Bi^II^ radical complex **5**[BAr^F^]_2_ in 77% yield (Scheme 3). Complex **5**[BAr^F^]_2_ is a brownish powder in the solid state and highly air-sensitive, but it can be stored under a N_2_ atmosphere at −30 °C for several weeks. In addition, treatment of **5**[BAr^F^]_2_ with 1 M equiv of Cp_2_Co in THF for 5 min at room temperature restored compound **4**[BAr^F^] in quantitative yields. We also conducted the single-electron reduction of **4**[BAr^F^] in an attempt to synthesize neutral Bi^0^ compound **6**. When one molar equiv of Cp_2_Co or KC_8_ was reacted with **4**[BAr^F^] in THF at −78 °C, the reaction ultimately resulted at ambient temperature in formation of elemental bismuth as a precipitate due to decomposition.

**Figure 3:**
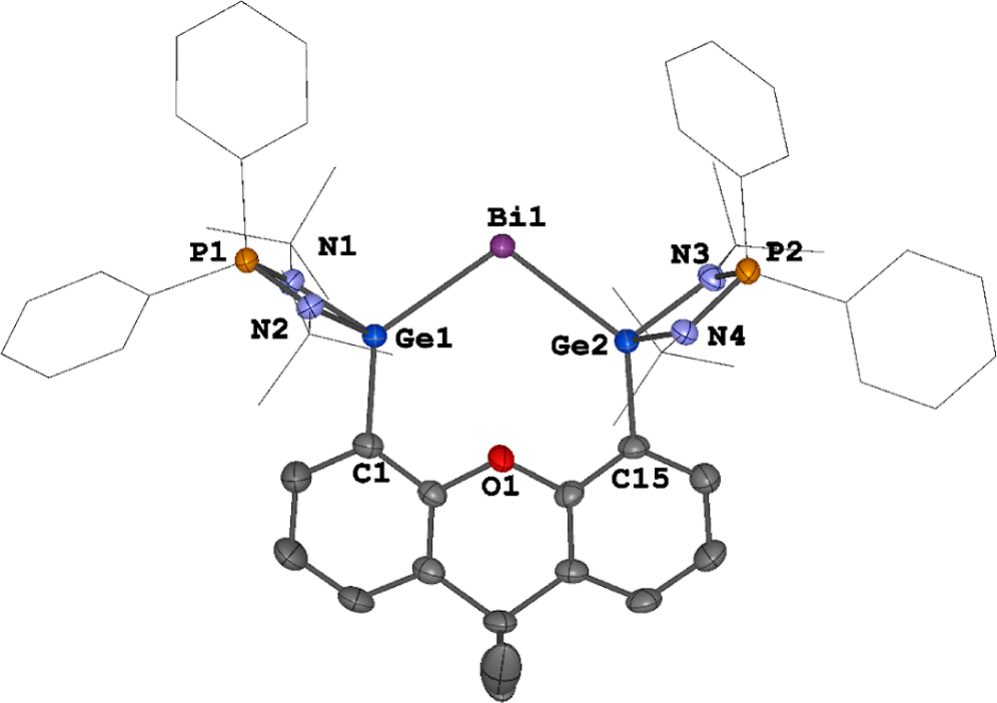
Molecular structure of the radical dication **5** in **5**[BAr^F^]_2_. Thermal ellipsoids are set at the 50% probability. Hydrogen atoms, anionic moieties and solvent molecules are omitted for clarity. Selected bond lengths (Å) and angles (deg): Bi1–Ge1 2.7112(3), Bi1–Ge2 2.7147(3), Ge1–Bi1–Ge2 107.583(9).

XRD analysis revealed that compound **5**[BAr^F^]_2_ is an ion triple that crystallizes in the triclinic space group *P*1̅ and contains the monatomic Bi^II^ moiety **5** as shown in Figure 3. Similar to **4**[BAr^F^], **5**[BAr^F^]_2_ features a two-coordinate Bi^II^ center, but there are two separated BAr^F^ counteranions present in the molecular structure, indicating that **5**[BAr^F^]_2_ is a Bi^II^ radical complex. The Ge1–Bi1–Ge2 angle of 107.583(9)° in **5**[BAr^F^]_2_ is slightly larger than that of **4** in **4**[BAr^F^] (103.981(12)°) and **4**[OTf] (104.67(3)°). Additionally, the Ge–Bi distances (2.7112(3) and 2.7147(3) Å) of **5**[BAr^F^]_2_ are slightly longer than those of **4** (2.6627(4)–2.6712(9) Å) but shorter than those of **3** (2.7737(5)–2.8035(5) Å), presumably due to the weaker π-back-donation from the Bi^II^ center to both Ge atoms in **5**[BAr^F^]_2_.

Compound **5**[BAr^F^]_2_ is paramagnetic and shows broad resonance peaks in the solution ^1^H NMR spectrum at room temperature (see the Supporting Information, Figure S37). The effective magnetic moment (μ_eff_) of a microcrystalline solid sample, measured with a superconducting quantum interference device (SQUID) magnetometer, is temperature-dependent (see the Supporting Information, Figure S60). The magnetic moment linearly decreases with decreasing temperature and shows a value of 1.98 μ_B_ at 300 K, which is comparable to reported Bi^II^ radical compounds.^49,50,53^ The latter value is consistent with a single unpaired electron *S* = 1/2 system.

**Figure 4:**
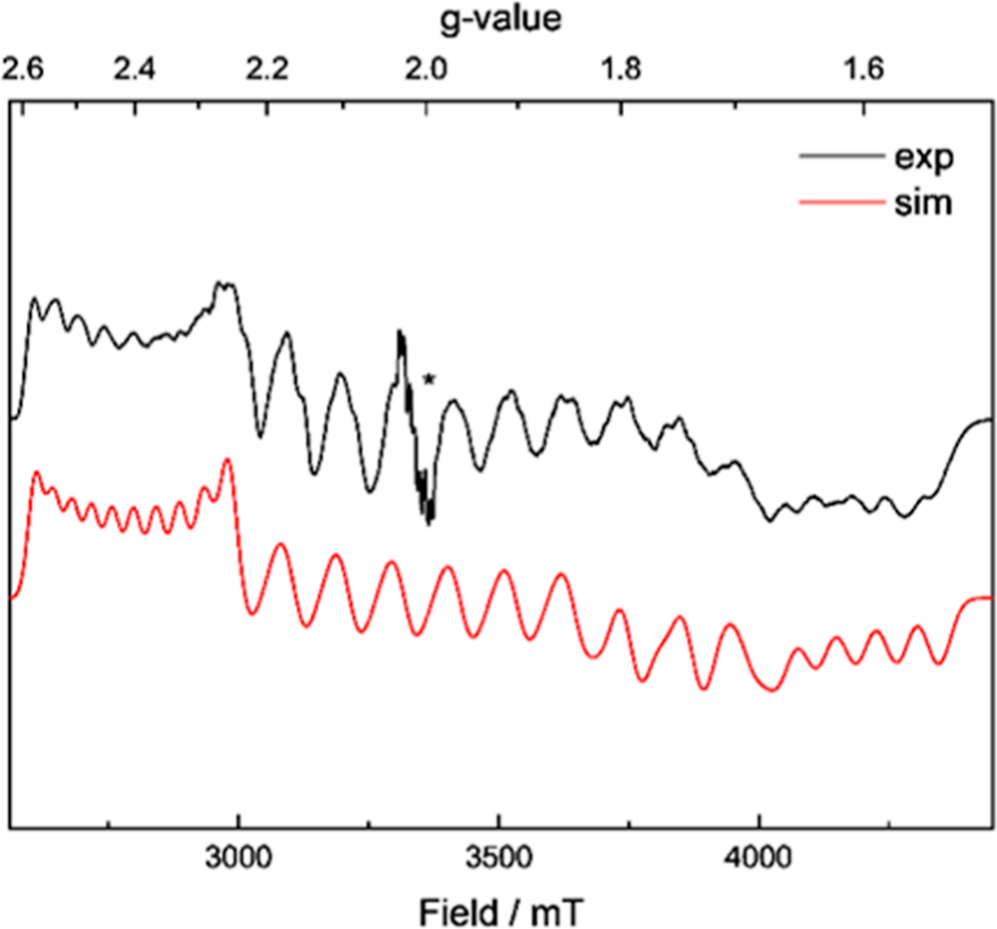
Pseudomodulated W-band field swept echo EPR-spectrum of **5**[BAr^F^]_2_ recorded at 10 K. The experimental spectrum is displayed as black and the corresponding simulation as red line. The *g*-matrix derived from the simulation is *g* = [2.39, 1.92, 1.66] and the hyperfine coupling *A* = [1370, 2920, 1650] MHz. The signal labeled by an asterisk is related to an impurity from manganese.

EPR spectroscopy was applied to explore the electronic nature of the Bi^II^ radical complex **5**[BAr^F^]_2_ in more detail. The continuous-wave X-band (9 GHz) and pseudomodulated Q-band field swept echo (34 GHz) spectra, respectively (see the Supporting Information, Figure S61), exhibit a very broad multiline EPR signal with components spanning the whole experimentally accessible spectral range. As a consequence of the typically very strong hyperfine coupling of ^209^Bi (I = 9/2) in the GHz range, yet moderate g-anisotropy, the number of EPR transitions monitored at X-band and Q-band frequencies are limited and much higher magnetic field is required to decouple g-matrix and A-tensor and completely resolve the EPR spectrum. Thus, we used W-band (94 GHz) EPR and obtained a quite well-resolved multiplet of lines with recognizable g-anisotropy, yielding for each g-component 10 lines, which partially overlap with each other (Figure 4). The simulation of the experimental data resulted in the following g-matrix principal values of *g* = [2.39, 1.92, 1.66] for an *S* = 1/2 system and the hyperfine tensor principal values *A* = [1370, 2920, 1650] MHz, with the largest A-component along the intermediate *g*-component. The average g value (*g*_av_ = (*g*_1_ + *g*_2_ + *g*_3_)/3) of **5**[BAr^F^]_2_ is *g*_av_ = 1.99 and rather close to the free electron g, as observed for the gallium-stabilized Bi radicals **E** and **F**^58^, but distinctly different from the nitrogen-coordinated Bi radicals **D**^49^ and **G**^53^, which show a *g*_av_ significantly smaller than 2. All EPR values of **G** refer only to the protonated compound (R = H); the methylated variant (R = Me) has almost identical values. The symmetry of the hyperfine tensor of **5**[BAr^F^]_2_ corresponds to that of **E** and **F**,^58^ which is also aligned with its largest component along the intermediate g-component. In contrast, the hyperfine tensors reported for **D**^49^ and **G**^53^ are aligned with their largest component along the smallest g-component. The magnitude of the hyperfine coupling of **5**[BAr^F^]_2_ with an average value (*A*_av_ = (*A*_1_ + *A*_2_ + *A*_3_)/3) *A*_av_ = 1980 MHz is intermediate between the smaller *A*_av_ = 1383 MHz and *A*_av_ = 1650 MHz of **E** and **F**,^58^ respectively, and the larger *A*_av_ = 3799 MHz and *A*_av_ = 3180 MHz reported for **D**^49^ and **G**^53^, respectively. The axial part *T* of the hyperfine coupling (*T* = ((*A*_1_ + *A*_3_)/2 – *A*_iso_)) found here for **5**[BAr^F^]_2_ with *T* = −470 MHz is in its absolute value again clearly larger than that found for **E** and **F** (−245 MHz and −333 MHz)^58^ and very similar to the axial parts given for **D**^49^ and **G**^53^ with *T* = −498 MHz and *T* = −462 MHz, respectively. Following the parameters, *A*_av_ and *T* can be used to estimate the spin density in the Bi 6s and 6p orbitals according to Morton and Preston.^59^ The *A*_av_ = 1980 MHz of **5**[BAr^F^]_2_ yields with the *A* = 77,530 MHz^59^ for a 100% occupied Bi 6s (obtained assuming *g* = 2.0023) a population P_6s_(Bi) ≅ 0.03. The *T* = −470 MHz of **5**[BAr^F^]_2_ on the other hand yields with the *P* = 1659 MHz^59^ for a 100% occupied Bi 6p orbital and the angular factor −2/5 a population *P*_6p_(Bi) ≅ 0.71. Thus, the SOMO orbital of **5**[BAr^F^]_2_ has predominant 6p character with only a small admixture of 6s in agreement with the DFT results (see below) and the findings for the other Bi radicals **D**, **E**, **F** and **G**.^49−53,58^ A total Bi spin population of about 3/4 is deduced from the EPR spectrum, suggesting substantial spin delocalization into the ligand as reported also for **E** and **F**.^58^ Significantly less spin delocalization is reported for **D**^49^ and **G**^53^. This might at first sight contradict the small difference between the *T*-values of **5**[BAr^F^]_2_ and **G**^53^ with −470 and −462 MHz, but can immediately be explained by the significantly different *g*_av_-values (1.99 and 1.79, respectively) of the two radicals. The larger *g*_av_ of **5**[BAr^F^]_2_ induces a larger magnetic moment, which partially compensates for the higher Bi spin density in **G** for the resulting hyperfine couplings. This effect explains the significantly different P_6p_(Bi) values deduced for **5**[BAr^F^]_2_ and **G** at rather similar *T* values. The interpretation of the different total P(Bi) spin densities for the complexes in terms of spin delocalization into the ligands has to be taken with care since the analysis following Morton and Preston^59^ is based on the assumption of atomic Bi 6s and 6p orbitals and can only give estimates. Quantitative comparison with DFT data requires information about ligand hyperfine couplings. Unfortunately, the direct neighbor Ge of Bi in **5**[BAr^F^]_2_ has only a relatively low abundance with ^73^Ge (7.76%) and a high spin stable isotope with *I* = 9/2, which is a rather bad reporter for spin delocalization into the ligand, and produces no discernible splitting in the EPR spectrum, making hyperfine selective methods necessary for obtaining further information.

### DFT Calculations

DFT calculations at the BP86-D3(BJ)/def2-TZVP level were performed to shed light on the electronic structure, stability, and chemical bonding of monocation **4**, and radical dication **5**. For completeness, we also calculated the hypothetical neutral analogue Bi^0^ compound **6**. Figure S71 shows the calculated geometries of the three compounds and the most important bond lengths and angles. It is interesting to note that the calculated Bi–Ge distances in neutral **6** (2.654 Å) stay nearly the same in cation **4** (2.655 Å) but are slightly longer in radical dication **5** (2.715 Å). The cation **4** has an electronic singlet ground state with a triplet state of 29.6 kcal mol^–1^ higher in energy, while **5** and **6** have doublet ground states where the quartet states are higher in energy by 47.7 and 29.3 kcal mol^–1^, respectively. The theoretical values for **4** and **5** are in very good agreement with the experimental data.

The energy minimum structure of neutral **6** has *C*_*s*_ symmetry. The geometry optimizations of **4** and **5** gave structures which are slightly distorted from *C*_*s*_ symmetry. Calculations with enforced *C*_*s*_ symmetry gave structures which have very small imaginary frequencies (2.4*i* for **4** and 9.4*i* for **5**) even after using a superfine grid. The energy difference between the *C*_*s*_ structures and the energy minima are >0.1 kcal mol^–1^ for **4** and 0.4 kcal mol^–1^ for **5** and the differences in the bond lengths and angles are negligible. We decided to use the *C*_*s*_ structures shown in Figure S71 (see the Supporting Information) for the bonding analysis for simplicity, which does not affect our conclusion.

The energetically most favorable reaction pathways for rupture of the Ge–Bi bonds in **4**, **5** and **6** suggest that bismuth dissociates always as neutral Bi atom even from radical dication **5**. This is in line with the calculated charge distribution. The NBO atomic charges on Bi are −0.23*e* in **4** and 0.26*e* in **5**. Notably, the partial charge on Bi in neutral **6** (−0.25*e*) is nearly the same as that in cation **4**. The calculated values of the bond dissociation energy (BDE) are *D*_e_ = 62.2 kcal mol^–1^ for neutral **6**, *D*_e_ = 87 kcal mol^–1^ for cation **4** and *D*_e_ = 64.1 kcal mol^–1^ for the radical dication **5**. The zero-point energy (ZPE) and thermal corrections (see Supporting Information, Figure S71) suggest that all three compounds are thermodynamically stable with respect to liberation of Bi^0^. It is amazing that the BDE for the dissociation of neutral **6** and radical dication **5** has nearly the same value. The difficulty in isolating neutral **6** can be explained by its very low ionization potential (IP). Notably, the calculated adiabatic IP for **6** is only 3.55 eV at the BP86-D3(BJ)/def2-TZVP level, which is even much less than for the cesium atom (4.04 eV at the BP86-D3(BJ)/def2-TZVP), 3.89 eV experimentally.^60^

**Figure 5:**
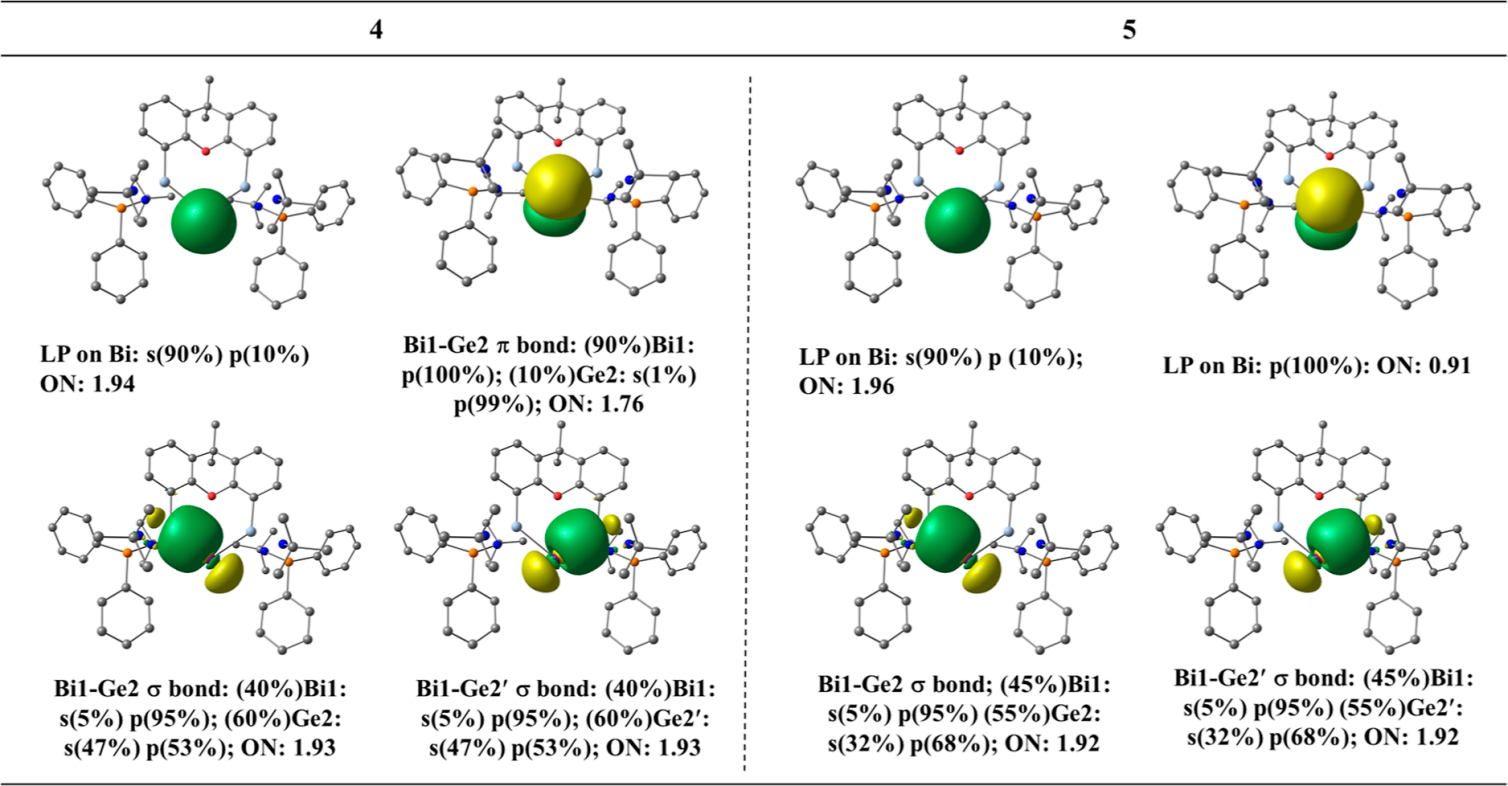
Natural orbitals and their AO compositions of cation **4** (left) and the radical dication **5** (right).

**Figure 6:**
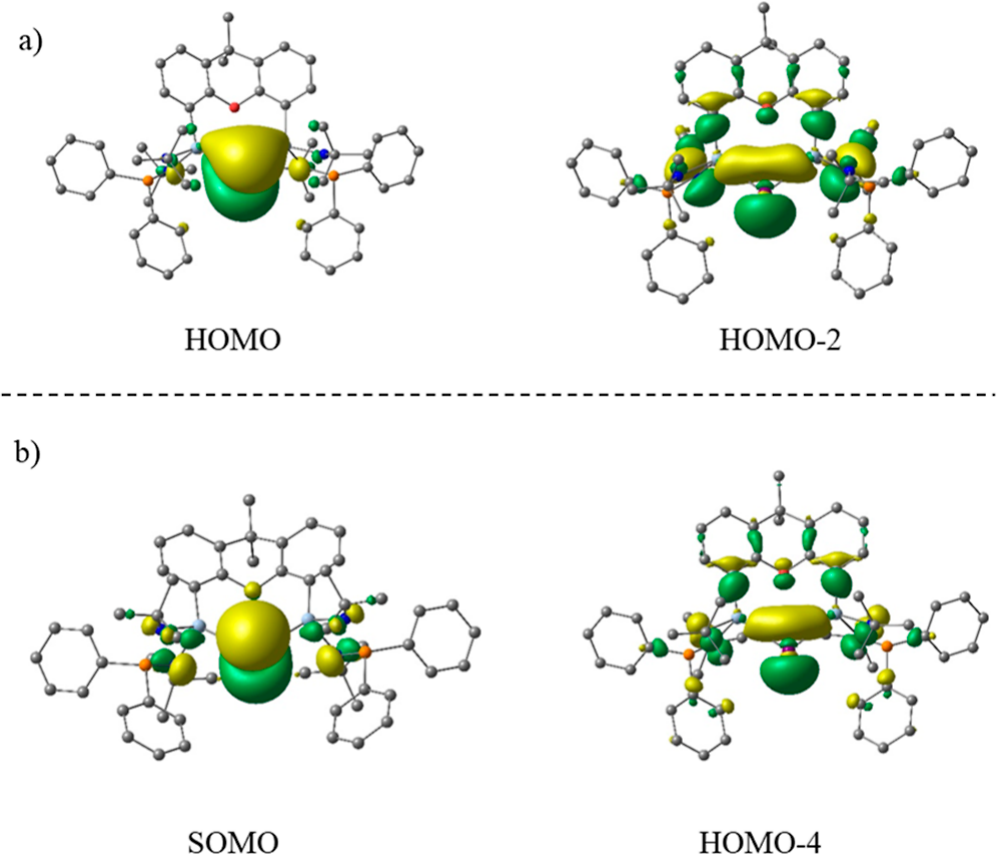
Molecular orbitals of cation **4** (a) and radical dication **5** (b).

We analyzed the bonding situation in **4** and **5** in more detail using a variety of methods. Figure 5 shows the natural orbitals that are related to the valence state of Bi in the two compounds. In **4**, there is a σ lone pair orbital at Bi with 90% s character and a strongly polarized Bi–Ge π orbital, which is 90% localized at Bi that mimics a π lone-pair orbital. There are two Bi–Ge σ-bonding orbitals which are slightly polarized toward Ge. The Bi atom in **4** has two lone-pair orbitals. The HOMO and HOMO – 2 of **4** correspond to a π-type and a σ-type lone pair at the Bi^I^ center, respectively, which is a characteristic feature of ylidones L→E←L, the heavy-atom homologues of carbones L→C←L (Figure 6a).^18,61−64^ The orbitals of the radical dication **5** are very similar but the π lone-pair orbital is occupied by only one electron, which may better be named as “lone single orbital” (Figure 6b). The NBO spin density of 0.87*e* on Bi also supports this assignment (see the Supporting Information, Figure S74 for the spin density plot). The unpaired electron in a π-type orbital is in agreement with the EPR data, showing a small s character and a predominant p orbital character of the unpaired electron.

**Scheme 4:**
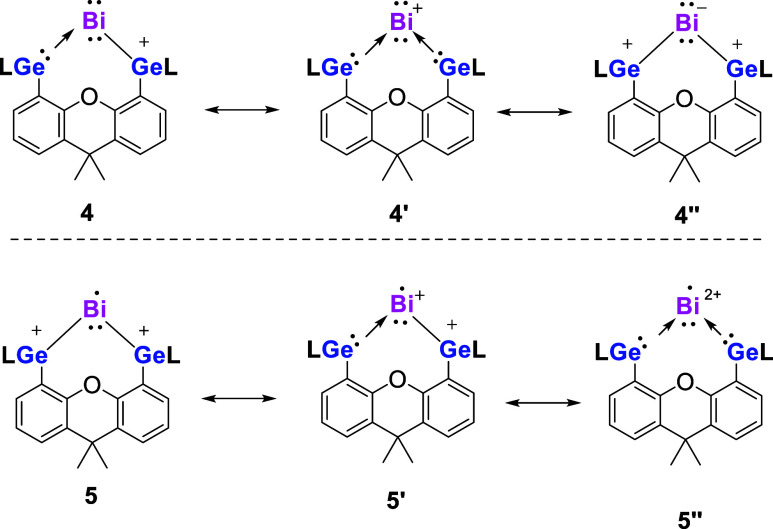
Main Resonance Structures of Cation **4** and Radical Dication **5**, Respectively L = Ph_2_P(N*t*Bu)_2_.

The NBO orbitals of **4** and **5** support the Lewis structures shown in Scheme 4. A more detailed analysis using the EDA-NOCV method reveals a bonding situation that is more complex than that described by the Lewis structure. We carried out EDA-NOCV calculations of **4** and **5** using Bi and the bis(germylene) ligand L with different charges and different electron configurations (see the Supporting Information, Tables S25 and S26). Table 1 shows the numerical results with the fragments that give the smallest energy values for the orbital interaction Δ*E*_orb_, indicating the most favorable moieties for describing the bonding situation.^65−70^ It turned out that the best description of the L(Ge)-Bi bonds in **4** and **5** has neutral Bi and singly or doubly charged ligand in the given electron configurations, which agrees with the calculated partial charges given by the NBO method. The electronic reference states of Bi are the ^2^D excited state for **4** and the ^4^S ground state for **5** whereas the ligands L^+^ and L^2+^ have doublet and triplet states, respectively.

**Table 1:** Results of EDA-NOCV Calculations of Cation **4** and Radical Dication **5** at the BP86-D3(BJ)/TZ2P-ZORA//BP86-D3(BJ)/def2-TZVP Level Using the Most Favorable Fragment Partitioning Schemes^a^

energy	orbital interaction	**4** Bi (^2^D, 6s^2^6p_π⊥_^2^6p_σ_^1^6p_π∥_^0^) + L^+^ (doublet)	**5** Bi (^4^S, 6s^2^6p_π⊥_16p_σ_^1^6p_π∥_^1^) + L^2+^ (triplet)
Δ*E*_int_		–147.0	–124.6
Δ*E*_Pauli_		204.2	240.3
Δ*E*_disp_^b^		–26.2 (7.5%)	–27.3 (7.5%)
Δ*E*_elstat_^b^		–154.8 (44.1%)	–171.7 (47.1%)
Δ*E*_orb_^b^		–170.2 (48.5%)	–165.9 (45.5%)
Δ*E*_orb(1)_^c^	Ge—Bi(6pσ)—Ge electron-sharing (+,+) σ bond	–81.0 (47.6%)	–69.2 (41.7%)
Δ*E*_orb(2)_^c^	Ge→Bi(6p_π∥_)←Ge (+,−) σ donation	–61.3 (36.0%)	
Δ*E*_orb(2)_^c^	Ge—Bi(6p_π∥_)—Ge electron-sharing (+,−) σ bond		–77.1 (46.5%)
Δ*E*_orb(3)_^c^	Ge←Bi(6p_π⊥_)→Ge π-backdonation	–17.6 (10.3%)	–6.0 (3.6%)
Δ*E*_orb(rest)_^c^		–10.3 (6.1%)	–13.6 (8.2%)

a Energy values are given in kcal mol^–1^.

b The percentage contribution with respect to total attraction is given in parentheses.

c The percentage contribution in parentheses is given with respect to total orbital interaction.

**Figure 7:**
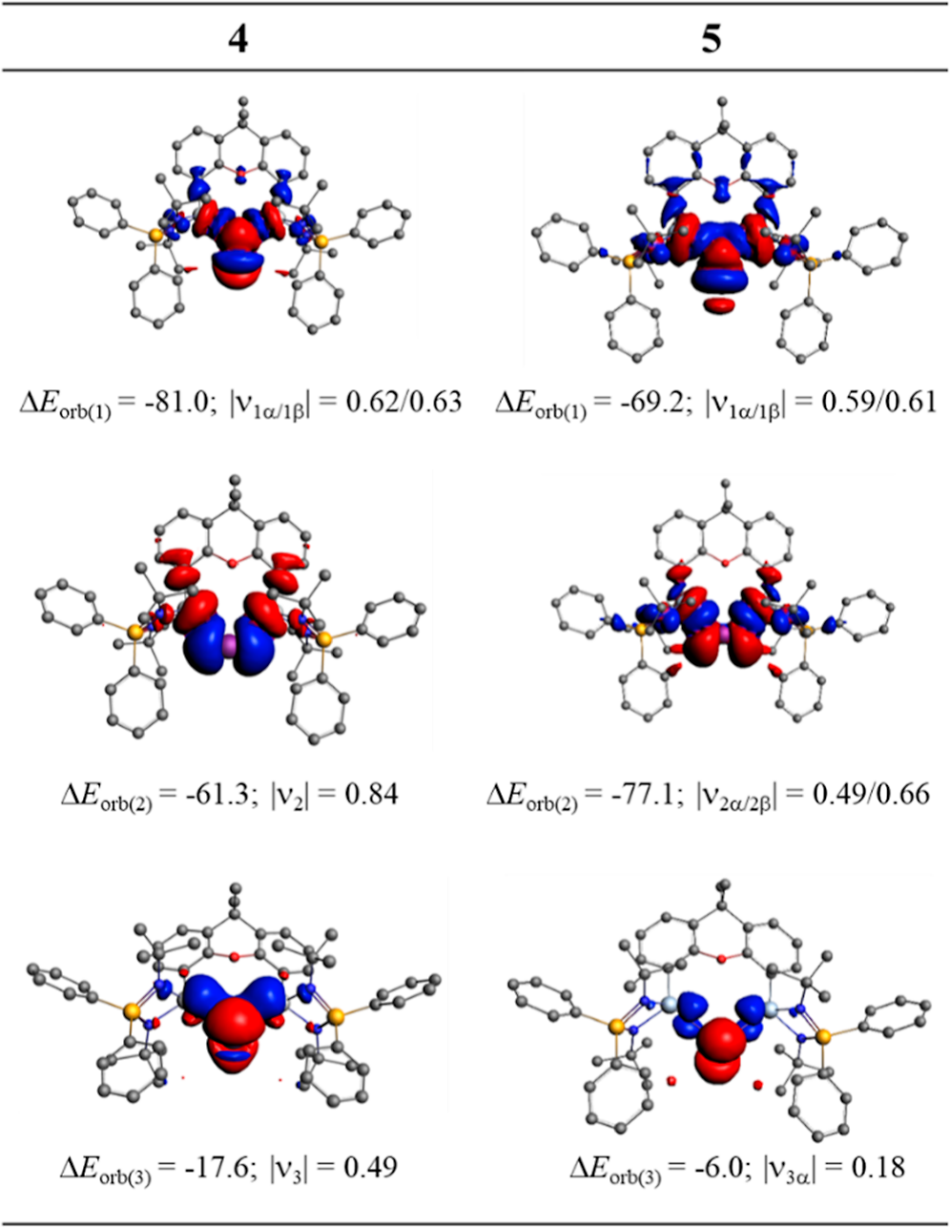
Plot of the deformation densities Δρ_(1)_–Δρ_(3)_ which are associated with the pairwise orbital interactions Δ*E*_orb(1)_–Δ*E*_orb(3)_ of cation **4** and radical dication **5**. The eigenvalues ν are a measure for the relative amount of charge transfer. The direction of the charge flow is red → blue.

The breakdown of the total orbital interactions Δ*E*_orb_ in pairwise contributions shows that in **4** and **5** there are two large σ terms Δ*E*_orb(1)_ and Δ*E*_orb(2)_ and one weaker π term Δ*E*_orb(3)_, which can be identified by inspecting the associated deformation densities displayed in Figure 7. The radical dication **5** has two electron-sharing σ Ge–Bi–Ge interactions and one very weak π Ge←Bi→Ge back-donation of the singly occupied π orbital at Bi. This supports the bonding, as suggested by the NBO method and depicted by the Lewis structure shown in Scheme 4. The EDA-NOCV results for cation **4** suggest that the two Ge–Bi–Ge σ bonds actually arise from one dative interaction into the vacant 6p_π∥_ AO of Bi Ge→Bi(6p_π∥_)←Ge (+,−) but the second σ bond comes from electron-sharing interactions of the singly occupied 6p_σ_ AO of bismuth, Ge–Bi(6p_σ_)—Ge. Such a detail of the interatomic interactions cannot adequately be sketched by a Lewis structure. It does not invalidate the description given by the Lewis structure shown in Scheme 4 but underlines the fact that the actual interatomic interactions are more complex than those suggested by Lewis formulas. We thus propose the relevance of the main resonance structures **4′**, **4″**, **5′** and **5″** (Scheme 4). The increase in the oxidation states of the Ge atoms in **4** and **5** is in line with the Ge(3d) binding energies obtained by X-ray photoelectron spectroscopy (XPS) (see Supporting Information, Figure S64). In fact, the values of **4** (32.38 eV) and **5** (32.48 eV) are significantly blue-shifted in comparison with that of the Ge^II^ atoms in the “free” bis-germylene **1** (30.88 eV).

Detailed inspection of the deformation densities reveals further interesting electronic information. The charge flow red → blue associated with Δ*E*_orb(1)_ shows an area charge accumulation at Bi for both compounds **4** and **5** that stems from the hybridization of the 6s/6p_σ_ AOs.

**Figure 8:**
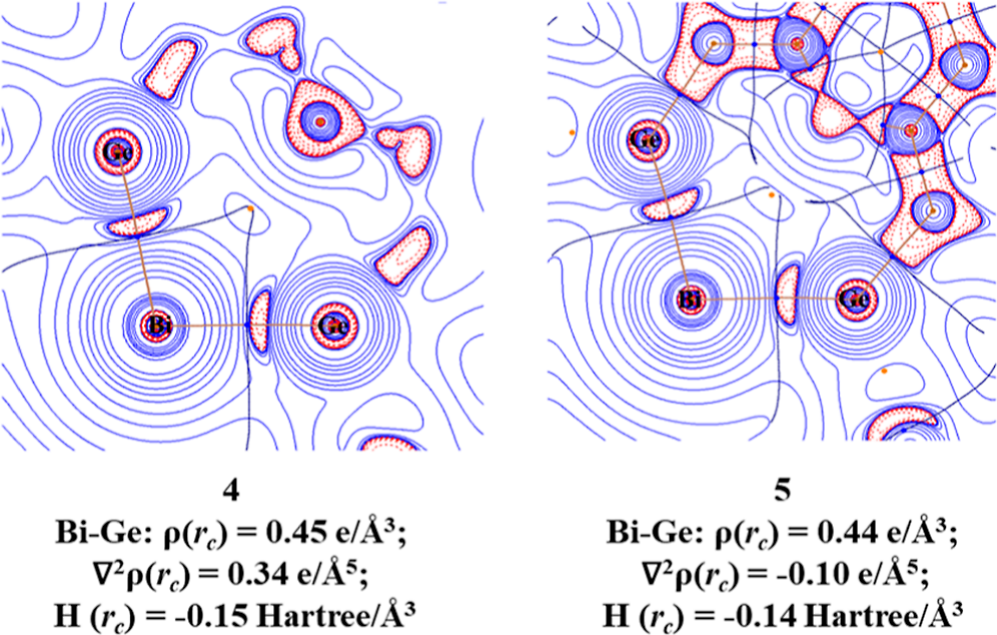
Contour plot of the Laplacian of electron density of cation **4** and radical dication **5** in the Ge–Bi–Ge plane calculated at the BP86-D3(BJ)/def2-TZVP level. Red lines indicate areas of charge concentration [∇^2^ρ(*r*) < 0] and blue lines show areas of charge depletion [∇^2^ρ(*r*) > 0].

We also analyzed the electronic charge in **4** and **5** with the quantum theory of atoms in molecules (QTAIM) method developed by Bader.^71^Figure 8 shows the Laplacian distribution of electron density ∇^2^ρ(*r*) in the Ge–Bi–Ge planes of the two compounds. As expected, there are bond critical points (BCPs) for the Bi–Ge bonds where the areas of charge accumulation (∇^2^ρ(*r*) < 0, indicated by red dotted lines) are closer to the Ge atoms, signaling a polarization of the bonds toward Ge. The negative values of the energy density *H*(r_c_) at the BCPs are characteristic of covalent interactions.^72^ Note that there is no area of charge accumulation for the σ lone-pair electrons at Bi, because the 6s AO of the latter is very diffuse.

We carried out further calculations on the precursor **3** precursor. The computed distances and angles are in good agreement with the experimental data (Supporting Information, Figure S67). Remarkably, the NBO analysis revealed that the BiI_2_ moiety carries an overall negative charge of −0.45*e* (Bi: 0.46*e*; I1: −0.48*e*; I2: −0.43*e*) and the ligand has a positive charge of +0.55*e*. EDA-NOCV calculations using the fragments BiI_2_ and the ligand L with charges and electronic state showed that the best description is given by BiI_2_^–^ (ate-type) and L^2+^ (T) in the electronic triple state, which give two electron-sharing Bi-L single bonds (see the Supporting Information, Table S24, Figure S68). This agrees with the shape of the natural orbitals of **3** suggested by the NBO analysis (see the Supporting Information, Figure S69). A lone-pair orbital at Bi is found in the NBO analysis, and it also appears as dominant part of the HOMO of **3** (see the Supporting Information, Figure S70). The EDA-NOCV and NBO methods suggest that the best description of the bonding situation of **3** is sketched as in Scheme 2.

### Reactivity of Bi^I^ Complex 4[OTf] toward AgOTf

**Scheme 5:**
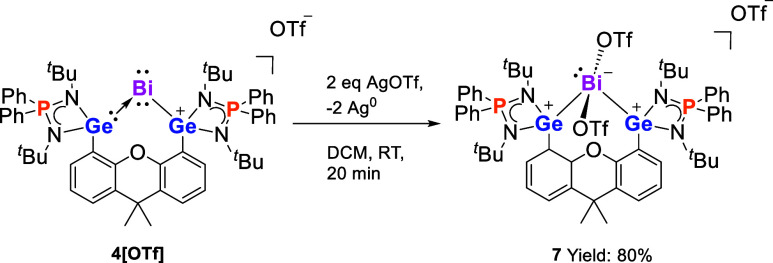
Reaction of **4**[OTf] with AgOTf

Interestingly, the CV of **4**[OTf] exhibits two quasi-reversible oxidation processes at *E*_1/2_ ≈ −0.46 V and −0.1 V and an irreversible oxidation wave at *E*_p,a_ = 0.44 V vs Fc/Fc^+^ (see the Supporting Information, Figure S36). Upon adding 1 M equiv of AgOTf to **4**[OTf] in DCM, only half of **4**[OTf] was consumed. NMR monitoring showed that a new compound was formed in the solution along with a black metallic precipitate. We thus speculated that a two-electron oxidation reaction occurred and compound **7** was furnished (Scheme 5). When two molar equivalents of AgOTf were added, nearly all of **4**[OTf] was converted to **7**. After workup, **7** was isolated as a yellow powder in 80% yield. The ^31^P{^1^H} NMR spectrum of **7** displays a singlet at δ = 58.9 ppm. The ^19^F{^1^H} NMR spectrum shows two signals at δ = −76.9 and δ = −78.6 ppm, indicating that the OTf anions have two distinct chemical environments. Notably, a lighter congener Sb^I^ cation undergoing Sb^I^→Ag^I^ coordination with AgOTf, instead of the two-electron oxidation, was recently reported.^26^

**Figure 9:**
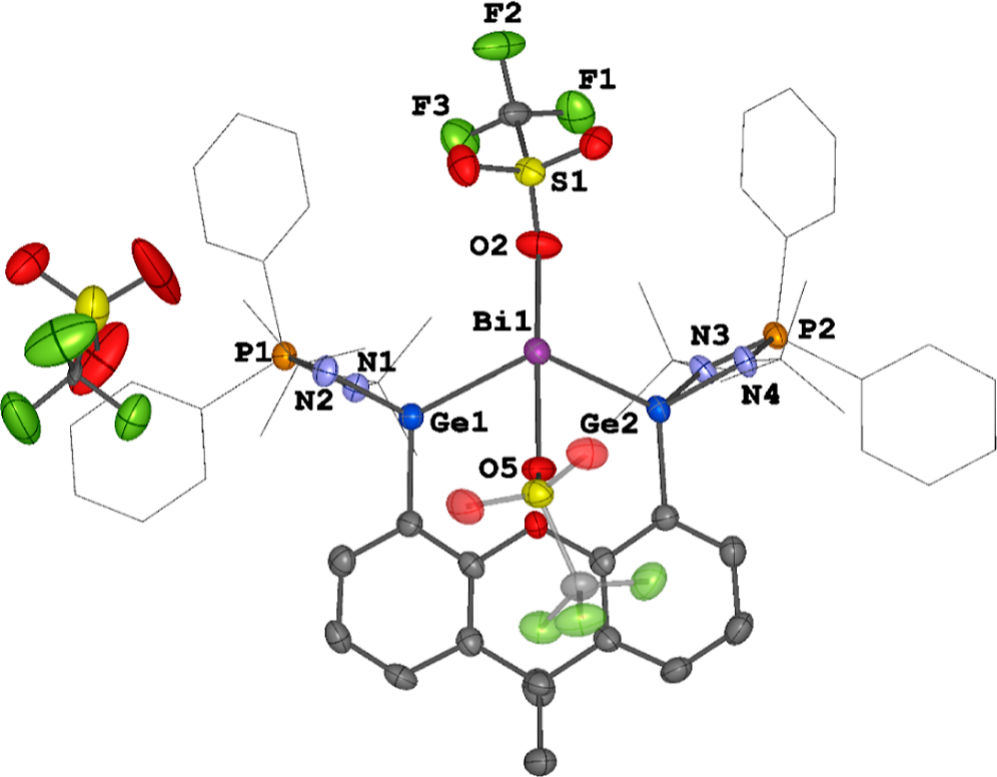
Molecular structure of **7**. Thermal ellipsoids are set at the 50% probability. Hydrogen atoms and solvent molecules are omitted for clarity. Selected bond lengths (Å) and angles (deg): Bi1–Ge1 2.7796(8), Bi1–Ge2 2.7670(8), Bi1–O2 2.410(5), Bi1–O5 2.397(5), Ge1–Bi1–Ge2 107.22(2), O5–Bi1–O2 169.85(18).

The molecular structure of compound **7** was confirmed by XRD analysis (Figure 9). Compound **7** crystallizes in monoclinic space group *P*12_1_/*c*1. Similar to compounds **2** and **3**, the central Bi^III^ site also adopts a seesaw geometry with the two OTf units located in the axial positions (O5–Bi1–O2:169.85(18)°). There is a strong interaction between these two OTf units and the Bi^III^ atom [Bi–O lengths: 2.410(5) and 2.397(5) Å]. In contrast, one OTf anion is noncoordinated to the Bi^III^ center. The Ge–Bi distances in **7** [2.7796(8) and 2.7670(8) Å] are comparable to those of **2** and **3** [2.7497(6) to 2.8035(5) Å].

### Reactivity of Bi^I^ Complex **4**[OTf] toward MeOTf

**Scheme 6:**
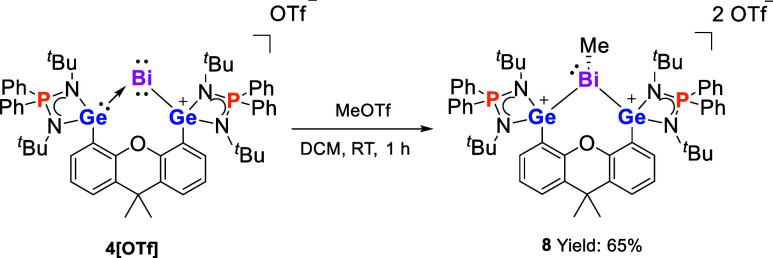
Reaction of **4**[OTf] with MeOTf

To investigate the nucleophilic character of the Bi^I^ complexes, we conducted the reaction of **4**[OTf] with electrophilic MeOTf. As expected, the red color of **4**[OTf] faded upon the addition of MeOTf. Multinuclear NMR analysis confirmed the formation of new species **8** (Scheme 6). After workup, compound **8** was isolated as a pale-yellow powder in 65% yield. Its ^1^H NMR spectrum shows two singlets at δ 1.04 and 1.09 ppm, respectively, for the *tert-*butyl groups, implying an asymmetric structure in solution. In addition, the ^1^H NMR resonance at δ 2.43 ppm corresponds to the methyl group on the bismuth center, which is downfield shifted compared to that of BiMe_3_ (δ 1.11 ppm).^73^ The ^31^P{^1^H} NMR spectrum of **8** displays a singlet at δ 53.4 ppm, downfield shifted compared to that of **4**[OTf] (δ 44.5 ppm). The ^19^F{^1^H} NMR spectrum exhibits a singlet at δ −78.6 ppm, indicating a weak coordinating interaction between the OTf anions and Bi^III^ atom in solution.

**Figure 10:**
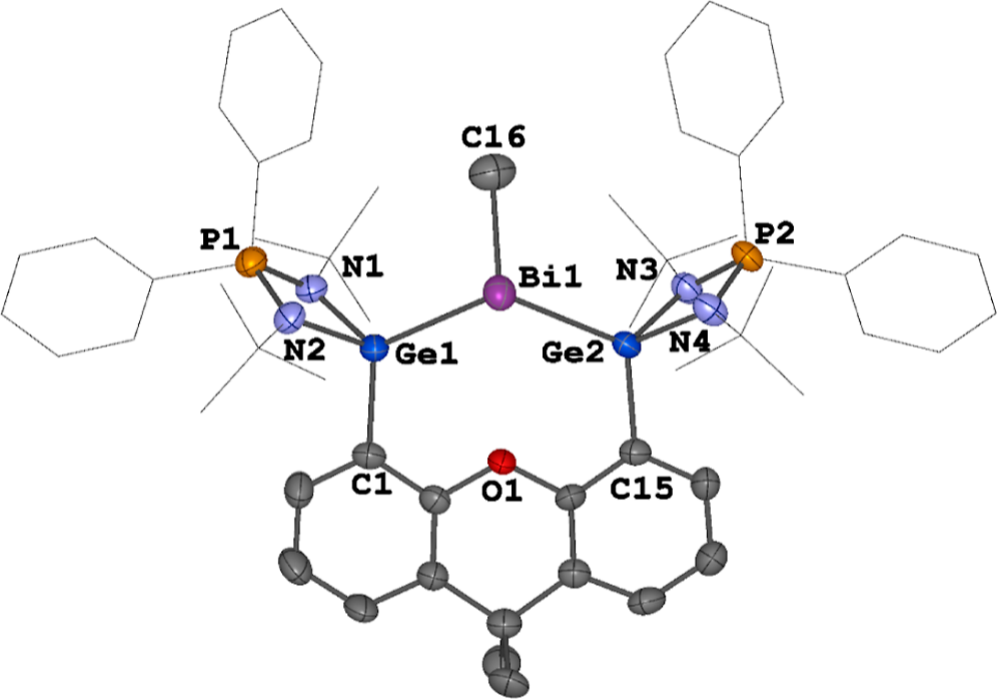
Molecular structure of the dication in **8**. Thermal ellipsoids are set at the 50% probability. Hydrogen atoms, anionic moieties and solvent molecules are omitted for clarity. Selected distances (Å) and angles (deg): Bi1–Ge1 2.7393(7), Bi1–Ge2 2.7426(7), Bi1–C16 2.247(7), Ge1–Bi1–Ge2 108.09(2), C16–Bi1–Ge1 97.6(2), C16–Bi1–Ge2 101.8(2).

Compound **8** was isolated as pale-yellow crystals and its molecular structure was established by XRD analysis (Figure 10). It crystallizes in the orthorhombic space group *P*2_1_2_1_2_1_ as an ion triple with a separated dication and two OTf counteranions. The Bi^III^ center adopts a trigonal pyramidal geometry due to methyl coordination. The Bi–C distance of 2.247(7) Å is shorter than that of [(TBDSi_2_)BiMe][BAr^F^]_2_ (2.300 Å).^23^ The Ge–Bi distances in **8** of 2.7393(7) and 2.7426(7) Å are longer than those in the two-coordinate Bi^I^ complex **4** and radical dication **5** (2.6627(4)–2.7147(3) Å), but shorter than those in the four-coordinate **2**, **3**, and **7** (2.7497(6) to 2.8035(5) Å), respectively.

## Conclusions

In summary, two remarkably resonance-stabilized Bi^I^ complexes, **4**[BAr^F^] and **4**[OTf], supported by an electron-rich chelating bis(iminophosphonamido-germylene) ligand **1,** have been synthesized by reduction of the bis(germylium)Bi^III^I_2_ cation precursors **3**[BAr^F^] and **3**[OTf] with Cp_2_Co, respectively. Featuring two lone pairs at the Bi^I^ atom center, Bi^I^ cation complex **4** can be considered as an isoelectronic analogue of a Pb^0^ complex. Notably, due to the redox noninnocent character of bis(germylene) ligand **1**, the positive charge of Bi^III^ I_2_ cation in **3** and Bi^I^ complex **4** migrates to the germanium atoms in **1** which increases the stability of the Bi^III^ and Bi^I^ centers substantially. This is also corroborated experimentally by the XPS data. The single-electron oxidation of **4**[BAr^F^] with Cp_2_Fe[BAr^F^] furnishes the Bi^II^ radical complex **5**[BAr^F^]_2_ with an unpaired electron located at the Bi^II^ center and both Ge atoms each featuring one positive charge. Oxidation of **4**[OTf] with 2 M equivs AgOTf and addition of MeOTf affords the Bi^III^ complexes **7** and **8**, respectively, demonstrating the nucleophilic character of Bi^I^ cation **4**. We are currently applying these redox-ligand-active Bi^I^ and Bi^II^ complexes in photoredox single-electron transfer catalysis in our laboratory. We believe that the stabilization of Bi^I^ cations and Bi^II^ radical dication complexes by using redox noninnocent ligands is a promising strategy to gain access to related low-valent p-block metal complexes that are otherwise very difficult to tame.

## Supporting Information Available

Experimental details, materials, and methods, including spectroscopic data and computational data (PDF)
