# Supplementary material for: Stabilizing Monoatomic Two-Coordinate Bismuth(I) and Bismuth(II) Using a Redox Noninnocent Bis(germylene) Ligand
Source: J Am Chem Soc. 2024 Feb 26;146(9):6025–36. doi: 10.1021/jacs.3c13016 (PMC10921399; doi:10.1021/jacs.3c13016)
Supplement: Supplementary file 1 — ja3c13016_si_001.pdf [file ja3c13016_si_001.pdf]

# Supporting Information

# Stabilizing Monoatomic Two-Coordinate Bismuth(I) and Bismuth(II) Using a Redox Non-Innocent Bis(germylene) Ligand

Jian Xu,[a] Sudip Pan,[b] Shenglai Yao,[a] Christian Lorent,[c] Christian Teutloff,[d] Zhaoyin Zhang,[e] Jun Fan,[a] Andrew Molino,[f] Konstantin B. Krause,[g] Johannes Schmidt,[h] Robert Bittl,[d] Christian Limberg,[g] Lili Zhao,[e] Gernot Frenking,[e, i] * and Matthias Driess[a] *

[a] *Metalorganic and Inorganic Materials, Department of Chemistry, Technische Universität Berlin, 10623 Berlin, Germany;*
[b] *Institute of Atomic and Molecular Physics, Jilin University, Changchun 130023, China;*
[c] *Physical and Biophysical Chemistry, Department of Chemistry, Technische Universität Berlin, 10623 Berlin, Germany;*
[d] *Fachbereich Physik, Freie Universität Berlin, 14195 Berlin, Germany;*
[e] *Institute of Advanced Synthesis, School of Chemistry and Molecular Engineering, Jiangsu National Synergetic Innovation Center for Advanced Materials, Nanjing Tech University, Nanjing 211816, China;*
[f] *Department of Chemistry and Physics, La Trobe Institute for Molecular Science, La Trobe University, Melbourne, 3086 Victoria, Australia;*
[g] *Institut für Chemie, Humboldt-Universität zu Berlin, 12489 Berlin, Germany;*
[h] *Functional Materials, Department of Chemistry, Technische Universität Berlin, 10623 Berlin, Germany;*
[i] *Philipps-Universität Marburg, Fachbereich Chemie, 35032 Marburg, Germany.*

## Contents

A. Experimental Procedures ........ 1
A1. General Considerations ........ 1
A2. Single-Crystal X-ray Structure Determination ........ 1
A3. Cyclic Voltammetry Measurement ........ 2
A4. EPR Spectroscopy ........ 2
A5. X-ray photoelectron spectroscopy (XPS) ........ 2
B. Synthesis and Characterization ........ 3
C. X-ray Crystallographic Data ........ 39
D. Magnetic susceptibility measurements ........ 59
E. Electron Paramagnetic Resonance (EPR) ........ 63
F. XPS spectra ........ 66
G. Theoretical Calculations ........ 67
References ........ 115

# A. Experimental Procedures

## A1. General Considerations

All experiments were carried out under dry oxygen-free nitrogen using standard Schlenk techniques or MBraun glove box fitted with a gas purification and recirculation unit. Solvents were dried by standard methods and freshly distilled prior to use. Iminophosphonamide and $Cp_2FeBAr^F$ were synthesized according to reported procedures.[1,2] The solution NMR spectra were recorded on Bruker Spectrometers AV 200, 400 and 500 with residual solvent signals as internal reference ($^1$H NMR: Benzene-$d_6$, 7.16 ppm, THF-$d_8$, 3.58 and 1.72 ppm, DCM-$d_2$, 5.32 ppm; $^{13}$C{$^1$H} NMR: Benzene-$d_6$, 128.06 ppm, THF-$d_8$: 67.21 and 25.31 ppm, DCM-d$_2$, 53.84 ppm) and external standards (85% $H_3PO_4$ for $^{31}$P{$^1$H} NMR, $^{11}$B{$^1$H} NMR and $^{19}$F{$^1$H} NMR: $BF_3.Et_2O$). The following abbreviations were used to describe peak patterns when appropriate: br = broad, s = singlet, d = doublet, t = triplet, dd = doublet of doublets, m = multiplet. Elemental analyses were performed by the analytical labor service in the Institute of Chemistry, Humboldt University of Berlin, Germany. High-resolution ESI-MS were measured on a Thermo Scientific LTQ orbitrap XL. UV/Vis spectra were recorded on an Analytik Jena Specord S600 diode array spectrometer.

## A2. Single-Crystal X-ray Structure Determination

Crystals were each mounted on a glass capillary in perfluorinated oil and measured in a cold $N_2$ flow. The data of all compounds were collected on an Oxford Diffraction SuperNova, Single source at offset, Atlas at 110 K or 150 K (Cu-Kα radiation, $\lambda$ = 1.54184 Å). The structures were solved by direct methods and refined on $F^2$ with the SHELX-2014[3] and OLEX2[4] software package. For the crystal of **2**, the residual density of I2 due to near heavy atom Bi1. Residual electron density was observed close to the center of the bismuth, which may be due to anharmonic displacement of the heavy metal atoms. For the crystal of compounds **2**, **3**[OTf], **4**[BAr$^F$], **4**[OTf], **5**[BAr$^F$]$_2$ and **7**, strongly disordered solvent molecules $CH_3CN$ (**2**), $C_6H_5F$ (**3**[OTf] and **5**), DCM (**4**[BAr$^F$] and **4**[OTf]) and $C_7H_8$ (**7**) were treated using Solvent Masking in Olex2. In the molecular structure of compound **2**, the I1 and I3 atoms are disordered over two positions with an approximate occupancy ratio of 0.95: 0.045 (I1: I1a) and 0.97: 0.03 (I3: I3a). Similarly, in the molecular structure of compound **3**[OTf], the Bi1 atom is disordered over two positions with an approximate occupancy ratio of 0.96:0.04 (Bi1: Bi1a).

**CCDC**: 2290234 (Iminophosphonamido-chlorogermylene: **(P)GeCl**), 2290235 (**1**), 2290236 (**2**), 2290237 (**3**[BAr$^F$]), 2290238 (**3**[OTf]), 2290239 (**4**[BAr$^F$]), 2290240 (**4**[OTf]), 2290241 (**5**[BAr$^F$]$_2$), 2290242 (**7**) and 2290243 (**8**) contain the supplementary crystallographic data for this paper. These data can be obtained free of charge from The Cambridge Crystallographic Data Centre via www.ccdc.cam.ac.uk/structures/

## A3. Cyclic Voltammetry Measurement

Cyclic voltammetry (CV) measurements were performed in a standard three-electrode electrochemical cell having Pt-wire used as an auxiliary electrode, glassy carbon (3 mm diameter) as working electrode and Pt-wire as a pseudo reference electrode at 295 K using a Biologic SP-150 potentiostat. All cyclic voltammograms were referenced against the $Cp_2Fe/Cp_2Fe^+$ redox couple ($Fc/Fc^+$), which was used as an internal standard. 0.1 M tetrabutyl ammonium hexaflurophosphate ($TBAPF_6$) in THF was used as an electrolyte. The iR-drop was determined and compensated by using the impedance measurement technique implemented in the EC-Lab Software V11.52.

## A4. EPR Spectroscopy

Quartz EPR tubes for X-band (3.0/4.0 mm i.d./o.d., QSIL, Germany), Q-band (2.0/2.9 mm i.d./o.d., QSIL, Germany) and for W-band (0.70 /0.87 mm i.d./o.d., VitroCom Inc, USA) were filled with the sample dissolved in diethylether in a glove box and immediately frozen in liquid nitrogen, then flame-sealed under a helium atmosphere at 400 mbar. W-band samples were sealed with Critoseal prior to freezing to prevent fast evaporation of the solvent.

EPR experiments at cryogenic temperature (10 K) were performed using an Elexsys E580 X-band (9 GHz) and Q-band (34 GHz) spectrometer using a home-built probehead, and an Elexsys E680 W-band (94 GHz) spectrometer equipped with a Teraflex EN600-1021H probe head (both Bruker). Temperature control was achieved by an ESR900 helium flow cryostat and an ITC4 temperature controller (both Oxford Instruments, UK) at X- and Q-band and a CF935 helium bath cryostat and an ITC503 temperature controller (both Oxford Instruments, UK) at W-Band. Simulation of the EPR spectrum at W-Band was done with the MATLAB toolbox EasySpin version 6.0.0-dev.53.

## A5. X-ray photoelectron spectroscopy (XPS)

X-ray photoelectron spectroscopy (XPS) measurements were carried out using a Thermo Scientific K-Alpha+ X-ray Photoelectron Spectrometer with Al Kα radiation. All samples were analyzed using a microfocused, monochromated Al K α X-ray source (1486.68 eV; 400 µm spot size). The analyser had a pass energy of 200 eV (survey), and 50 eV (high resolution spectra), respectively. To prevent any localized charge buildup during analysis the K-Alpha+ charge compensation system was employed at all measurements. The samples were mounted on conductive carbon tape the resulting spectra analyzed using the Avantage software from Thermo Scientific.

## B. Synthesis and Characterization

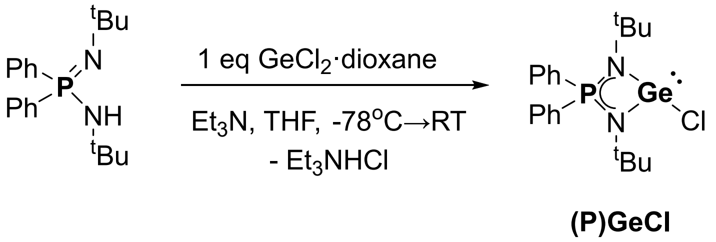

**Synthesis of iminophosphonamido-chlorogermylene( (P)GeCl).** To a mixture of iminophosphonamide (5.01 g, 15.3 mmol) and $NEt_3$ (3.0 mL, 21.6 mmol) in THF (70 mL), $Ge_2Cl_2$·dioxane (3.5 g, 15.3 mmol) in 50 mL THF was added at -78 ˚C and then the mixture was gradually warmed up to room temperature. After stirring overnight, the reaction mixture was filtered and all volatiles of the filtrate were removed under vacuum. Recrystallization from THF gave the corresponding iminophosphonamido-chlorogermylene **(P)GeCl** (4.3 g, 65%) as colorless crystals. Colorless block crystals suitable for X-ray diffraction analysis were obtained from a concentrated THF solution at room temperature.

M.p. 159-164 ºC.

$^{1}$H NMR (200 MHz, Benzene-d$_6$) $\delta$/ppm = 8.13 – 7.95 (m, 4H, Ar-*H*), 7.15 – 7.03 (m, 6H, Ar-*H*), 1.04 (s, 18H, C(C*H*$_3$)$_3$).

$^{13}$C{$^{1}$H} NMR (50 MHz, Benzene-*d*$_6$) $\delta$/ppm = 133.59 (d, *J* = 11.5 Hz, Ar-*C*), 132.65 (d, *J* = 3.0 Hz, Ar-*C*), 132.35 (d, *J* = 90.2 Hz, Ar-*C*), 128.86 (d, *J* = 12.0 Hz, Ar-*C*), 53.04 (*C*(CH$_3$)$_3$), 32.39 (d, *J* = 6.5 Hz, C(*C*H$_3$)$_3$).

$^{31}$P{$^{1}$H} NMR (81 MHz, Benzene-*d*$_6$) $\delta$/ppm = 42.25.

HR-MS (ESI): (m/z) calcd for $[M-Cl]^+$ $(C_{20}H_{28}GeN_2P)^+$: 401.1196; found: 401.1189.

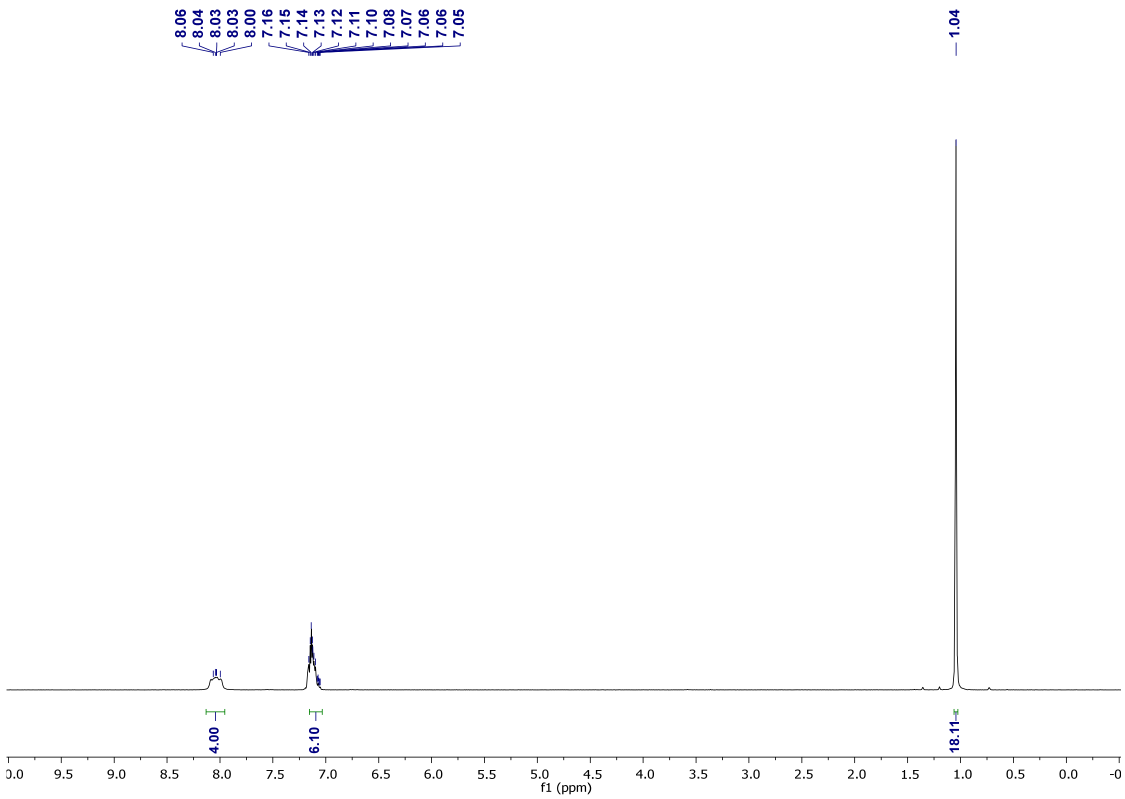


**Figure S1.** $^{1}$H NMR spectrum of iminophosphonamido-chlorogermylene **(P)GeCl** in Benzene-*d*$_6$.

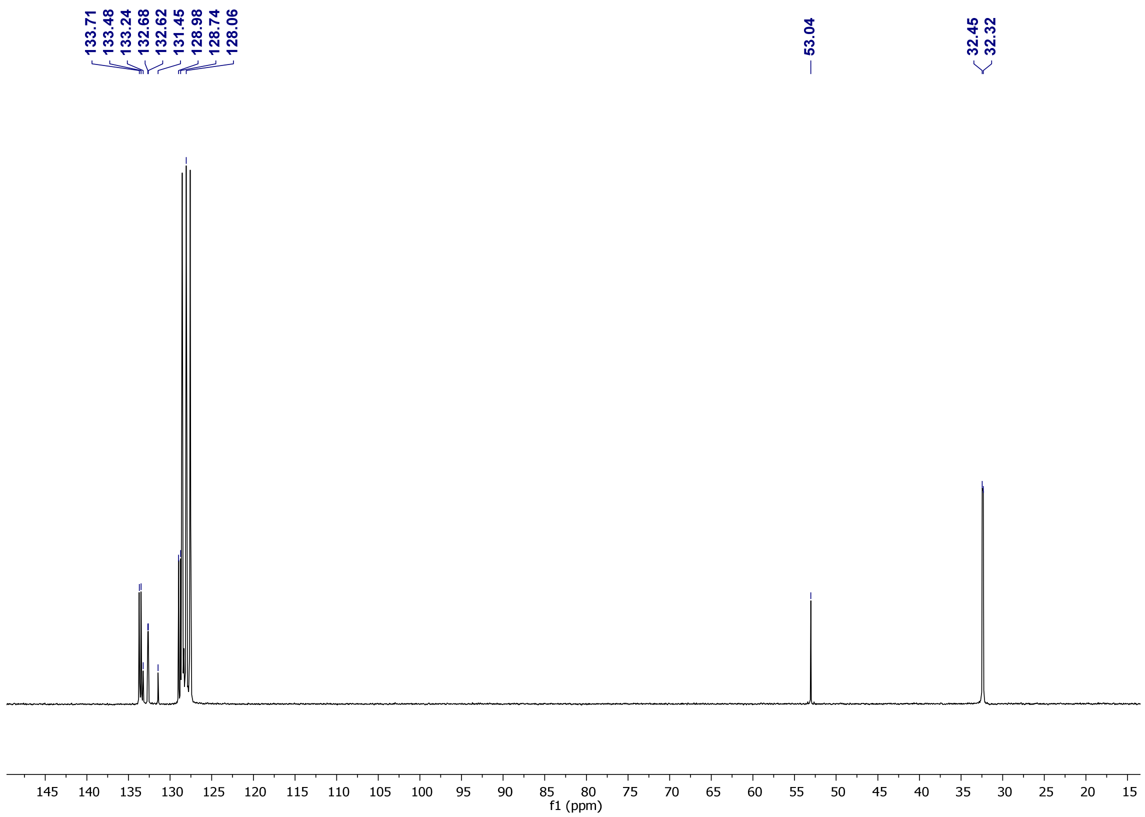


**Figure S2.** $^{13}$C{$^{1}$H} NMR spectrum of iminophosphonamido-chlorogermylene **(P)GeCl)** in Benzene-*d*$_6$.

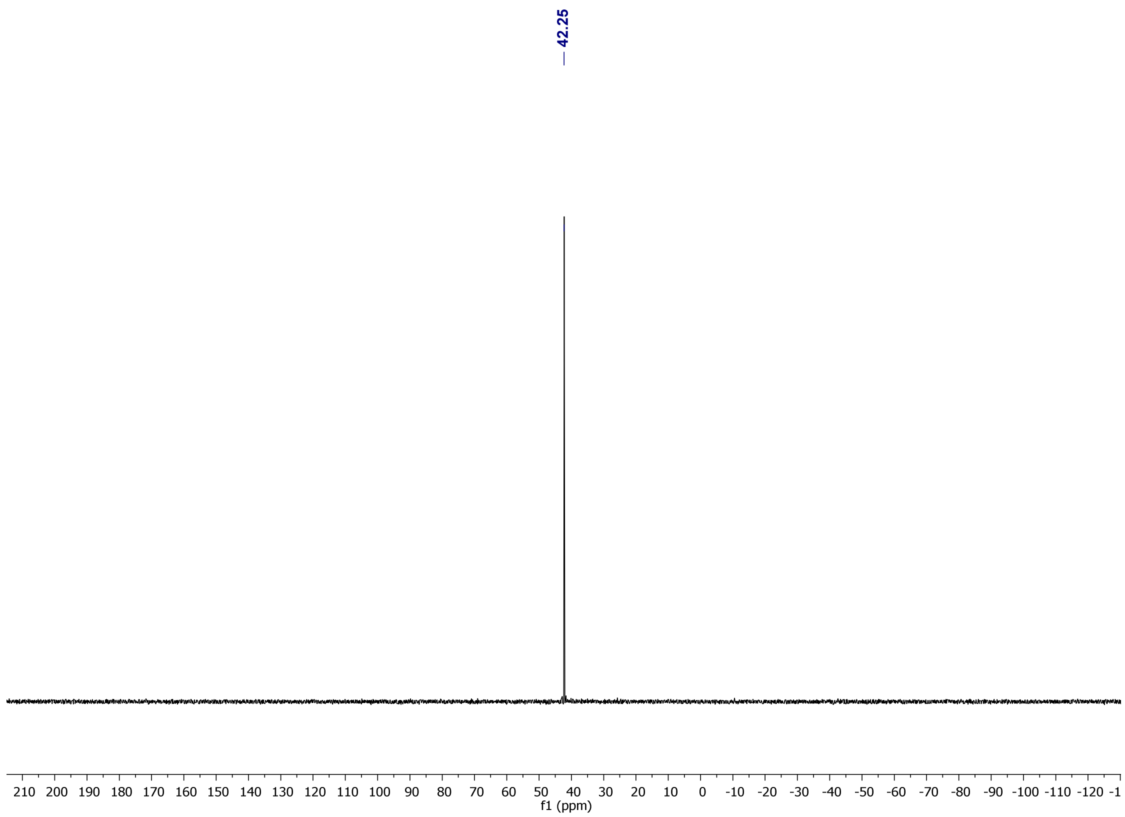


**Figure S3.** $^{31}P\{^1H\}$ NMR spectrum of iminophosphonamido-chlorogermylene **(P)GeCl** in Benzene-$d_6$.

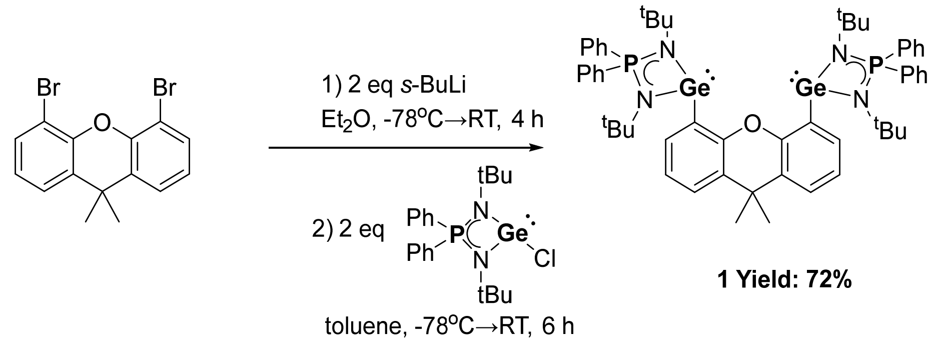


**Synthesis of compound 1.** 1.3 M *s*-BuLi (15.5 mL, 20.15 mmol) was added into a solution of 4,5-dibromo-9,9-dimethylxanthene (3.68 g, 10 mmol) in 60 ml $Et_2O$ at -78 °C. After stirring for 0.5 h in cooling bath, the mixture was allowed to warm up to room temperature and stirred for 4 h. Then the reaction mixture was cooled to -78 °C again, a solution of iminophosphonamido-chlorogermylene **(P)GeCl** (8.7 g, 20 mmol) in 80 mL toluene was added dropwise into the mixture. After stirring 0.5 h at -78 °C, the cooling bath was removed and the reaction mixture was stirred at room temperature for 4 h. The resulting orange suspension was filtered via cannula, then the filtrate was concentrated to 20 mL and crystallized overnight at -30 °C affording **1** as yellow powder. (7.56 g, 72% isolated yields). Yellow block crystals suitable for X-ray diffraction analysis were obtained from a concentrated $Et_2O$/toluene solution at -20 °C.

M.p. 236-239 °C.

$^{1}H$ NMR (200 MHz, THF-$d_8$) $\delta$/ppm = 8.21 – 8.07 (m, 8H, Ar-*H*), 8.03 – 7.96 (m, 2H, Ar-*H*), 7.58 (m, *J* = 5.8, 12H, Ar-*H*), 7.26 (dd, *J* = 7.6, 1.6 Hz, 2H, Ar-*H*), 6.95 (t, *J* = 7.3 Hz, 2H, Ar-*H*), 1.58 (s, 6H, $C(CH_3)_2$), 0.99 (s, 36H, $C(CH_3)_3$).

$^{13}C\{^{1}H\}$ NMR (101 MHz, THF-$d_8$) $\delta$/ppm = 157.87 (s, Ar-*C*), 152.53 (s, Ar-*C*), 136.64 (d, *J* = 85.2 Hz, Ar-*C*), 135.49 (d, *J* = 90.2 Hz, Ar-*C*), 134.53 (d, *J* = 11.2 Hz, Ar-*C*), 134.06 (d, *J* = 11.4 Hz, Ar-*C*), 133.27 (s, Ar-*C*), 132.19 (dd, *J* = 7.8, 2.8 Hz, Ar-*C*), 129.67 (s, Ar-*C*), 128.74 (dd, *J* = 23.3, 11.6 Hz, Ar-*C*), 124.56 (s, Ar-*C*), 121.98 (s, Ar-*C*), 52.57 (s, $C(CH_3)_3$), 34.63 (s, $C(CH_3)_2$, 32.86 (d, *J* = 7.0 Hz, $C(CH_3)_3$), 31.92 ($C(CH_3)_2$).

$^{31}P\{^{1}H\}$ NMR (81 MHz, THF-$d_8$) $\delta$/ppm = 26.63.

HR-MS (ESI): (m/z) calcd for $[M+H]^+$ $(C_{55}H_{68}Ge_2ON_4P_2+H)^+$: 1009.3374; found: 1009.3345.

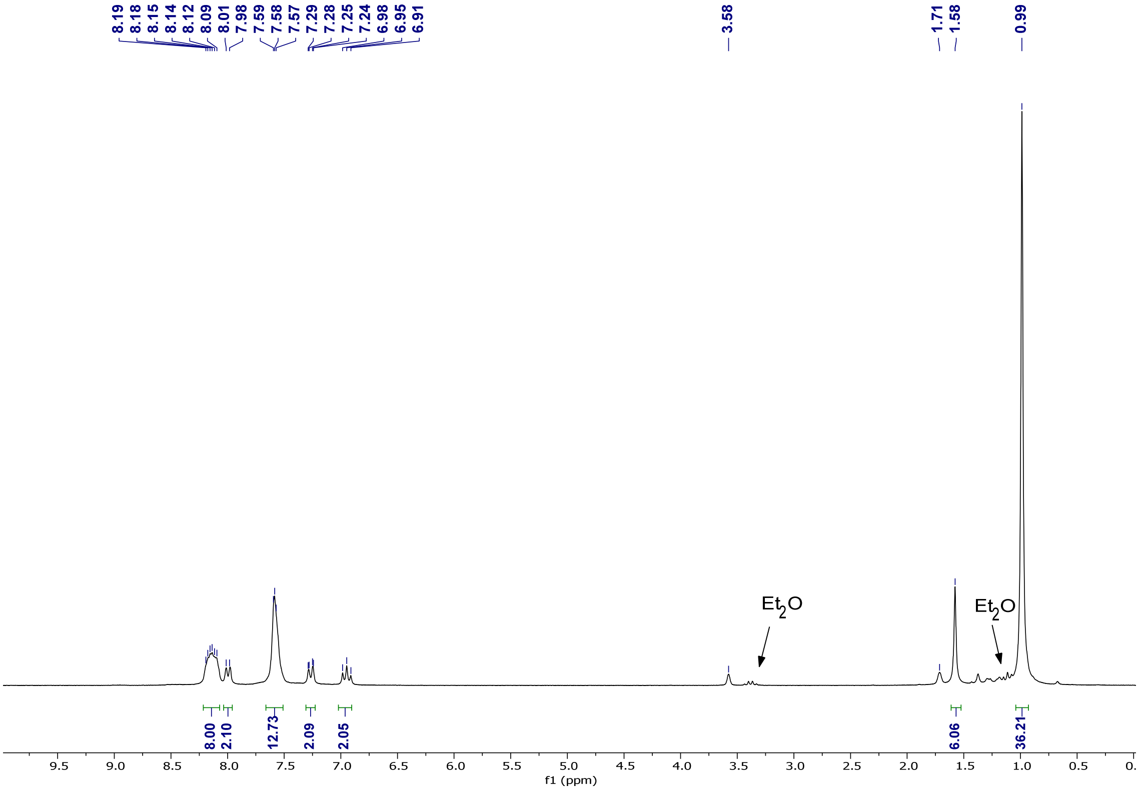

**Figure S4.** $^{1}H$ NMR spectrum of **1** in THF-$d_8$.

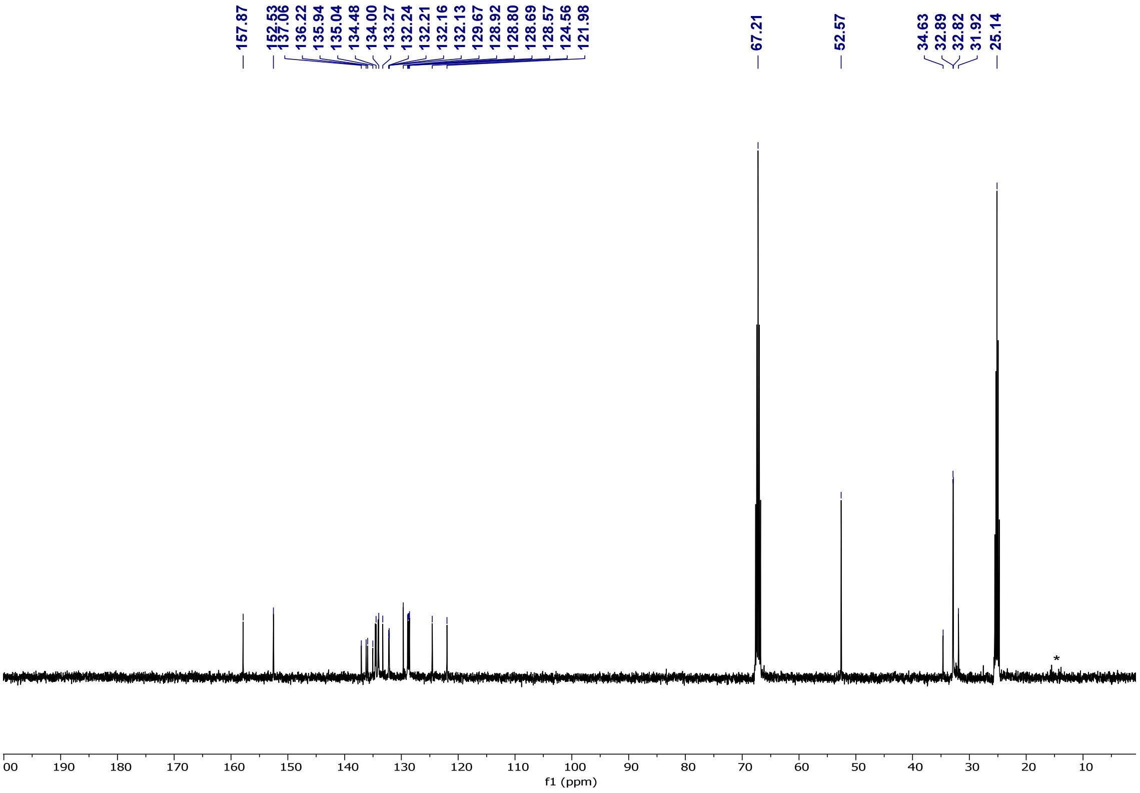

**Figure S5.** $^{13}C\{^{1}H\}$ NMR spectrum of **1** in THF-$d_8$.*Unidentified impurities.

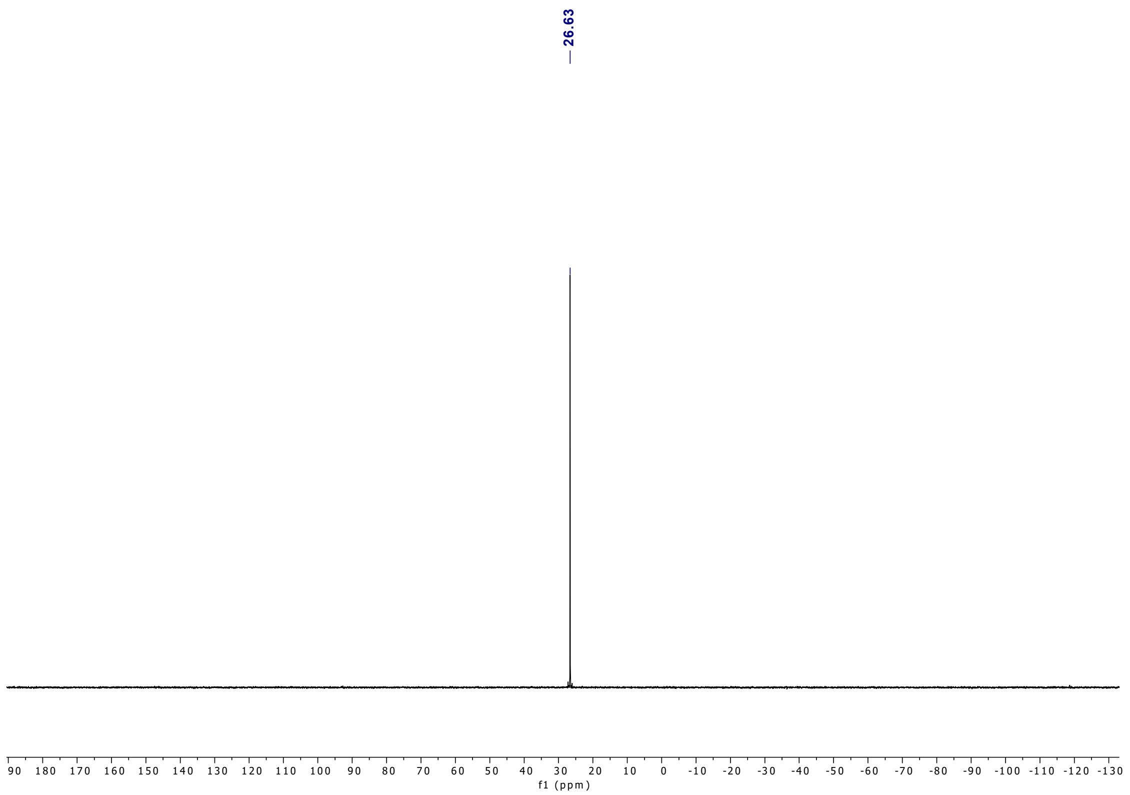


**Figure S6.** $^{31}P\{^1H\}$ NMR spectrum of **1** in THF-$d_8$.

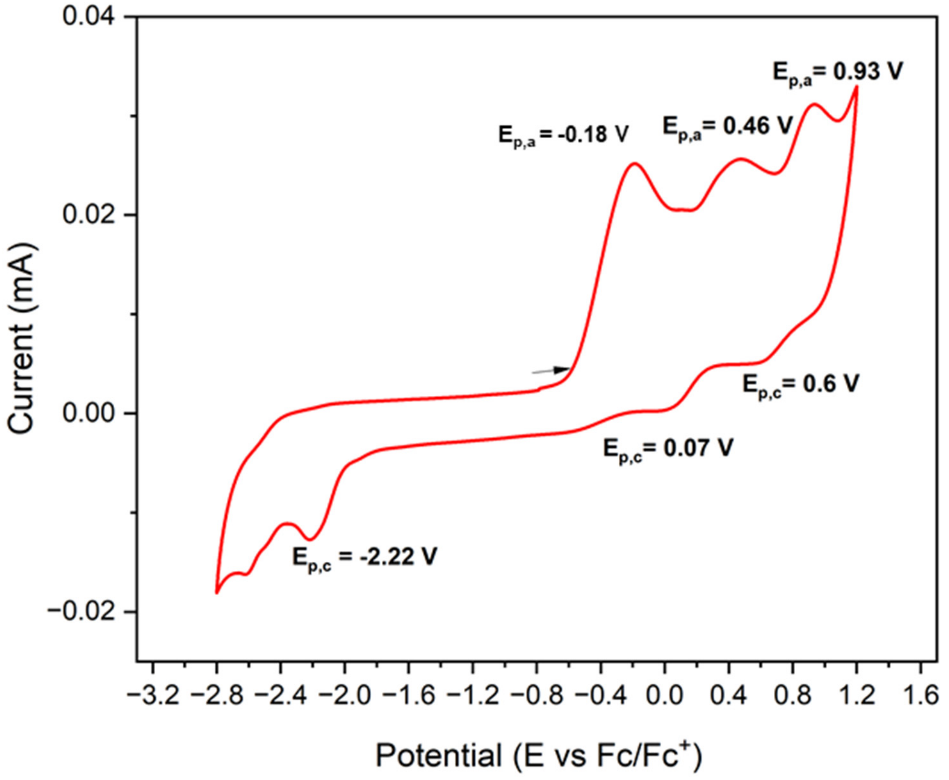


**Figure S7.** CV of **1** at a scan anodically rate of v = 100 mVs$^{-1}$ (1 mM in THF/ 0.1 M $TBAPF_6$). Initial potential E= -0.8 V vs. Fc/Fc$^+$.

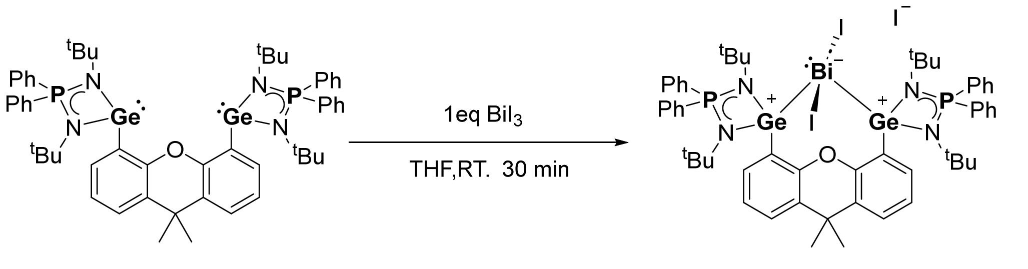


1 2

**Synthesis of compound 2.** To the mixture of **1** (1.05 g, 1 mmol) and $BiI_3$ (589 mg, 1 mmol) in a 100 mL Schlenk flask was added 40 mL THF at room temperature under stirring. After stirring 30 min, a brown precipitate formed slowly. The brown precipitate was separated by filtration and dried under vacuum affording compound **2** as a brown solid (1.36 g, 85% isolated yields). Orange block crystals suitable for X-ray diffraction analysis were obtained from a concentrated $CH_3CN$ solution at -30 ºC.

M.p. 216-218 ºC (decomp.).

$^1$H NMR (200 MHz, DCM-$d_2$) $\delta$/ppm = 8.68 (dd, $J$ = 13.4, 7.6 Hz, 4H, Ar-$H$), 8.23 (m, 8H, Ar-$H$), 7.89 – 7.74 (m, 12H, Ar-$H$), 7.59 (m, 2H, Ar-$H$), 1.63 (s, 6H, C(C$H_3$)$_2$), 1.20 (s, 36H, C(C$H_3$)$_3$).

$^{13}$C{$^1$H} NMR (50 MHz, DCM-$d_2$) $\delta$/ppm = 154.74 (s, Ar-$C$), 136.11 (d, $J$ = 12.8 Hz, Ar-$C$), 135.46 (dd, $J$ = 7.3, 3.0 Hz, Ar-$C$), 134.63 (d, $J$ = 11.9 Hz, Ar-$C$), 132.88 (s, Ar-$C$), 131.92 (s, Ar-$C$), 130.32 (s, Ar-$C$), 130.02 (d, $J$ = 12.9 Hz, Ar-$C$), 129.67 (d, $J$ = 13.3 Hz, Ar-$C$), 129.07 (s, Ar-$C$), 128.86 (d, $J$ = 32.9 Hz, Ar-$C$), 126.92 (d, $J$ = 34.4 Hz, Ar-$C$), 125.73 (s, Ar-$C$), 55.79 (s, $C$($CH_3$)$_3$), 36.79 (s, $C$($CH_3$)$_2$), 33.31 (d, $J$ = 5.7 Hz, C($C$H$_3$)$_3$), 30.03 (br, C($C$H$_3$)$_2$).

$^{31}$P{$^1$H} NMR (81 MHz, DCM-$d_2$) $\delta$/ppm = 54.60.

HR-MS (ESI): (m/z) calcd for $[M-I]^+$ $(C_{55}H_{68}Ge_2N_4P_2BiI_2)^+$: 1471.1189; found: 1471.1166.

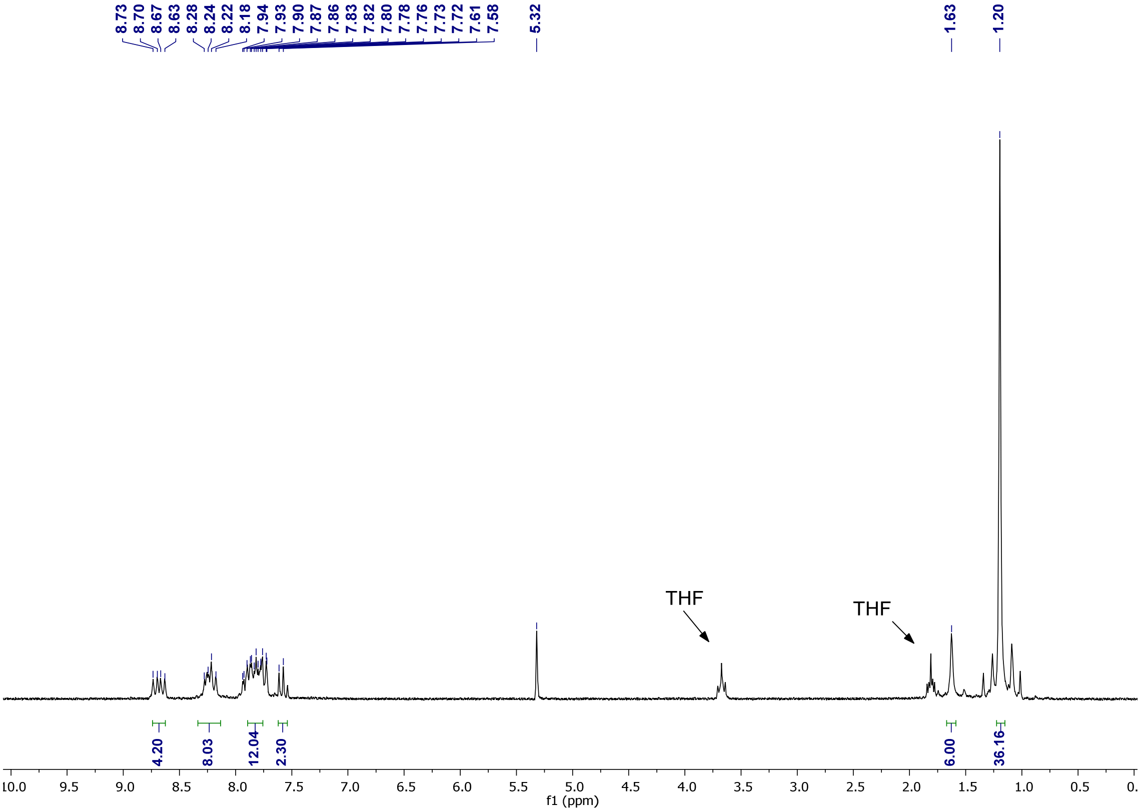


**Figure S8.** $^{1}H$ NMR spectrum of **2** in DCM-$d_2$.

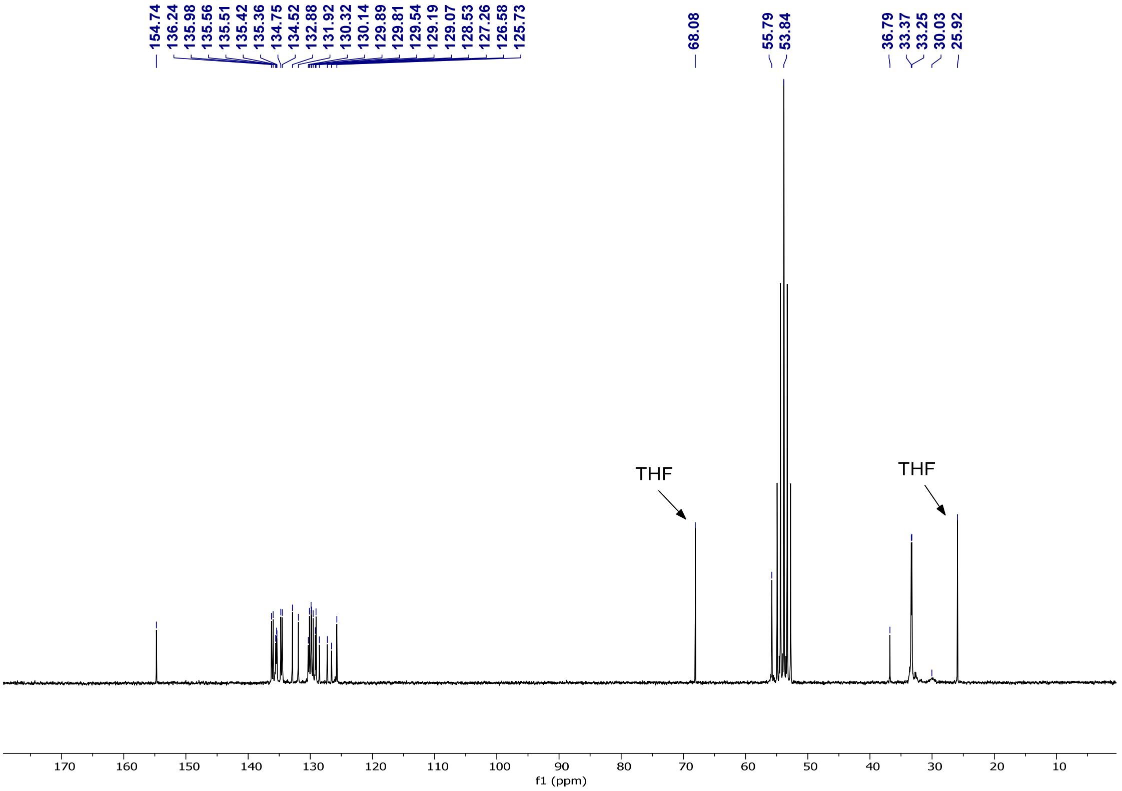


**Figure S9.** $^{13}C\{^{1}H\}$ NMR spectrum of **2** in DCM-$d_2$.

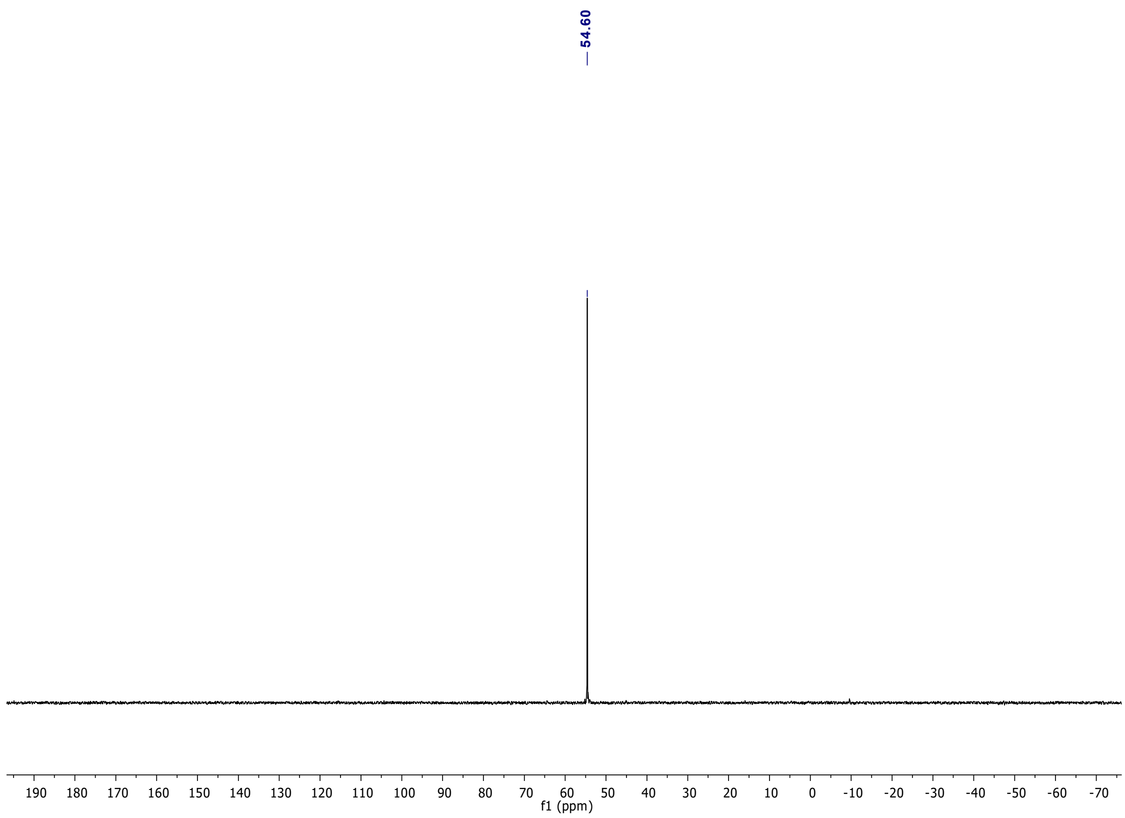


**Figure S10.** $^{31}P\{^1H\}$ spectrum of **2** in DCM-$d_2$.

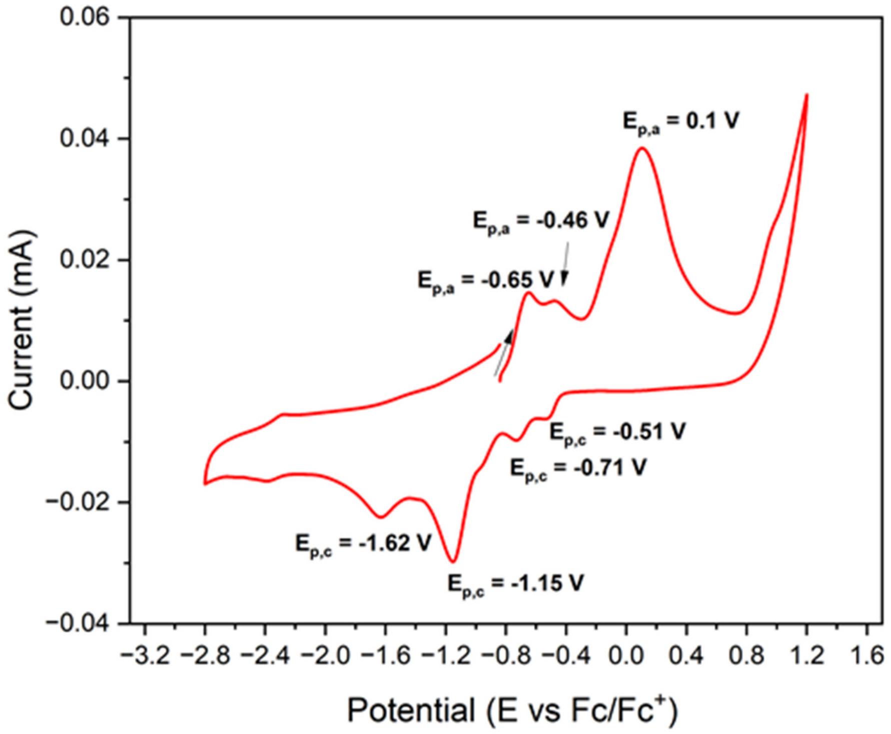


**Figure S11.** CV of **2** at a scan anodically rate of v = 100 $mVs^{-1}$ (1 mM in THF/ 0.1 M $TBAPF_6$). Initial potential E= -0.8 V vs. $Fc/Fc^+$.

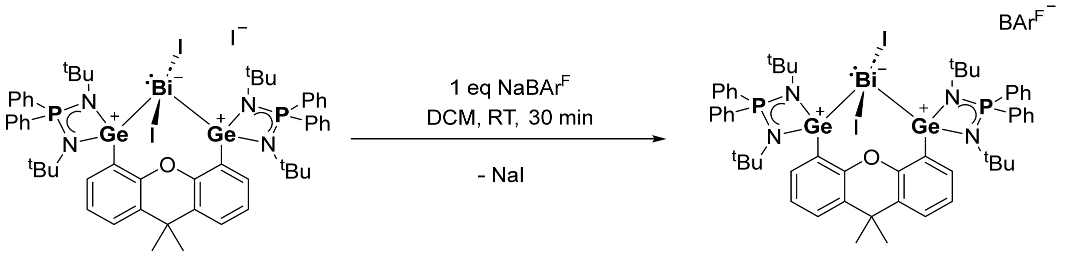


**2** **3**[BArF]

**Synthesis of compound 3[BAr$^F$].** To a mixture of compound **2** (1.6 g, 1 mmol) and NaBAr$^F$ (886 mg, 1 mmol) in a 100 mL Schlenk flask was added 60 mL DCM at room temperature under stirring. The color of the mixture changed to orange immediately. After stirring 30 min, the mixture was filtered and all volatiles were removed and dried under vacuum to afford compound **3**[BAr$^F$] as an orange powder (2.17 g, 92% isolated yield). Orange block crystals suitable for X-ray diffraction analysis were obtained from a DCM/hexane solution at -30 °C.

M.p. 226-230 °C (decomp.).

$^1$H NMR (400 MHz, DCM-$d_2$) $\delta$/ppm = 8.75 – 8.66 (m, 4H, Ar-*H*), 8.28 – 8.24 (m, 4H, Ar-*H*), 8.24 – 8.21 (m, 2H, Ar-*H*), 7.92 – 7.83 (m, 4H, Ar-*H*), 7.77 (m, 18H, Ar-*H*), 7.56 (s, 6H, Ar-*H*), 1.64 (s, 6H, C(C$H_3$)$_2$), 1.23 (s, 36H, C(C$H_3$)$_3$).

$^{13}$C{$^1$H} NMR (101 MHz, DCM-$d_2$) $\delta$/ppm = 162.16 (dd, $J_{C-B}$ = 99.6, 49.8 Hz, BAr$^F$-Ar*C*), 154.83 (s, Ar-*C*), 136.16 (d, $J$ = 12.8 Hz, Ar-*C*), 135.42 (s, Ar-*C*), 134.67 (d, $J$ = 12.0 Hz, Ar-*C*), 132.98 (s, Ar-*C*), 131.94(s, Ar-*C*), 130.36 (s, Ar-*C*), 129.91 (d, $J$ = 12.9 Hz, Ar-*C*), 129.69 (d, $J$ = 13.4 Hz, Ar-*C*), 129.44 (m, BAr$^F$-Ar*C*), 129.21 – 129.03 (m, BAr$^F$-Ar*C*), 129.12(s, Ar-*C*), 128.36 (d, $J$ = 96.9 Hz, Ar-*C*),127.59 (d, $J$ = 98.3 Hz, Ar-*C*), 125.69(s, Ar-*C*), 124.98 (q, , $J_{C-F}$ = 272.4 Hz, BAr$^F$-$CF_3$), 118.12 – 117.67 (m, BAr$^F$-Ar*C*), 55.86 (s, (*C*($CH_3$)$_3$), 36.82 (s, *C*($CH_3$)$_2$), 33.32 (d, $J$ = 5.7 Hz, C(*C*$H_3$)$_3$). 30.41 (br, (C(*C*$H_3$)$_2$)).

$^{31}$P{$^1$H} NMR (81 MHz, DCM-$d_2$) $\delta$/ppm = 54.63.

$^{11}$B{$^1$H} NMR (64 MHz, DCM-$d_2$) $\delta$/ppm = -6.60.

$^{19}$F{$^1$H} NMR (188 MHz, DCM-$d_2$) $\delta$/ppm = -62.81.

HR-MS (ESI): (m/z) calcd for [M-BAr$^F$]$^+$ ($C_{55}H_{68}Ge_2N_4P_2BiI_2$)$^+$: 1471.1189; found: 1471.1187. [BAr$^F$]$^-$ ($C_{32}H_{112}BF_{24}$)$^-$: 863.0643 found 863.0632.

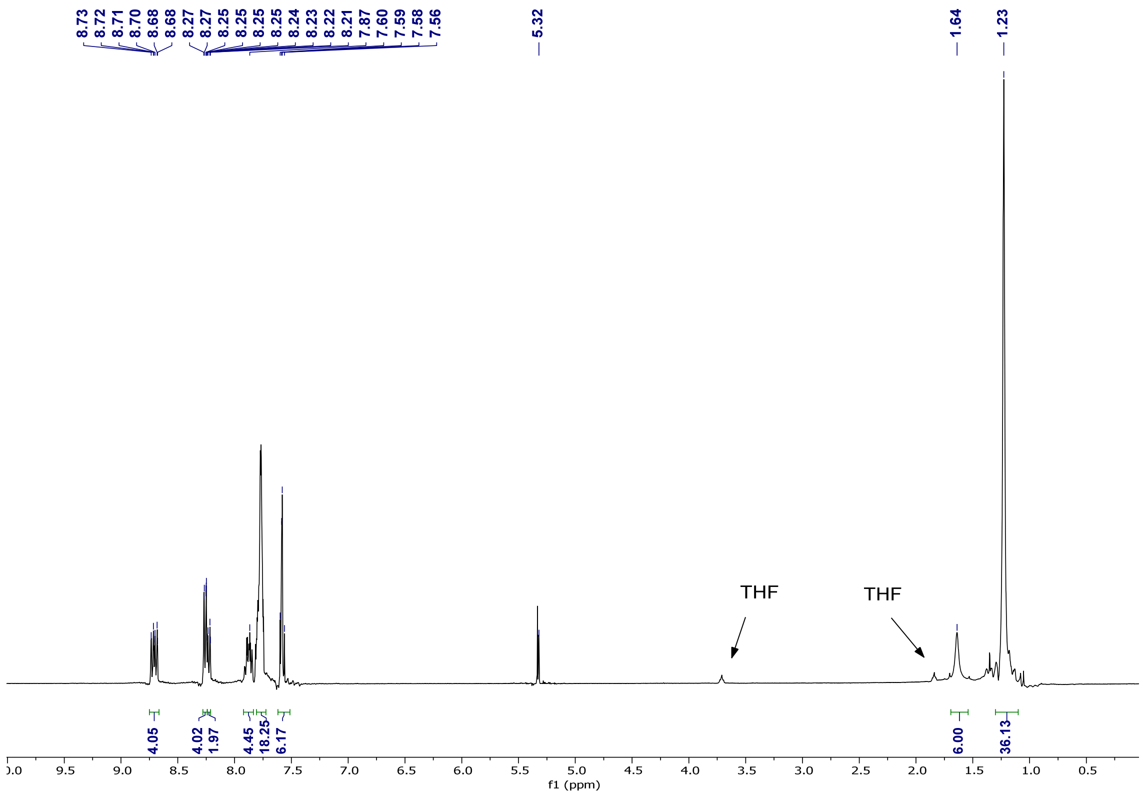


**Figure S12.** $^{1}H$ NMR spectrum of **3**[BAr$^{F}$] in DCM-$d_2$.

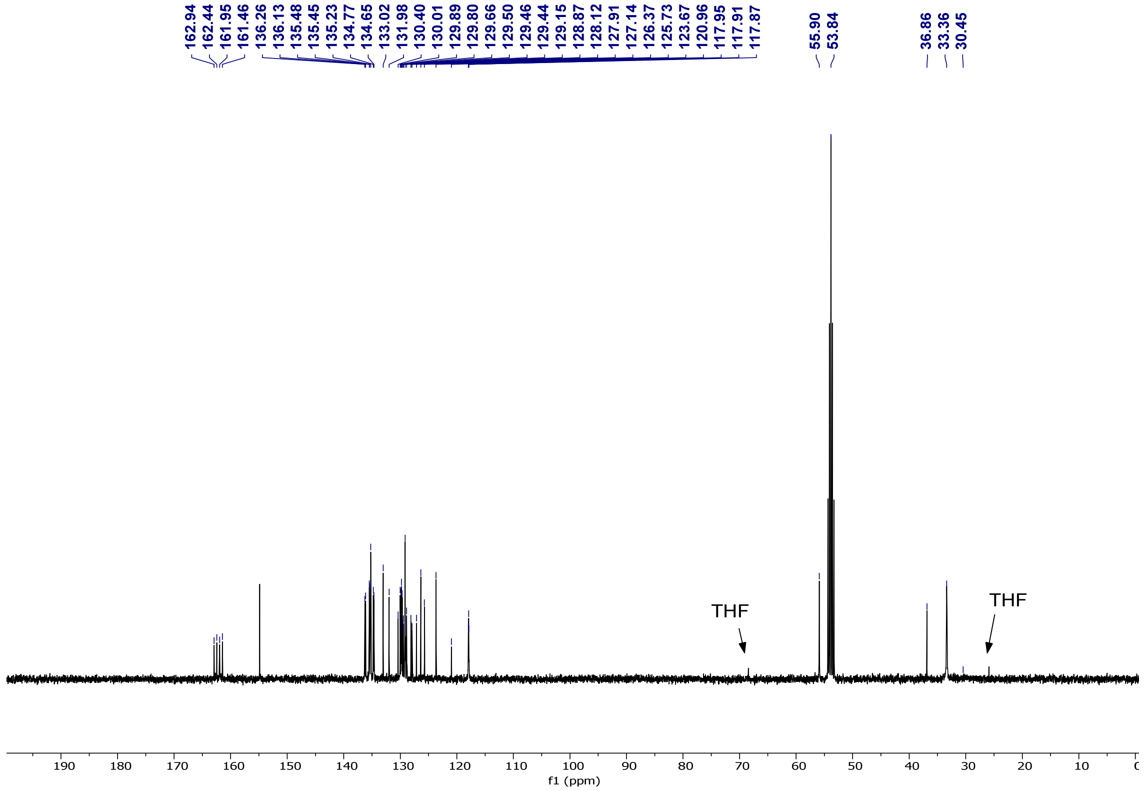


**Figure S13.** $^{13}C\{^{1}H\}$ NMR spectrum of **3**[BAr$^{F}$] in DCM-$d_2$.

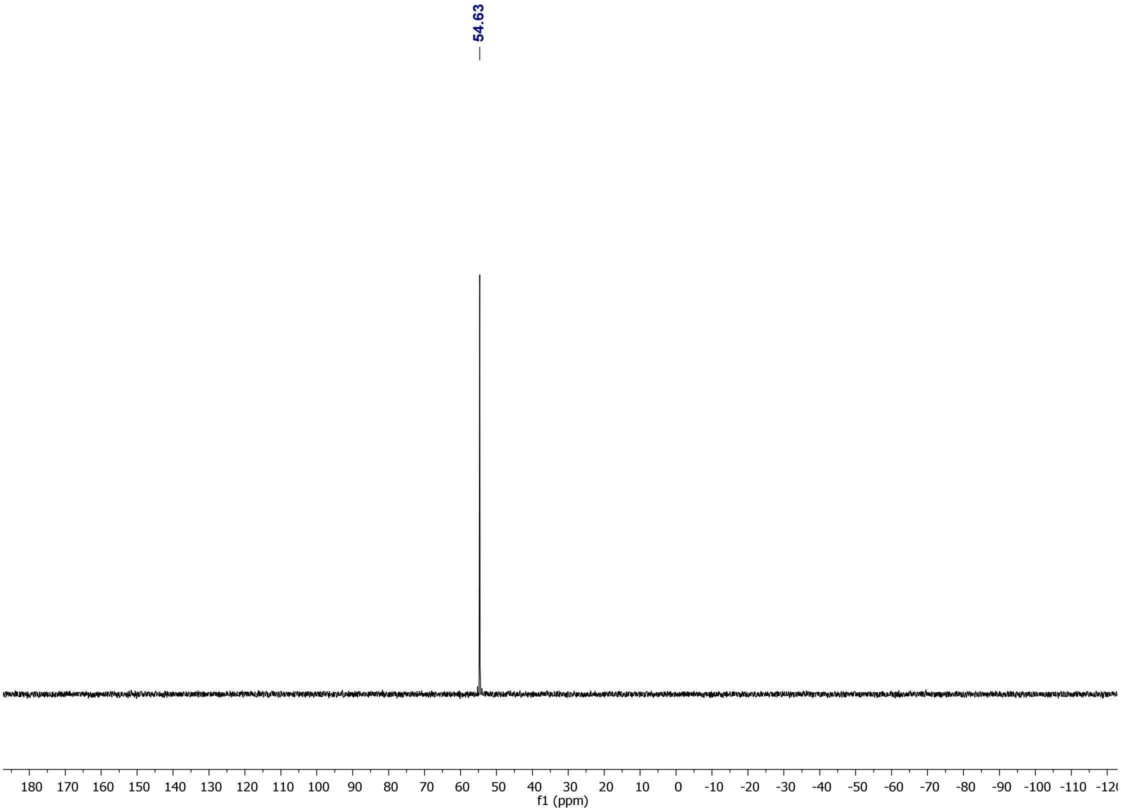


**Figure S14.** $^{31}P\{^{1}H\}$ NMR spectrum of **3**[BAr$^{F}$] in DCM-$d_2$.

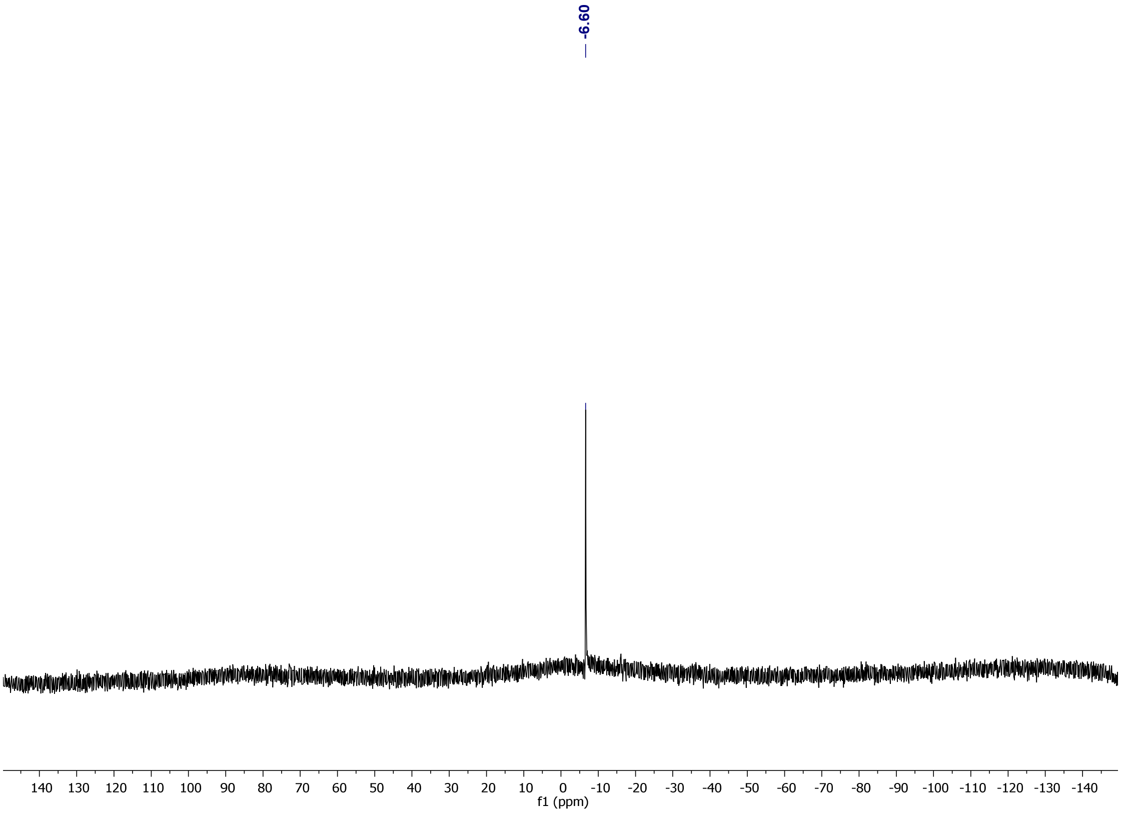


**Figure S15.** $^{11}B\{^{1}H\}$ NMR spectrum of **3**[BAr$^{F}$] in DCM-$d_2$.

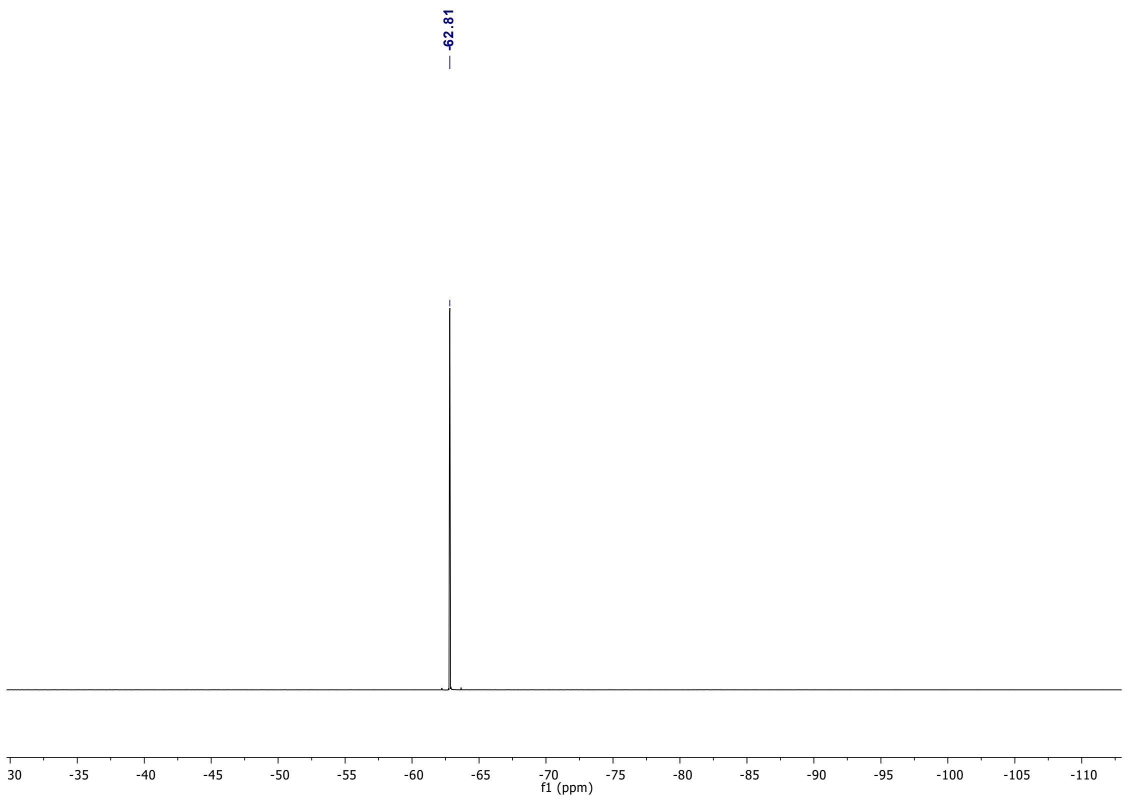


**Figure S16.** $^{19}F\{^1H\}$ spectrum of **3**[BAr$^F$] in DCM-$d_2$.

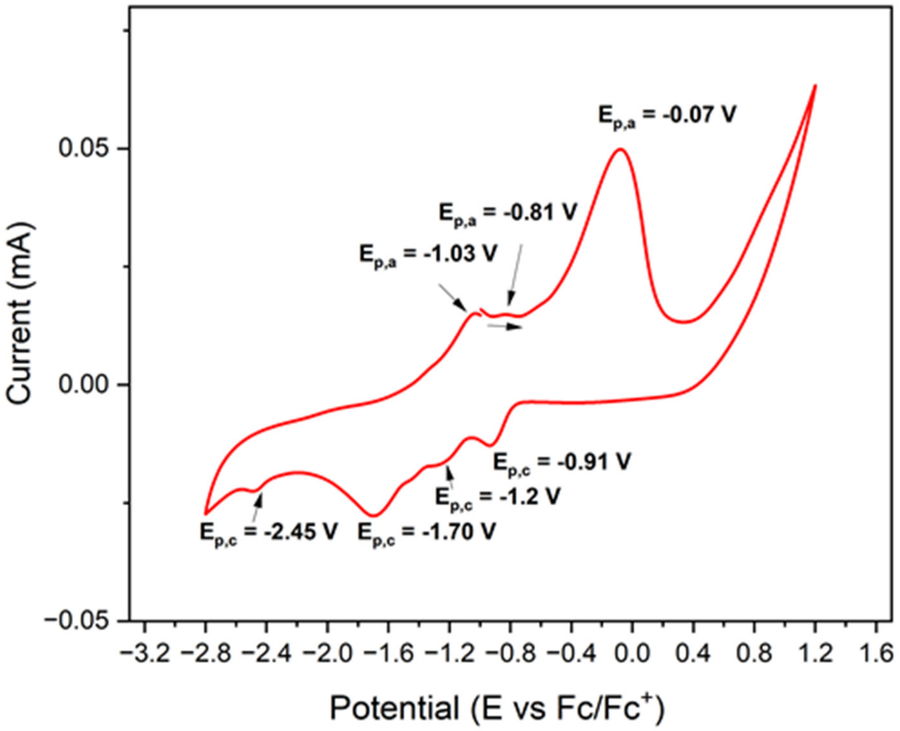


**Figure S17.** CV of **3**[BAr$^F$] at a scan anodically rate of v = 100 mVs$^{-1}$ (1 mM in THF/ 0.1 M $TBAPF_6$). Initial potential E= -0.8 V vs. Fc/Fc$^+$.

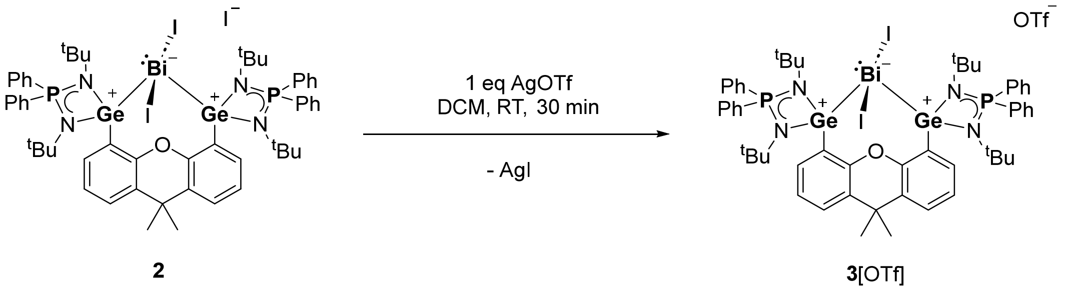


**Synthesis of compound 3[OTf].** To a mixture of compound **2** (1.6 g, 1 mmol) and AgOTf (257 mg, 1 mmol) in a 100 mL Schlenk flask was added 60 mL DCM at room temperature under stirring and dark conditions. The color of the mixture changed to orange immediately. After stirring 30 min, the mixture was filtered and volatiles were removed under vacuum to afford compound **3**[OTf] as an orange powder after dried under vacuum (1.2 g, 72% isolated yield). Orange block crystals suitable for X-ray diffraction analysis were obtained from a concentrated fluorobenzene solution at room temperature.

M.p. 199-201 °C (decomp.).

$^{1}$H NMR (200 MHz, DCM-$d_2$) $\delta$/ppm = 8.74 – 8.64 (m, 4H, Ar-*H*), 8.30 – 8.17 (m, 8H, Ar-*H*), 7.89 – 7.77 (m, 12H, Ar-*H*), 7.55 (d, *J* = 7.6 Hz, 2H, Ar-*H*), 1.63 (s, 6H, C(C$H_3$)$_2$), 1.20 (s, 36H, C(C$H_3$)$_3$).

$^{13}$C{$^{1}$H} NMR (50 MHz, DCM-$d_2$) $\delta$/ppm = 154.38 (s, Ar-*C*), 135.76 (d, *J* = 12.8 Hz, Ar-*C*), 135.10 (dd, *J* = 6.4, 3.1 Hz, Ar-*C*), 134.28 (d, *J* = 12.0 Hz, Ar-*C*), 132.54 (s, Ar-*C*), 131.57 (s, Ar-*C*), 129.96 (s, Ar-*C*), 129.62 (d, *J* = 13.1 Hz, Ar-*C*), 129.31 (d, *J* = 13.3 Hz, Ar-*C*), 128.73 (s, Ar-*C*), 126.56 (d, *J* = 34.6 Hz, Ar-*C*), 125.35 (s, Ar-*C*),12.09 (q, $^{1}J_{c,F}$ = 321 Hz, *C*$F_3$), 55.44 (s, (*C*($CH_3$)$_3$), 36.42 (s, *C*($CH_3$)$_2$), 32.94 (d, *J* = 5.7 Hz, C(*C*$H_3$)$_3$), 29.66 (br, C(*C*$H_3$)$_2$).

$^{31}$P{$^{1}$H} NMR (81 MHz, DCM-$d_2$) $\delta$/ppm = 54.60.

$^{19}$F{$^{1}$H} NMR (188 MHz, DCM-$d_2$) $\delta$/ppm = -78.81.

HR-MS (ESI): (m/z) calcd for [M-OTf]$^+$ ($C_{55}H_{68}Ge_2N_4P_2BiI_2$)$^+$: 1471.1189; found: 1471.1196.

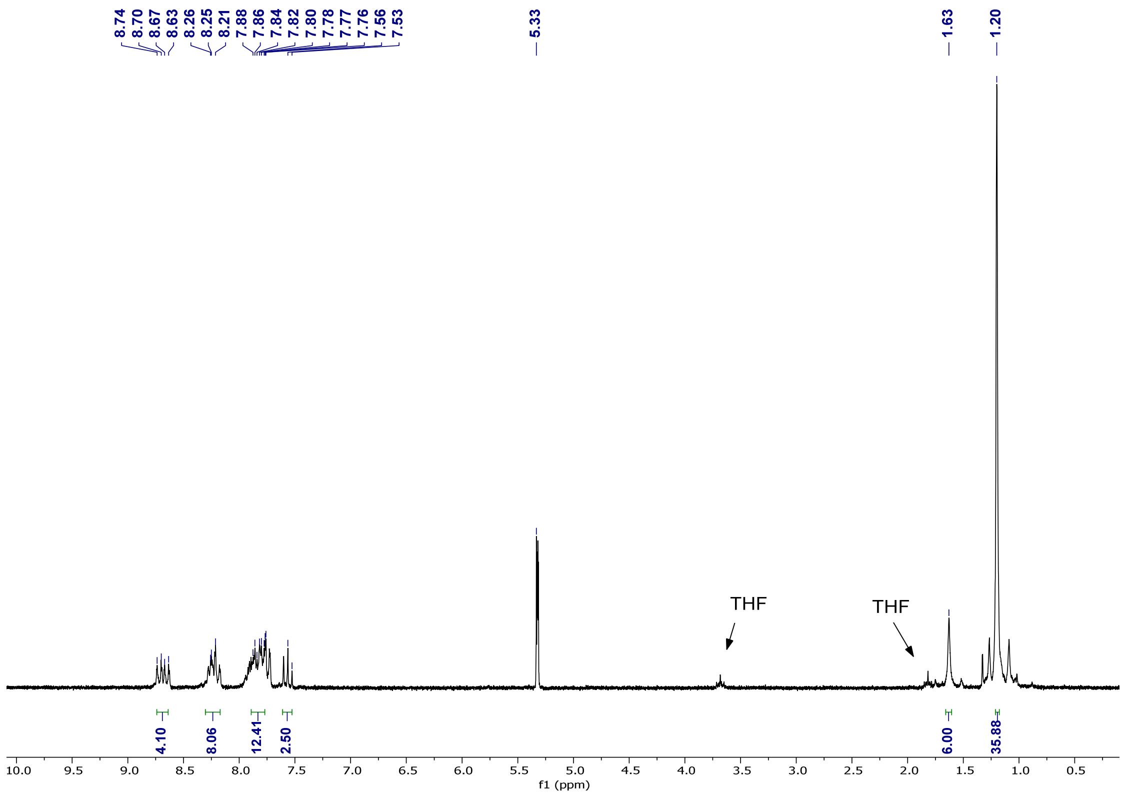


**Figure S18.** $^1$H NMR spectrum of **3**[OTf] in DCM-$d_2$.

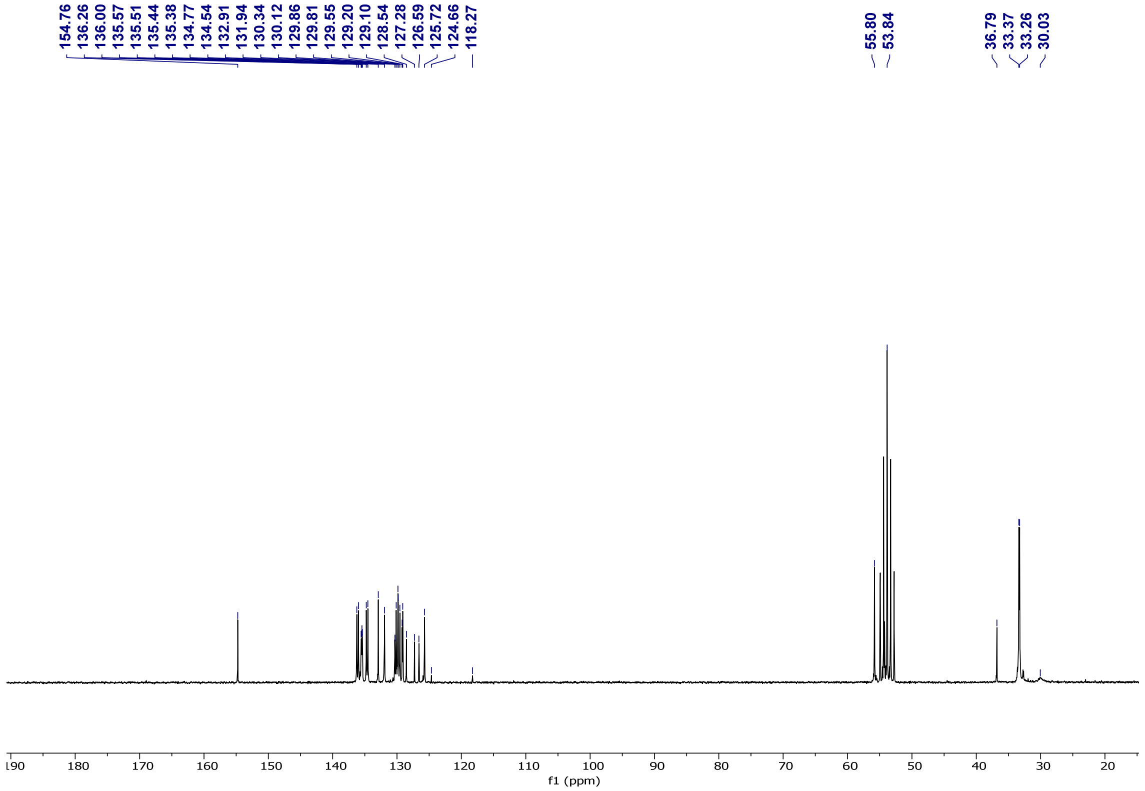


**Figure S19.** $^{13}$C{$^1$H} NMR spectrum of **3**[OTf] in DCM-$d_2$.

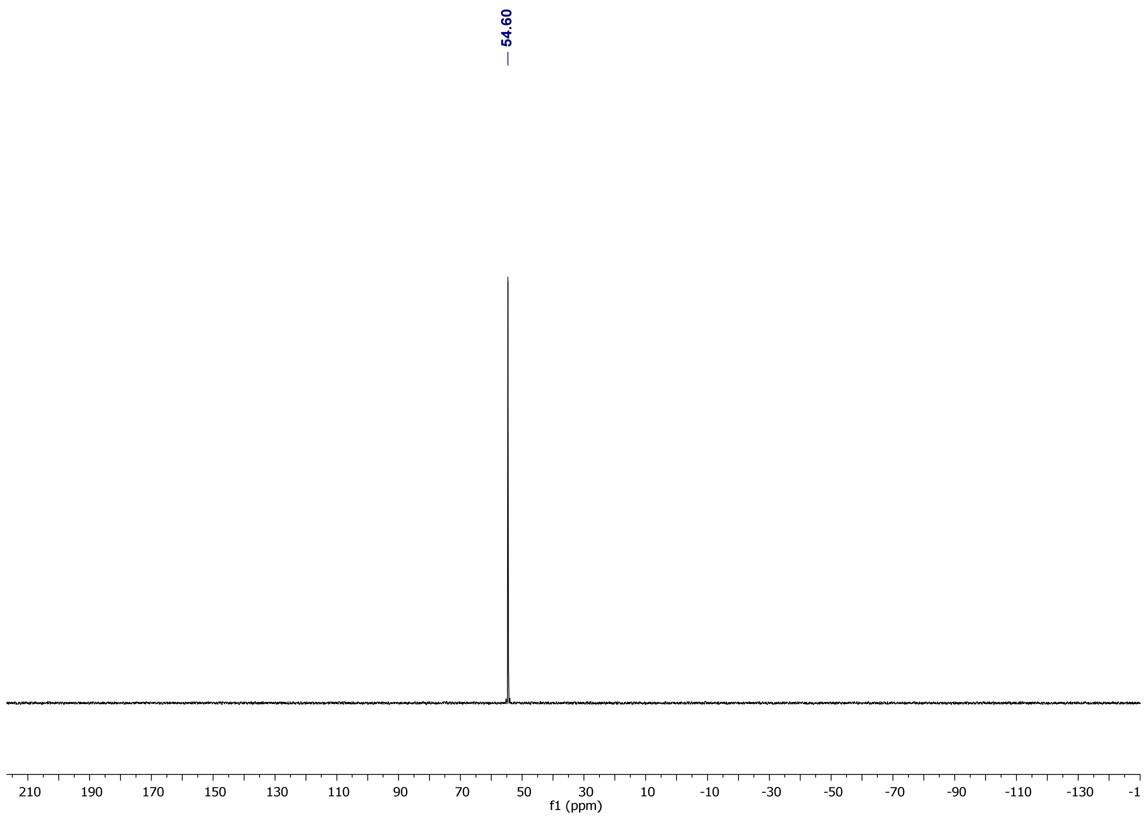


**Figure S20.** $^{31}P\{^1H\}$ spectrum of **3**[OTf] in DCM-$d_2$.

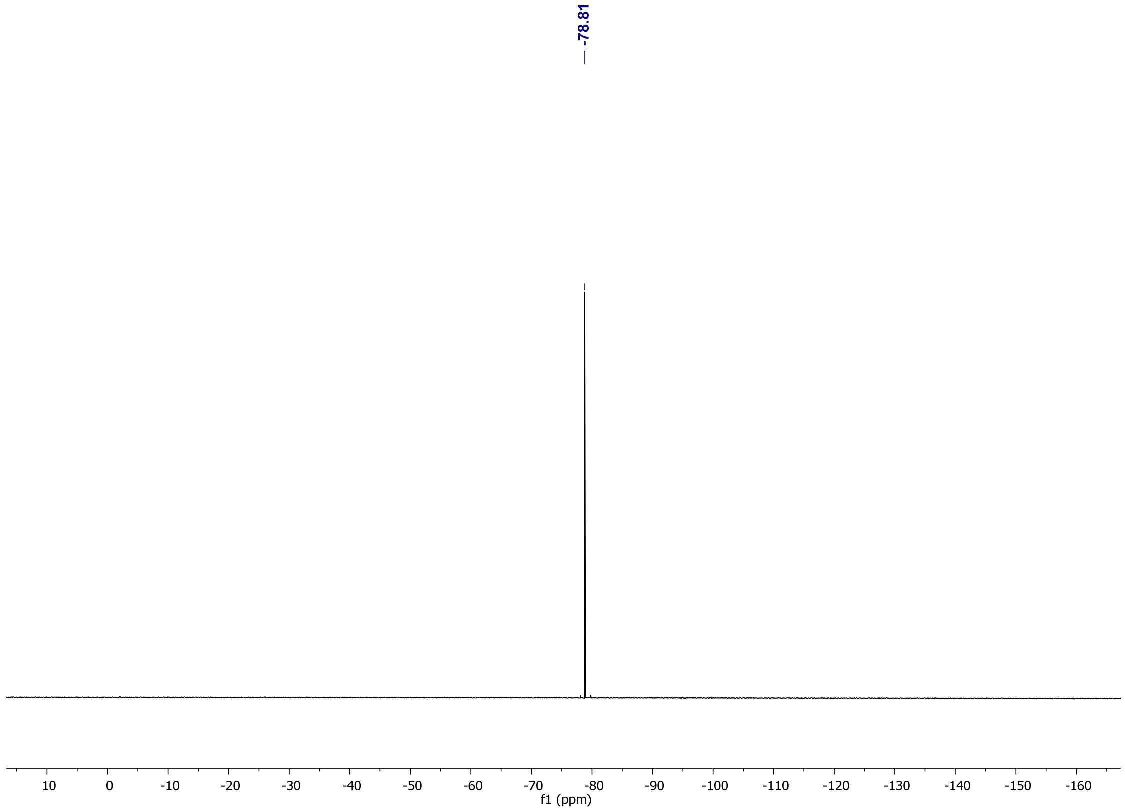


**Figure S21.** $^{19}F\{^1H\}$ spectrum of **3**[OTf] in DCM-$d_2$.

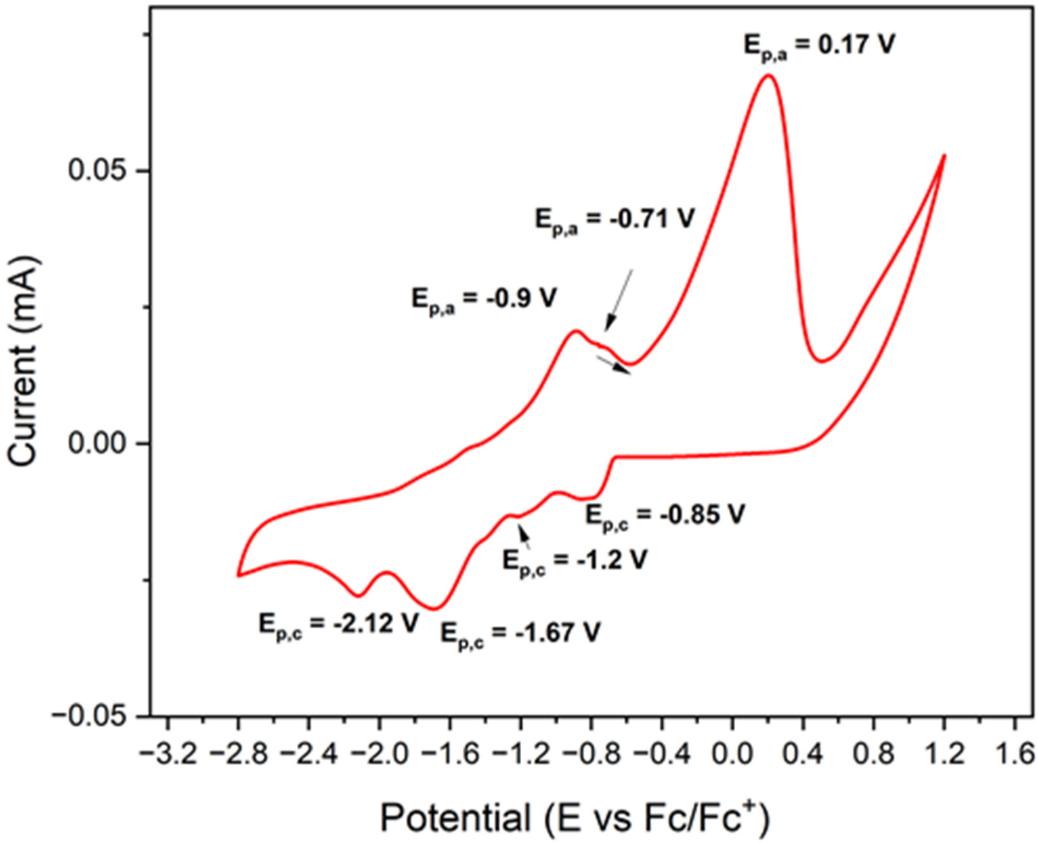


**Figure S22.** CV of **3**[OTf] at a scan anodically rate of v = 100 mVs$^{-1}$ (1 mM in THF/ 0.1 M TBAPF$_6$). Initial potential E= -0.8 V vs. Fc/Fc$^+$.

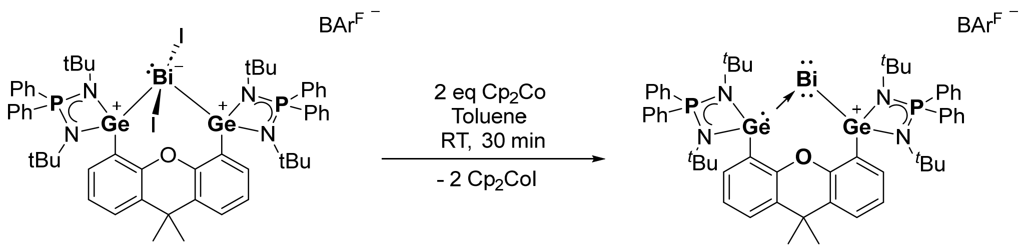


**3**[BArF]

**4**[BArF]

**Synthesis of compound 4[BAr$^F$].** To a mixture of compound **3**[BAr$^F$] (1.17g, 0.5 mmol) and $Cp_2Co$ (189 mg, 1 mmol) in a 100 mL Schlenk flask was added 50 mL toluene at room temperature under stirring. The color of the mixture from orange changed to red slowly. After stirring 30 mins, the dark red mixture was filtered and the residue was washed with toluene (10 mL x 3). The all volatiles were removed and recrystallization from DCM/hexane formed compound **4**[BAr$^F$] as red crystals (863 mg, 83% isolated yield). Red crystals suitable for X-ray diffraction analysis were obtained from a DCM/hecane solution at -30 ºC.

M.p. 123-127 ºC (decomp.).

$^1$H NMR (200 MHz, DCM-$d_2$) $\delta$/ppm = 8.25 – 8.11 (m, 8H, Ar-*H*), 8.07 (dd, *J* = 7.3, 1.7 Hz, 2H, Ar-*H*), 7.86 – 7.66 (m, 20H, Ar-*H*), 7.58 (dd, *J* = 7.7, 1.6 Hz, 6H, Ar-*H*), 7.49 – 7.40 (m, 2H, Ar-*H*), 1.62 (s, 6H, C(C$H_3$)$_2$), 1.08 (s, 36H, C(C$H_3$)$_3$).

$^{13}$C{$^1$H} NMR (101 MHz, DCM-$d_2$) $\delta$/ppm = 162.18 (dd, *J* = 99.6, 49.8 Hz, BAr$^F$-*C*), 155.90 (s, Ar-*C*), 142.09 (s, Ar-*C*), 135.21 (s, BAr$^F$-*C*), 134.78 (d, *J* = 11.7 Hz, Ar-*C*), 134.49 (d, *J* = 2.9 Hz, Ar-*C*), 134.35 (d, *J* = 3.1 Hz, Ar-*C*), 134.22 (d, *J* = 11.7 Hz, Ar-*C*), 132.42 (s, Ar-*C*), 131.69 (d, *J* = 96.9 Hz, Ar-*C*), 130.47 (s, Ar-*C*), 129.94 (d, *J* = 94.8 Hz, Ar-*C*), 129.73 (d, *J* = 12.6 Hz, Ar-*C*), 129.45 (d, *J* = 12.5 Hz, Ar-*C*), 129.15 (m, BAr$^F$-*C*), 128.10 (s, Ar-*C*), 125.00 (d, $J_{C\text{-}F}$ = 272.2 Hz, BAr$^F$-*C*$F_3$ ), 124.37 (s, Ar-*C*), 117.87 (m, BAr$^F$-*C*), 55.03 (s, *C*($CH_3$)$_3$), 36.94 (s, *C*($CH_3$)$_2$), 34.02 (d, *J* = 5.9 Hz, C(*C*$H_3$)$_3$), 30.04 (s, C(*C*$H_3$)$_2$).

$^{31}$P{$^1$H} NMR (81 MHz, DCM-$d_2$) $\delta$/ppm = 44.51.

$^{11}$B{$^1$H} NMR (64 MHz, DCM-$d_2$) $\delta$/ppm = -6.58.

$^{19}$F{$^1$H} NMR (188 MHz, DCM-$d_2$) $\delta$/ppm = -62.87.

HR-MS(ESI): (m/z) calcd for [M-BAr$^F$]$^+$ ($C_{55}H_{68}Ge_2N_4P_2Bi$)$^+$: 1217.3099; found: 1217.3105.

UV/Vis (THF): $\lambda_{max}$ = 484 nm.

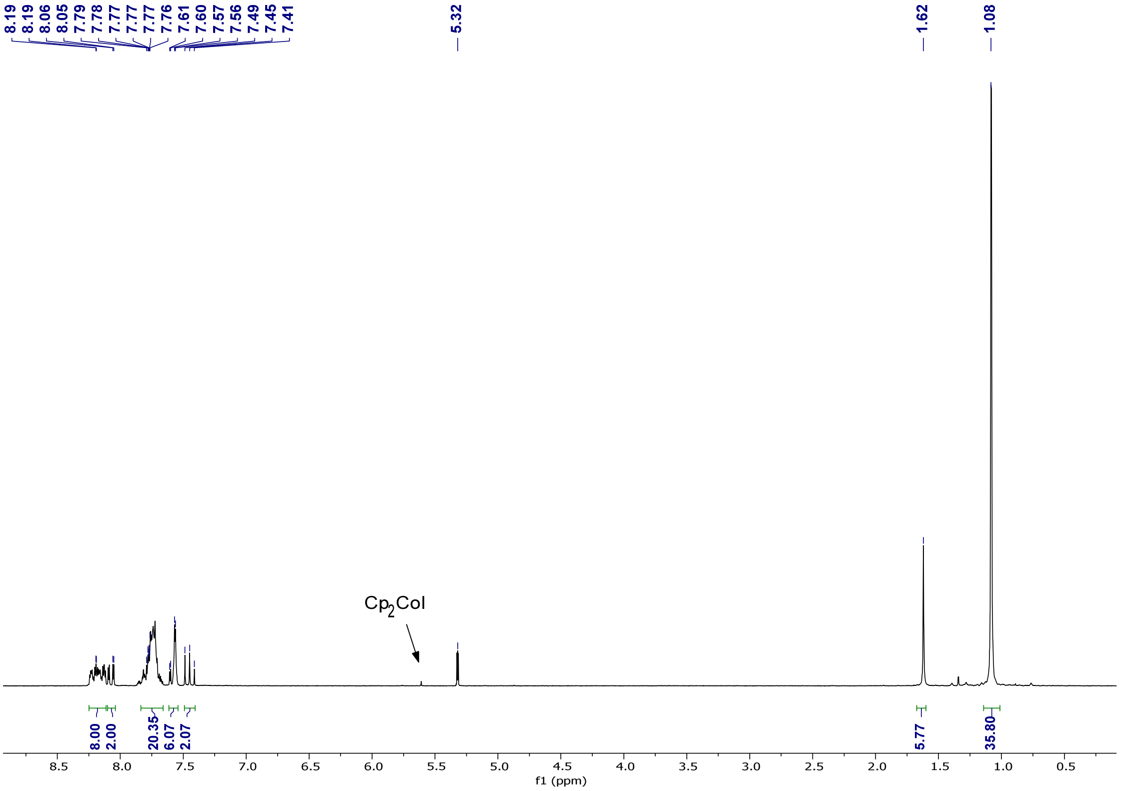


**Figure S23.** $^{1}$H NMR spectrum of **4**[BAr$^{F}$] in DCM-$d_2$.

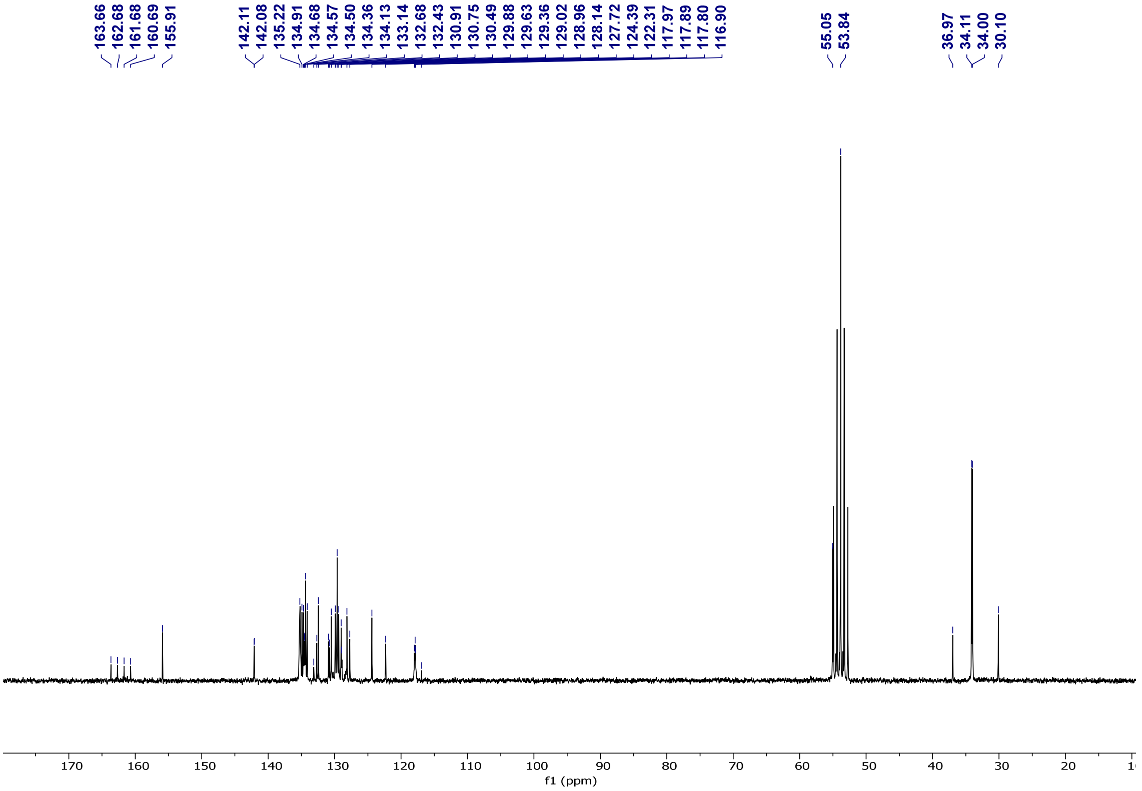


**Figure S24.** $^{13}$C{$^{1}$H} NMR spectrum of **4**[BAr$^{F}$] in DCM-$d_2$.

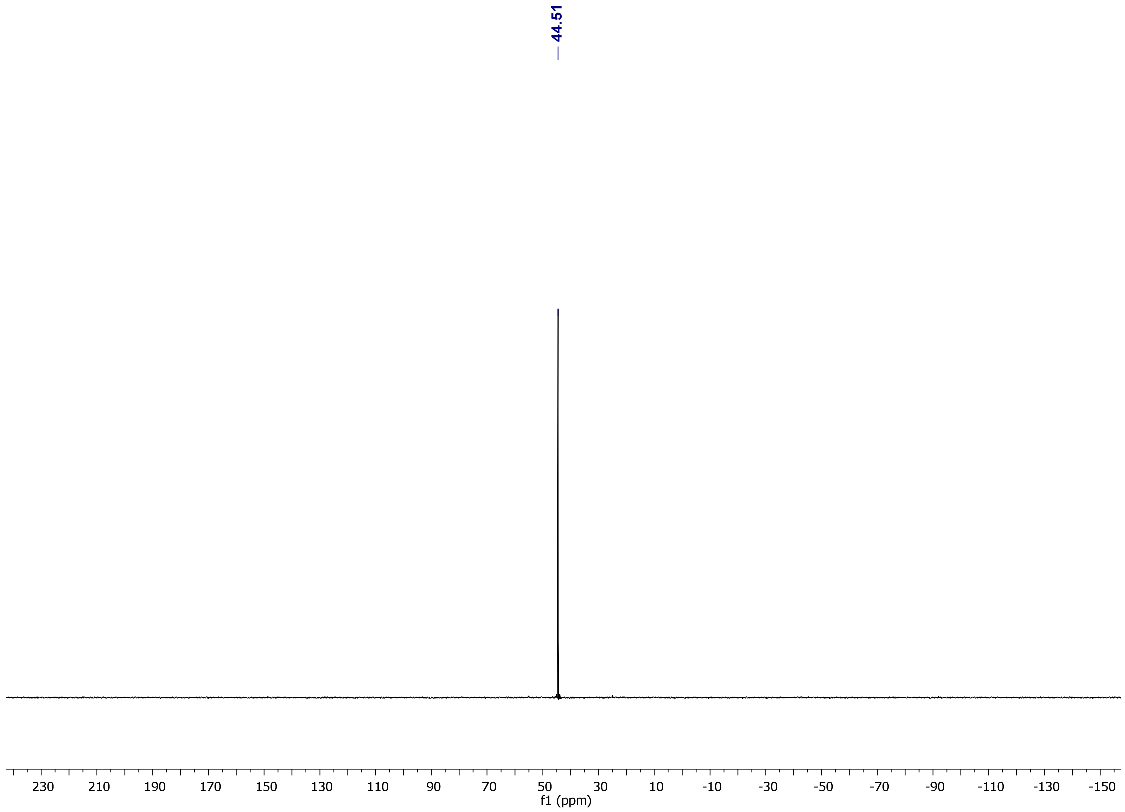


**Figure S25.** $^{31}P\{^{1}H\}$ NMR spectrum of **4**[BAr$^{F}$] in DCM-$d_2$.

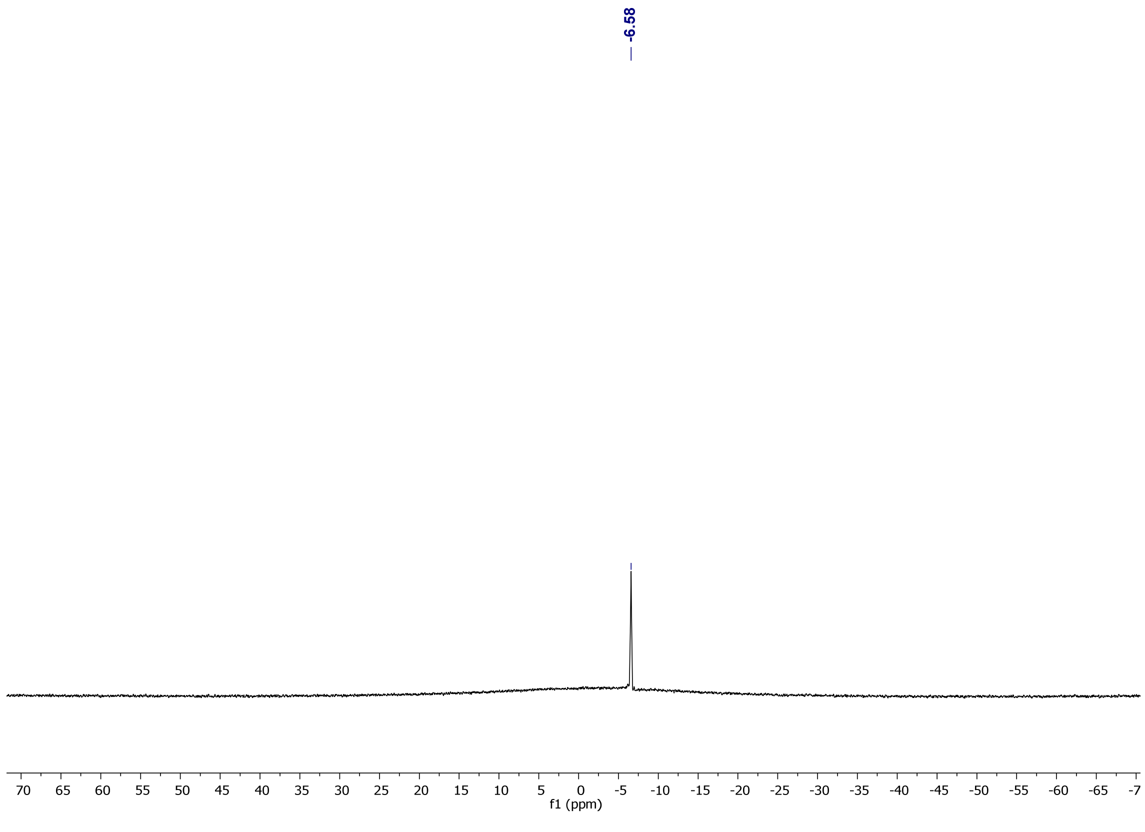


**Figure S26.** $^{11}B\{^{1}H\}$ NMR spectrum of **4**[BAr$^{F}$] in DCM-$d_2$.

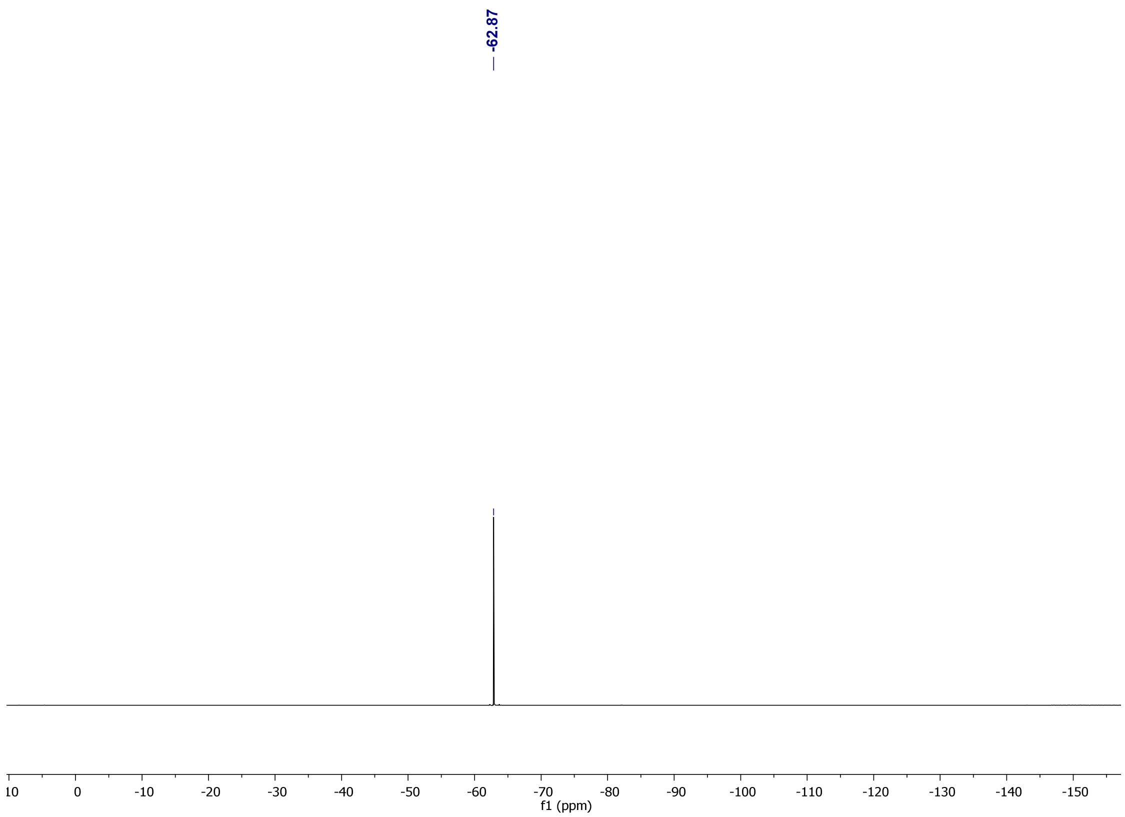


**Figure S27.** $^{19}F\{^1H\}$ spectrum of **4**[BAr$^F$] in DCM-$d_2$.

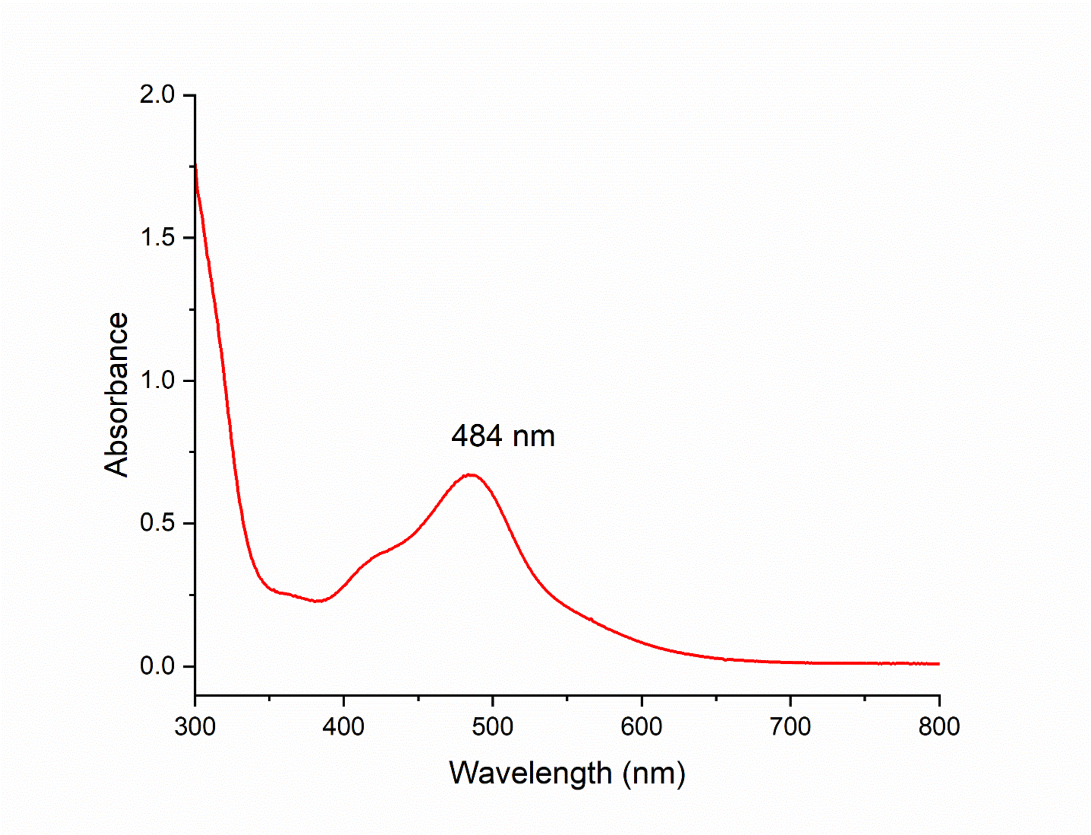


**Figure S28.** UV-Vis spectrum of **4**[BAr$^F$] in THF.

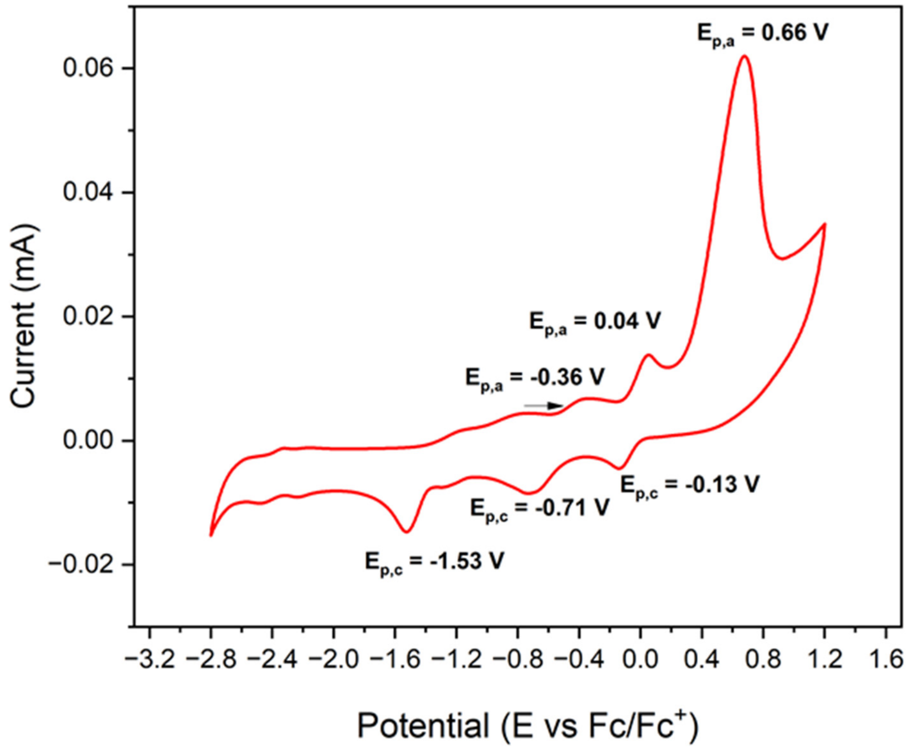


**Figure S29.** CV of **4**[BAr$^F$] at a scan anodically rate of v = 100 mVs$^{-1}$ (1 mM in THF/ 0.1 M TBAPF$_6$). Initial potential E= -0.8 V vs. Fc/Fc$^+$.

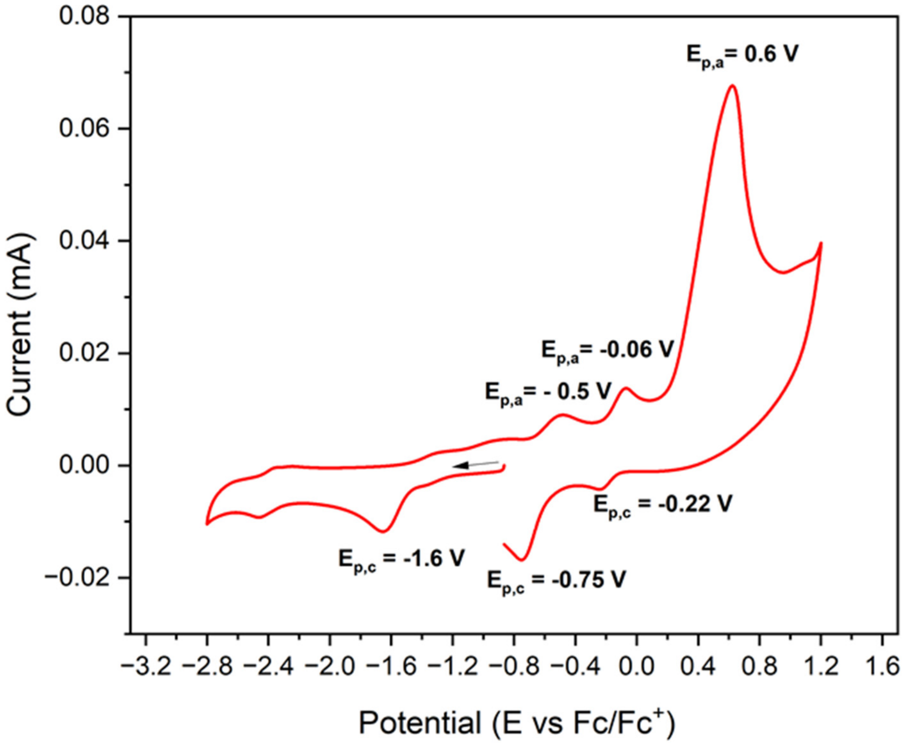


**Figure S30.** CV of complex **4**[BAr$^F$] at first scan cathodically rate of v = 100 mVs$^{-1}$. (1 mM in THF/ 0.1 M TBAPF$_6$). Initial potential E = -0.8 V vs. Fc/Fc$^+$.

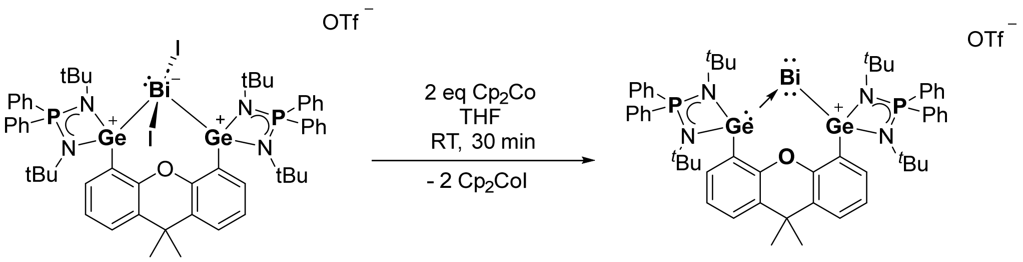


**Synthesis of Compound 4[OTf].** To a mixture of compound **3**[OTf] (685 mg, 0.5 mmol) and $Cp_2Co$ (189 mg, 1 mmol) in a 100 mL Schlenk flask was added 50 mL THF at room temperature under stirring. The color of the mixture from orange changed to red slowly. After stirring 30 min, the dark red mixture was filtered and the residue was washed with THF (10 mL x 3). The all volatiles were removed under vacuum from the combined filtrate to give red residue. Recrystallization from DCM/hexane furnished compound **4**[OTf] as brown-red crystals with some $Cp_2CoI$ yellow powder (307 mg, 45% isolated yield). Red crystals suitable for X-ray diffraction analysis were obtained from a DCM/hexane solution at -30°C.

M.p. 155-157 °C (decomp.).

$^1$H NMR (200 MHz, DCM-$d_2$) $\delta$/ppm = 8.24 – 8.12 (m, 8H, Ar-*H*), 8.06 (dd, $J$ = 7.3, 1.6 Hz, 2H, Ar-*H*), 7.84 – 7.72 (m, 12H, Ar-*H*), 7.58 (dd, $J$ = 7.7, 1.6 Hz, 2H, Ar-*H*), 7.45 (t, $J$ = 7.5 Hz, 2H, Ar-*H*), 1.62 (s, 6H, C(C*H*$_3$)$_2$), 1.08 (s, 36H, C(CH$_3$)$_3$).

$^{13}$C{$^1$H} NMR (101 MHz, DCM-$d_2$) $\delta$/ppm = 155.85 (s, Ar-*C*), 142.11 (s, Ar-*C*), 134.77 (d, $J$ = 11.5 Hz, Ar-*C*), 134.60 (d, $J$ = 3.5 Hz, Ar-*C*), 134.45 (d, $J$ = 2.9 Hz, Ar-*C*), 134.22 (d, $J$ = 11.5 Hz, Ar-*C*), 132.38 (s, Ar-*C*), 131.64 (d, $J$ = 97.0 Hz, Ar-*C*), 130.49 (s, Ar-*C*), 129.89 (d, $J$ = 95.0 Hz, Ar-*C*), 129.79 (d, $J$ = 12.4 Hz, Ar-*C*), 129.53 (d, $J$ = 12.5 Hz, Ar-*C*), 128.13 (s, Ar-*C*), 124.42 (s, Ar-*C*), 121.54 (q, $^1J_{C,F}$ = 321 Hz, *C*F$_3$), 55.02 (s, *C*(CH$_3$)$_3$), 36.93 (s, *C*(CH$_3$)$_2$), 34.06 (d, $J$ = 5.6 Hz, C(*C*H$_3$)$_3$), 30.13 (s, C(*C*H$_3$)$_2$).

$^{31}$P{$^1$H} NMR (81 MHz, DCM-$d_2$) $\delta$/ppm = 44.54.

$^{19}$F{$^1$H} NMR (188 MHz, DCM-$d_2$) $\delta$/ppm = -78.90.

HR-MS(ESI): (m/z) calcd for [M-OTf]$^+$ ($C_{55}H_{68}Ge_2N_4P_2Bi$)$^+$: 1217.3099; found: 1217.3097.

UV/Vis (THF): $\lambda_{max}$ = 486 nm.

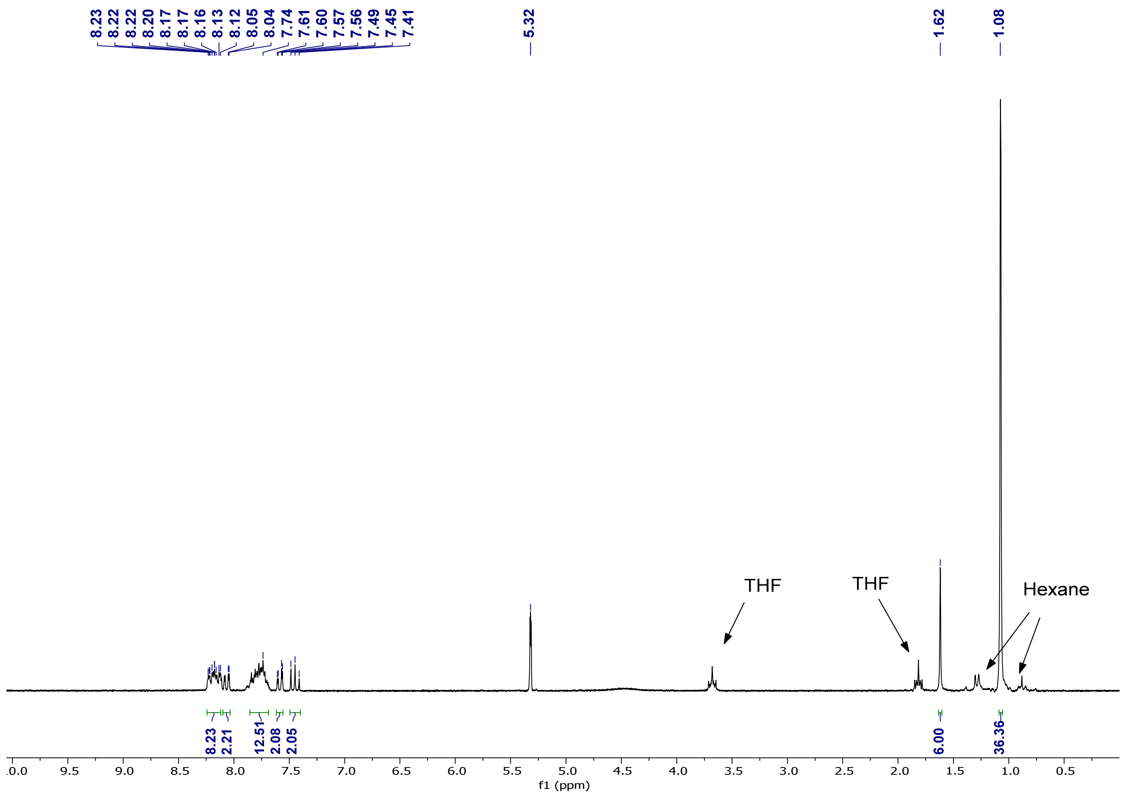


**Figure S31.** $^{1}$H NMR spectrum of **4**[OTf] in DCM-$d_2$.

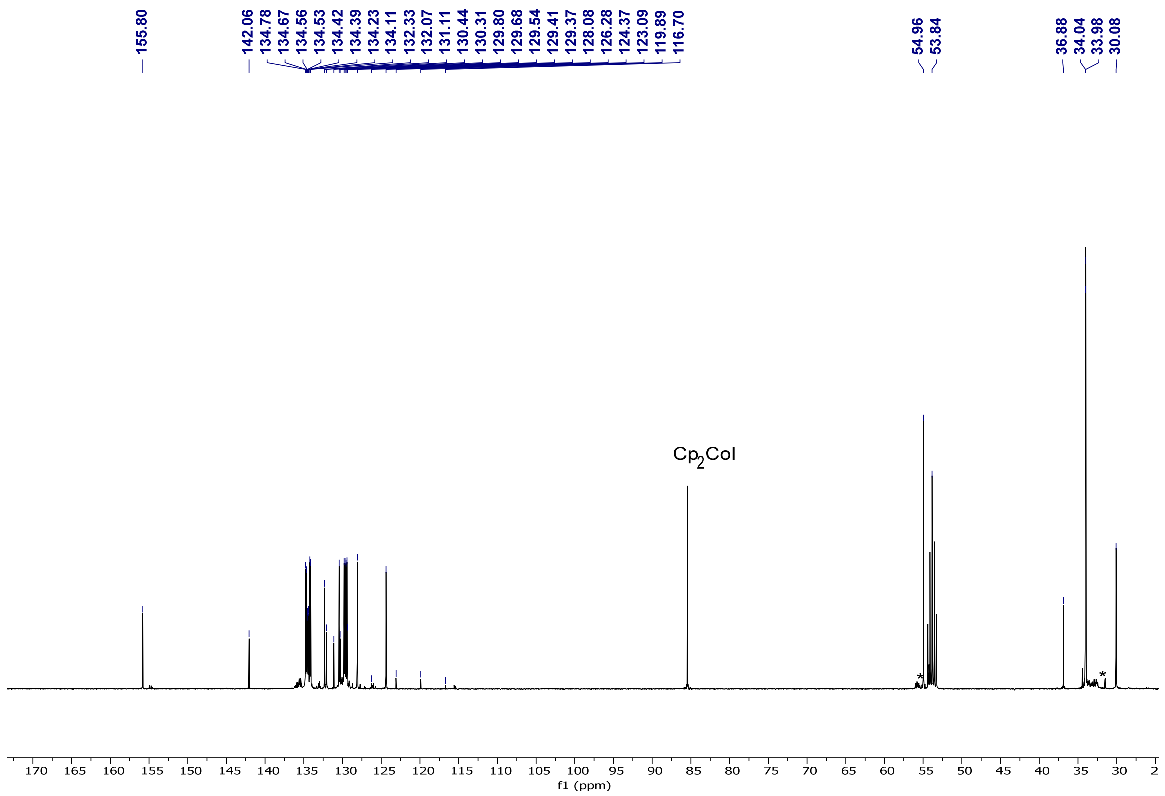


**Figure S32.** $^{13}$C{$^{1}$H} NMR spectrum of **4**[OTf] in DCM-$d_2$. *Unidentified impurities.

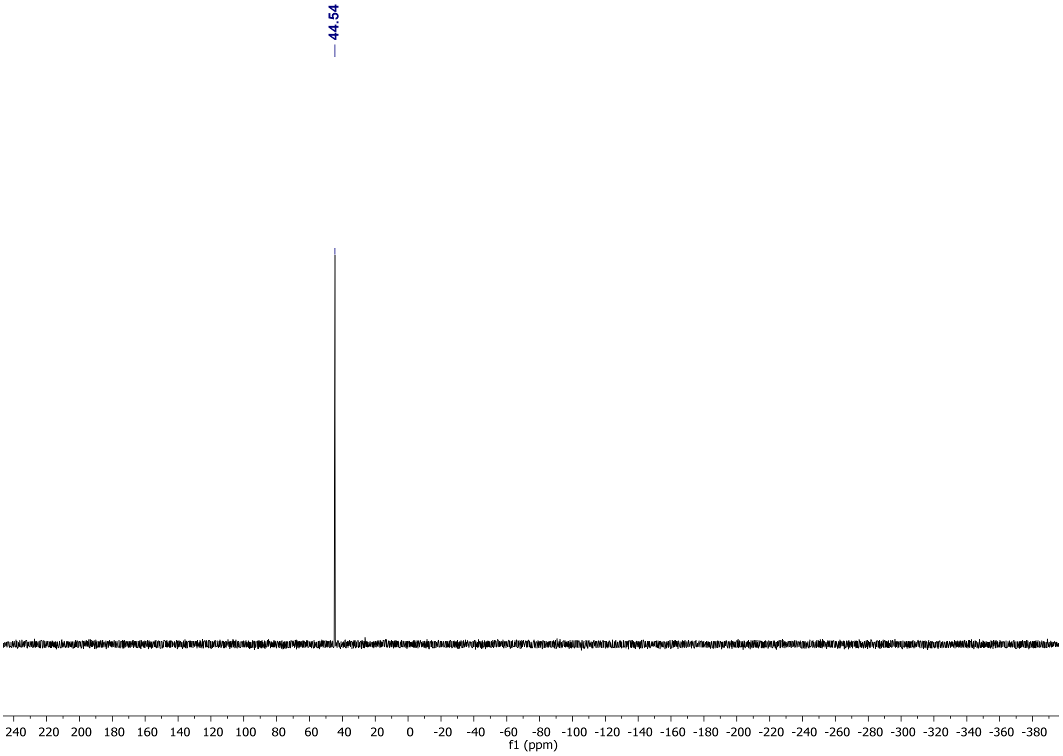


**Figure S33.** $^{31}P\{^{1}H\}$ NMR spectrum of **4**[OTf] in DCM-$d_2$.

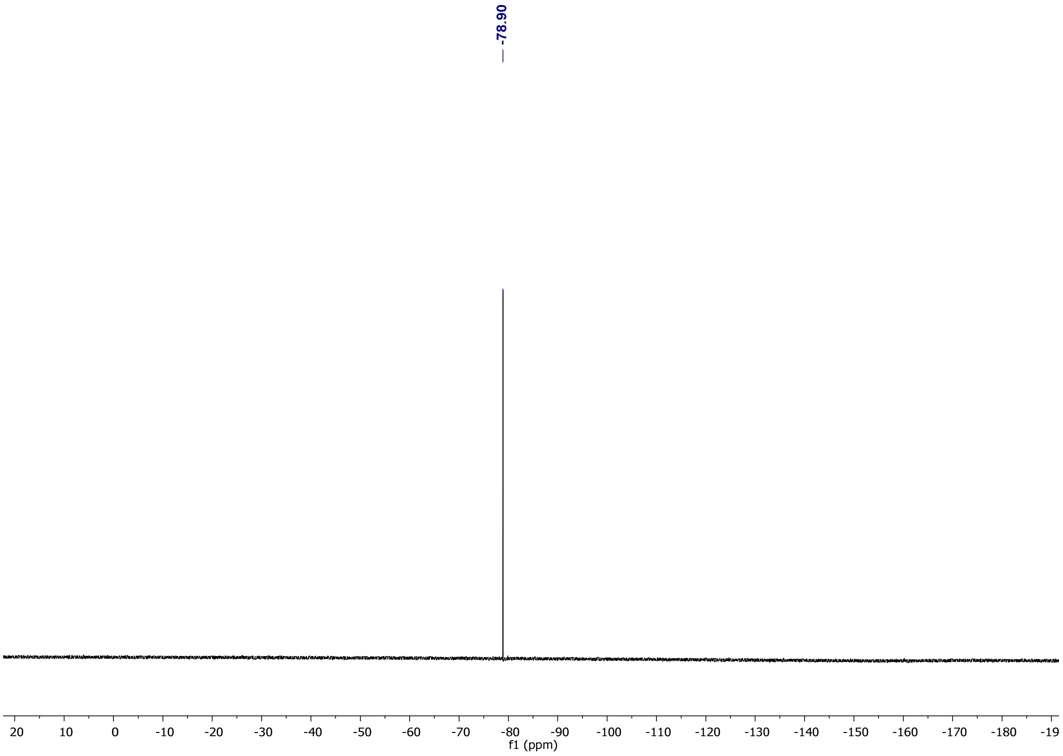


**Figure S34.** $^{19}F\{^{1}H\}$ spectrum of **4**[OTf] in DCM-$d_2$.

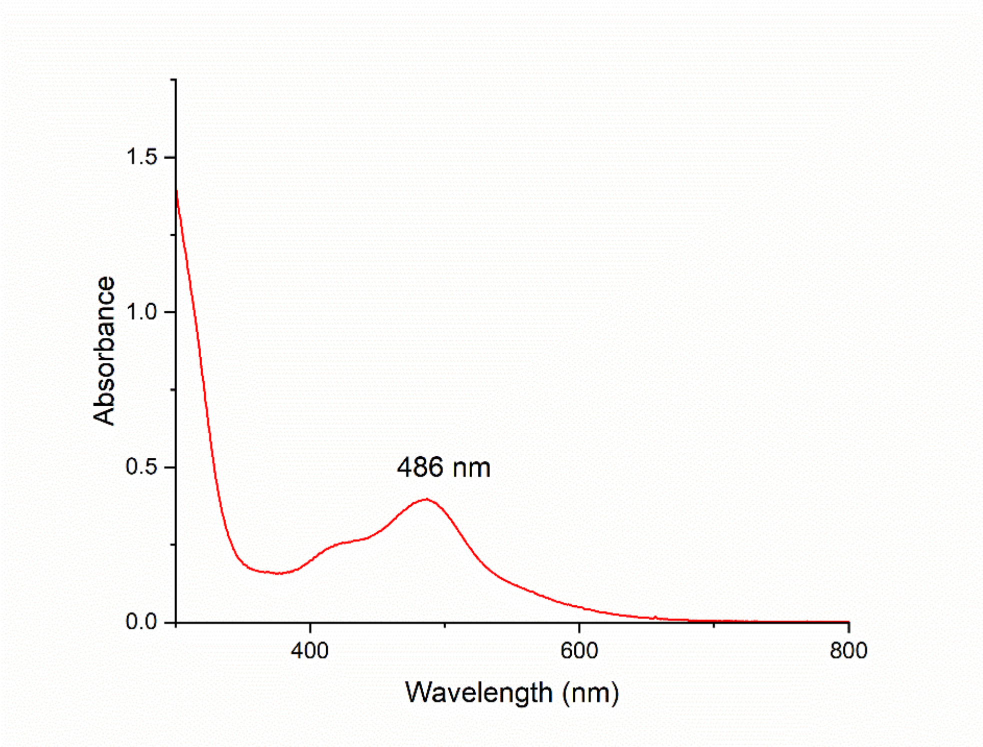


**Figure S35.** UV-Vis spectrum of **4**[OTf] in THF.

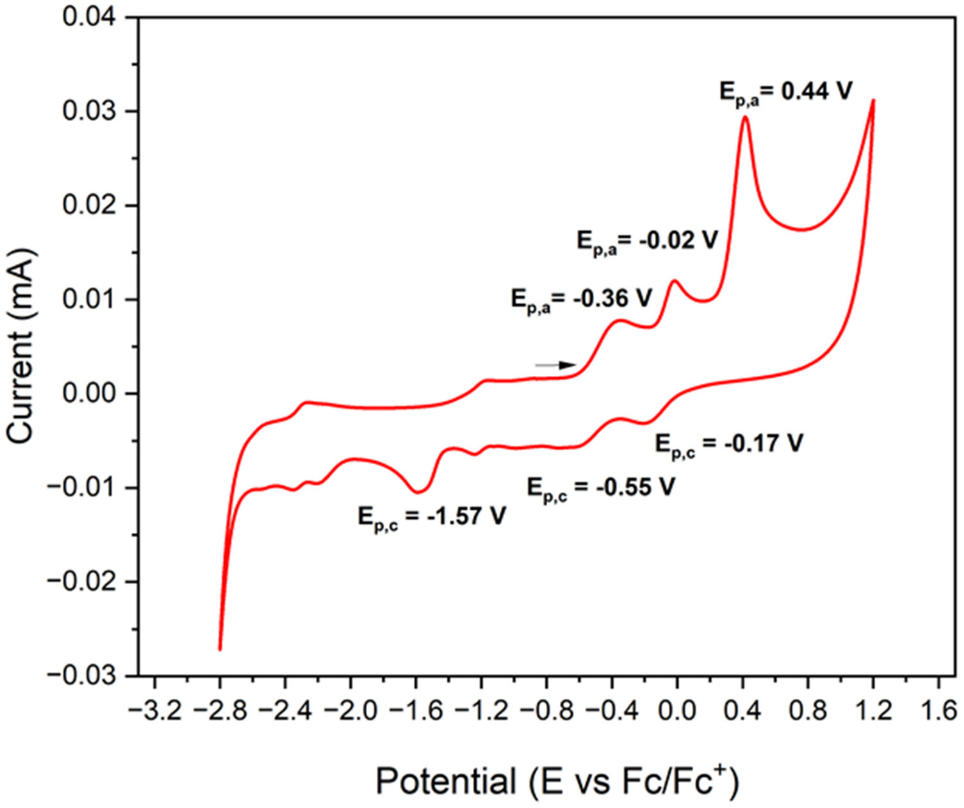


**Figure S36.** CV of **4**[OTf] at a scan anodically rate of v = 100 mVs$^{-1}$ (1 mM in THF/ 0.1 M TBAPF$_6$). Initial potential E= -0.8 V vs. Fc/Fc$^+$.

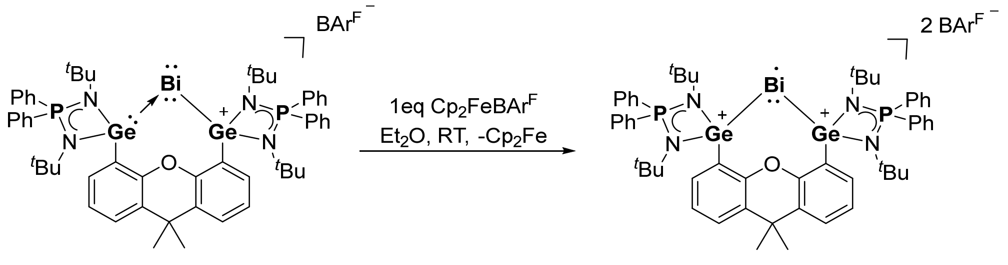


**4**[BAr$^F$] **5**[BAr$^F$]$_2$

**Synthesis of compound 5[BAr$^F$]$_2$.** To a mixture of compound **4**[BAr$^F$] (1 g, 0.5 mmol) and $Cp_2FeBAr^F$ (525 mg, 0.5 mmol) in a 50 mL Schlenk flask was added 25 mL $Et_2O$ at room temperature under stirring. The color of the mixture from red changed to brown slowly. After stirring 30 min, the all volatiles were removed and the residue was washed with hexane (20 mL x 3). Recrystallization from fluorobenzene formed compound **5**[BAr$^F$]$_2$ as brown crystals. Brown block crystals suitable for X-ray diffraction analysis were obtained from a concentrated fluorobenzene solution at -30 °C.

M.p. 92-95 °C (decomp.).

$^1$H NMR (500 MHz, THF-$d_8$) $\delta$/ppm = 8.78 (br, Ar-*H*), 8.67 (br, Ar-*H*), 7.74 (br, BAr$^F$), 7.57 (br, Ar-*H*), 7.52 (br, BAr$^F$), 6.73 (br, Ar-*H*), 5.82 (s, $CH_3$), 1.80 (br, $CH_3$).

$^{19}$F NMR (471 MHz, THF-$d_8$) $\delta$/ppm = -64.56.

$^{11}$B NMR (160 MHz, THF-$d_8$) $\delta$/ppm = -6.54.

Elemental analysis calcd for $C_{119}H_{92}B_2BiF_{48}Ge_2N_4OP_2$: C, 48.55; H, 3.15; N, 1.90. Found: C, 47.74; H, 2.93; N, 1.73.

UV/Vis (THF): $\lambda_{max}$ = 483 nm and 762 nm.

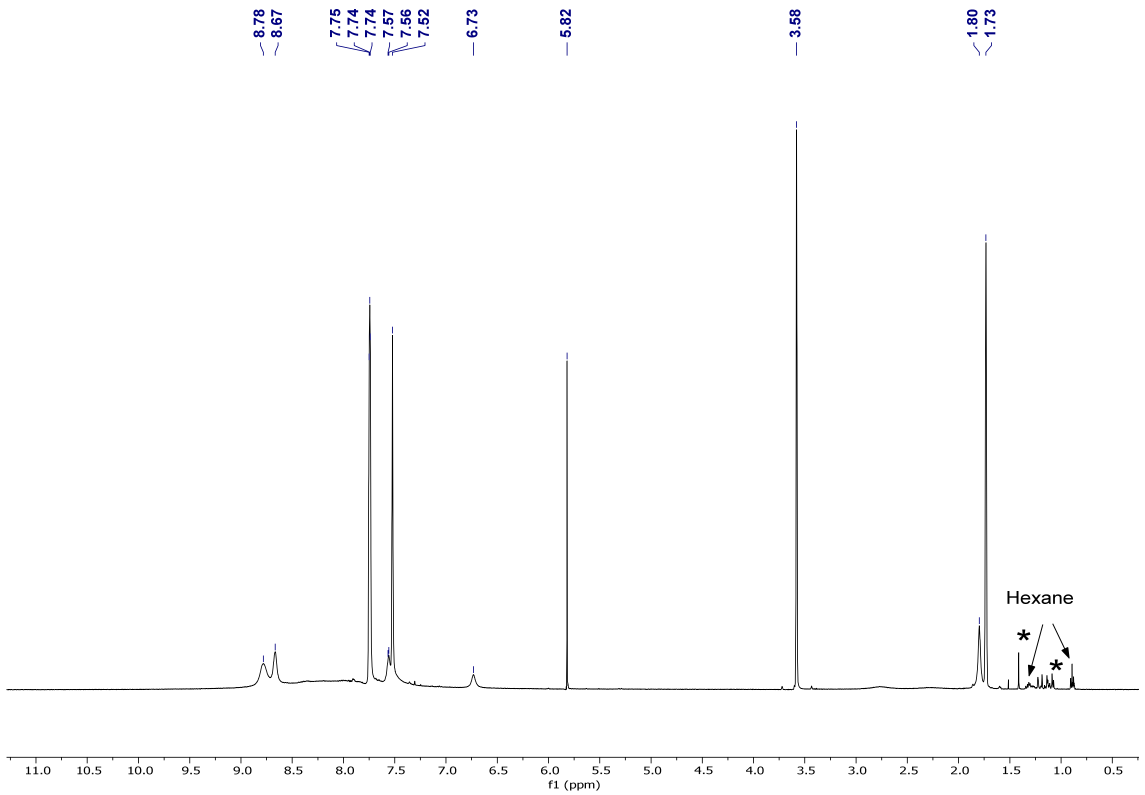


**Figure S37.** $^{1}H$ NMR spectrum of **5**[BAr$^F$]$_2$ in THF-$d_8$. ⋆Unidentified impurities.

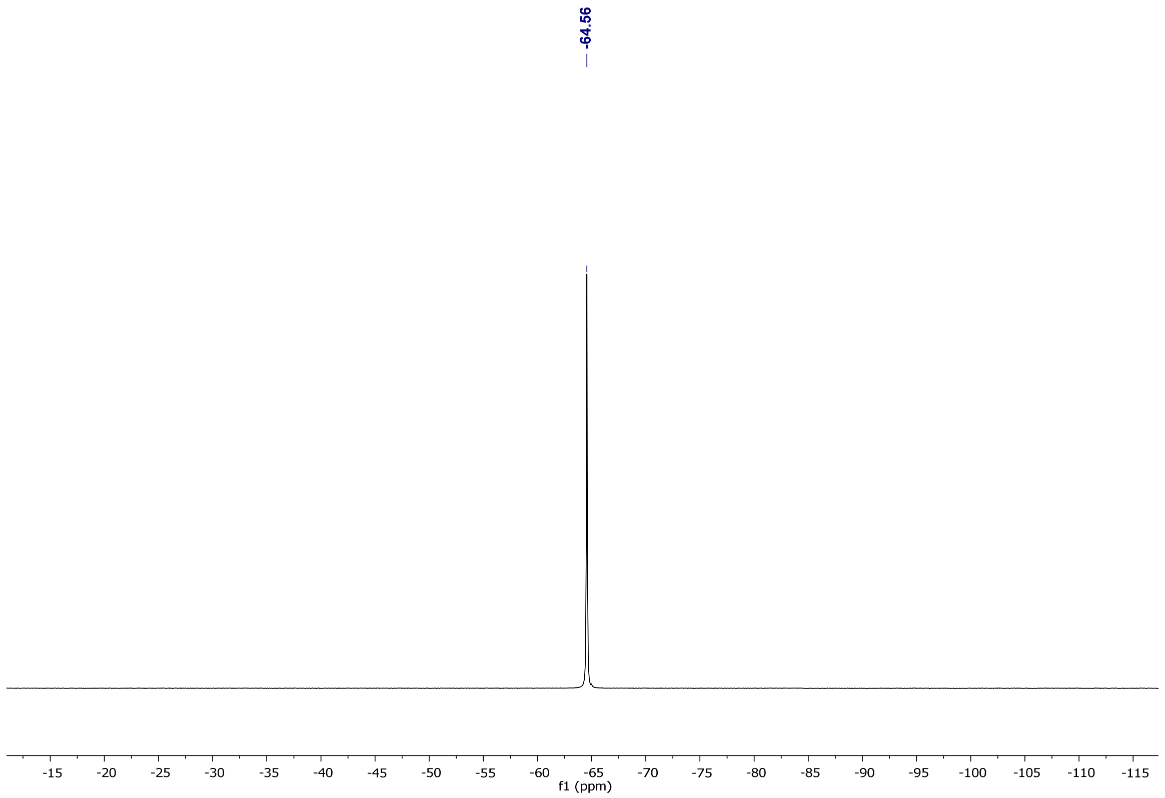


**Figure S38.** $^{19}F\{^{1}H\}$ spectrum of **5**[BAr$^F$]$_2$ in THF-$d_8$.

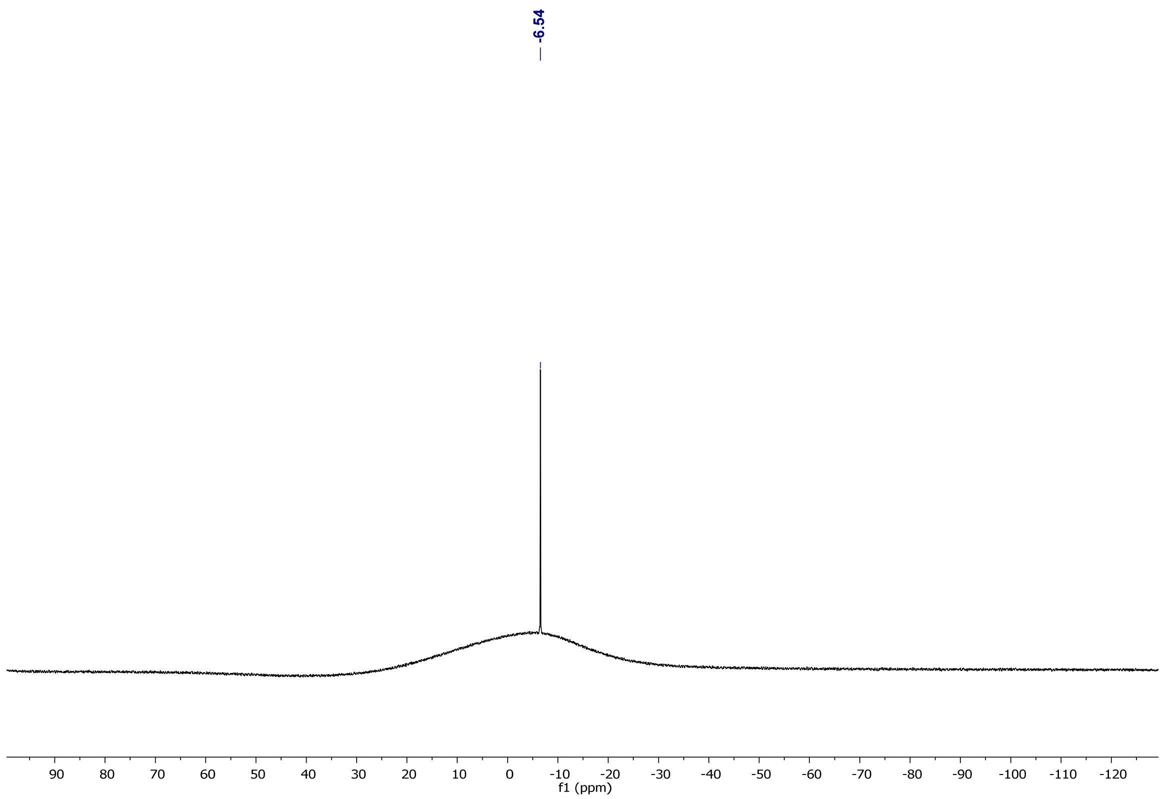


**Figure S39.** $^{11}B\{^{1}H\}$ NMR spectrum of **5**[BAr$^{F}$]$_2$ in THF-$d_8$.

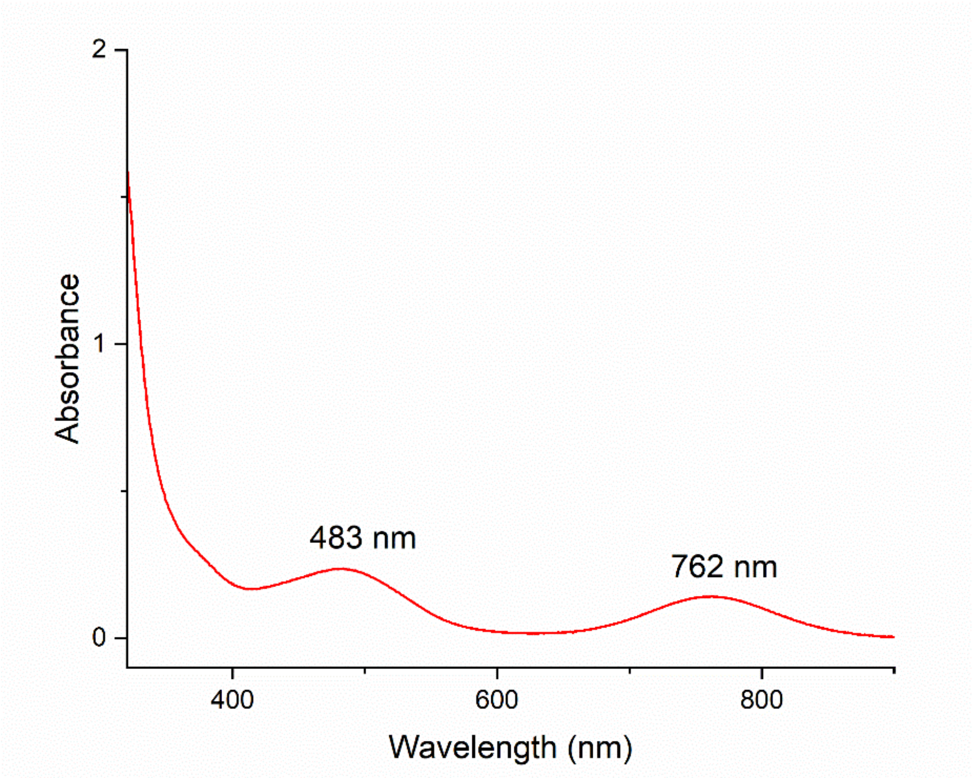


**Figure S40.** UV-Vis spectrum of **5** in THF.

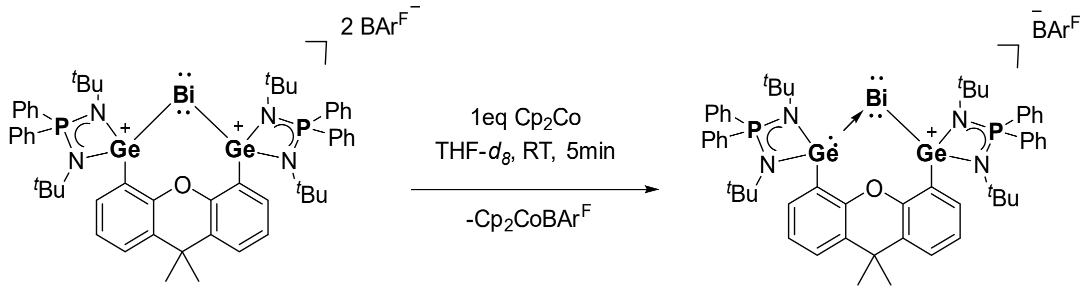


**5**[BAr$^F$]$_2$ **4**[BAr$^F$]

**5[BArF]$_2$ with 1eq Cp$_2$Co in THF-*d$_8$* restored 4[BArF].** To a mixture of compound **5**[BAr$^F$]$_2$ (29.5 mg 0.01mol) and Cp$_2$Co (1.89 mg, 0.01 mmol) in a NMR tube was added 0.5 mL THF-d$_8$ at room temperature. The color of the mixture from brown changed to red immediately.

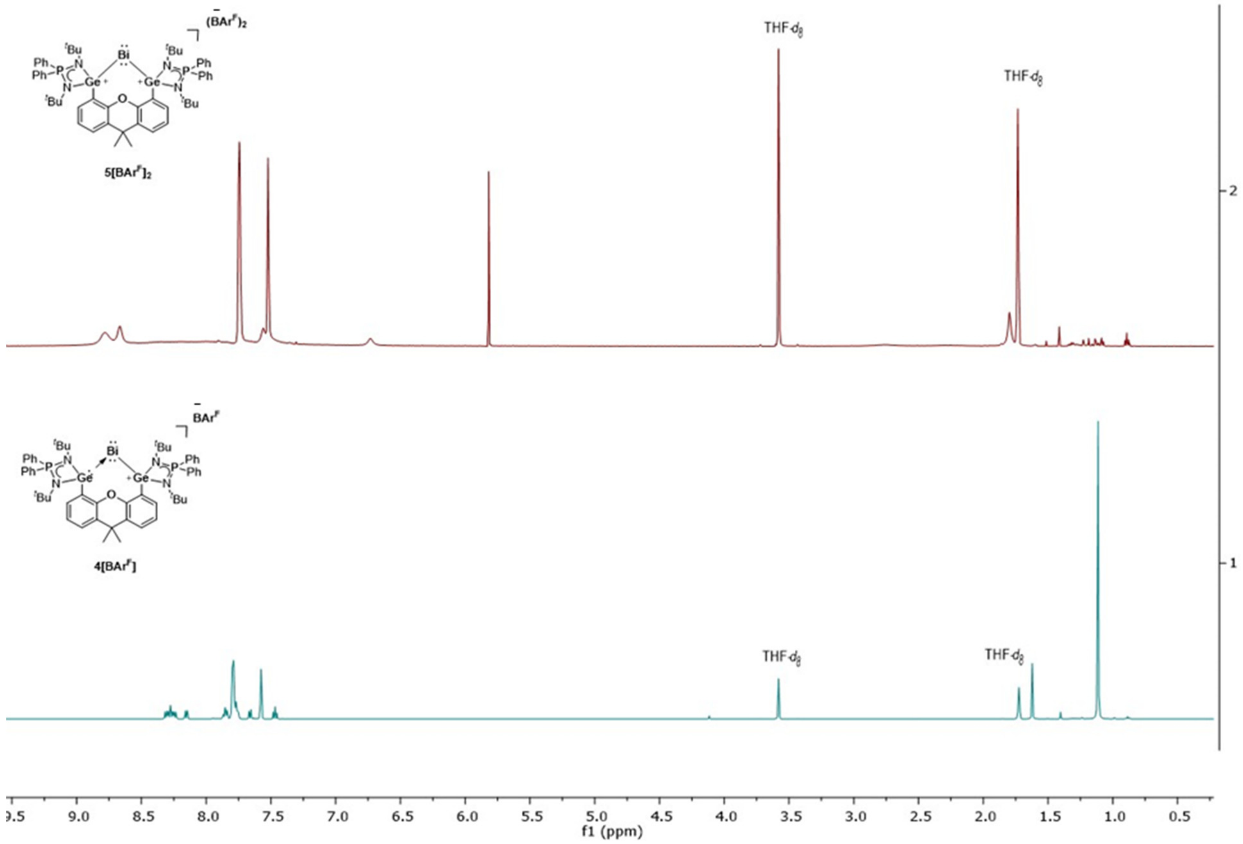


**Figure S41.** $^1$H spectra of the reduction of **5**[BAr$^F$]$_2$ (top) with 1eq Cp$_2$Co in THF-*d$^8$* restored **4**[BAr$^F$] (bottom) in 5 min.

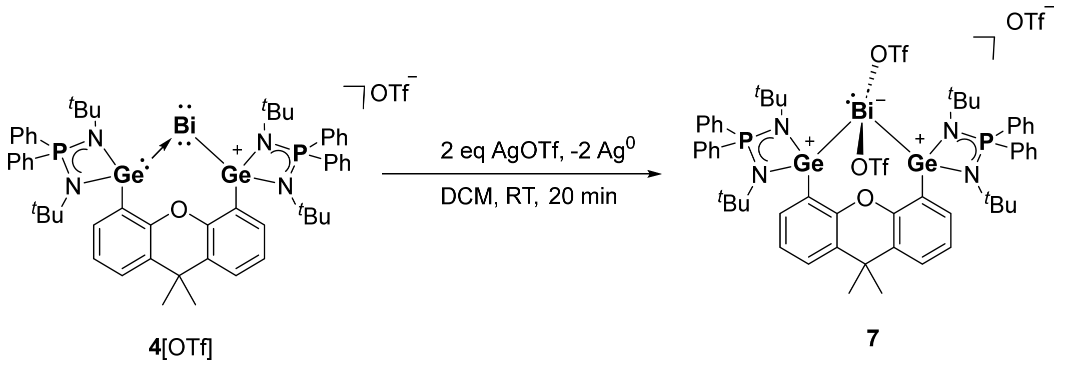


**Synthesis of compound 7.** To a mixture of compound **4**[OTf] (685 mg, 0.5 mmol) and AgOTf (189 mg, 1 mmol) in a 100 mL Schlenk flask was added 50 mL DCM at room temperature under stirring in dark conditions. The color of the mixture from red changed to yellow slowly. After stirring 30 min, the yellow mixture was filtered and the residue was washed with DCM (5 mL x 3). The all volatiles were removed under vacuum from the combined filtrate to give yellow powder **7** (600 mg, 80% isolated yield). Yellow crystals suitable for X-ray diffraction analysis were obtained from a toluene/DCM solution at -30°C.

M.p. 186-188 °C (decomp.).

$^1$H NMR (500 MHz, DCM-$d_2$) $\delta$/ppm = 8.34 (dd, $J$ = 13.7, 7.7 Hz, 4H), 8.13 (dd, $J$ = 13.1, 7.8 Hz, 4H), 8.03 (d, $J$ = 7.5 Hz, 2H), 7.99 (t, $J$ = 7.4 Hz, 2H), 7.90 – 7.79 (m, 12H), 7.65 (t, $J$ = 7.6 Hz, 2H), 1.71 (s, 6H), 1.23 (s, 36H).

$^{13}$C{$^1$H} NMR (126 MHz, DCM-$d_2$) $\delta$/ppm = 153.48 (s, Ar-*C*), 139.93 (s, Ar-*C*), 136.04 (dd, $J$ = 6.6, 2.7 Hz, Ar-*C*), 135.21 (d, $J$ = 13.0 Hz, Ar-*C*), 134.93 (d, $J$ = 11.8 Hz, Ar-*C*), 133.85 (s, Ar-*C*), 133.66 (s, Ar-*C*), 130.48 (s, Ar-*C*), 130.42 (d, $J$ = 13.9 Hz, Ar-*C*), 130.14 (d, $J$ = 12.9 Hz, Ar-*C*), 129.52 (d, $J$ = 100.3 Hz, Ar-*C*), 127.25 (s, Ar-*C*), 125.53 (d, $J$ = 96.9 Hz, Ar-*C*), 120.98 (q, $J_{C\text{-}F}$ = 320.5 Hz), 56.11(s, *C*($CH_3$)$_2$), 36.28 (s, *C*($CH_3$)$_3$), 33.64 (d, $J$ = 4.9 Hz, C($CH_3$)$_3$), 30.95 (br, C(*C*$H_3$)$_2$).

$^{31}$P{$^1$H} NMR (202 MHz, DCM-$d_2$) $\delta$/ppm = 58.96.

$^{19}$F{$^1$H} NMR (471 MHz, DCM-$d_2$) $\delta$/ppm = -76.88, -78.60.

Elemental analysis calcd for $C_{58}H_{68}BiF_9Ge_2N_4O_{10}P_2S_3$: C, 41.85; N, 3.36; H, 4.18. Found: C, 41.05; N, 2.98; H, 3.75.

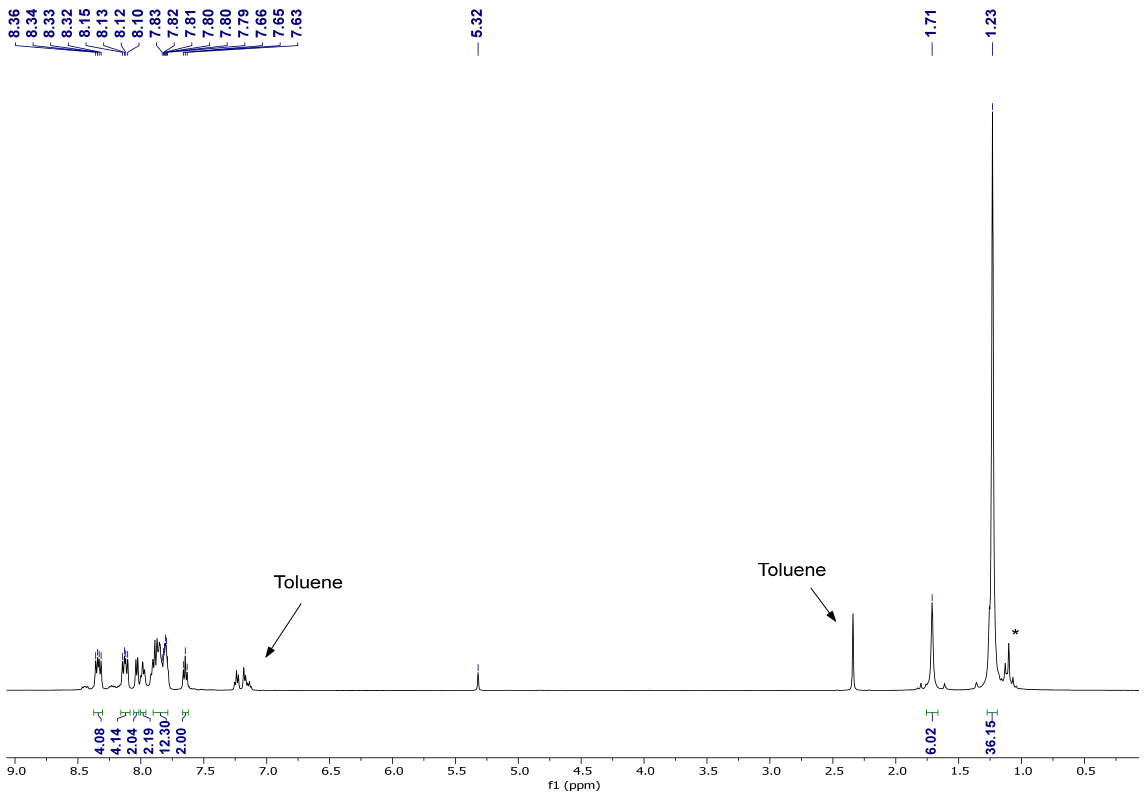


**Figure S42.** $^{1}$H NMR spectrum of **7** in DCM-$d_2$. *Unidentified impurities.

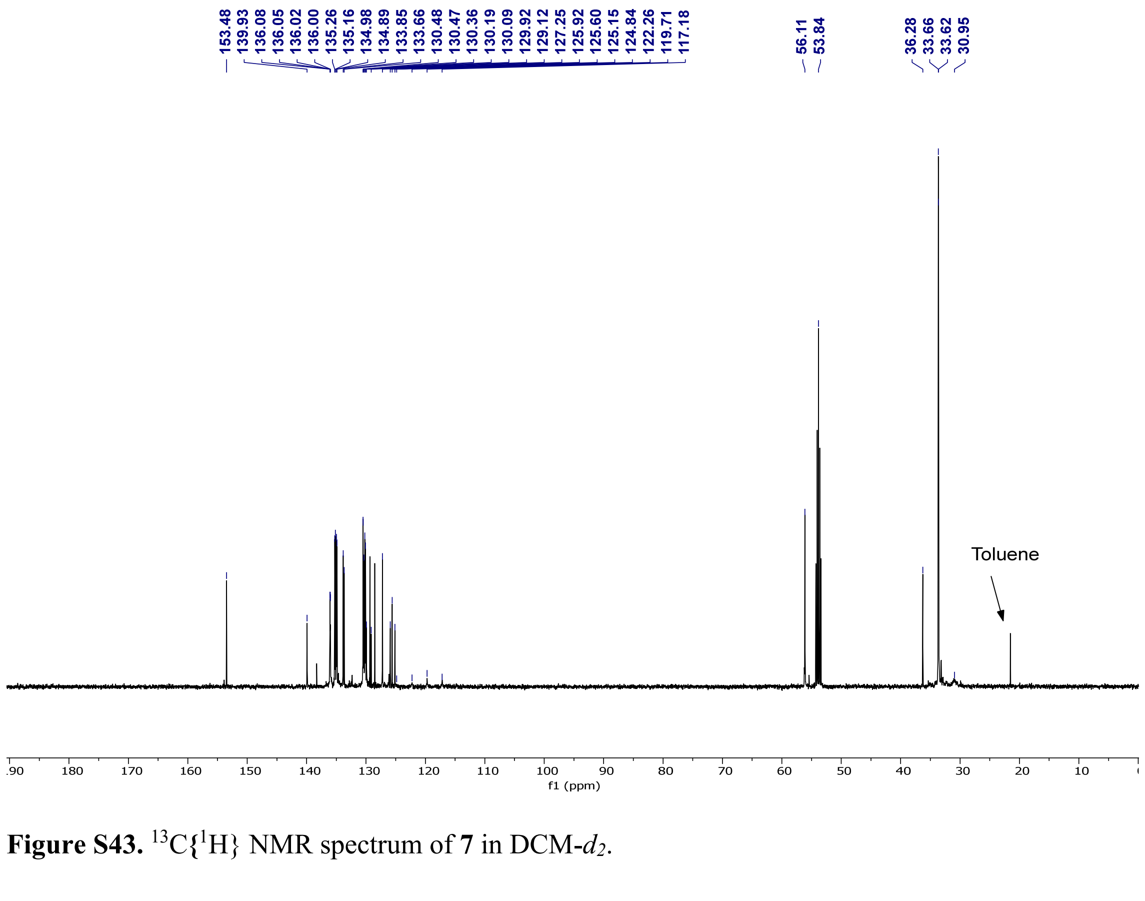


**Figure S43.** $^{13}$C{$^{1}$H} NMR spectrum of **7** in DCM-$d_2$.

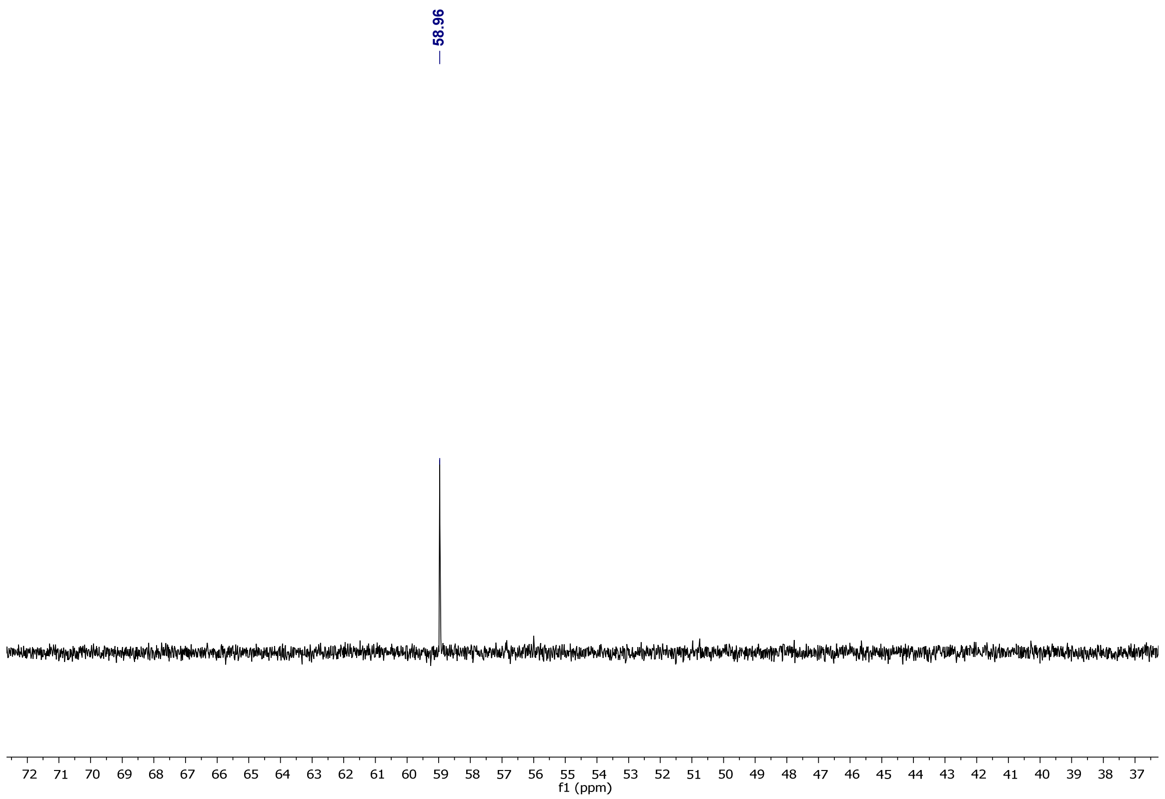


**Figure S44.** $^{31}P\{^{1}H\}$ NMR spectrum of **7** in DCM-$d_2$.

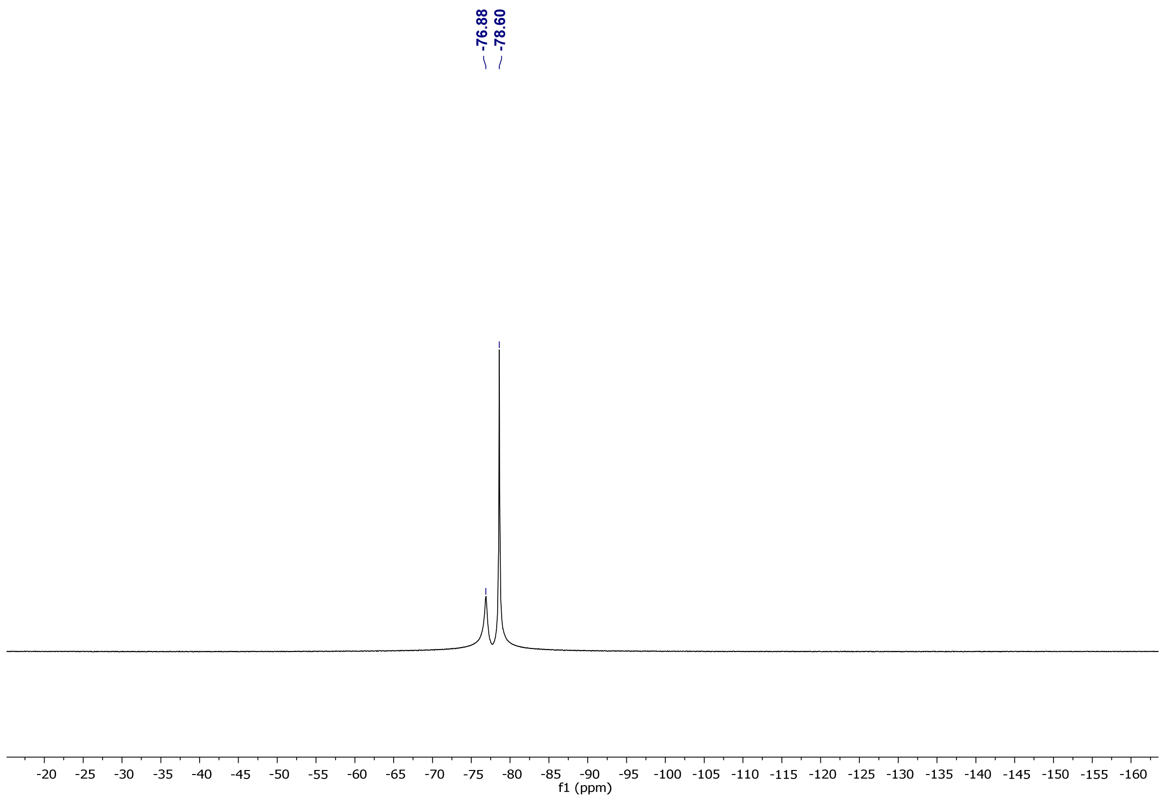


**Figure S45.** $^{19}F$ spectrum of **7** in DCM-$d_2$.

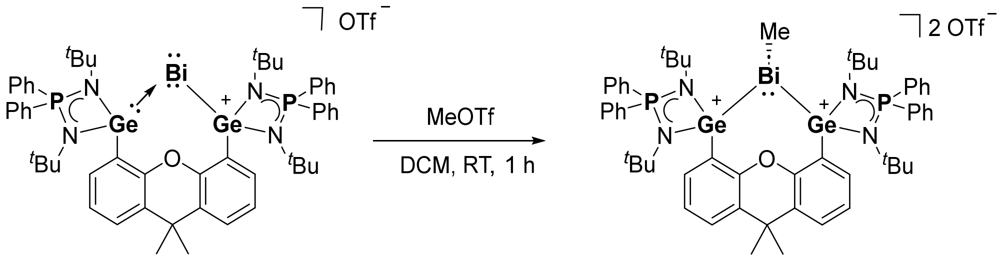


**Synthesis of compound 8.** To a mixture of compound **4**[OTf] (685 mg, 0.5 mmol) and 60 mL DCM in a 100 mL Schlenk flask, then 1.5 eq MeOTf (80 umL,0.75 mmol) was added at room temperature under stirring. The color of the mixture changed to orange immediately. After stirring 1 h, the color of the mixture changed to light yellow, then all volatiles were removed and dried under vacuum to afford compound **8** as a light-yellow powder. Recrystallization from DME formed compound **8** as light-yellow crystals (498 mg, 65% isolated yield). Light-yellow block crystals suitable for X-ray diffraction analysis were obtained from a concentrated DME solution at room temperature.

M.p. 177-181 °C (decomp.).

$^{1}$H NMR (200 MHz, DCM-*d*$_2$) $\delta$/ppm = 8.24 – 8.11 (m, 8H, Ar-*H*), 8.02 (m, 2H, Ar-*H*), 7.93 – 7.78 (m, 15H, Ar-*H*), 7.54 (t, *J* = 7.6 Hz, 2H, Ar-*H*), 2.43 (s, 3H, C*H*$_3$), 1.74 (s, 3H, C(C*H*$_3$)$_2$), 1.66 (s, 3H, C(C*H*$_3$)$_2$), 1.09 (s, 18H, C(C*H*$_3$)$_3$), 1.04 (s,18H, C(C*H*$_3$)$_3$).

$^{13}$C{$^{1}$H} NMR (50 MHz, DCM -*d*$_2$) $\delta$/ppm = 154.70 (s, Ar-*C*), 135.61 (d, *J* = 3.1 Hz, Ar-*C*), 135.40 (d, *J* = 3.1 Hz Ar-*C*), 134.09 (d, *J* = 12.2 Hz Ar-*C*), 133.74 (d, *J* = 12.2 Hz Ar-*C*), 132.16 (s, Ar-*C*), 131.38 (s, Ar-*C*), 130.43 (d, *J* = 7.2 Hz Ar-*C*), 130.06 (d, *J* = 4.2 Hz, Ar-*C*), 129.76, (s, Ar-*C*), 127.97 (d, *J* = 33.5 Hz, Ar-*C*), 126.58 (s, Ar-*C*), 126.03 (d, *J* = 34.0 Hz, Ar-*C*), 125.4 (s, Ar-*C*), 120.97 (q, $J_{c\text{-}F}$ = 321.2 Hz, *C*F$_3$), 55.56 (s, C(*C*H$_3$)$_2$), 54.89 (s, C(*CH*$_3$)$_2$), 36.02 (s, *C*(CH$_3$)$_2$), 32.72 (t, *J* = 5.7 Hz, C(*C*H$_3$)$_3$), 31.69 (s, *C*(CH$_3$)$_3$), 30.10 (s, *C*(CH$_3$)$_3$).

$^{31}$P{$^{1}$H} NMR (81 MHz, DCM-*d*$_2$) $\delta$/ppm = 53.44.

$^{19}$F{$^{1}$H} NMR (188 MHz, DCM-*d*$_2$) $\delta$/ppm = -78.58.

HR-MS(ESI): (m/z) calcd for ½ $[M\text{-}2OTf]^{2+}$ ½ $(C_{55}H_{68}Ge_2N_4P_2Bi)^{2+}$: 616.1667; found: 616.1656.

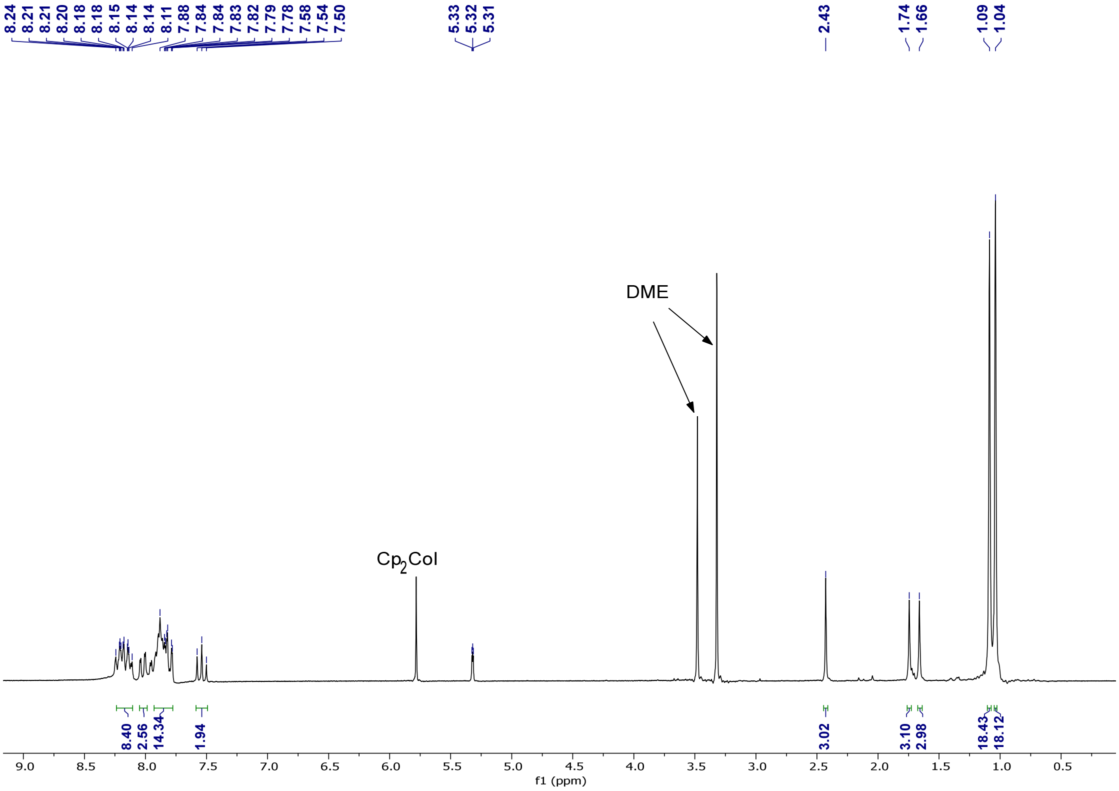


**Figure S46.** $^{1}H$ NMR spectrum of **8** in DCM-$d_2$.

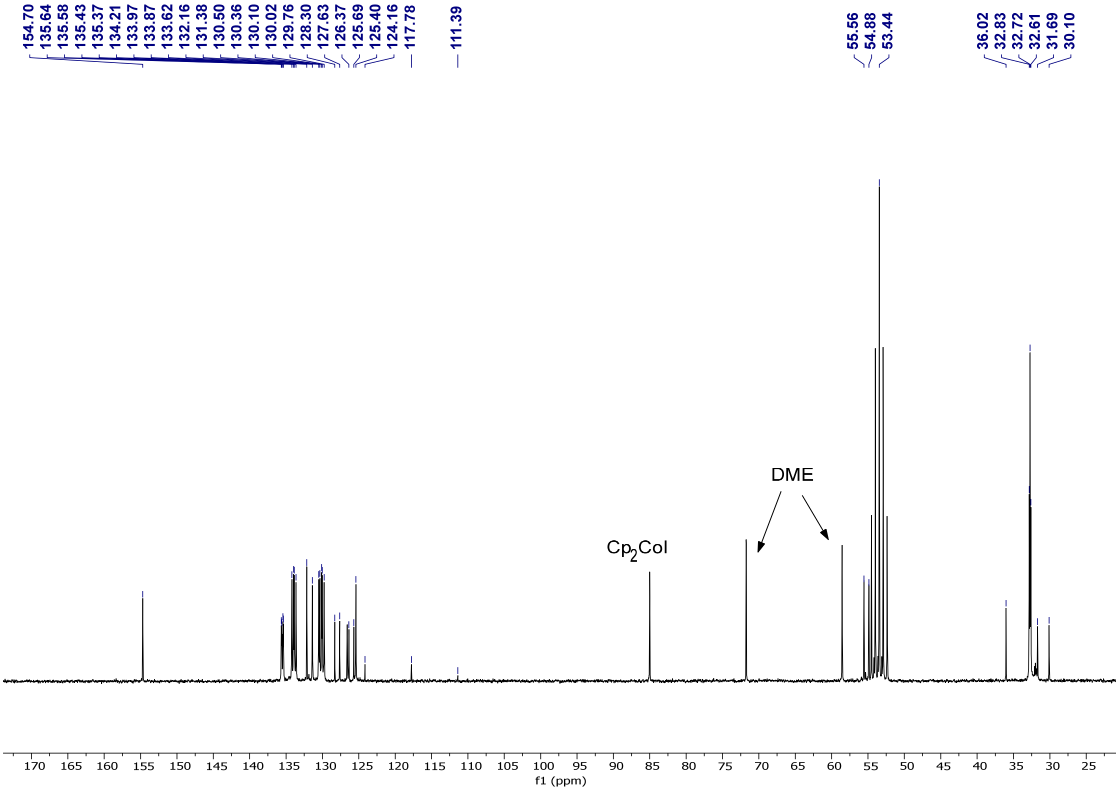


**Figure S47.** $^{13}C\{^{1}H\}$ NMR spectrum of **8** in DCM-$d_2$.

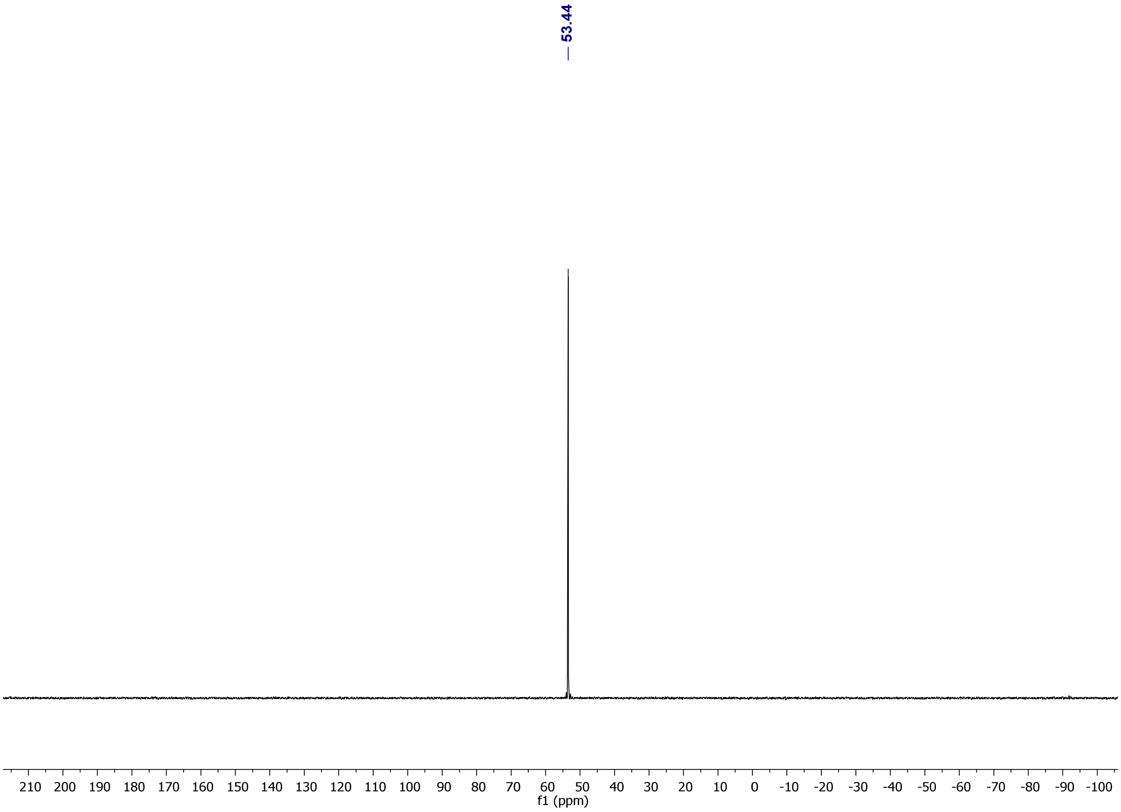


**Figure S48.** $^{31}P\{^{1}H\}$ NMR spectrum of **8** in DCM-*d*$_2$.

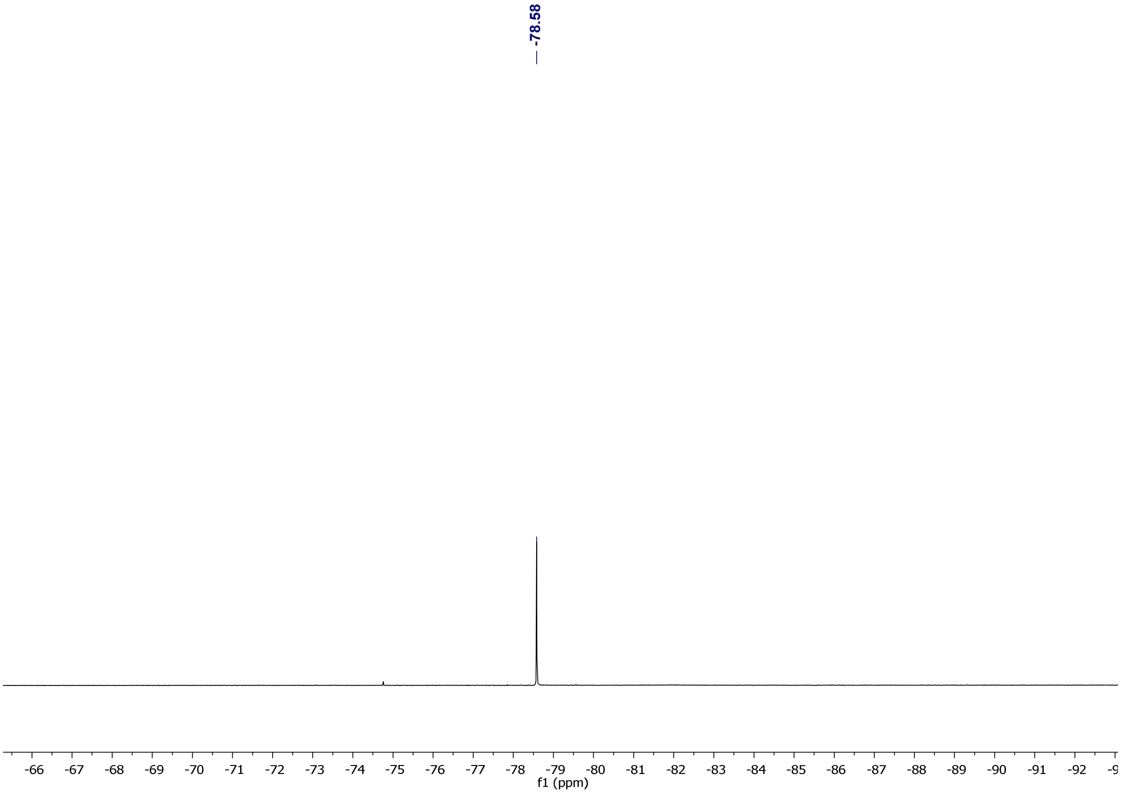


**Figure S49.** $^{19}F\{^{1}H\}$ spectrum of **8** in DCM-*d*$_2$.

## C. X-ray Crystallographic Data

**Table 1.** Crystal data and structure refinement for **(P)GeCl.**

| | | |
|---|---|---|
| Empirical formula | C20 H28 Cl Ge N2 P | |
| Formula weight | 435.45 | |
| Temperature | 150.00(10) K | |
| Wavelength | 1.54184 Å | |
| Crystal system | Monoclinic | |
| Space group | P 1 21/n 1 | |
| Unit cell dimensions | a = 11.0634(2) Å | a= 90°. |
| | b = 13.3590(2) Å | b= 92.486(2)°. |
| | c = 14.4693(3) Å | g = 90°. |
| Volume | 2136.49(7) $Å^3$ | |
| Z | 4 | |
| Density (calculated) | 1.354 $Mg/m^3$ | |
| Absorption coefficient | 3.833 $mm^{-1}$ | |
| F(000) | 904 | |
| Crystal size | 0.09 x 0.06 x 0.05 $mm^3$ | |
| Theta range for data collection | 4.507 to 72.381°. | |
| Index ranges | -13<=h<=13, -16<=k<=16, -12<=l<=17 | |
| Reflections collected | 8470 | |
| Independent reflections | 4120 [R(int) = 0.0152] | |
| Completeness to theta = 67.684° | 99.9 % | |
| Absorption correction | Semi-empirical from equivalents | |
| Max. and min. transmission | 1.00000 and 0.52871 | |
| Refinement method | Full-matrix least-squares on $F^2$ | |
| Data / restraints / parameters | 4120 / 0 / 232 | |
| Goodness-of-fit on $F^2$ | 1.079 | |
| Final R indices [I>2sigma(I)] | R1 = 0.0251, wR2 = 0.0669 | |
| R indices (all data) | R1 = 0.0261, wR2 = 0.0676 | |
| Extinction coefficient | n/a | |
| Largest diff. peak and hole | 0.342 and -0.602 e.$Å^{-3}$ | |

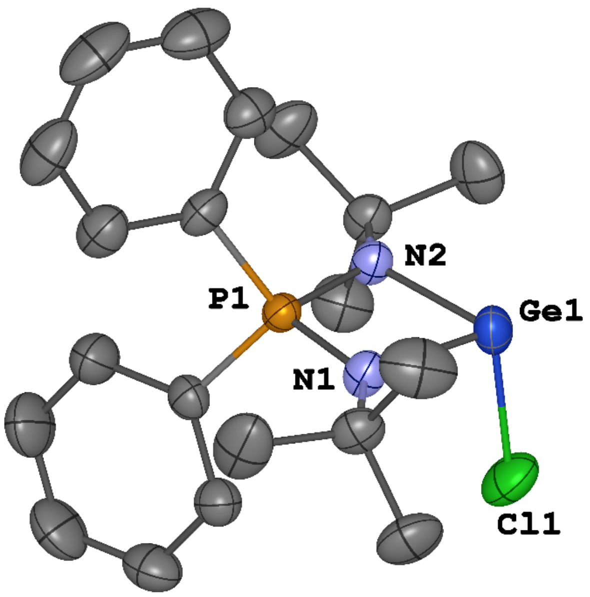


**Figure S50.** Molecular structure of iminophosphonamido-chlorogermylene **(P)GeCl**. Thermal ellipsoids are drawn at the 50% probability level. H atoms are omitted for clarity.

**Table S2.** Selected interatomic distances and angles of compound **(P)GeCl.**

| Bond lengths [Å] | | Angles [°] | |
|---|---|---|---|
| Ge(1)-Cl(1) | 2.3456(4) | N(1)-Ge(1)-P(1) | 36.77(4) |
| Ge(1)-N(1) | 1.9780(12) | N(1)-Ge(1)-Cl(1) | 98.27(4) |
| Ge(1)-N(2) | 1.9749(12) | N(2)-Ge(1)-Cl(1) | 98.27(4) |
| P(1)-C(9) | 1.8097(15) | N(1)-P(1)-N(2) | 94.27(6) |
| P(1)-C(15) | 1.8017(15) | | |

**Table S3.** Crystal data and structure refinement for **1.**

| | | |
|---|---|---|
| Empirical formula | C55 H68 Ge2 N4 O P2 | |
| Formula weight | 1008.25 | |
| Temperature | 150(10) K | |
| Wavelength | 1.54184 Å | |
| Crystal system | Monoclinic | |
| Space group | P 1 21/c 1 | |
| Unit cell dimensions | a = 17.2150(2) Å | a= 90°. |
| | b = 17.2724(2) Å | b= 114.656(2)°. |
| | c = 19.2182(3) Å | g = 90°. |
| Volume | 5193.43(14) $Å^3$ | |
| Z | 4 | |
| Density (calculated) | 1.290 $Mg/m^3$ | |
| Absorption coefficient | 2.320 $mm^{-1}$ | |
| F(000) | 2112 | |
| Crystal size | 0.05 x 0.04 x 0.02 $mm^3$ | |
| Theta range for data collection | 2.824 to 67.491°. | |
| Index ranges | -19<=h<=20, -20<=k<=20, -23<=l<=22 | |
| Reflections collected | 36151 | |
| Independent reflections | 9353 [R(int) = 0.0247] | |
| Completeness to theta = 67.492° | 99.9 % | |
| Absorption correction | Semi-empirical from equivalents | |
| Max. and min. transmission | 1.00000 and 0.29812 | |
| Refinement method | Full-matrix least-squares on $F^2$ | |
| Data / restraints / parameters | 9353 / 0 / 591 | |
| Goodness-of-fit on $F^2$ | 1.041 | |
| Final R indices [I>2sigma(I)] | R1 = 0.0259, wR2 = 0.0689 | |
| R indices (all data) | R1 = 0.0278, wR2 = 0.0705 | |
| Extinction coefficient | n/a | |
| Largest diff. peak and hole | 0.386 and -0.417 e.$Å^{-3}$ | |

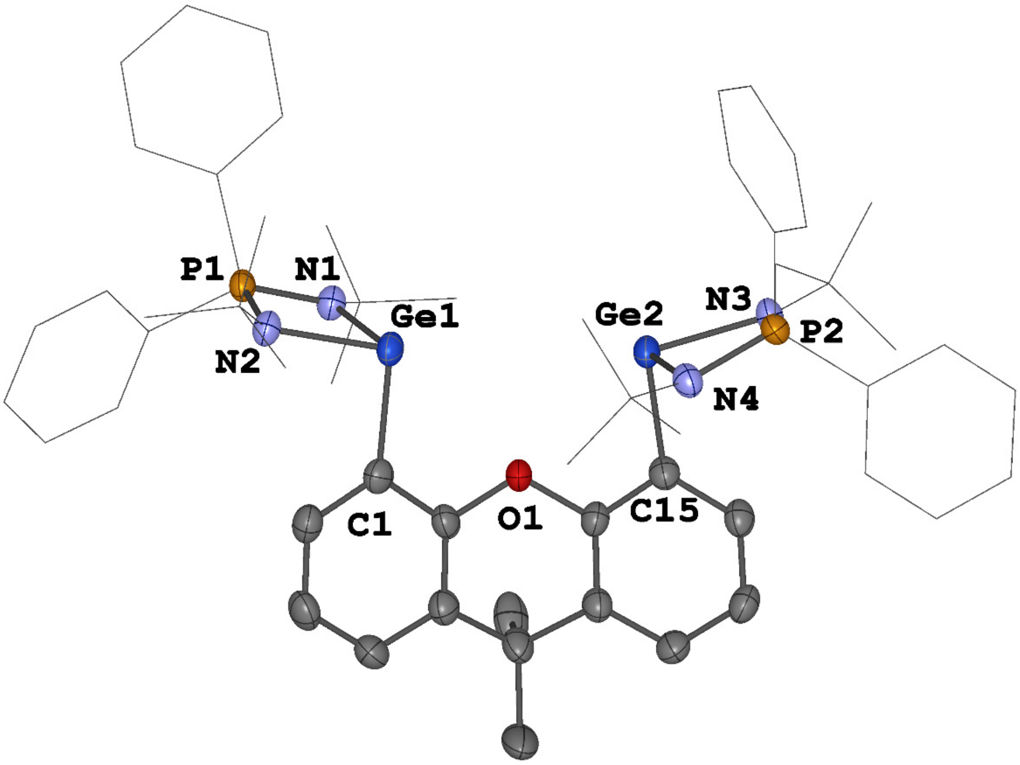


**Figure S51.** Molecular structure of compound **1**. Thermal ellipsoids are drawn at the 50% probability level. H atoms are omitted for clarity.

**Table S4.** Selected interatomic distances and angles of compound **1.**

| Bond lengths [Å] | | Angles [°] | |
|---|---|---|---|
| Ge(1)-C(1) | 2.0607(15) | N(1)-Ge(1)-P(1) | 35.93(3) |
| Ge(2)-C(15) | 2.0458(15) | N(2)-Ge(1)-P(1) | 36.42(3) |
| Ge(1)-N(1) | 2.0357(12) | N(1)-Ge(1)-C(1) | 99.11(5) |
| Ge(1)-N(2) | 2.0442(12) | N(2)-Ge(1)-C(1) | 96.42(5) |
| Ge(2)-N(3) | 2.0412(12) | N(3)-Ge(2)-P(2) | 35.96(4) |
| Ge(2)-N(4) | 2.0510(12) | N(4)-Ge(2)-P(2) | 36.12(3) |
| | | N(3)-Ge(2)-C(15) | 99.21(5) |
| | | N(4)-Ge(2)-C(15) | 92.06(5) |

**Table S5.** Crystal data and structure refinement for **2.**

| | | |
|---|---|---|
| Empirical formula | C63 H80 Bi Ge2 I3 N8 O P2 | |
| Formula weight | 1762.15 | |
| Temperature | 110.70(13) K | |
| Wavelength | 1.54184 Å | |
| Crystal system | Monoclinic | |
| Space group | P 1 21/n 1 | |
| Unit cell dimensions | a = 14.3685(2) Å | α= 90°. |
| | b = 22.6570(2) Å | β= 104.9760(10)°. |
| | c = 21.8782(2) Å | γ = 90°. |
| Volume | 6880.47(13) Å$^3$ | |
| Z | 4 | |
| Density (calculated) | 1.701 Mg/m$^3$ | |
| Absorption coefficient | 17.321 mm$^{-1}$ | |
| F(000) | 3432 | |
| Crystal size | 0.13 x 0.09 x 0.08 mm$^3$ | |
| Theta range for data collection | 2.859 to 67.494°. | |
| Index ranges | -17<=h<=15, -26<=k<=27, -17<=l<=26 | |
| Reflections collected | 48229 | |
| Independent reflections | 12406 [R(int) = 0.0317] | |
| Completeness to theta = 67.494° | 100.0 % | |
| Absorption correction | Semi-empirical from equivalents | |
| Max. and min. transmission | 1.00000 and 0.48001 | |
| Refinement method | Full-matrix least-squares on F$^2$ | |
| Data / restraints / parameters | 12406 / 0 / 647 | |
| Goodness-of-fit on F$^2$ | 1.033 | |
| Final R indices [I>2sigma(I)] | R1 = 0.0409, wR2 = 0.1103 | |
| R indices (all data) | R1 = 0.0431, wR2 = 0.1127 | |
| Extinction coefficient | n/a | |
| Largest diff. peak and hole | 1.974 and -3.430 e.Å$^{-3}$ | |

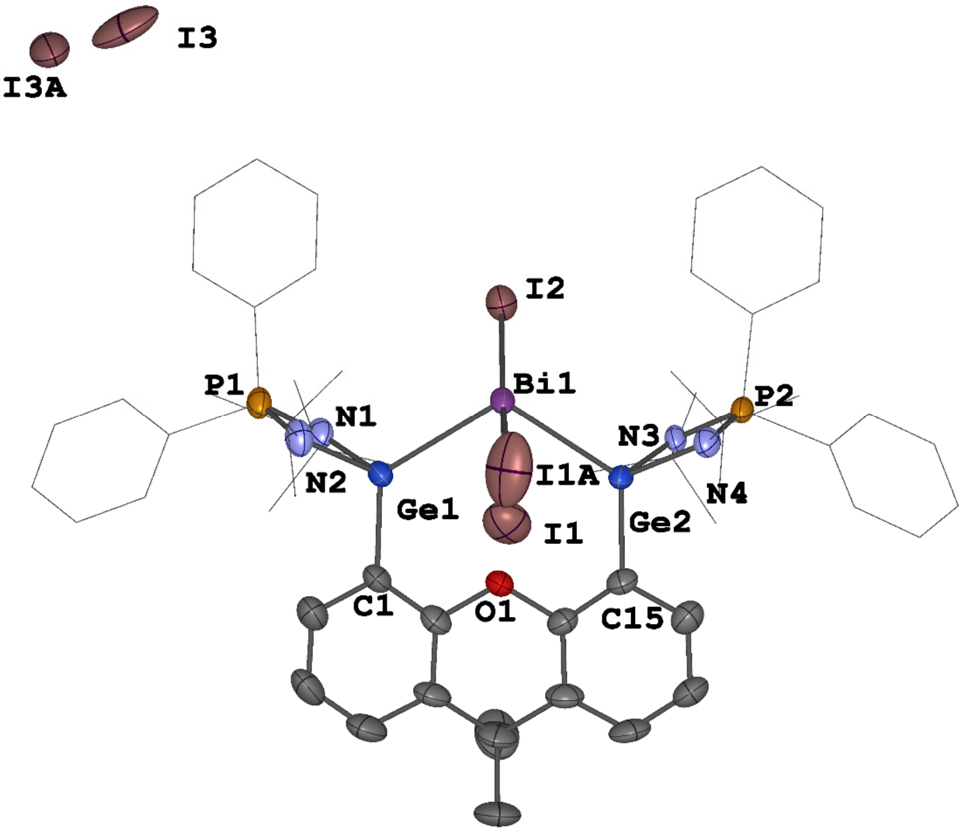


**Figure S52.** Molecular structure of compound **2**·[4 MeCN]. Thermal ellipsoids are drawn at 50% probability level. H atoms and solvent MeCN are omitted for clarity.

**Table S6.** Selected interatomic distances and angles of compound **2**.

| Bond lengths [Å] | | Angles [°] | |
|---|---|---|---|
| Bi(1)-Ge(1) | 2.7578(6) | I(1)-Bi(1)-I(2) | 169.779(15) |
| Bi(1)-Ge(2) | 2.7497(6) | Ge(1)-Bi(1)-Ge(2) | 109.244(18) |
| Bi(1)-I(1) | 3.0921(5) | Ge(1)-Bi(1)-I(1) | 80.318(16) |
| Bi(1)-I(2) | 2.9991(4) | Ge(1)-Bi(1)-I(2) | 95.147(15) |
| Ge(1)-C(1) | 1.953(5) | Ge(2)-Bi(1)-I(1) | 77.501(16) |
| Ge(2)-C(15) | 1.943(5) | Ge(2)-Bi(1)-I(2) | 95.574(15) |

**Table S7.** Crystal data and structure refinement for **3**[BAr$^F$].

| | | |
|---|---|---|
| Empirical formula | C88 H82 B Bi Cl2 F24 Ge2 I2 N4 O P2 | |
| Formula weight | 2419.18 | |
| Temperature | 150.00(10) K | |
| Wavelength | 1.54184 Å | |
| Crystal system | Monoclinic | |
| Space group | P 1 21/c 1 | |
| Unit cell dimensions | a = 20.6819(3) Å | a= 90°. |
| | b = 25.1786(3) Å | b= 111.093(2)°. |
| | c = 19.0877(3) Å | g = 90°. |
| Volume | 9273.8(3) Å$^3$ | |
| Z | 4 | |
| Density (calculated) | 1.733 Mg/m$^3$ | |
| Absorption coefficient | 11.356 mm$^{-1}$ | |
| F(000) | 4736 | |
| Crystal size | 0.06 x 0.04 x 0.03 mm$^3$ | |
| Theta range for data collection | 2.885 to 67.498°. | |
| Index ranges | -24<=h<=21, -28<=k<=30, -15<=l<=22 | |
| Reflections collected | 36510 | |
| Independent reflections | 16640 [R(int) = 0.0340] | |
| Completeness to theta = 67.498° | 99.6 % | |
| Absorption correction | Semi-empirical from equivalents | |
| Max. and min. transmission | 1.00000 and 0.44259 | |
| Refinement method | Full-matrix least-squares on F$^2$ | |
| Data / restraints / parameters | 16640 / 0 / 1158 | |
| Goodness-of-fit on F$^2$ | 1.016 | |
| Final R indices [I>2sigma(I)] | R1 = 0.0331, wR2 = 0.0762 | |
| R indices (all data) | R1 = 0.0438, wR2 = 0.0825 | |
| Extinction coefficient | n/a | |
| Largest diff. peak and hole | 1.346 and -1.161 e.Å$^{-3}$ | |

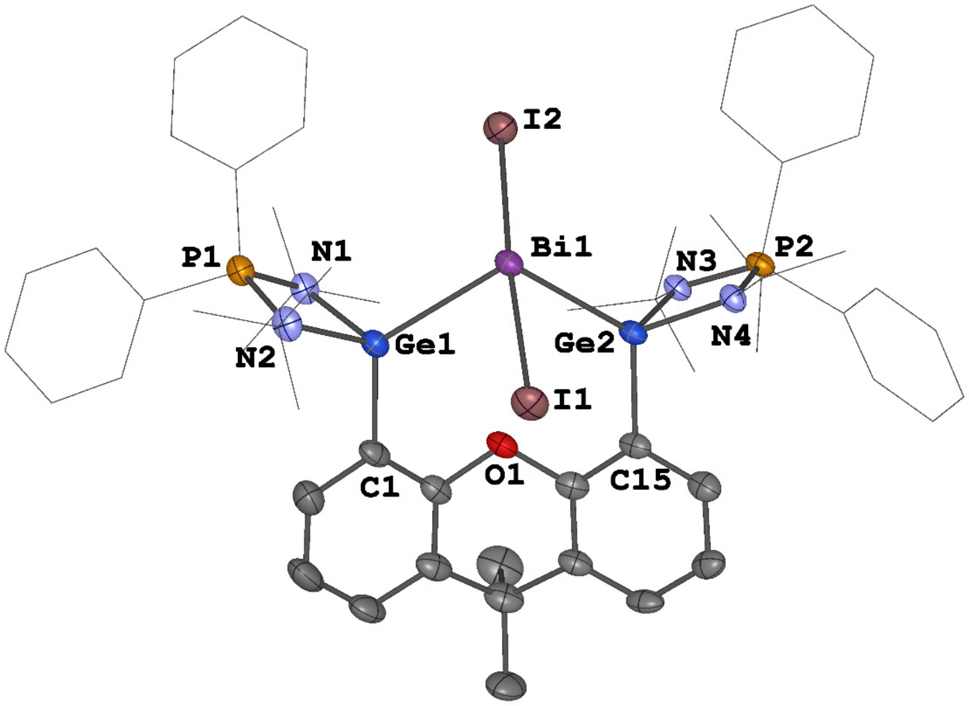


**Figure S53.** Molecular structure of the cation in **3**[BAr$^F$]. Thermal ellipsoids are drawn at 50% probability level. H atoms and inion BAr$^F$ are omitted for clarity.

**Table S8.** Selected interatomic distances and angles of compound **3**[BAr$^F$].

| Bond lengths [Å] | | Angles [°] | |
|---|---|---|---|
| Bi(1)-Ge(1) | 2.7737(5) | I(1)-Bi(1)-I(2) | 172.326(10) |
| Bi(1)-Ge(2) | 2.7800(5) | Ge(1)-Bi(1)-Ge(2) | 103.895(14) |
| Bi(1)-I(1) | 3.0406(3) | Ge(1)-Bi(1)-I(1) | 97.781(12) |
| Bi(1)-I(2) | 3.0424(3) | Ge(1)-Bi(1)-I(2) | 84.876(12) |
| Ge(1)-C(1) | 1.959(4) | Ge(2)-Bi(1)-I(1) | 100.119(12) |
| Ge(2)-C(15) | 1.948(4) | Ge(2)-Bi(1)-I(2) | 86.151(12) |

**Table S9.** Crystal data and structure refinement for **3**[OTf].

| | | |
|---|---|---|
| Empirical formula | C65 H75.50 Bi F4.50 Ge2 I2 N4 O4 P2 S | |
| Formula weight | 1764.25 | |
| Temperature | 111(1) K | |
| Wavelength | 1.54184 Å | |
| Crystal system | Orthorhombic | |
| Space group | Pbcn | |
| Unit cell dimensions | a = 41.1017(4) Å | a= 90°. |
| | b = 16.9469(2) Å | b= 90°. |
| | c = 20.0416(3) Å | g = 90°. |
| Volume | 13959.9(3) $Å^3$ | |
| Z | 8 | |
| Density (calculated) | 1.679 $Mg/m^3$ | |
| Absorption coefficient | 14.032 $mm^{-1}$ | |
| F(000) | 6920 | |
| Crystal size | 0.4 x 0.07 x 0.06 $mm^3$ | |
| Theta range for data collection | 2.820 to 72.579°. | |
| Index ranges | -50<=h<=47, -20<=k<=20, -24<=l<=24 | |
| Reflections collected | 101539 | |
| Independent reflections | 13736 [R(int) = 0.0506] | |
| Completeness to theta = 67.684° | 100.0 % | |
| Absorption correction | Semi-empirical from equivalents | |
| Max. and min. transmission | 1.00000 and 0.01766 | |
| Refinement method | Full-matrix least-squares on $F^2$ | |
| Data / restraints / parameters | 13736 / 0 / 786 | |
| Goodness-of-fit on $F^2$ | 1.116 | |
| Final R indices [I>2sigma(I)] | R1 = 0.0371, wR2 = 0.0856 | |
| R indices (all data) | R1 = 0.0434, wR2 = 0.0902 | |
| Extinction coefficient | n/a | |
| Largest diff. peak and hole | 1.009 and -1.327 e.$Å^{-3}$ | |

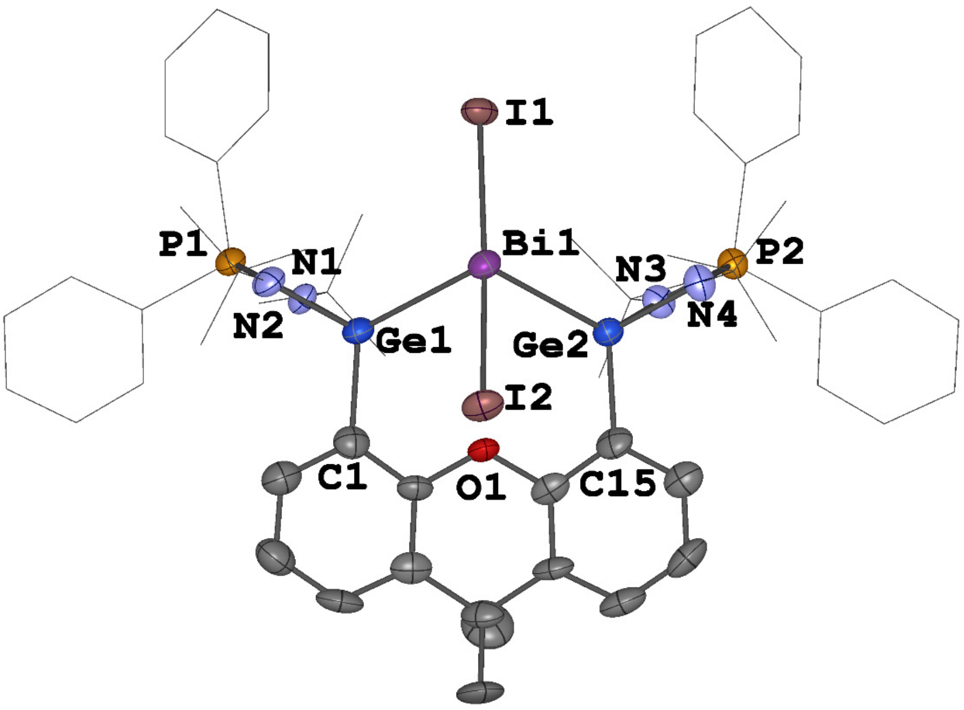


**Figure S54.** Molecular structure of the cation in **3**[OTf] [1.5·$C_6H_5F$]. Thermal ellipsoids are drawn at 50% probability level. H atoms, inion OTf and solvent $C_6H_5F$ are omitted for clarity.

**Table S10.** Selected interatomic distances and angles of compound **3**[OTf].

| Bond lengths [Å] | | Angles [°] | |
|---|---|---|---|
| Bi(1)-Ge(1) | 2.8035(5) | I(1)-Bi(1)-I(2) | 174.507(10) |
| Bi(1)-Ge(2) | 2.7813(5) | Ge(1)-Bi(1)-Ge(2) | 106.995(16) |
| Bi(1)-I(1) | 3.0404(3) | Ge(1)-Bi(1)-I(1) | 82.333(13) |
| Bi(1)-I(2) | 3.0679(3) | Ge(1)-Bi(1)-I(2) | 99.018(13) |
| Ge(1)-C(1) | 1.952(5) | Ge(2)-Bi(1)-I(1) | 81.305(13) |
| Ge(2)-C(15) | 1.957(4) | Ge(2)-Bi(1)-I(2) | 93.215(13) |

**Table S11.** Crystal data and structure refinement for **4**[BAr$^F$].

| | | |
|---|---|---|
| Empirical formula | C89 H84 B Bi Cl4 F24 Ge2 N4 O P2 | |
| Formula weight | 2250.31 | |
| Temperature | 110.48(10) K | |
| Wavelength | 1.54184 Å | |
| Crystal system | Triclinic | |
| Space group | P-1 | |
| Unit cell dimensions | a = 16.4898(6) Å | a= 76.595(2)°. |
| | b = 17.6446(5) Å | b= 66.411(3)°. |
| | c = 18.6985(5) Å | g = 78.098(2)°. |
| Volume | 4810.6(3) Å$^3$ | |
| Z | 2 | |
| Density (calculated) | 1.554 Mg/m$^3$ | |
| Absorption coefficient | 6.421 mm$^{-1}$ | |
| F(000) | 2240 | |
| Crystal size | 0.05 x 0.03 x 0.02 mm$^3$ | |
| Theta range for data collection | 2.595 to 67.500°. | |
| Index ranges | -19<=h<=19, -16<=k<=21, -22<=l<=22 | |
| Reflections collected | 35058 | |
| Independent reflections | 17315 [R(int) = 0.0441] | |
| Completeness to theta = 67.500° | 99.9 % | |
| Absorption correction | Semi-empirical from equivalents | |
| Max. and min. transmission | 1.00000 and 0.54800 | |
| Refinement method | Full-matrix least-squares on F$^2$ | |
| Data / restraints / parameters | 17315 / 6 / 1141 | |
| Goodness-of-fit on F$^2$ | 1.022 | |
| Final R indices [I>2sigma(I)] | R1 = 0.0394, wR2 = 0.1056 | |
| R indices (all data) | R1 = 0.0405, wR2 = 0.1069 | |
| Extinction coefficient | n/a | |
| Largest diff. peak and hole | 1.863 and -2.369 e.Å$^{-3}$ | |

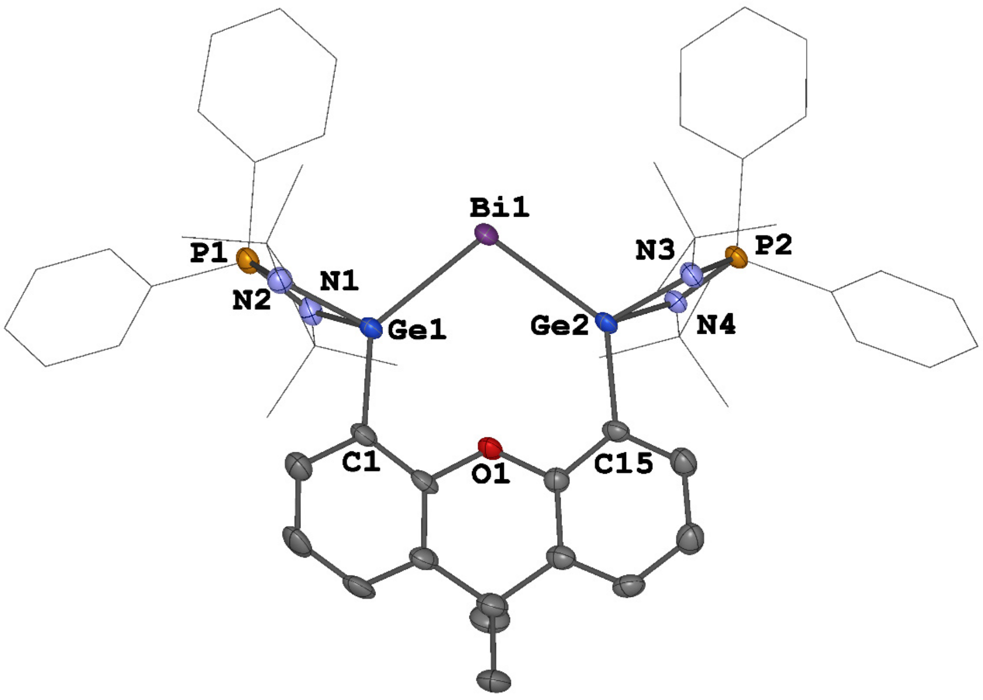


**Figure S55.** Molecular structure of the cation in **4**[BAr$^F$] [2·DCM]. Thermal ellipsoids are drawn at 50% probability level. H atoms, inion BAr$^F$ and solvent DCM molecules are omitted for clarity.

**Table S12.** Selected interatomic distances and angles of compound **4**[BAr$^F$].

| Bond lengths [Å] | | Angles [°] | |
|---|---|---|---|
| Bi(1)-Ge(1) | 2.6672(4) | Ge(2)-Bi(1)-Ge(1) | 103.981(12) |
| Bi(1)-Ge(2) | 2.6627(4) | C(1)-Ge(1)-Bi(1) | 129.16(10) |
| Ge(1)-C(1) | 1.959(3) | C(15)-Ge(2)-Bi(1) | 129.98(9) |
| Ge(2)-C(15) | 1.965(3) | | |

**Table S13.** Crystal data and structure refinement for **4**[OTf].

| | | |
|---|---|---|
| Empirical formula | C60 H76 Bi Cl8 F3 Ge2 N4 O4 P2 S | |
| Formula weight | 1706.00 | |
| Temperature | 110.9(3) K | |
| Wavelength | 1.54184 Å | |
| Crystal system | Orthorhombic | |
| Space group | Pbca | |
| Unit cell dimensions | a = 22.5976(4) Å | $\alpha$= 90°. |
| | b = 22.8569(4) Å | $\beta$= 90°. |
| | c = 28.8812(4) Å | $\gamma$ = 90°. |
| Volume | 14917.5(4) $Å^3$ | |
| Z | 8 | |
| Density (calculated) | 1.519 $Mg/m^3$ | |
| Absorption coefficient | 9.234 $mm^{-1}$ | |
| F(000) | 6816 | |
| Crystal size | 0.08 x 0.05 x 0.03 $mm^3$ | |
| Theta range for data collection | 3.060 to 68.249°. | |
| Index ranges | -18<=h<=27, -27<=k<=27, -34<=l<=34 | |
| Reflections collected | 64250 | |
| Independent reflections | 13637 [R(int) = 0.0520] | |
| Completeness to theta = 67.684° | 99.8 % | |
| Absorption correction | Semi-empirical from equivalents | |
| Max. and min. transmission | 1.00000 and 0.29985 | |
| Refinement method | Full-matrix least-squares on $F^2$ | |
| Data / restraints / parameters | 13637 / 0 / 676 | |
| Goodness-of-fit on $F^2$ | 1.077 | |
| Final R indices [I>2sigma(I)] | R1 = 0.0656, wR2 = 0.1929 | |
| R indices (all data) | R1 = 0.0830, wR2 = 0.2095 | |
| Extinction coefficient | n/a | |
| Largest diff. peak and hole | 1.736 and -1.104 e.$Å^{-3}$ | |

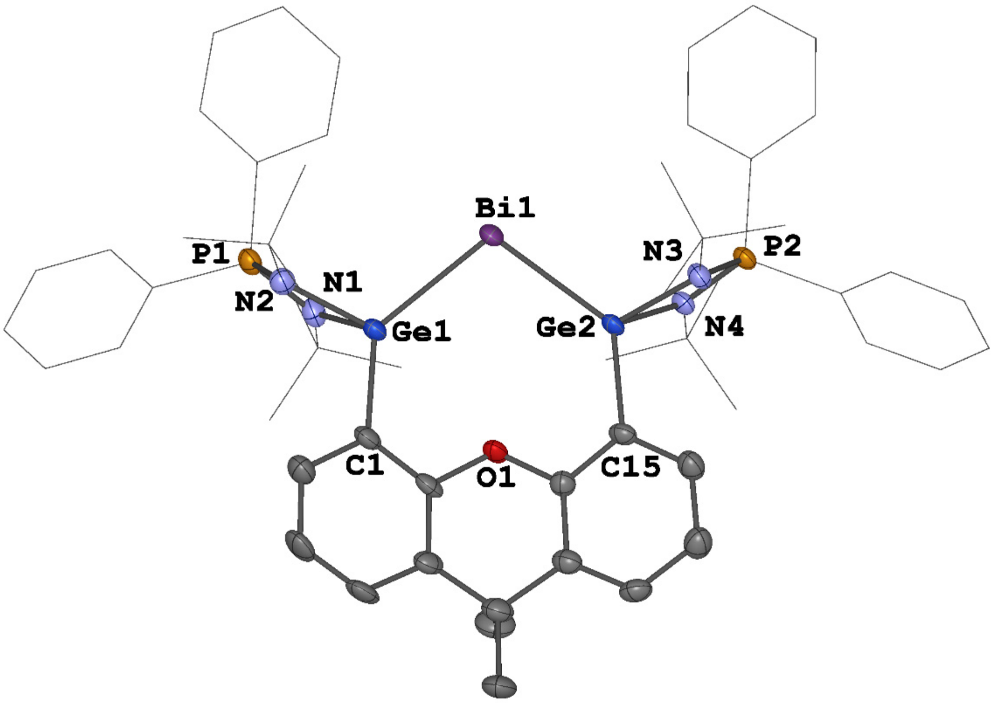


**Figure S56.** Molecular structure of the cation in **4**[OTf] [2·DCM]. Thermal ellipsoids are drawn at 50% probability level. H atoms, inion OTf and solvent DCM molecules are omitted for clarity.

**Table S14.** Selected interatomic distances and angles of compound **4**[OTf].

| Bond lengths [Å] | | Angles [°] | |
|---|---|---|---|
| Bi(1)-Ge(1) | 2.6693(9) | Ge(2)-Bi(1)-Ge(1) | 104.67(3) |
| Bi(1)-Ge(2) | 2.6712(9) | C(1)-Ge(1)-Bi(1) | 129.8(2) |
| Ge(1)-C(1) | 1.964(8) | C(15)-Ge(2)-Bi(1) | 129.9(2) |
| Ge(2)-C(15) | 1.980(8) | | |

**Table S15.** Crystal data and structure refinement for **5**[$BAr^F$]$_2$.

| | | |
|---|---|---|
| Empirical formula | C164 H129.50 B2 Bi F55.50 Ge2 N4 O P2 | |
| Formula weight | 3664.43 | |
| Temperature | 110.67 K | |
| Wavelength | 1.54184 Å | |
| Crystal system | Triclinic | |
| Space group | P-1 | |
| Unit cell dimensions | a = 16.3129(4) Å | a= 85.719(2)°. |
| | b = 18.1742(4) Å | b= 76.671(2)°. |
| | c = 28.0716(5) Å | g = 76.243(2)°. |
| Volume | 7864.3(3) Å$^3$ | |
| Z | 2 | |
| Density (calculated) | 1.547 Mg/m$^3$ | |
| Absorption coefficient | 3.865 mm$^{-1}$ | |
| F(000) | 3672 | |
| Crystal size | 0.15 x 0.13 x 0.11 mm$^3$ | |
| Theta range for data collection | 2.953 to 72.602°. | |
| Index ranges | -19<=h<=20, -21<=k<=22, -29<=l<=34 | |
| Reflections collected | 58646 | |
| Independent reflections | 30322 [R(int) = 0.0369] | |
| Completeness to theta = 67.684° | 99.8 % | |
| Absorption correction | Semi-empirical from equivalents | |
| Max. and min. transmission | 1.00000 and 0.09163 | |
| Refinement method | Full-matrix least-squares on F$^2$ | |
| Data / restraints / parameters | 30322 / 159 / 1682 | |
| Goodness-of-fit on F$^2$ | 1.030 | |
| Final R indices [I>2sigma(I)] | R1 = 0.0366, wR2 = 0.0939 | |
| R indices (all data) | R1 = 0.0429, wR2 = 0.0988 | |
| Extinction coefficient | n/a | |
| Largest diff. peak and hole | 1.005 and -0.958 e.Å$^{-3}$ | |

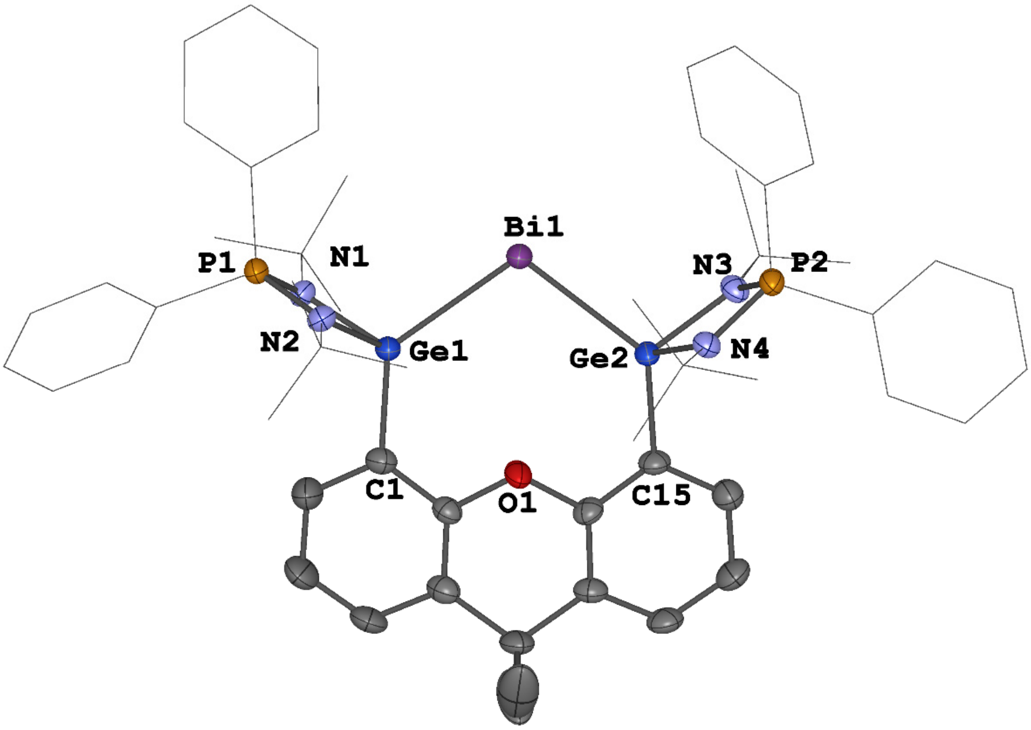


**Figure S57.** Molecular structure of compound **5**[BAr$^F$]$_2$ [7.5 $C_6H_5F$]. Thermal ellipsoids are drawn at 50% probability level. H atoms, two inions BAr$^F$ and solvent $C_6H_5F$ molecules are omitted for clarity.

**Table S16.** Selected interatomic distances and angles of compound **5**[BAr$^F$]$_2$.

| Bond lengths [Å] | | Angles [°] | |
|---|---|---|---|
| Bi(1)-Ge(1) | 2.7112(3) | Ge(1)-Bi(1)-Ge(2) | 107.583(9) |
| Bi(1)-Ge(2) | 2.7147(3) | C(1)-Ge(1)-Bi(1) | 127.99(8) |
| Ge(1)-C(1) | 1.941(3) | C(15)-Ge(2)-Bi(1) | 127.47(8) |
| Ge(2)-C(15) | 1.944(3) | N(2)-Ge(1)-N(1) | 77.05(9) |
| | | N(3)-Ge(2)-N(4) | 76.55(10) |

**Table S17**. Crystal data and structure refinement for **7**.

| | | |
|---|---|---|
| Empirical formula | C65 H76 Bi F9 Ge2 N4 O10 P2 S3 | |
| Formula weight | 1756.57 | |
| Temperature | 110.66(19) K | |
| Wavelength | 1.54184 Å | |
| Crystal system | Monoclinic | |
| Space group | P 1 21/c 1 | |
| Unit cell dimensions | a = 21.6309(3) Å | a= 90°. |
| | b = 14.8347(2) Å | b= 105.0970(10)°. |
| | c = 23.0182(3) Å | g = 90°. |
| Volume | 7131.33(17) $Å^3$ | |
| Z | 4 | |
| Density (calculated) | 1.636 $Mg/m^3$ | |
| Absorption coefficient | 7.710 $mm^{-1}$ | |
| F(000) | 3520 | |
| Crystal size | 0.04 x 0.01 x 0.01 $mm^3$ | |
| Theta range for data collection | 3.582 to 67.500°. | |
| Index ranges | -25<=h<=20, -17<=k<=17, -23<=l<=27 | |
| Reflections collected | 49080 | |
| Independent reflections | 12828 [R(int) = 0.0506] | |
| Completeness to theta = 67.500° | 99.9 % | |
| Absorption correction | Semi-empirical from equivalents | |
| Max. and min. transmission | 1.00000 and 0.26203 | |
| Refinement method | Full-matrix least-squares on $F^2$ | |
| Data / restraints / parameters | 12828 / 3 / 824 | |
| Goodness-of-fit on $F^2$ | 1.110 | |
| Final R indices [I>2sigma(I)] | R1 = 0.0536, wR2 = 0.1285 | |
| R indices (all data) | R1 = 0.0619, wR2 = 0.1355 | |
| Extinction coefficient | n/a | |
| Largest diff. peak and hole | 1.836 and -1.307 e.$Å^{-3}$ | |

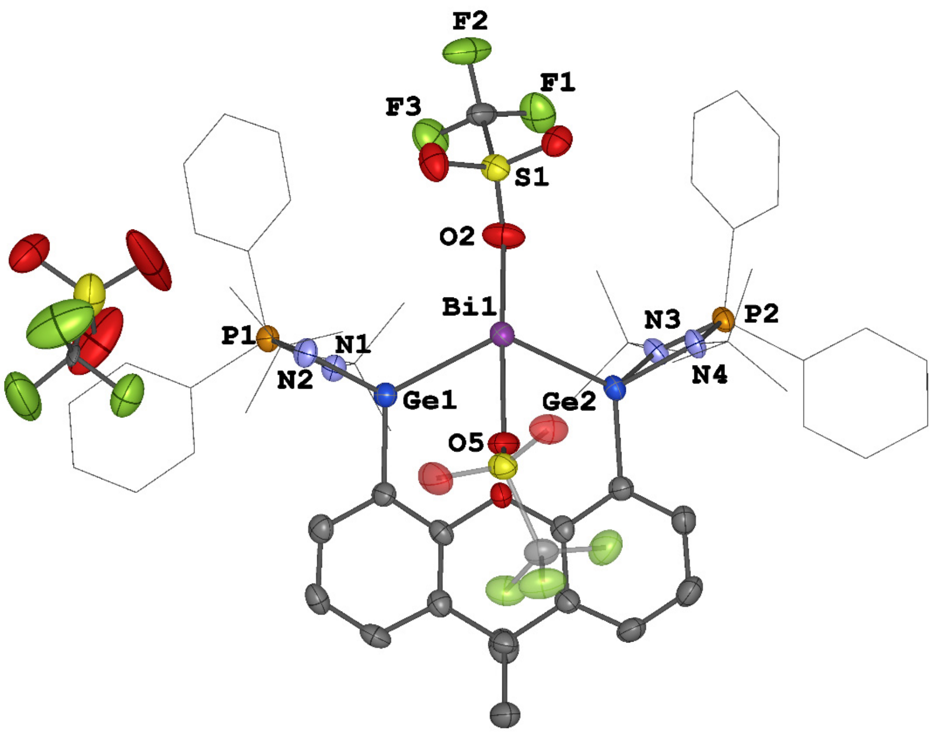


**Figure S58.** Molecular structure of compound **7** [$C_7H_8$]. Thermal ellipsoids are drawn at 50% probability level. H atoms and solvent $C_7H_8$ molecules are omitted for clarity.

**Table S18.** Selected interatomic distances and angles of compound **7**.

| Bond lengths [Å] | | Angles [°] | |
|---|---|---|---|
| Bi(1)-Ge(1) | 2.7796(8) | Ge(1)-Bi(1)-Ge(2) | 107.22(2) |
| Bi(1)-Ge(2) | 2.7670(8) | C(1)-Ge(1)-Bi(1) | 115.8(2) |
| Ge(1)-C(1) | 1.935(7) | C(15)-Ge(2)-Bi(1) | 120.00(19) |
| Ge(2)-C(15) | 1.962(7) | C(16)-Bi(1)-Ge(1) | 115.3(2) |
| Bi(1)-O(2) | 2.410(5) | O(5)-Bi(1)-O(2) | 169.85(18) |
| Bi(1)-O(5) | 2.397(5) | | |

**Table S19.** Crystal data and structure refinement for **8**.

| | | |
|---|---|---|
| Empirical formula | C62 H81 Bi F6 Ge2 N4 O9 P2 S2 | |
| Formula weight | 1620.52 | |
| Temperature | 110.0 K | |
| Wavelength | 1.54184 Å | |
| Crystal system | Orthorhombic | |
| Space group | $P2_12_12_1$ | |
| Unit cell dimensions | a = 11.43200(10) Å | a= 90°. |
| | b = 23.7107(3) Å | b= 90°. |
| | c = 25.1189(3) Å | g = 90°. |
| Volume | 6808.75(13) Å$^3$ | |
| Z | 4 | |
| Density (calculated) | 1.581 Mg/m$^3$ | |
| Absorption coefficient | 7.662 mm$^{-1}$ | |
| F(000) | 3264 | |
| Crystal size | 0.32 x 0.07 x 0.05 mm$^3$ | |
| Theta range for data collection | 2.563 to 72.506°. | |
| Index ranges | -13<=h<=9, -28<=k<=29, -30<=l<=31 | |
| Reflections collected | 49129 | |
| Independent reflections | 13271 [R(int) = 0.0454] | |
| Completeness to theta = 67.684° | 100.0 % | |
| Absorption correction | Semi-empirical from equivalents | |
| Max. and min. transmission | 1.00000 and 0.01768 | |
| Refinement method | Full-matrix least-squares on F$^2$ | |
| Data / restraints / parameters | 13271 / 0 / 811 | |
| Goodness-of-fit on F$^2$ | 1.059 | |
| Final R indices [I>2sigma(I)] | R1 = 0.0312, wR2 = 0.0756 | |
| R indices (all data) | R1 = 0.0348, wR2 = 0.0787 | |
| Absolute structure parameter | 0.492(5) | |
| Extinction coefficient | n/a | |
| Largest diff. peak and hole | 0.995 and -0.845 e.Å$^{-3}$ | |

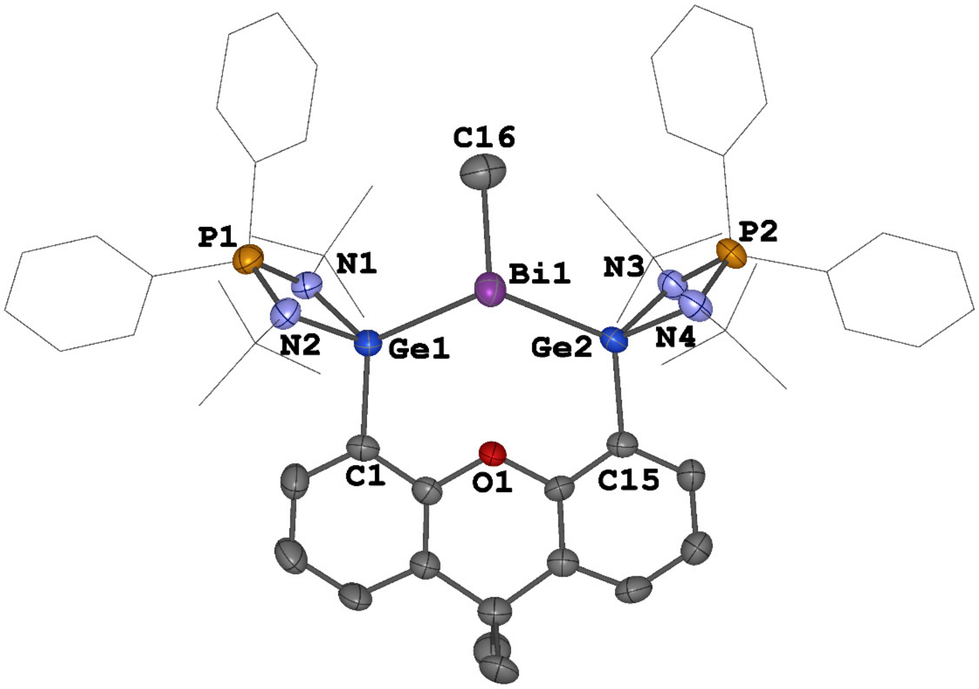


Figure **S59**. Molecular structure of compound **8**[DME]. Thermal ellipsoids are drawn at 50% probability level. H atoms, two inions OTf and solvent DME molecules are omitted for clarity.

**Table S20.** Selected interatomic distances and angles of compound **8**.

| Bond lengths [Å] | | Angles [°] | |
|---|---|---|---|
| Bi(1)-Ge(1) | 2.7393(7) | Ge(1)-Bi(1)-Ge(2) | 108.09(2) |
| Bi(1)-Ge(2) | 2.7426(7) | C(1)-Ge(1)-Bi(1) | 119.82(19) |
| Ge(1)-C(1) | 1.960(6) | C(15)-Ge(2)-Bi(1) | 120.00(19) |
| Ge(2)-C(15) | 1.952(6) | C(16)-Bi(1)-Ge(1) | 97.6(2) |
| Bi(1)-C(16) | 2.247(7) | C(16)-Bi(1)-Ge(2) | 101.8(2) |

# D. Magnetic susceptibility measurements

## Superconducting quantum interference device (SQUID)

Magnetic measurements were performed with a QuantumDesign MPMS3 SQUID magnetometer. The samples were prepared in a glove box in VSM powder capsules which were sealed with a piece of Teflon tape. Both the capsules and the Teflon tape were dried in a Schlenk flask under vacuum at 110 °C for five days. A brass sample holder was used. The measurement was carried out in VSM mode from 300 K to 2 K in a magnetic field of 7 T. A background correction was applied by subtracting the magnetic moments of an empty capsule sealed with a piece of Teflon tape using the same measurement sequence as for the sample. A diamagnetic correction was performed using Pascal's constants.[5] The simulation of the experimental magnetic data was performed with N. F. Chilton's PHI software (version 3.1.6)[6] using the following spin Hamiltonian:

$$\hat{H} = g * \mu_B * B_0 * \hat{S}$$

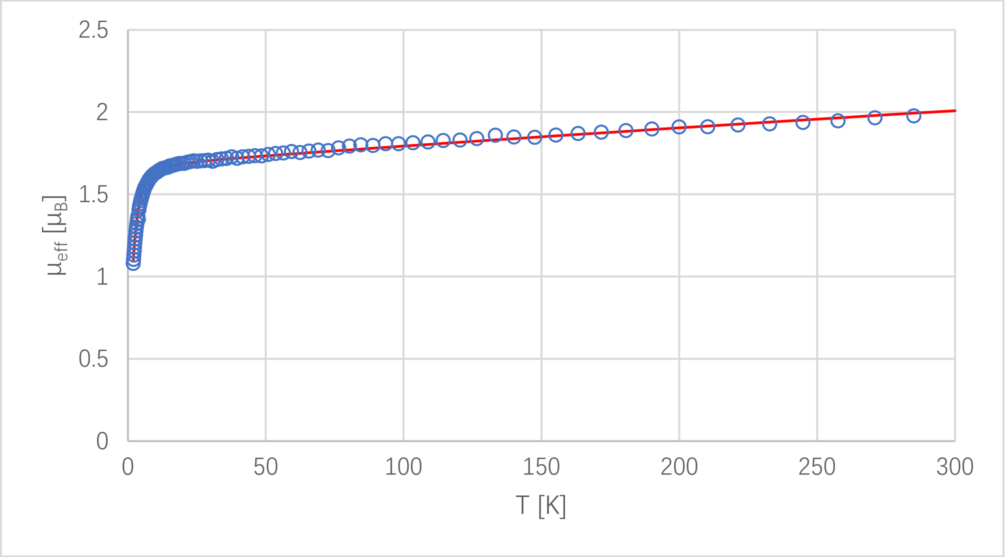


**Figure S60.** Effective magnetic moment of **5**[BAr$^F$]$_2$ in dependence on the temperature as obtained from the SQUID measurement (blue circles) and as simulated (red line).

**Table S21.** Experimental magnetic data of **5**[BAr$^F$]$_2$ obtained from the SQUID measurement.[a]

| ***T*** **[K]** | **Magnetic Field [Oe]** | **Magnetic Moment [emu]** | $\chi_{mol}$ **[cm³ mol⁻¹]** | $\chi_p$[b] **[cm³ mol⁻¹]** | $\mu_{eff}$ **[μB]** |
|---|---|---|---|---|---|
| 300.002 | 69999.55 | 0.00013 | 0.00043 | 0.00163 | 1.9796 |
| 285.088 | 69999.55 | 0.00016 | 0.00051 | 0.00171 | 1.9769 |
| 271.001 | 69999.55 | 0.00018 | 0.00057 | 0.00178 | 1.9646 |
| 257.593 | 69999.55 | 0.00020 | 0.00063 | 0.00184 | 1.9463 |
| 244.875 | 69999.55 | 0.00022 | 0.00071 | 0.00192 | 1.9367 |
| 232.826 | 69999.55 | 0.00024 | 0.00079 | 0.00200 | 1.9280 |
| 221.292 | 69999.55 | 0.00027 | 0.00088 | 0.00209 | 1.9211 |

| | | | | | |
|---|---|---|---|---|---|
| 210.360 | 69999.55 | 0.00030 | 0.00096 | 0.00217 | 1.9102 |
| 199.967 | 69999.55 | 0.00033 | 0.00107 | 0.00228 | 1.9095 |
| 190.089 | 69999.55 | 0.00036 | 0.00116 | 0.00237 | 1.8969 |
| 180.691 | 69999.55 | 0.00039 | 0.00126 | 0.00246 | 1.8873 |
| 171.775 | 69999.55 | 0.00042 | 0.00136 | 0.00257 | 1.8776 |
| 163.308 | 69999.55 | 0.00045 | 0.00147 | 0.00268 | 1.8697 |
| 155.253 | 69999.55 | 0.00049 | 0.00158 | 0.00279 | 1.8596 |
| 147.583 | 69999.55 | 0.00052 | 0.00168 | 0.00289 | 1.8464 |
| 140.073 | 69999.55 | 0.00057 | 0.00184 | 0.00305 | 1.8483 |
| 133.332 | 69999.55 | 0.00063 | 0.00203 | 0.00324 | 1.8588 |
| 126.595 | 69999.55 | 0.00066 | 0.00213 | 0.00334 | 1.8382 |
| 120.489 | 69999.55 | 0.00070 | 0.00227 | 0.00347 | 1.8299 |
| 114.437 | 69999.55 | 0.00075 | 0.00244 | 0.00364 | 1.8265 |
| 108.906 | 69999.55 | 0.00080 | 0.00259 | 0.00379 | 1.8180 |
| 103.439 | 69999.55 | 0.00086 | 0.00277 | 0.00397 | 1.8132 |
| 98.220 | 69999.55 | 0.00091 | 0.00295 | 0.00416 | 1.8069 |
| 93.520 | 69999.55 | 0.00098 | 0.00316 | 0.00437 | 1.8072 |
| 88.935 | 69999.55 | 0.00103 | 0.00333 | 0.00454 | 1.7967 |
| 84.544 | 69999.55 | 0.00111 | 0.00359 | 0.00480 | 1.8007 |
| 80.384 | 69999.55 | 0.00117 | 0.00379 | 0.00500 | 1.7925 |
| 76.440 | 69999.55 | 0.00123 | 0.00399 | 0.00520 | 1.7824 |
| 72.683 | 69999.55 | 0.00128 | 0.00415 | 0.00536 | 1.7654 |
| 69.106 | 69999.55 | 0.00138 | 0.00445 | 0.00566 | 1.7684 |
| 65.703 | 69999.55 | 0.00145 | 0.00470 | 0.00591 | 1.7623 |
| 62.463 | 69999.55 | 0.00153 | 0.00495 | 0.00616 | 1.7537 |
| 59.379 | 69999.55 | 0.00164 | 0.00531 | 0.00652 | 1.7600 |
| 56.440 | 69999.55 | 0.00172 | 0.00558 | 0.00679 | 1.7504 |
| 53.666 | 69999.55 | 0.00183 | 0.00591 | 0.00712 | 1.7477 |
| 51.007 | 69999.55 | 0.00193 | 0.00623 | 0.00744 | 1.7417 |
| 48.487 | 69999.55 | 0.00202 | 0.00654 | 0.00775 | 1.7334 |
| 46.098 | 69999.55 | 0.00215 | 0.00695 | 0.00816 | 1.7345 |
| 43.821 | 69999.55 | 0.00227 | 0.00734 | 0.00855 | 1.7307 |
| 41.652 | 69999.55 | 0.00240 | 0.00775 | 0.00896 | 1.7278 |
| 39.600 | 69999.55 | 0.00251 | 0.00812 | 0.00933 | 1.7190 |
| 37.665 | 69999.55 | 0.00269 | 0.00870 | 0.00991 | 1.7280 |

| | | | | | |
|---|---|---|---|---|---|
| 35.805 | 69999.55 | 0.00281 | 0.00910 | 0.01031 | 1.7178 |
| 34.038 | 69999.55 | 0.00297 | 0.00960 | 0.01080 | 1.7150 |
| 32.358 | 69999.55 | 0.00312 | 0.01010 | 0.01131 | 1.7107 |
| 30.761 | 69999.55 | 0.00326 | 0.01055 | 0.01175 | 1.7004 |
| 29.242 | 69999.55 | 0.00348 | 0.01126 | 0.01247 | 1.7076 |
| 27.799 | 69999.55 | 0.00367 | 0.01186 | 0.01307 | 1.7044 |
| 26.427 | 69999.55 | 0.00387 | 0.01251 | 0.01372 | 1.7028 |
| 25.123 | 69999.55 | 0.00407 | 0.01318 | 0.01439 | 1.7002 |
| 23.883 | 69999.55 | 0.00432 | 0.01398 | 0.01519 | 1.7033 |
| 22.704 | 69999.55 | 0.00454 | 0.01468 | 0.01589 | 1.6986 |
| 21.584 | 69999.55 | 0.00477 | 0.01543 | 0.01664 | 1.6947 |
| 20.518 | 69999.55 | 0.00499 | 0.01614 | 0.01735 | 1.6873 |
| 19.506 | 69999.55 | 0.00527 | 0.01704 | 0.01825 | 1.6873 |
| 18.543 | 69999.55 | 0.00556 | 0.01798 | 0.01919 | 1.6869 |
| 17.628 | 69999.55 | 0.00583 | 0.01885 | 0.02006 | 1.6818 |
| 16.758 | 69999.55 | 0.00612 | 0.01981 | 0.02102 | 1.6784 |
| 15.931 | 69999.55 | 0.00642 | 0.02077 | 0.02198 | 1.6734 |
| 15.145 | 69999.55 | 0.00677 | 0.02189 | 0.02310 | 1.6725 |
| 14.397 | 69999.55 | 0.00705 | 0.02281 | 0.02402 | 1.6630 |
| 13.687 | 69999.55 | 0.00743 | 0.02402 | 0.02523 | 1.6619 |
| 13.011 | 69999.55 | 0.00780 | 0.02523 | 0.02644 | 1.6586 |
| 12.369 | 69999.55 | 0.00819 | 0.02650 | 0.02771 | 1.6556 |
| 11.759 | 69999.55 | 0.00855 | 0.02767 | 0.02888 | 1.6479 |
| 11.178 | 69999.55 | 0.00896 | 0.02897 | 0.03018 | 1.6426 |
| 10.627 | 69999.55 | 0.00935 | 0.03025 | 0.03146 | 1.6351 |
| 10.102 | 69999.55 | 0.00979 | 0.03168 | 0.03288 | 1.6299 |
| 9.603 | 69999.55 | 0.01024 | 0.03314 | 0.03434 | 1.6241 |
| 9.130 | 69999.55 | 0.01069 | 0.03459 | 0.03580 | 1.6168 |
| 8.679 | 69999.55 | 0.01115 | 0.03608 | 0.03728 | 1.6087 |
| 8.251 | 69999.55 | 0.01162 | 0.03759 | 0.03880 | 1.6001 |
| 7.843 | 69999.55 | 0.01212 | 0.03920 | 0.04041 | 1.5921 |
| 7.456 | 69999.55 | 0.01256 | 0.04064 | 0.04185 | 1.5797 |
| 7.088 | 69999.55 | 0.01307 | 0.04228 | 0.04349 | 1.5702 |
| 6.738 | 69999.55 | 0.01357 | 0.04389 | 0.04510 | 1.5590 |
| 6.406 | 69999.55 | 0.01408 | 0.04553 | 0.04674 | 1.5474 |

| | | | | | |
|---|---|---|---|---|---|
| 6.090 | 69999.55 | 0.01460 | 0.04722 | 0.04843 | 1.5358 |
| 5.789 | 69999.55 | 0.01509 | 0.04881 | 0.05002 | 1.5218 |
| 5.498 | 69999.55 | 0.01558 | 0.05041 | 0.05162 | 1.5066 |
| 5.227 | 69999.55 | 0.01605 | 0.05191 | 0.05311 | 1.4901 |
| 4.971 | 69999.55 | 0.01658 | 0.05364 | 0.05485 | 1.4767 |
| 4.727 | 69999.55 | 0.01708 | 0.05526 | 0.05647 | 1.4611 |
| 4.495 | 69999.55 | 0.01755 | 0.05678 | 0.05798 | 1.4437 |
| 4.272 | 69999.55 | 0.01802 | 0.05831 | 0.05952 | 1.4261 |
| 4.062 | 69999.55 | 0.01845 | 0.05968 | 0.06089 | 1.4065 |
| 3.862 | 69999.55 | 0.01782 | 0.05765 | 0.05886 | 1.3483 |
| 3.671 | 69999.55 | 0.01928 | 0.06237 | 0.06358 | 1.3663 |
| 3.490 | 69999.55 | 0.01968 | 0.06368 | 0.06489 | 1.3457 |
| 3.318 | 69999.55 | 0.01998 | 0.06464 | 0.06585 | 1.3219 |
| 3.154 | 69999.55 | 0.02035 | 0.06584 | 0.06705 | 1.3005 |
| 2.998 | 69999.55 | 0.02066 | 0.06682 | 0.06803 | 1.2772 |
| 2.850 | 69999.55 | 0.02092 | 0.06769 | 0.06890 | 1.2532 |
| 2.710 | 69999.55 | 0.02116 | 0.06844 | 0.06965 | 1.2285 |
| 2.574 | 69999.55 | 0.02142 | 0.06930 | 0.07051 | 1.2048 |
| 2.449 | 69999.55 | 0.02157 | 0.06978 | 0.07099 | 1.1792 |
| 2.328 | 69999.55 | 0.02175 | 0.07036 | 0.07157 | 1.1544 |
| 2.214 | 69999.55 | 0.02192 | 0.07092 | 0.07212 | 1.1300 |
| 2.104 | 69999.55 | 0.02203 | 0.07127 | 0.07248 | 1.1043 |
| 1.999 | 69999.55 | 0.02217 | 0.07171 | 0.07292 | 1.0797 |

[a] $m$ = 13.0 mg

[b] $\chi_p$ = molar magnetic susceptibility after correction for the diamagnetism of the sample ($\chi_{dia}$ = $-1207.84 \cdot 10^{-6}$ cm$^3$ mol$^{-1}$)

**Table S22.** Fit parameters of **5**[BAr$^F$]$_2$ for the simulation of the SQUID data.

| | | |
|---|---|---|
| Average g factor | $g_{avg}$ | 1.935 |
| | error | 0.002 |
| Temperature-independent paramagnetism [cm$^3$ mol$^{-1}$] | TIP | 0.000511 |
| | error | 0.000006 |

# E. Electron Paramagnetic Resonance (EPR)

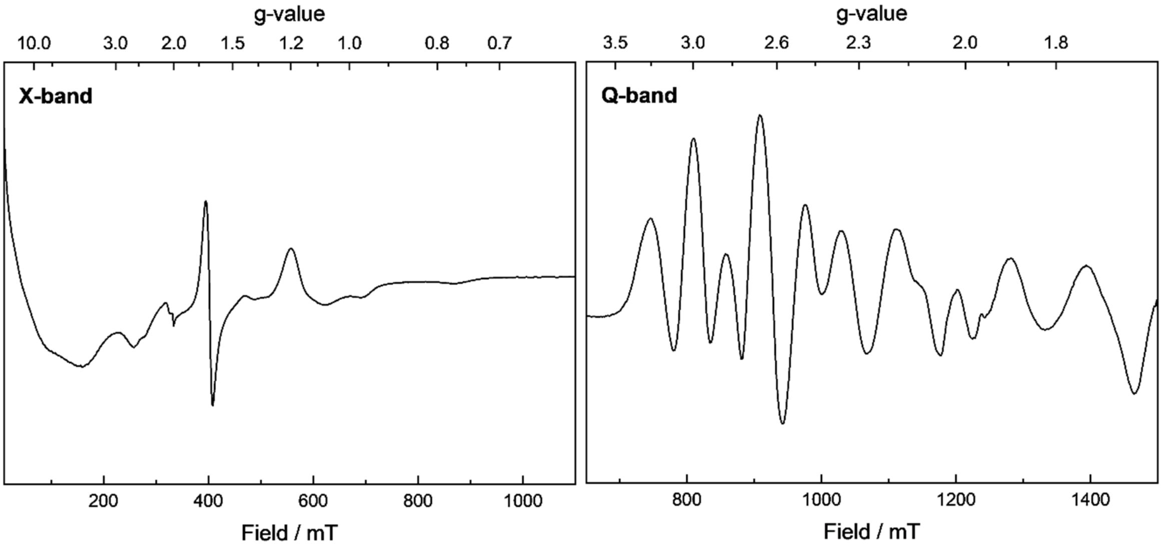


**Figure S61**: EPR spectra of **5**[BAr$^F$]$_2$ recorded at 10 K. Continuous-wave X-band EPR spectrum (left). Pseudo-modulated Q-B and field swept echo EPR spectrum (right).

The paramagnetic properties of compound **5**[BAr$^F$]$_2$ were theoretically evaluated using Orca 5.0.4,[7] employing the B3LYP-D3(BJ)/ZORA-def2-TZVP (Bismuth: SARC-ZORA-TZVP) level of theory.[7–10] The *g*-tensor was computed by placing the gauge origin of angular momentum at the centre of the spin density, with an alignment of the molecular frame's *x*-*y* plane along the Ge-Bi-Ge bond plane. Hyperfine couplings for bismuth were calculated, accounting for isotropic, dipolar, and second-order spin-orbit coupling contributions. Figure S62 shows a representation of the *g*-tensor orientation for dication **5** in **5**[BAr$^F$]$_2$, the eigenvectors associated with the largest *g*-value are plotted along the z-axis of the molecular frame. Table S23 lists the atomic contributions to the paramagnetic spin-orbit coupling term, highlighting that the $g_x$ component is predominantly influenced by the bismuth atom. The principal values of the hyperfine-coupling tensor reveal that the largest component aligns along the *z*-axis of the molecular frame, while the intermediate and small components align along the *y* and *x* axes, respectively (Figure S63).

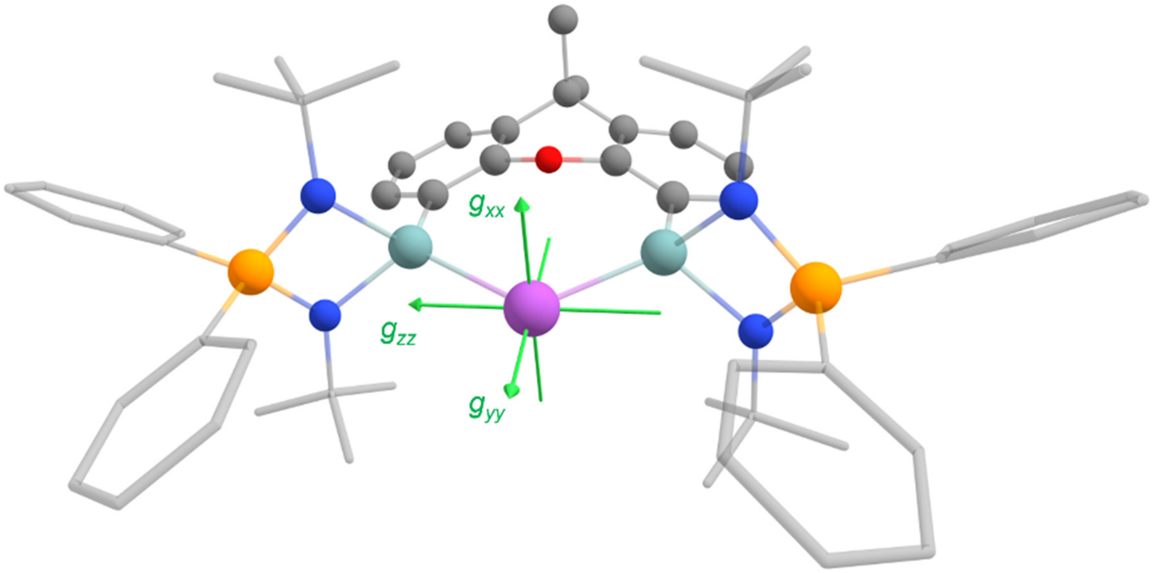


**Figure S62:** Visualisation of the *g*-tensor orientation for dication **5** in **5**[BAr$^F$]$_2$. The molecular frame in the depicted coordinate system is defined by the Ge-Bi-Ge bond aligning along the *x*-*y* plane.

**Table S23:** Atomic contributions to the paramagnetic spin-orbit coupling term.

| | $g_x$ | $g_y$ | $g_z$ | $g_{iso}$ |
|---|---|---|---|---|
| Bi | 0.28212 | 0.12960 | 0.00104 | 0.13759 |
| Ge | -0.00005 | -0.00117 | -0.00102 | -0.00075 |
| Ge | -0.00005 | -0.00118 | -0.00102 | -0.00075 |

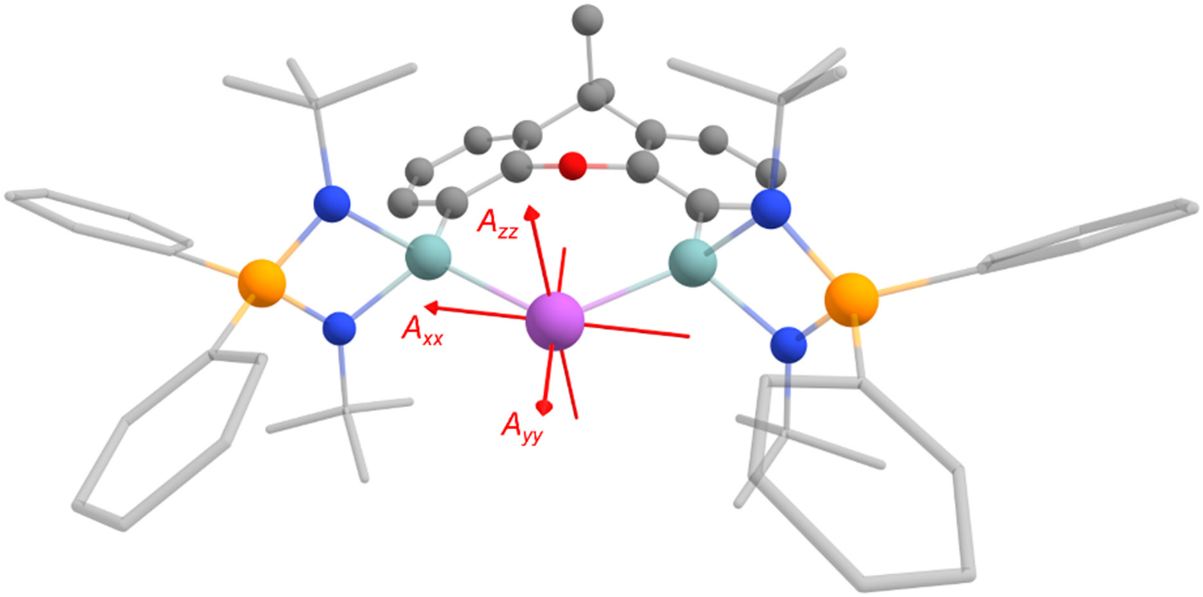


**Figure S63:** Visualisation of the HFC-tensor Orientation for the bismuth atom in cation **5** in **5**[BAr$^F$]$_2$. The molecular frame in the depicted coordinate system is defined by the Ge-Bi-Ge bond aligning along the *x*-*y* plane.

# F. XPS spectra

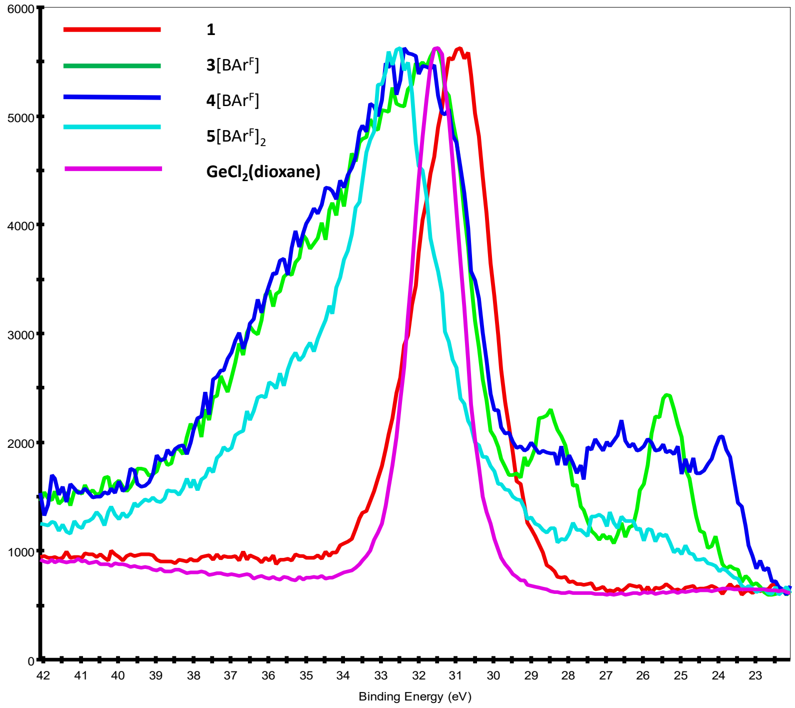


**Figure S64.** The XPS Ge 3d spectra of in $GeCl_2$(dioxane) (31.48 eV), **1** (30.88 eV), **3**[$BAr^F$] (31.48 eV), **4**[$BAr^F$] (32.38 eV) and **5**[$BAr^F$]$_2$ (32.48 eV).

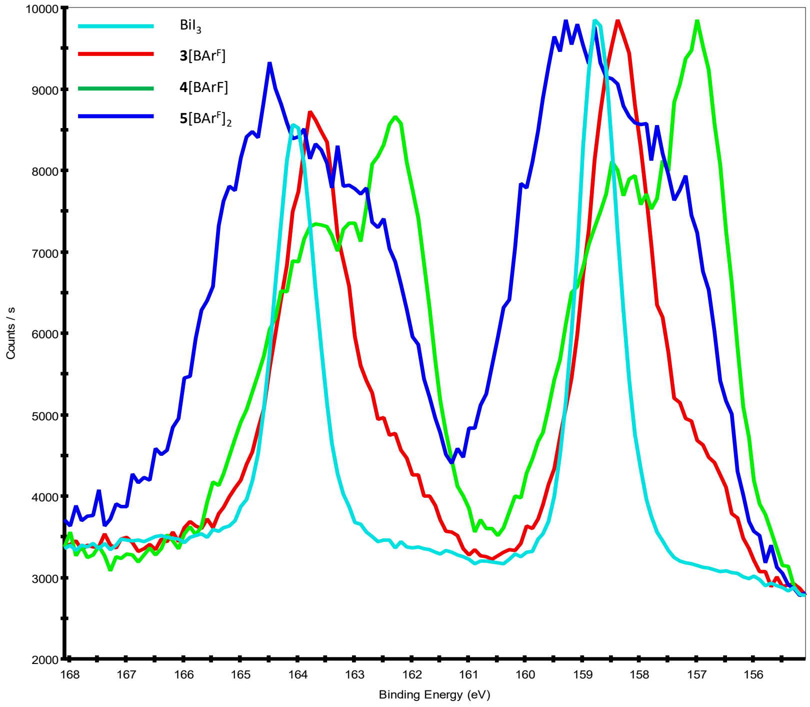


**Figure S65.** The XPS Bi 4f spectra of in $BiI_3$ (164.08 and 158.78 eV), **3**[$BAr^F$] (163.78 and 158.38 eV), **4**[$BAr^F$] (162.18 and 156.98 eV) and **5**[$BAr^F$]$_2$ (164.48 and 159.28 eV).

# G. Theoretical Calculations

## Computational details

The HOMO energy levels of compounds amidinato-chlorosilylene **A**, amidinato-chlorogermylene **B**, iminophosphonamido-chlorogermylene **C** and **1** were performed at the B3LYP-D3/6-31+G(d,p) and BP86-D3(BJ)/def2-TZVP level (Figure S66). The geometry optimizations followed by the harmonic vibrational frequency calculations of cation **3**, cation **4**, dication **5** and neutral **6** complexes in singlet/doublet and triplet/quartet spin states were performed at the BP86-D3(BJ)/def2-SVP level[11–15] using Gaussian16 program.[16] For **6**, $C_s$ symmetric doublet state is a minimum on the potential energy surface. However, for **4** and **5**, the minimum energy geometry is only slightly distorted from $C_s$ symmetry. The $C_s$ symmetric structure is almost isoenergetic to the minimum energy structure but has a very small imaginary frequency (2.4*i* for **4** and 9.4*i* for **5**) for them. Since a symmetric structure will be very helpful for convergence in energy decomposition analysis (EDA), we considered the $C_s$ symmetric structure optimized at the BP86-D3(BJ)/def2-TZVP level for bonding analysis. EPR spectrum was simulated using EasySpin program.[17]

Natural charges were computed using NBO6 program,[18,19] while the quantum theory of atoms-in-molecules (QTAIM)[20] was carried out using Multiwfn program.[21] The energy decomposition analyses (EDA)[22] together with the natural orbitals for chemical valence (NOCV)[23] method were carried out by using the ADF 2019.303 program package.[24] The EDA-NOCV calculations were carried out at the BP86-D3(BJ)/TZ2P-ZORA//BP86-D3(BJ)/def2TZVP level. This calculation divides the intrinsic interaction energy ($\Delta E_{\mathrm{int}}$) between two fragments into three energy components as follows:

$$\Delta E_{\mathrm{int}} = \Delta E_{\mathrm{elstat}} + \Delta E_{\mathrm{Pauli}} + \Delta E_{\mathrm{orb}} + \Delta E_{\mathrm{disp}} \qquad (1).$$

While the electrostatic $\Delta E_{\mathrm{elstat}}$ term accounts for the quasiclassical electrostatic interaction between the unperturbed charge distributions of the prepared fragments, the Pauli repulsion $\Delta E_{\mathrm{Pauli}}$ represents the energy change associated with the transformation from the superposition of the unperturbed electron densities of the isolated fragments to the wavefunction, that properly obeys the Pauli principle through explicit antisymmetrization and renormalization of the production wavefunction. Since we used D3(BJ), it provides us with the dispersion interactions between the fragments. Finally, the mixing of orbitals, charge transfer and polarization between the isolated fragments provide the orbital term $\Delta E_{\mathrm{orb}}$, which can be further decomposed into contributions from each irreducible representation of the point group of the interacting system as follows:

$$\Delta E_{orb} = \sum_{r} \Delta E_{r} \qquad (2)$$

The combination of the EDA with NOCV makes the partition of the total $\Delta E_{\rm orb}$ into pairwise contributions of the orbital interactions which give very important information about the bonding situation. The charge deformation $\Delta\rho_k(r)$, resulting from the mixing of the orbital pairs $\psi_{\rm k}$(r) and $\psi_{\rm -k}$(r) of the interacting fragments provides the amount and the shape of the charge flow due to the orbital interactions (see Equation 3), and the associated energy term $\Delta E_{\rm orb}$ shows the size of stabilizing orbital energy originated from such interaction (Equation 4). More details about this method can be found in the related reviews.[25–27]

$$\Delta\rho_{orb}(r) = \sum_k \Delta\rho_k(r) = \sum_{k=1}^{N/2} v_k[-\psi_{-k}^2(r) + \psi_k^2(r)] \quad (3)$$

$$\Delta E_{orb} = \sum_k \Delta E_k^{orb} = \sum_{k=1}^{N/2} v_k[-F_{-k,-k}^{TS} + F_{k,k}^{TS}] \quad (4)$$

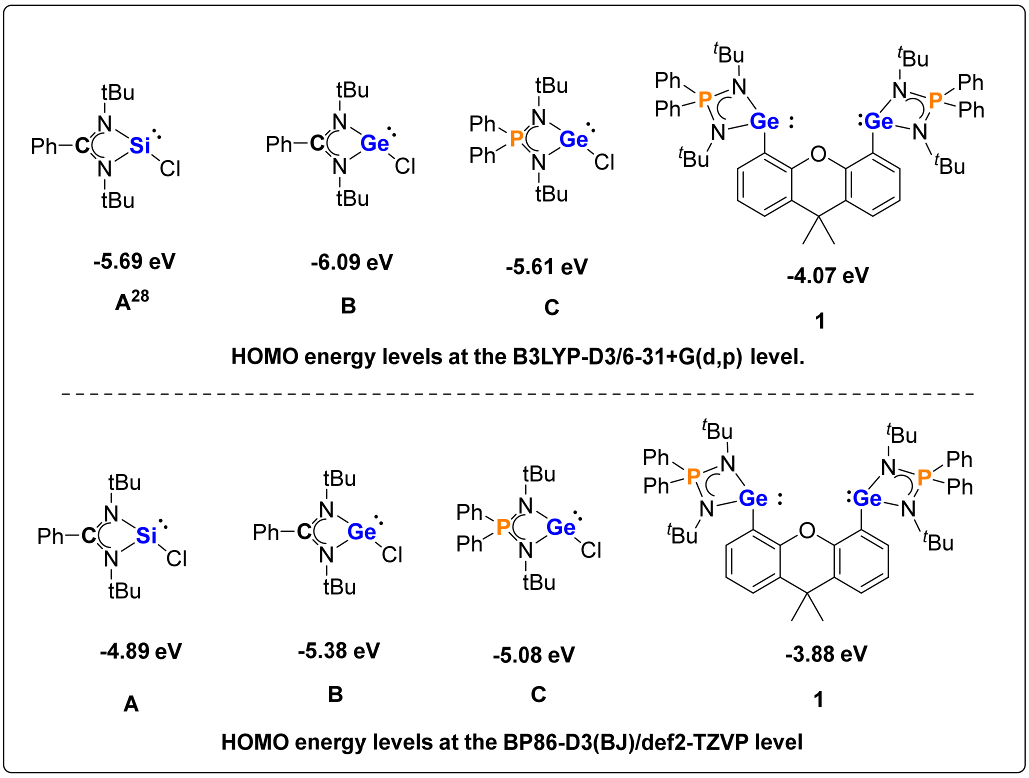


**Figure S66.** Energy levels for the HOMO in amidinato-chlorosilylene **A**[28], amidinato-chlorogermylene **B**, iminophosphonamido-chlorogermylene **C** and bis(germylene) **1**; B3LYP-D3/6-31+G(d,p) level (top), BP86-D3(BJ)/def2-TZVP level (bottom).

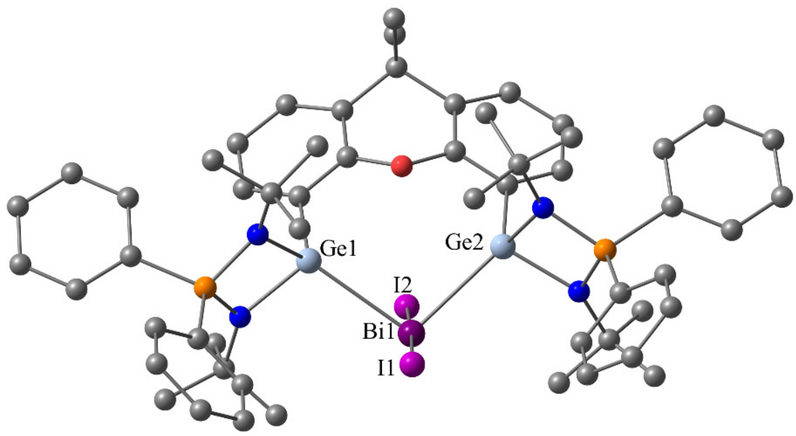


Comput (exp)
Bi1-Ge1: 2.752 (2.773, 2.804)
Bi1-Ge2: 2.757 (2.780, 2.781)
Bi1-I1: 3.030 (3.041, 3.040)
Bi1-I2: 3.054 (3.042, 3.068)
Ge1-Bi1-Ge2: 101.2 (103.9, 107.0)
I1-Bi1-I2: 177.5 (172.3 174.5)

**3**, ($C_1$, $^1$A)

**Figure S67.** The optimized geometry of cation **3** at the BP86-D3(BJ)/def2-TZVP level. Bond distances are in Å and angles are in °. The experimental values are given in parentheses in **3**[BArF], **3**[OTF] fashion.

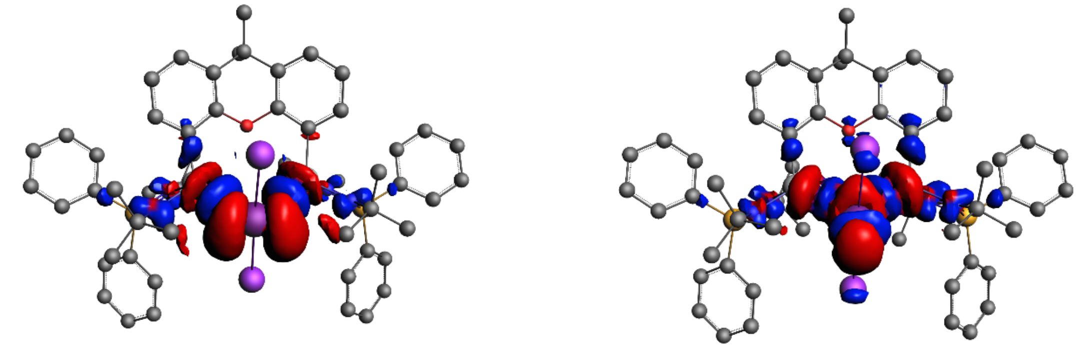

$\Delta E_{orb(1)}$ = -75.7; $|\nu_{1\alpha/1\beta}|$ = 0.68/0.49

$\Delta E_{orb(2)}$ = -66.3; $|\nu_{2\alpha/2\beta}|$ = 0.66/0.55

**Figure S68.** Plot of the deformation densities, $\Delta\rho_{(1)-(2)}$ associated to $\Delta E_{orb(1)-(2)}$ for cation **3** at the BP86-D3(BJ)/TZ2P-ZORA level. The eigenvalues ν indicate the size of the charge flow, and the direction of charge flow is red→blue. The isovalue is 0.001 au.

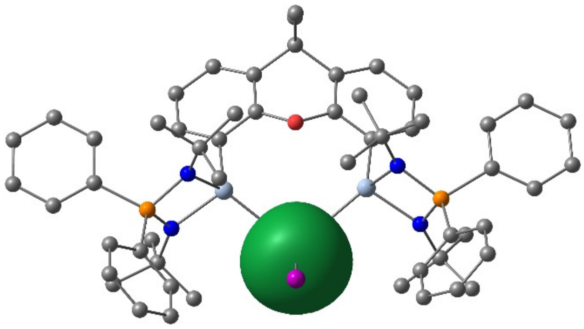

LP on Bi: s(92%) p (8%)
ON: 1.96

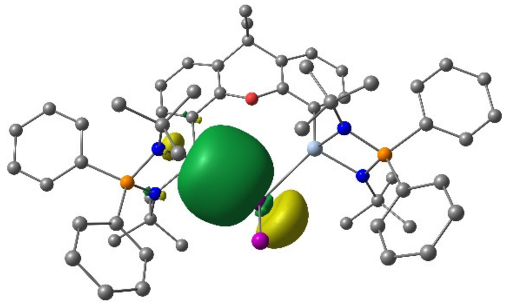

Bi1-Ge1 σ bond
(41%)Bi1: s(4%) p(96%)
(59%)Ge1: s(43%) p(57%)
ON: 1.93

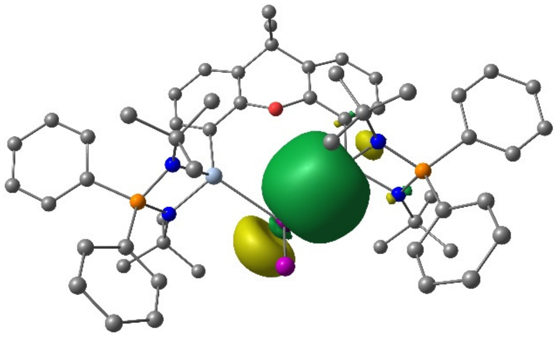

Bi1-Ge2 σ bond:
(41%)Bi1: s(4%) p(96%)
(59%)Ge2: s(43%) p(57%)
ON: 1.93

**Figure S69**. The natural orbitals and their atomic compositions in cation **3**.

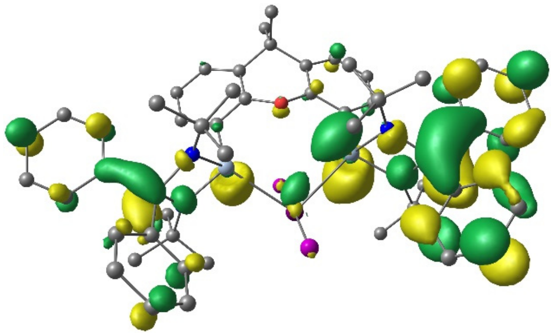

LUMO

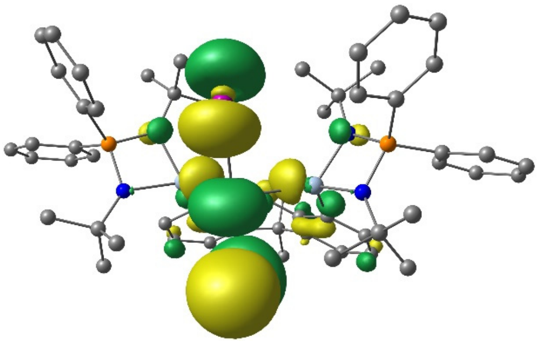

HOMO

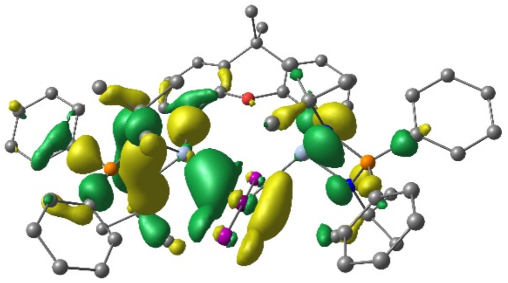

HOMO-8

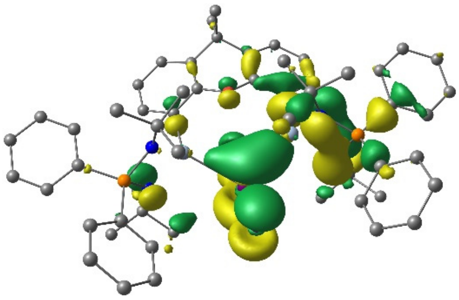

HOMO-9

**Figure S70.** Some relevant Kohn-Sham molecular orbitals of cation **3**.

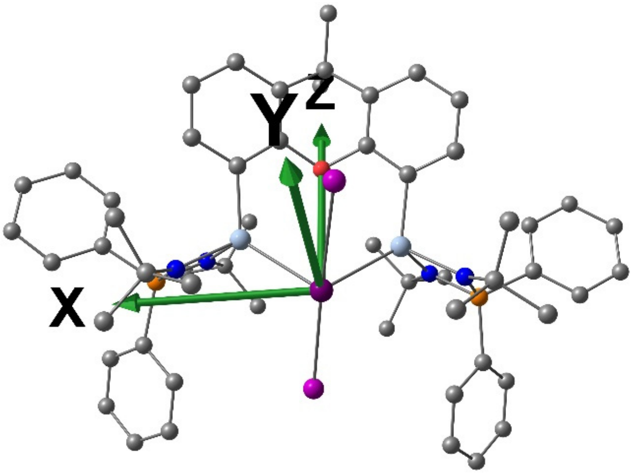


**Table 24.** The EDA results for the cation of complex **3** considering $BiI_2$ and the remaining part, L in different charges and electronic states as interacting fragments at the BP86-D3(BJ)/TZ2P-ZORA//BP86-D3(BJ)/def2-TZVP level.

| Energy | Orbital Interaction | $BiI_2$ (D) + $L^+$ (D) | $BiI_2^-$ (S) + $L^{2+}$ (S) | $BiI_2^-$ (T) + $L^{2+}$ (T) | $BiI_2^+$ (S) + L (S) |
|---|---|---|---|---|---|
| $\Delta E_{int}$ | | -141.5 | -252.2 | -234.3 | -216.0 |
| $\Delta E_{Pauli}$ | | 248.6 | 361.0 | 272.8 | 300.8 |
| $\Delta E_{disp}$[a] | | -49.5 | -49.5 | -49.5 | -49.5 |
| $\Delta E_{elstat}$[a] | | -153.4 | -320.7 | -280.2 | -213.7 |
| $\Delta E_{orb}$[a] | | -187.2 | -243.1 | -177.5 | -253.5 |
| $\Delta E_{orb(1)}$[b] | Ge-Bi-Ge electron-sharing (+,-) σ bond | - | - | -75.7 (42.6%) | - |
| $\Delta E_{orb(2)}$[b] | Ge-Bi-Ge electron-sharing (+,+) σ bond | - | - | -66.3 (37.4%) | - |
| $\Delta E_{orb(rest)}$[b] | | - | - | -35.5 (20.0%) | - |

[a]The percentage contribution with respect to total attraction is given in parentheses.

[b]The percentage contribution in parentheses is given with respect to total orbital interaction.

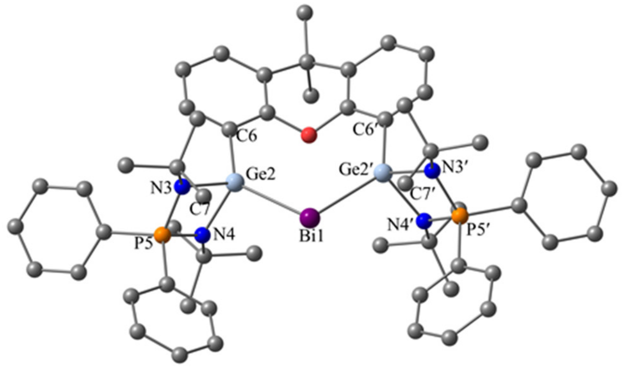


Comput (exp)

Bi1-Ge2: 2.655 (2.663, 2.667)
Ge2-C6: 1.965 (1.964, 1.958)
Ge2-N3: 1.965 (1.943, 1.943)
Ge2-N4: 1.962 (1.944, 1.943)
N3-P5: 1.652 (1.632, 1.637)
N4-P5: 1.634 (1.624, 1.620)
Ge2-Bi1-Ge2′: 102.4 (104.0)
Bi1-Ge2-C6: 131.5 (129.9, 129.2)
N3-Ge2-C6: 106.2 (106.8, 107.2)
N4-Ge2-C6: 106.3 (105.1, 107.3)
N3-Ge2-N4: 75.1 (75.1, 75.1)
N3-P5-N4: 93.6 (93.3, 93.4)

**4 ($C_s$, $^1$A′)**; q(Bi) = -0.23 e

**4** → Bi [$^4$S] + bis(NHGe)$^+$ [D] $D_e$ = 87.0 kcal/mol ($\Delta G^{298}$ = 76.3 kcal/mol)

**4** → Bi$^+$ [$^3$P] + bis(NHGe) [S] $D_e$ = 180.7 kcal/mol ($\Delta G^{298}$ = 164.9 kcal/mol)

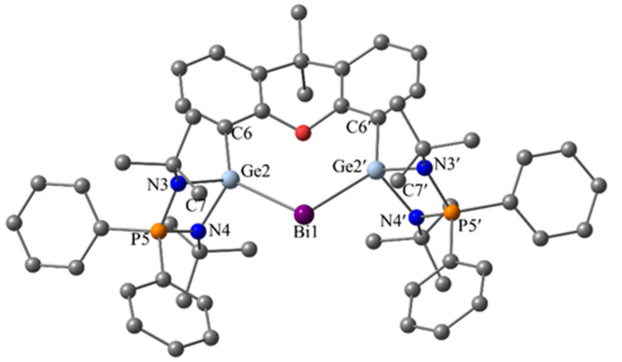


Comput (exp)

Bi1-Ge2: 2.715 (2.715, 2.711)
Ge2-C6: 1.946 (1.941, 1.944)
Ge2-N3: 1.926 (1.918, 1.905)
Ge2-N4: 1.923 (1.906, 1.906)
N3-P5: 1.664 (1.645, 1.643)
N4-P5: 1.651 (1.644, 1.638)
Ge2-Bi1-Ge2′: 105.2 (107.6)
Bi1-Ge2-C6: 130.2 (128.0, 127.5)
N3-Ge2-C6: 110.3 (110.0, 114.3)
N4-Ge2-C6: 112.5 (112.1, 108.3)
N3-Ge2-N4: 76.9 (77.1, 76.6)
N3-P5-N4: 92.4 (92.8, 92.0)

**5 ($C_s$, $^2$A′)**; q(Bi) = 0.26 e

**5** → Bi [$^4$S] + bis(NHGe)$^{2+}$ [S] $D_e$ = 64.1 kcal/mol ($\Delta G^{298}$ = 60.8 kcal/mol)

**5** → Bi$^+$ [$^3$P] + bis(NHGe)$^+$ [D] $D_e$ = 112.9 kcal/mol ($\Delta G^{298}$ = 104.9 kcal/mol)

**5** → Bi$^{2+}$ [$^2$P] + bis(NHGe) [S] $D_e$ = 372.9 kcal/mol ($\Delta G^{298}$ = 359.9 kcal/mol)

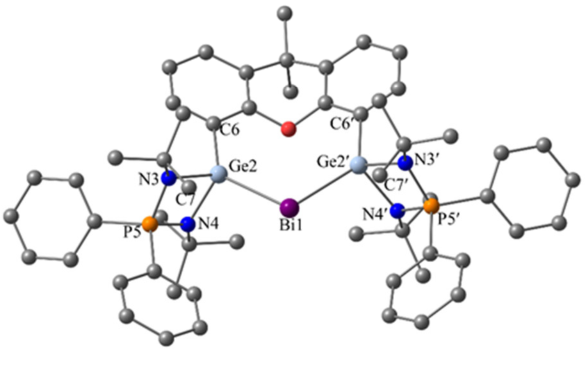


Bi1-Ge2: 2.654
Ge2-C6: 1.968
Ge2-N3: 1.954
Ge2-N4: 1.954
N3-P5: 1.669
N4-P5: 1.647
Ge2-Bi1-Ge2′: 103.3
Bi1-Ge2-C6: 130.2
N3-Ge2-C6: 106.7
N4-Ge2-C6: 106.1
N3-Ge2-N4: 75.0
N3-P5-N4: 91.6

**6 ($C_s$, $^2$A′)**; q(Bi) = -0.25 e

**6** → Bi [$^4$S] + bis(NHGe) [S] $D_e$ = 62.2 kcal/mol ($\Delta G^{298}$ = 52.1 kcal/mol)

**Figure S71.** Optimized geometries of **4**, **5**, and **6** at the BP86-D3(BJ)/def2-TZVP level. The bond distances are in Å and angles are in °. Hydrogen atoms are omitted for clarity. The Ge-Bi bond dissociation energies are given at 0 K ($D_e$) and at 298 K ($\Delta G^{298}$). For **4**, **5**, and **6**, the thermal corrections to free energy are calculated at BP86-D3(BJ)/def2-SVP.

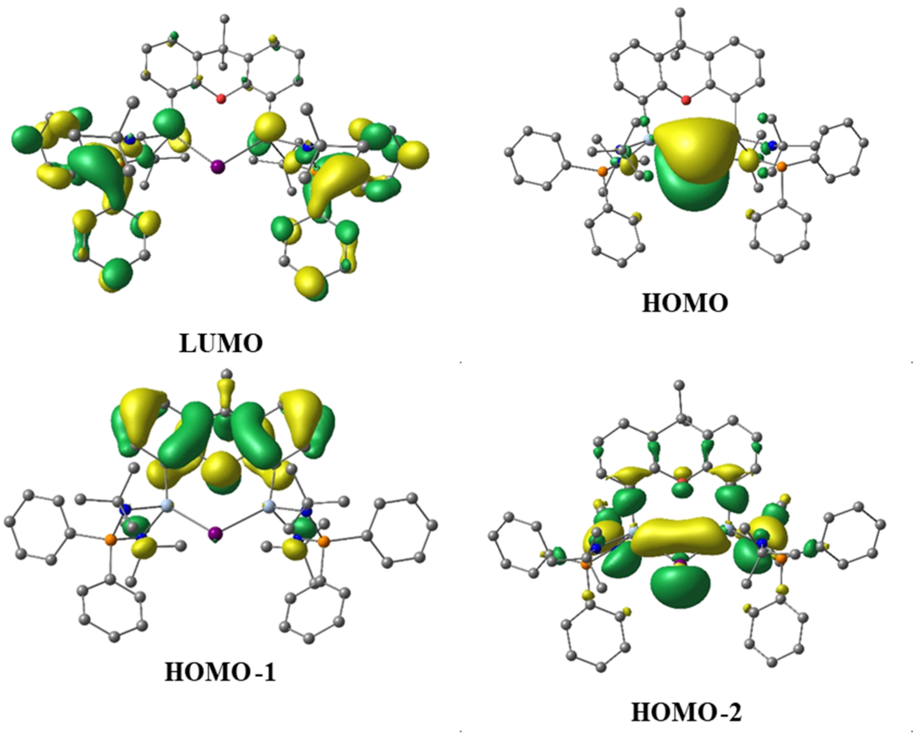


**Figure S72.** Some relevant Kohn-Sham molecular orbitals in cation **4** complex.

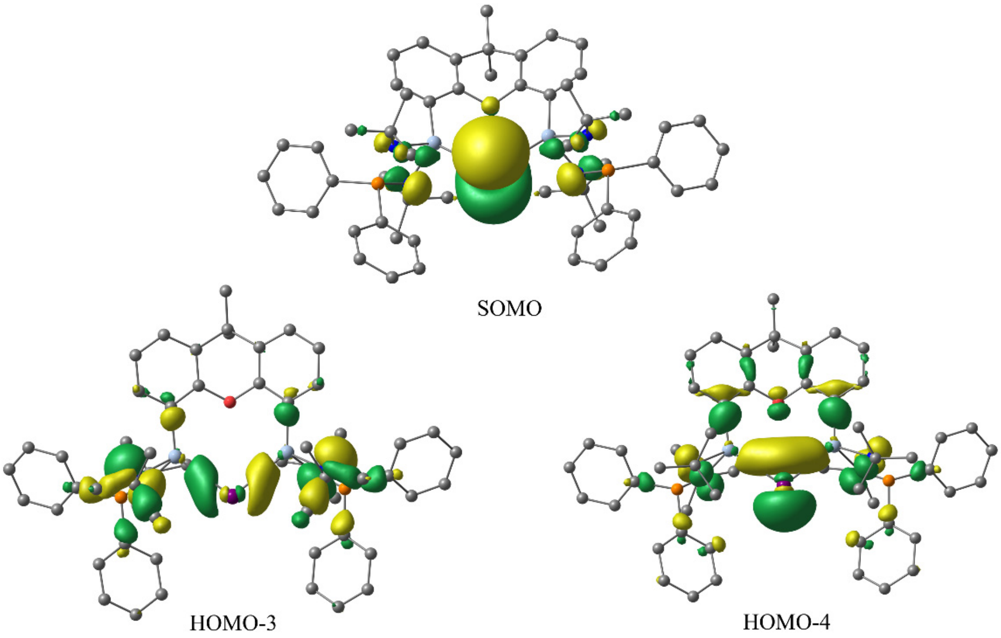


**Figure S73.** Some relevant Kohn-Sham molecular orbitals of dicatonic **5** complex.

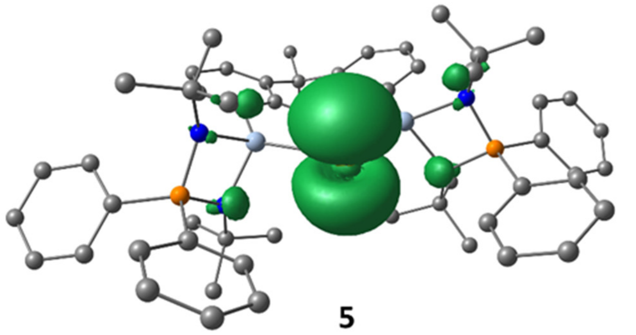


| | NBO Spin |
|---|---|
| Bi1 | 0.87 |
| Ge2, Ge2′ | 0.00 |
| N3, N3′ | 0.01 |
| N4, N4′ | 0.02 |
| C7, C7′ | 0.01 |

**Figure S74.** The spin density plot for dication **5** and the values of NBO spin density on the atomic centers.

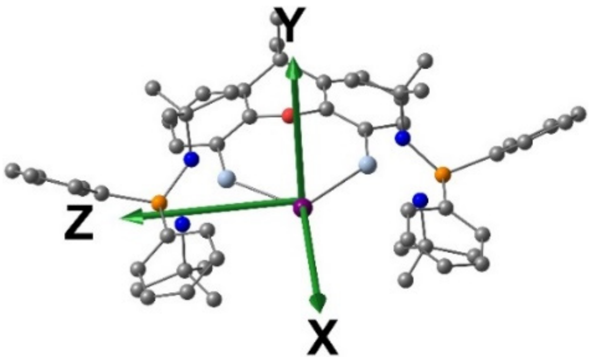


**Table S25.** The EDA results for cation **4** considering Bi and the remaining part, L in different charges and electronic states as interacting fragments at the BP86-D3(BJ)/TZ2P-ZORA//BP86-D3(BJ)/def2-TZVP level.

| Energy | Bi (D, $6s^26p_x^16p_y^26p_z^0$) + $L^+$ (D) | Bi (D, $6s^26p_x^06p_y^26p_z^1$) + $L^+$ (D) | Bi (D, $6s^26p_x^06p_y^16p_z^2$) + $L^+$ (D) | Bi (D, $6s^26p_x^26p_y^16p_z^0$) + $L^+$ (D) | Bi (Q, $6s^26p_x^16p_y^16p_z^1$) + $L^+$ (Q) | $Bi^+$ (S, $6s^26p_x^06p_y^26p_z^0$) + L (S) |
|---|---|---|---|---|---|---|
| $\Delta E_{int}$ | -147.0 | -134.1 | -181.1 | -204.9 | -151.6 | -228.4 |
| $\Delta E_{Pauli}$ | 204.2 | 315.6 | 387.0 | 173.4 | 269.8 | 258.1 |
| $\Delta E_{disp}$[a] | -26.2 | -26.2 | -26.2 | -26.2 | -26.2 | -26.2 |
| $\Delta E_{elstat}$[a] | -154.8 | -228.0 | -269.4 | -124.2 | -190.7 | -215.8 |
| $\Delta E_{orb}$[a] | -170.2 | -195.5 | -272.6 | -227.9 | -204.6 | -244.6 |
| Energy | $Bi^+$ (T, $6s^26p_x^16p_y^16p_z^0$) + L (T) | $Bi^+$ (T, $6s^26p_x^16p_y^06p_z^1$) + L (T) | $Bi^+$ (T, $6s^26p_x^06p_y^16p_z^1$) + L (T) | $Bi^-$ (S, $6s^26p_x^26p_y^26p_z^0$) + $L^{2+}$ (S) | $Bi^-$ (S, $6s^26p_x^06p_y^26p_z^2$) + $L^+$ (S) | $Bi^-$ (T, $6s^26p_x^16p_y^26p_z^1$) + $L^+$ (T) |
| $\Delta E_{int}$ | -235.0 | -273.8 | -221.7 | -306.9 | -284.1 | -265.4 |
| $\Delta E_{Pauli}$ | 183.5 | 242.2 | 289.5 | 228.8 | 474.7 | 343.9 |
| $\Delta E_{disp}$[a] | -26.2 | -26.2 | -26.2 | -26.2 | -26.2 | -26.2 |
| $\Delta E_{elstat}$[a] | -141.3 | -155.0 | -209.9 | -283.8 | -451.4 | -359.7 |
| $\Delta E_{orb}$[a] | -251.1 | -334.9 | -275.2 | -225.7 | -281.3 | -223.4 |

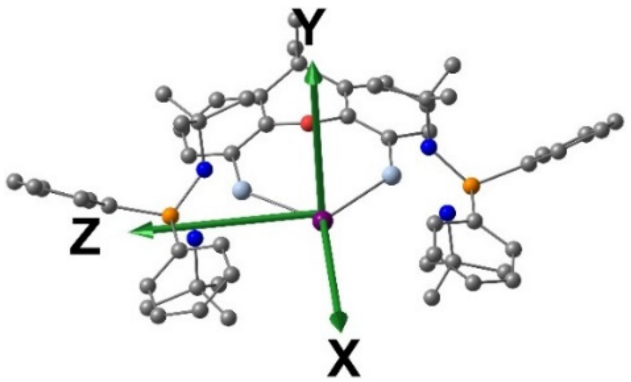


**Table S26.** The EDA results for dication **5** considering Bi and the remaining part, L in different charges and electronic states as interacting fragments at the BP86-D3(BJ)/TZ2P-ZORA//BP86-D3(BJ)/def2-TZVP level.

| Energy | Bi (Q, $6s^26p_x^16p_y^16p_z^1$) + $L^{2+}$ (T) | Bi (D, $6s^26p_x^26p_y^16p_z^0$) + $L^{2+}$ (S) | Bi (D, $6s^26p_x^06p_y^16p_z^2$) + $L^{2+}$ (S) | Bi (Q, $6s^16p_x^26p_y^16p_z^1$) + $L^{2+}$ (T) | Bi (D, $6s^06p_x^26p_y^16p_z^2$) + $L^{2+}$ (S) | $Bi^+$ (T, $6s^26p_x^16p_y^16p_z^0$) + $L^+$ (D) |
|---|---|---|---|---|---|---|
| $\Delta E_{int}$ | -124.6 | -163.3 | -141.7 | -361.2 | -605.9 | -155.5 |
| $\Delta E_{Pauli}$ | 240.3 | 144.1 | 345.4 | 222.6 | 293.3 | 161.0 |
| $\Delta E_{disp}$[a] | -27.3 | -27.3 | -27.3 | -27.3 | -27.3 | -27.3 |
| $\Delta E_{elstat}$[a] | -171.7 | -108.7 | -248.3 | -182.5 | -255.9 | -81.1 |
| $\Delta E_{orb}$[a] | -165.9 | -171.4 | -211.5 | -373.9 | -616.0 | -208.1 |
| Energy | $Bi^+$ (T, $6s^26p_x^06p_y^16p_z^1$) + $L^+$ (D) | $Bi^+$ (T, $6s^16p_x^26p_y^16p_z^0$) + $L^+$ (D) | $Bi^+$ (T, $6s^06p_x^26p_y^16p_z^1$) + $L^+$ (D) | $Bi^{2+}$ (D, $6s^26p_x^06p_y^16p_z^0$) + L (S) | $Bi^{2+}$ (D, $6s^06p_x^26p_y^16p_z^0$) + L (S) | |
| $\Delta E_{int}$ | -143.6 | -416.2 | -646.0 | -409.9 | -954.7 | |
| $\Delta E_{Pauli}$ | 260.0 | 136.7 | 204.1 | 237.1 | 165.0 | |
| $\Delta E_{disp}$[a] | -27.3 | -27.3 | -27.3 | -27.3 | -27.3 | |
| $\Delta E_{elstat}$[a] | -148.7 | -96.0 | -169.9 | -237.1 | -261.3 | |
| $\Delta E_{orb}$[a] | -227.6 | -429.6 | -652.9 | -382.6 | -831.1 | |

**Table S27.** The Cartesian coordinates of **3**

| **3** |
|---|
| E = -8080.200624 au |
| 1 1 |
| Bi -0.03413200 -0.64834600 -1.43607300 |
| I -0.02402300 -3.41320400 -0.19540300 |
| I -0.14229200 2.09851700 -2.76637600 |
| Ge 2.13649600 0.43755700 -0.12738100 |
| Ge -2.11919700 0.49439400 -0.05069000 |
| P 4.37605700 -0.85781200 0.48392100 |
| P -4.39843600 -0.78396400 0.44409800 |
| O 0.08264100 2.42904200 0.82973400 |
| N 3.11657600 -0.29327600 1.37228000 |
| N -3.14308400 -0.29558800 1.38906300 |
| N 3.70088300 -0.36917200 -0.94128900 |
| N -3.73332700 -0.14231900 -0.92060000 |
| C -1.09886300 3.14419500 0.68298600 |
| C -2.22282600 2.42415900 0.27685700 |
| C 2.70207500 -0.51488200 2.77586300 |
| C -5.96124500 -0.02300200 0.93149600 |
| C 5.92229400 -0.00860100 0.86770100 |
| C -1.12716200 4.52220000 0.93117100 |
| C -4.62823000 -2.56572100 0.42248600 |
| C 1.25795700 3.12427600 0.57416000 |
| C 4.24094900 -0.37385700 -2.32623700 |
| C 2.33325500 2.37632500 0.09249600 |
| C -5.50896300 -3.20868300 1.31180000 |
| H -6.16744100 -2.62405300 1.95505900 |
| C -3.43040600 3.11204500 0.11148300 |
| H -4.31802500 2.57474900 -0.21957700 |
| C 5.84838700 1.23907500 1.51109300 |
| H 4.88009000 1.61302500 1.84449600 |
| C 7.16770900 -0.48779500 0.42448400 |
| H 7.24048700 -1.45333900 -0.07731900 |
| C 4.64540200 -2.62851600 0.61307000 |
| C 3.54186700 3.03532100 -0.15925500 |
| H 4.40138000 2.47578000 -0.52422300 |
| C -8.33120600 1.33114200 1.55678500 |
| H -9.25517200 1.85685900 1.80264100 |
| C 1.33473900 4.50057900 0.81746800 |
| C 3.91719000 -0.86968200 3.64441100 |
| H 4.37691300 -1.81346000 3.31986200 |

H 3.59362300 -1.00213000 4.68617100
H 4.67756300 -0.07750900 3.62139400
C -2.73548200 -0.67338600 2.76208700
C -2.34486400 5.18364700 0.73072000
H -2.40684000 6.25892000 0.89736300
C -7.20525800 -0.57973700 0.58635300
H -7.25309900 -1.53641600 0.06545700
C -5.91451300 1.21879300 1.58874100
H -4.94880000 1.64916600 1.85380700
C 5.66554200 -3.18530400 1.40429500
H 6.38551200 -2.54082600 1.90950000
C 2.07752100 0.79595400 3.27298600
H 2.80732500 1.61716000 3.23813200
H 1.72333100 0.68366100 4.30736400
H 1.21583900 1.08414700 2.65296800
C -8.38422300 0.09684300 0.90154600
H -9.34704100 -0.34026000 0.63314500
C -3.48890000 4.48930800 0.32815900
H -4.42634400 5.02640000 0.17692700
C 0.13718700 5.19035200 1.46888800
C 3.64352000 4.41320400 0.04035300
H 4.58040700 4.92780600 -0.17782300
C 1.67582000 -1.65321900 2.82206300
H 0.82417500 -1.43413200 2.16306800
H 1.29461700 -1.80035400 3.84308800
H 2.11982300 -2.59511100 2.47253800
C -7.09580300 1.89139000 1.89819100
H -7.05242400 2.85348500 2.41084300
C -4.20996800 -0.08027600 -2.33012600
C -2.17361100 -2.10131700 2.79617600
H -1.36806500 -2.22542500 2.05988600
H -2.95172800 -2.84018900 2.56223300
H -1.77440400 -2.33176600 3.79479100
C -4.66828800 -5.35732100 0.57790600
H -4.68451200 -6.44660400 0.63901800
C 2.54727600 5.13494300 0.52030400
H 2.64577600 6.20882500 0.67848000
C 5.74504700 -4.57026200 1.55687100
H 6.53874300 -5.00236200 2.16799200
C -3.76036800 -3.32967300 -0.37447600
H -3.04598900 -2.83377000 -1.03106600
C 8.32013700 0.27348100 0.62331400
H 9.28366000 -0.10326000 0.27724200
C 3.69953300 -3.46592300 -0.00216900

H 2.88361900 -3.03673600 -0.58551500
C 7.00353900 1.99490600 1.70754400
H 6.93829600 2.96154800 2.20913200
C -5.52730600 -4.60166200 1.38348500
H -6.21065600 -5.09894000 2.07332800
C 4.80694400 -5.40029100 0.93393900
H 4.87244800 -6.48241800 1.05822900
C 3.05203400 -0.48978200 -3.28674300
H 3.40350600 -0.49601800 -4.32795900
H 2.36621000 0.36667500 -3.18227700
H 2.49952200 -1.42543300 -3.10920200
C -3.78247800 -4.72043200 -0.29669900
H -3.09254300 -5.30281100 -0.90828700
C 8.23962600 1.51423700 1.26351500
H 9.14295600 2.10629900 1.41814000
C 0.20742500 4.91414100 2.99734900
H -0.66118800 5.36119300 3.50267100
H 1.12569400 5.34913500 3.41836900
H 0.21030200 3.83465000 3.20380000
C -1.65031300 0.33200500 3.16285500
H -1.26085000 0.10399800 4.16419200
H -2.04035900 1.35961000 3.15706000
H -0.80382900 0.28112500 2.45969400
C 0.13961300 6.70477100 1.23034200
H 0.09044600 6.94605200 0.15920100
H 1.04491200 7.16185400 1.65156800
H -0.71240100 7.17830800 1.73608500
C -5.54738600 -0.81977500 -2.46708600
H -5.86872500 -0.79591900 -3.51724800
H -6.33133600 -0.33570200 -1.86966000
H -5.46223900 -1.87368200 -2.16596300
C -3.92953000 -0.56046900 3.72082100
H -4.74420100 -1.23759800 3.42508100
H -4.32563700 0.46278800 3.75417300
H -3.61595100 -0.84481700 4.73515000
C 3.78036500 -4.84664300 0.16116000
H 3.03155600 -5.48550600 -0.30908800
C 5.16716600 -1.58114800 -2.52803100
H 4.64021300 -2.52702300 -2.34968500
H 6.04053300 -1.53827400 -1.86209200
H 5.54326400 -1.58288300 -3.56030100
C 5.00773300 0.92684300 -2.61185500
H 5.44227800 0.90017400 -3.62158100
H 5.82654200 1.06622500 -1.89099300

H 4.33423200 1.79293000 -2.55874900
C -3.16612700 -0.74121900 -3.24356100
H -2.94966100 -1.77084000 -2.92490400
H -2.23501200 -0.15382700 -3.25748200
H -3.53438200 -0.77437300 -4.27886000
C -4.38531300 1.38768100 -2.74032900
H -4.67469200 1.45375300 -3.79901800
H -3.44579500 1.94563800 -2.61389300
H -5.17050500 1.86785700 -2.13953200

**Table S28.** The Cartesian coordinates of the studied complexes **4**, **5** and **6** at the BP86-D3(BJ)/def2-SVP level in two spin states.

**4** (in Singlet) ($C_1$ symmetry)

E = -7480.994037 au

1 1
Ge -2.06090200 0.46960400 -0.25911900
Ge 2.07630900 0.35414200 -0.43743100
P -4.38363600 -0.81628600 0.22999500
P 4.32959700 -0.91962200 0.30882900
O 0.14439700 2.57003800 0.18302600
N -3.21072400 -0.16432900 1.20258200
N -3.70065100 -0.16960300 -1.15301500
N 3.17332100 -0.07257100 1.13791100
N 3.67674300 -0.50617500 -1.17873600
C -2.18341300 2.43274800 -0.26318700
C -3.40843900 3.08106900 -0.51224400
H -4.31149600 2.47436300 -0.66570300
C -3.46996200 4.48076200 -0.58621200
H -4.42871200 4.98119300 -0.79090700
C -2.30537900 5.25008700 -0.40840400
H -2.36877800 6.34560200 -0.47632600
C -1.06727100 4.64009100 -0.13478700
C -1.04675300 3.23113500 -0.06601000
C 0.23919600 5.39305000 0.15243300
C 0.21725300 6.82671200 -0.39812800
H 0.07160800 6.84211700 -1.49702000
H 1.16360100 7.35197100 -0.16222800
H -0.59393300 7.41676400 0.07183100
C 0.44240900 5.43449700 1.69612000

| | | | |
|---|---|---|---|
| H | -0.38601300 | 5.99324900 | 2.17763700 |
| H | 1.40131300 | 5.93409600 | 1.94360000 |
| H | 0.46503100 | 4.41280100 | 2.12525300 |
| C | 1.28723300 | 3.15577300 | -0.33963400 |
| C | 1.37721700 | 4.55745600 | -0.44872900 |
| C | 2.54578700 | 5.07668400 | -1.03771000 |
| H | 2.65940600 | 6.16405600 | -1.15449500 |
| C | 3.57838500 | 4.22585500 | -1.47424300 |
| H | 4.47919400 | 4.65600900 | -1.93804400 |
| C | 3.46330800 | 2.83524600 | -1.31762500 |
| H | 4.26452500 | 2.16467300 | -1.66259700 |
| C | 2.30463400 | 2.28025200 | -0.74311100 |
| C | 3.18936000 | 0.53036500 | 2.48899500 |
| C | 3.79298200 | -0.46352700 | 3.49865300 |
| H | 3.76441500 | -0.03804300 | 4.52214800 |
| H | 4.85445400 | -0.68060900 | 3.26291700 |
| H | 3.23248500 | -1.41955500 | 3.50164300 |
| C | 1.72271800 | 0.81024900 | 2.85753300 |
| H | 1.13534300 | -0.12870900 | 2.84579200 |
| H | 1.25167400 | 1.51062400 | 2.13713000 |
| H | 1.65400100 | 1.26353300 | 3.86677700 |
| C | 3.99682900 | 1.84532400 | 2.47931800 |
| H | 3.97487500 | 2.33006400 | 3.47715200 |
| H | 3.57680700 | 2.55340000 | 1.73706200 |
| H | 5.05640100 | 1.65790000 | 2.21287700 |
| C | 3.85082200 | -1.17281800 | -2.49660900 |
| C | 5.35389400 | -1.35564500 | -2.77544700 |
| H | 5.88498900 | -0.38336200 | -2.76800600 |
| H | 5.50628500 | -1.82852200 | -3.76636200 |
| H | 5.82927600 | -2.01327500 | -2.01855200 |
| C | 3.13561100 | -2.53946000 | -2.54902700 |
| H | 3.54435400 | -3.24641100 | -1.80209600 |
| H | 3.24776300 | -2.99778200 | -3.55274000 |
| H | 2.04856000 | -2.41710000 | -2.35789700 |
| C | 3.23508000 | -0.22996400 | -3.54670500 |
| H | 2.15407300 | -0.07140400 | -3.34504800 |
| H | 3.32817800 | -0.66733500 | -4.56087800 |
| H | 3.73945700 | 0.75669900 | -3.54192400 |
| C | 4.24131600 | -2.69741200 | 0.66755700 |
| C | 5.28228800 | -3.59602600 | 0.34391000 |
| H | 6.24574200 | -3.22093700 | -0.03427400 |
| C | 5.08295600 | -4.97735200 | 0.49263300 |
| H | 5.89481300 | -5.67706800 | 0.24170300 |
| C | 3.84740000 | -5.46603000 | 0.95522500 |

H 3.69494900 -6.55053000 1.06848100
C 2.80926900 -4.57240500 1.27452700
H 1.84211900 -4.95411200 1.63627300
C 3.00163800 -3.19019400 1.13541100
H 2.19142400 -2.48033000 1.36968700
C 6.04460600 -0.37966100 0.54779900
C 6.49662000 0.75892000 -0.15433800
H 5.83104500 1.24016000 -0.88408700
C 7.77736700 1.27212000 0.09119200
H 8.12680100 2.15840300 -0.46013800
C 8.61313400 0.65625500 1.04102400
H 9.61885800 1.06074900 1.23302300
C 8.16507200 -0.47402200 1.74686100
H 8.81696000 -0.95454100 2.49233500
C 6.88364500 -0.99381300 1.50519500
H 6.53611400 -1.87440100 2.06633300
C -3.21161900 0.18290100 2.64015200
C -1.74647500 0.46289400 3.01534700
H -1.11638500 -0.42530300 2.81053100
H -1.66071100 0.72649700 4.08841300
H -1.33956200 1.31119500 2.42571300
C -3.73887200 -1.00274800 3.46907800
H -4.79182400 -1.23558000 3.21119500
H -3.71077600 -0.76178500 4.55106400
H -3.12986000 -1.91230000 3.29660200
C -4.07104800 1.44046900 2.88757800
H -3.70574400 2.28546000 2.27006700
H -4.03129200 1.74307100 3.95432800
H -5.13191900 1.25551300 2.62482500
C -3.87600400 -0.57243100 -2.57453700
C -3.23884200 -1.94663400 -2.86963300
H -3.71709000 -2.75463600 -2.28320500
H -2.15476800 -1.93319900 -2.63108000
H -3.34278300 -2.19722300 -3.94510400
C -5.37687300 -0.61359100 -2.91503000
H -5.52056700 -0.91832400 -3.97110600
H -5.85118500 0.37756300 -2.77497700
H -5.91687200 -1.34824300 -2.28217600
C -3.17920700 0.51468700 -3.41363600
H -3.28793700 0.29915800 -4.49543500
H -2.09287900 0.54901300 -3.18352300
H -3.61205100 1.51350300 -3.20466600
C -4.36397500 -2.63172400 0.27507700
C -3.15143600 -3.25478200 0.64700000

H -2.31405700 -2.63623900 1.01006300
C -3.01924100 -4.64625400 0.53425500
H -2.07189700 -5.12893200 0.81969800
C -4.09318200 -5.42128300 0.06023700
H -3.98812500 -6.51391100 -0.02516400
C -5.30422900 -4.80391000 -0.30221300
H -6.14478700 -5.41112500 -0.67160300
C -5.44232600 -3.41083000 -0.19958400
H -6.38713500 -2.93063600 -0.49803600
C -6.08935700 -0.28848400 0.55692700
C -6.89937400 -0.99695700 1.47389300
H -6.53782500 -1.93288100 1.92661600
C -8.17026900 -0.50537800 1.80982700
H -8.79779300 -1.05857600 2.52520500
C -8.64029200 0.68813700 1.23375000
H -9.63788900 1.07062500 1.49917500
C -7.83995700 1.39058000 0.31445300
H -8.21089600 2.31990500 -0.14418000
C -6.56851600 0.90594500 -0.02238000
H -5.93905200 1.44355700 -0.74539300
Bi -0.04960300 -1.21560200 -0.74798900

**4** (in Singlet) ($C_s$ symmetry)

E = -7480.994014 au

1 1
Ge -0.41742400 -0.36112200 2.07072000
Ge -0.41742400 -0.36112200 -2.07072000
P 0.87289500 0.27572400 4.35148900
P 0.87289500 0.27572400 -4.35148900
O -2.58498400 0.20569500 0.00000000
N 0.11085900 1.17062900 3.18453500
N 0.34271500 -1.17131200 3.69538500
N 0.11085900 1.17062900 -3.18453500
N 0.34271500 -1.17131200 -3.69538500
C -2.36720500 -0.52263700 2.25007700
C -2.96908000 -0.96393500 3.44388400
H -2.32769500 -1.23951700 4.29347000
C -4.36475000 -1.07861700 3.53525800
H -4.82944000 -1.43624100 4.46678700
C -5.17591600 -0.74275700 2.43559000
H -6.26788600 -0.83543500 2.52404500
C -4.61086700 -0.28618500 1.23026600

| | | | |
|---|---|---|---|
| C | -3.20478900 | -0.19775600 | 1.17355700 |
| C | -5.40387200 | 0.17498100 | 0.00000000 |
| C | -6.84267700 | -0.36165800 | 0.00000000 |
| H | -6.86791900 | -1.47000200 | 0.00000000 |
| H | -7.39732500 | 0.00184600 | -0.88736600 |
| H | -7.39732500 | 0.00184600 | 0.88736600 |
| C | -5.43307700 | 1.73205900 | 0.00000000 |
| H | -5.96007900 | 2.10572400 | 0.90163200 |
| H | -5.96007900 | 2.10572400 | -0.90163200 |
| H | -4.40785300 | 2.15304800 | 0.00000000 |
| C | -3.20478900 | -0.19775600 | -1.17355700 |
| C | -4.61086700 | -0.28618500 | -1.23026600 |
| C | -5.17591600 | -0.74275700 | -2.43559000 |
| H | -6.26788600 | -0.83543500 | -2.52404500 |
| C | -4.36475000 | -1.07861700 | -3.53525800 |
| H | -4.82944000 | -1.43624100 | -4.46678700 |
| C | -2.96908000 | -0.96393500 | -3.44388400 |
| H | -2.32769500 | -1.23951700 | -4.29347000 |
| C | -2.36720500 | -0.52263700 | -2.25007700 |
| C | -0.38635600 | 2.56365900 | -3.19170300 |
| C | 0.69626300 | 3.50486300 | -3.75138200 |
| H | 0.34830000 | 4.55716800 | -3.71826500 |
| H | 0.92539200 | 3.26777300 | -4.81000200 |
| H | 1.63379800 | 3.42733800 | -3.16558000 |
| C | -0.67414700 | 2.92461400 | -1.72496600 |
| H | 0.24568200 | 2.82725600 | -1.11558600 |
| H | -1.44105800 | 2.25125900 | -1.28822100 |
| H | -1.05094200 | 3.96381600 | -1.64408300 |
| C | -1.67860600 | 2.66867700 | -4.02843100 |
| H | -2.08926600 | 3.69887400 | -3.99373400 |
| H | -2.44844700 | 1.97158300 | -3.64093000 |
| H | -1.48660900 | 2.41316500 | -5.08983000 |
| C | 0.88602900 | -2.54390900 | -3.87966500 |
| C | 1.00473900 | -2.84487400 | -5.38484700 |
| H | 0.02167700 | -2.76855500 | -5.88984800 |
| H | 1.39644600 | -3.87001200 | -5.54119600 |
| H | 1.70456400 | -2.14341500 | -5.88468600 |
| C | 2.26140900 | -2.71686400 | -3.20133700 |
| H | 3.02208800 | -2.04058700 | -3.63610800 |
| H | 2.62231200 | -3.75901100 | -3.31896400 |
| H | 2.18772200 | -2.50950900 | -2.11305100 |
| C | -0.13214700 | -3.50141500 | -3.23340000 |
| H | -0.23332000 | -3.29010900 | -2.14738700 |
| H | 0.20200900 | -4.55259900 | -3.34249200 |

H -1.13027700 -3.39744000 -3.70360600
C 2.67562200 0.48516300 -4.28444000
C 3.52720700 0.08829500 -5.33943500
H 3.10576300 -0.25289100 -6.29763900
C 4.91905100 0.11724600 -5.16103000
H 5.58235800 -0.19105600 -5.98362700
C 5.46425500 0.53328600 -3.93257400
H 6.55661600 0.55187900 -3.79622400
C 4.61715200 0.92750500 -2.88127600
H 5.04305700 1.25386700 -1.92002900
C 3.22540900 0.90859000 -3.05316800
H 2.55098200 1.20509600 -2.23305200
C 0.34889100 0.57050700 -6.06349700
C -0.81825500 -0.06325600 -6.54167900
H -1.33653000 -0.79070600 -5.90191700
C -1.30860400 0.23842700 -7.81947600
H -2.21700300 -0.26121800 -8.18922100
C -0.64037900 1.17711300 -8.62687000
H -1.02724600 1.41407000 -9.62993400
C 0.52189200 1.81208000 -8.15500400
H 1.04579500 2.54625300 -8.78595400
C 1.01913000 1.51258000 -6.87703600
H 1.92787100 2.01508200 -6.51236500
C -0.38635600 2.56365900 3.19170300
C -0.67414700 2.92461400 1.72496600
H 0.24568200 2.82725600 1.11558600
H -1.05094200 3.96381600 1.64408300
H -1.44105800 2.25125900 1.28822100
C 0.69626300 3.50486300 3.75138200
H 0.92539200 3.26777300 4.81000200
H 0.34830000 4.55716800 3.71826500
H 1.63379800 3.42733800 3.16558000
C -1.67860600 2.66867700 4.02843100
H -2.44844700 1.97158300 3.64093000
H -2.08926600 3.69887400 3.99373400
H -1.48660900 2.41316500 5.08983000
C 0.88602900 -2.54390900 3.87966500
C 2.26140900 -2.71686400 3.20133700
H 3.02208800 -2.04058700 3.63610800
H 2.18772200 -2.50950900 2.11305100
H 2.62231200 -3.75901100 3.31896400
C 1.00473900 -2.84487400 5.38484700
H 1.39644600 -3.87001200 5.54119600
H 0.02167700 -2.76855500 5.88984800

H 1.70456400 -2.14341500 5.88468600
C -0.13214700 -3.50141500 3.23340000
H 0.20200900 -4.55259900 3.34249200
H -0.23332000 -3.29010900 2.14738700
H -1.13027700 -3.39744000 3.70360600
C 2.67562200 0.48516300 4.28444000
C 3.22540900 0.90859000 3.05316800
H 2.55098200 1.20509600 2.23305200
C 4.61715200 0.92750500 2.88127600
H 5.04305700 1.25386700 1.92002900
C 5.46425500 0.53328600 3.93257400
H 6.55661600 0.55187900 3.79622400
C 4.91905100 0.11724600 5.16103000
H 5.58235800 -0.19105600 5.98362700
C 3.52720700 0.08829500 5.33943500
H 3.10576300 -0.25289100 6.29763900
C 0.34889100 0.57050700 6.06349700
C 1.01913000 1.51258000 6.87703600
H 1.92787100 2.01508200 6.51236500
C 0.52189200 1.81208000 8.15500400
H 1.04579500 2.54625300 8.78595400
C -0.64037900 1.17711300 8.62687000
H -1.02724600 1.41407000 9.62993400
C -1.30860400 0.23842700 7.81947600
H -2.21700300 -0.26121800 8.18922100
C -0.81825500 -0.06325600 6.54167900
H -1.33653000 -0.79070600 5.90191700
Bi 1.20990000 -0.76944000 0.00000000

**4** (in triplet state)

E = -7480.946863 au
1 3
Ge 2.12816900 0.33613400 -0.67901000
Ge -1.82207900 0.21715900 0.55051200
P 4.27035300 -0.89371500 0.38426000
P -4.33572100 -0.75296200 0.11149900
O -0.05499700 2.46676600 -0.43681300
N 3.81961600 -0.49403400 -1.17238300
N 2.95913800 -0.09942800 1.03711000
N -3.28453100 0.10454200 -0.84392100
N -3.38633700 -0.61989900 1.47483000
C 2.28888200 2.28827000 -0.78472400

| | | | |
|---|---|---|---|
| C | 3.55340400 | 2.90331800 | -0.86764200 |
| H | 4.44826600 | 2.28303000 | -1.02020400 |
| C | 3.67565000 | 4.29383800 | -0.72173900 |
| H | 4.66622700 | 4.76962300 | -0.78449800 |
| C | 2.53379200 | 5.08107100 | -0.48809700 |
| H | 2.64594600 | 6.16847700 | -0.37107600 |
| C | 1.25237500 | 4.50406600 | -0.40794700 |
| C | 1.16350500 | 3.10342300 | -0.56206500 |
| C | -0.05012000 | 5.29337000 | -0.22490000 |
| C | 0.19305400 | 6.68237800 | 0.38415200 |
| H | 0.66949400 | 6.61518200 | 1.38300100 |
| H | -0.76051600 | 7.23613700 | 0.48796900 |
| H | 0.84026100 | 7.29485700 | -0.27403300 |
| C | -0.71533400 | 5.45276400 | -1.62430600 |
| H | -0.05609500 | 6.03388600 | -2.30134600 |
| H | -1.68550100 | 5.98169600 | -1.52812100 |
| H | -0.90690800 | 4.46610200 | -2.09156800 |
| C | -0.99403400 | 3.04418700 | 0.41825900 |
| C | -0.98365700 | 4.43460200 | 0.63888400 |
| C | -1.90918500 | 4.93753800 | 1.57466400 |
| H | -1.93293200 | 6.01486000 | 1.79275500 |
| C | -2.81366300 | 4.08135800 | 2.22932700 |
| H | -3.52299200 | 4.49668500 | 2.96200000 |
| C | -2.82457600 | 2.70729200 | 1.93702100 |
| H | -3.54070300 | 2.03754700 | 2.43875000 |
| C | -1.91418100 | 2.16655300 | 1.00980700 |
| C | -3.59620100 | 0.95055400 | -2.02333600 |
| C | -4.50679500 | 0.17570800 | -2.99589700 |
| H | -4.70703500 | 0.78181100 | -3.90266500 |
| H | -5.48755700 | -0.05597400 | -2.53486800 |
| H | -4.03557800 | -0.77682800 | -3.31061900 |
| C | -2.27265200 | 1.27690900 | -2.73727100 |
| H | -1.79362100 | 0.35264800 | -3.12511100 |
| H | -1.55469200 | 1.78043300 | -2.05987600 |
| H | -2.45878200 | 1.94971500 | -3.59874800 |
| C | -4.27913600 | 2.26729500 | -1.59056900 |
| H | -4.48001600 | 2.91194900 | -2.47094700 |
| H | -3.63446900 | 2.82730300 | -0.88558600 |
| H | -5.24569200 | 2.06837700 | -1.08726900 |
| C | -3.25551600 | -1.53274600 | 2.63543900 |
| C | -4.64506000 | -1.83587100 | 3.22394700 |
| H | -5.16957100 | -0.90144100 | 3.50614300 |
| H | -4.55247300 | -2.47165400 | 4.12784400 |
| H | -5.27929000 | -2.38237100 | 2.49753400 |

C -2.53861300 -2.84103300 2.23668800
H -3.13316100 -3.42147300 1.50460900
H -2.36310200 -3.48403900 3.12358000
H -1.55509800 -2.61153100 1.77524500
C -2.40669900 -0.77648400 3.67546500
H -1.41535000 -0.51304400 3.24765900
H -2.23671300 -1.40173500 4.57506900
H -2.90572900 0.16332000 3.98529500
C -4.55615000 -2.45222600 -0.51092200
C -5.58884800 -3.30197800 -0.05588100
H -6.36834900 -2.91714600 0.61959900
C -5.62235300 -4.64463800 -0.46418300
H -6.42907400 -5.30383800 -0.10850400
C -4.62890500 -5.14668300 -1.32447800
H -4.65892100 -6.20053600 -1.64174300
C -3.60374100 -4.30145300 -1.78613300
H -2.83146500 -4.68937000 -2.46826800
C -3.56877500 -2.95859900 -1.38406200
H -2.77907000 -2.27834800 -1.73988000
C -6.00274200 -0.06744500 0.35693100
C -6.17538800 0.91741900 1.35363700
H -5.31747800 1.19759200 1.98112300
C -7.42435100 1.52966600 1.52737400
H -7.55435600 2.29738100 2.30551800
C -8.50864800 1.16273200 0.70931200
H -9.48925800 1.64397700 0.84674500
C -8.34237900 0.17993100 -0.28251200
H -9.19069000 -0.10916500 -0.92171300
C -7.09386700 -0.43604900 -0.46182800
H -6.97042700 -1.20405400 -1.24057200
C 4.54245200 -0.65385800 -2.45959400
C 3.50880700 -0.44360600 -3.57969400
H 2.70020400 -1.20105900 -3.52332400
H 3.99501700 -0.51833900 -4.57297500
H 3.04660600 0.56407500 -3.50567600
C 5.13924400 -2.07047000 -2.56095500
H 5.86995200 -2.26109400 -1.74742700
H 5.67591400 -2.19233500 -3.52317000
H 4.35145900 -2.84622200 -2.50183200
C 5.67209900 0.39000300 -2.59488300
H 5.26000000 1.41750900 -2.61834600
H 6.23416000 0.23216100 -3.53779800
H 6.38991700 0.31956100 -1.75397700
C 2.32060600 -0.22325000 2.36876500

C 1.49377200 -1.52168500 2.47514600
H 2.13327300 -2.42104000 2.38177400
H 0.72632400 -1.55567600 1.67303400
H 0.96776100 -1.57282000 3.45024300
C 3.40238600 -0.19756100 3.46413200
H 2.93197600 -0.26852800 4.46537100
H 3.99486600 0.73734900 3.42394300
H 4.09799000 -1.05620100 3.36410300
C 1.39460800 0.99738600 2.51155400
H 0.89220100 0.99903400 3.49922700
H 0.59634500 0.97375900 1.73882800
H 1.95959500 1.94377600 2.39603000
C 4.26214000 -2.67378400 0.71969800
C 3.32801900 -3.46042900 0.00651400
H 2.73385400 -3.00103100 -0.80082500
C 3.14753800 -4.80804100 0.34310300
H 2.41935800 -5.41743300 -0.21385500
C 3.89489500 -5.37989100 1.39012700
H 3.75438800 -6.44025900 1.65065900
C 4.81939600 -4.59885700 2.10577300
H 5.39944000 -5.04438400 2.92836900
C 5.00286900 -3.24570400 1.77779500
H 5.71486600 -2.63484200 2.35344900
C 5.86068000 -0.19454100 0.88407000
C 7.07276600 -0.90297900 0.71910400
H 7.06531800 -1.95058700 0.38076600
C 8.29408500 -0.26047900 0.97487100
H 9.23836300 -0.81152600 0.84723000
C 8.31167900 1.08400100 1.38821100
H 9.27212300 1.58396200 1.58697100
C 7.10520800 1.79114900 1.54697200
H 7.12013800 2.84325200 1.87051000
C 5.88019000 1.15805700 1.29480800
H 4.92942500 1.70168800 1.40394500
Bi 0.05273400 -1.22045000 -1.33054800

**5** (in doublet state) ($C_1$ symmetry)

E = -7480.720509 au

2 2
Ge -2.10437900 0.52711200 0.06317500
Ge 2.12807900 0.32992300 -0.53352000
P -4.42736400 -0.80810300 0.06101700

P 4.33581800 -0.94970600 0.25744800
O 0.25485700 2.52516200 0.16373900
N -3.32530700 -0.37103100 1.24617500
N -3.56162500 0.03536700 -1.10893100
N 3.15798600 -0.06148000 1.05001000
N 3.61953100 -0.64804500 -1.24177400
C -2.11375200 2.46460500 0.28010500
C -3.34891500 3.13987400 0.36452700
H -4.28303300 2.56617400 0.44696200
C -3.38380600 4.54095300 0.32843600
H -4.34624000 5.07030800 0.39203100
C -2.18997200 5.27480600 0.19992500
H -2.23976200 6.37207000 0.15671100
C -0.93711400 4.63575300 0.14452400
C -0.93596700 3.22686700 0.20488400
C 0.40953500 5.37110500 0.09983600
C 0.28052600 6.77927000 -0.50275800
H -0.10133900 6.74843000 -1.54285400
H 1.25894600 7.29764000 -0.50258700
H -0.39968600 7.40808900 0.10367100
C 0.94377900 5.47493200 1.56025000
H 0.24625200 6.07138500 2.18249200
H 1.93776300 5.96625100 1.57071600
H 1.04865900 4.47289700 2.02330700
C 1.30489500 3.09739100 -0.53410500
C 1.39413300 4.49366600 -0.68521800
C 2.46641400 4.97719100 -1.46007500
H 2.57410400 6.05942500 -1.62046000
C 3.41844900 4.10509000 -2.02091100
H 4.24239700 4.51399700 -2.62463000
C 3.33073600 2.72197500 -1.79801700
H 4.08438400 2.04007800 -2.21930000
C 2.25983400 2.20406000 -1.04422300
C 3.18294200 0.62608800 2.36789600
C 3.90316800 -0.25895900 3.40012900
H 3.89338000 0.23211900 4.39332800
H 4.96552600 -0.41549400 3.12531800
H 3.41468900 -1.24842900 3.49885000
C 1.71445300 0.82382100 2.78549600
H 1.19918100 -0.15471200 2.87887100
H 1.16497900 1.44118400 2.04398000
H 1.65471100 1.34184300 3.76327000
C 3.89291700 1.98909300 2.23714900
H 3.89317300 2.52714600 3.20657800

H 3.38654600 2.62804400 1.48584200
H 4.94611400 1.85559600 1.91892400
C 3.70396700 -1.42969500 -2.50620200
C 5.18537400 -1.64421500 -2.86418900
H 5.71647300 -0.67734600 -2.96462700
H 5.27537700 -2.19377800 -3.82200600
H 5.70426100 -2.24475300 -2.08956100
C 2.98012000 -2.78625000 -2.38017000
H 3.43696300 -3.42838300 -1.60340400
H 3.01290200 -3.33675800 -3.34171600
H 1.90987800 -2.63677100 -2.11835000
C 3.01534000 -0.57831000 -3.58668400
H 1.95479200 -0.37475100 -3.31627400
H 3.00944100 -1.11134800 -4.55768700
H 3.52907000 0.39282100 -3.72648600
C 4.31654000 -2.68793300 0.75603800
C 5.37260000 -3.56758000 0.42546900
H 6.28912200 -3.18859000 -0.05186000
C 5.24813200 -4.93805600 0.70211200
H 6.07025400 -5.62351900 0.44646800
C 4.07775300 -5.43331400 1.30478400
H 3.98692900 -6.50854400 1.52235300
C 3.02714700 -4.55791700 1.63714600
H 2.11742000 -4.94678600 2.11955600
C 3.14277700 -3.18790700 1.36393400
H 2.33187900 -2.48942600 1.62343500
C 6.02186900 -0.32004500 0.37816100
C 6.37714100 0.79144100 -0.41944600
H 5.65160900 1.19640400 -1.13856300
C 7.64541100 1.37075400 -0.28136700
H 7.92549600 2.23263000 -0.90570300
C 8.55943500 0.85023400 0.65398100
H 9.55551500 1.30664200 0.75994300
C 8.20510100 -0.25013600 1.45490200
H 8.92039300 -0.65350500 2.18747400
C 6.93797900 -0.83857000 1.32295500
H 6.66499000 -1.69557600 1.95699200
C -3.44031700 -0.32144900 2.72701600
C -2.00445500 -0.17402800 3.26108000
H -1.38025400 -1.04242600 2.96294700
H -2.00193000 -0.10771700 4.36688600
H -1.52925100 0.75355900 2.87320000
C -4.06345500 -1.62845200 3.24658900
H -5.09023400 -1.76843100 2.85206700

H -4.13926900 -1.60411500 4.35176600
H -3.45782100 -2.51038200 2.95932700
C -4.29198900 0.88982300 3.15800200
H -3.83694600 1.83608200 2.80374300
H -4.37247600 0.93931500 4.26262500
H -5.31807200 0.82033200 2.74510100
C -3.53057600 -0.11715600 -2.58742300
C -2.85902800 -1.44084700 -3.00768200
H -3.41221200 -2.32210700 -2.62945600
H -1.81934000 -1.49443100 -2.61434600
H -2.80216500 -1.51831100 -4.11201400
C -4.96637200 -0.06572600 -3.14191000
H -4.95470400 -0.18364500 -4.24345800
H -5.45703300 0.89749400 -2.90318300
H -5.58812800 -0.88596700 -2.72831100
C -2.71550000 1.07785300 -3.11273000
H -2.64646300 1.05146100 -4.21805000
H -1.67532900 1.05191100 -2.71613200
H -3.17532100 2.03859000 -2.80731100
C -4.48900000 -2.59635200 -0.19967500
C -3.37419200 -3.37103100 0.19188400
H -2.56242900 -2.89672700 0.76516900
C -3.31742000 -4.73169500 -0.13849900
H -2.45297100 -5.33752800 0.17319100
C -4.37085100 -5.32480000 -0.85962100
H -4.32654100 -6.39415700 -1.11689000
C -5.48501000 -4.55803200 -1.24506800
H -6.31047800 -5.02525900 -1.80311900
C -5.54915400 -3.19418400 -0.91813500
H -6.42207000 -2.59794000 -1.22546100
C -6.11595100 -0.21097500 0.28229500
C -7.06191400 -0.97930800 1.00062800
H -6.80916000 -1.99053100 1.35488400
C -8.33421000 -0.44787500 1.26034400
H -9.07111000 -1.04374600 1.81978400
C -8.66811200 0.83974700 0.80265900
H -9.66889900 1.25067000 1.00576100
C -7.73131900 1.60050100 0.07893500
H -8.00051400 2.60193500 -0.28971800
C -6.45534600 1.08054800 -0.17996500
H -5.71852600 1.66146700 -0.75322800
Bi -0.06952600 -1.25030000 -0.29516800

**5** (in doublet state) ($C_s$ symmetry)

E = -7480.719800 au

2 2
Ge -0.51188100 -0.31134200 2.15971100
Ge -0.51188100 -0.31134200 -2.15971100
P 0.93611700 0.19294900 4.35324800
P 0.93611700 0.19294900 -4.35324800
O -2.63500300 0.20257000 0.00000000
N 0.17340600 1.15093500 3.21107800
N 0.31349200 -1.21070200 3.65407700
N 0.17340600 1.15093500 -3.21107800
N 0.31349200 -1.21070200 -3.65407700
C -2.44771400 -0.46600500 2.27612800
C -3.05523300 -0.86513500 3.48400700
H -2.42648500 -1.10256700 4.35352700
C -4.45051900 -0.97146600 3.56516000
H -4.92585800 -1.29356200 4.50364000
C -5.24813800 -0.66596500 2.44693700
H -6.34099200 -0.74830300 2.53094700
C -4.67588500 -0.24569600 1.23191700
C -3.26922400 -0.16787600 1.17683200
C -5.47165800 0.20341400 0.00000000
C -6.90569200 -0.34776300 0.00000000
H -6.92243300 -1.45610800 0.00000000
H -7.46510900 0.01244200 -0.88511600
H -7.46510900 0.01244200 0.88511600
C -5.51467700 1.76099700 0.00000000
H -6.04677200 2.12878300 0.90053600
H -6.04677200 2.12878300 -0.90053600
H -4.49439200 2.19452600 0.00000000
C -3.26922400 -0.16787600 -1.17683200
C -4.67588500 -0.24569600 -1.23191700
C -5.24813800 -0.66596500 -2.44693700
H -6.34099200 -0.74830300 -2.53094700
C -4.45051900 -0.97146600 -3.56516000
H -4.92585800 -1.29356200 -4.50364000
C -3.05523300 -0.86513500 -3.48400700
H -2.42648500 -1.10256700 -4.35352700
C -2.44771400 -0.46600500 -2.27612800
C -0.29285900 2.56059500 -3.26927500
C 0.82761100 3.45545000 -3.82593300
H 0.50059400 4.51408500 -3.84226900
H 1.08241200 3.17692800 -4.86820400

| | | | |
|---|---|---|---|
| H | 1.74599200 | 3.38470200 | -3.20998800 |
| C | -0.61111300 | 2.96014700 | -1.81818600 |
| H | 0.29643500 | 2.88496600 | -1.18464400 |
| H | -1.39713500 | 2.30580500 | -1.38245600 |
| H | -0.98424200 | 4.00210800 | -1.77031400 |
| C | -1.55934500 | 2.66703800 | -4.14277200 |
| H | -1.93900500 | 3.70863100 | -4.15823200 |
| H | -2.36229500 | 2.01019800 | -3.75197700 |
| H | -1.34589700 | 2.36724100 | -5.18800900 |
| C | 0.80877100 | -2.61318200 | -3.72098700 |
| C | 0.98469400 | -3.02120300 | -5.19490600 |
| H | 0.02932200 | -2.94735000 | -5.74979500 |
| H | 1.34428200 | -4.06690800 | -5.26267200 |
| H | 1.73299300 | -2.38101400 | -5.70523100 |
| C | 2.14333200 | -2.78069600 | -2.96569300 |
| H | 2.94697100 | -2.15751800 | -3.40258200 |
| H | 2.47663800 | -3.83753600 | -2.99121100 |
| H | 2.02813700 | -2.49428100 | -1.89716100 |
| C | -0.27793700 | -3.47699600 | -3.05783400 |
| H | -0.42661000 | -3.17985900 | -1.99531800 |
| H | 0.01439500 | -4.54546600 | -3.06235500 |
| H | -1.24840400 | -3.37441100 | -3.58213300 |
| C | 2.73676500 | 0.34328400 | -4.25525800 |
| C | 3.57334100 | -0.16571700 | -5.27459600 |
| H | 3.14158400 | -0.56213200 | -6.20646700 |
| C | 4.96545400 | -0.16796500 | -5.09529400 |
| H | 5.61782800 | -0.56307400 | -5.88864600 |
| C | 5.52493700 | 0.33515100 | -3.90681500 |
| H | 6.61749300 | 0.33345300 | -3.77265200 |
| C | 4.69329100 | 0.85014200 | -2.89517600 |
| H | 5.13495800 | 1.25819800 | -1.97328000 |
| C | 3.30186300 | 0.85592300 | -3.06624600 |
| H | 2.63849100 | 1.26716100 | -2.28944300 |
| C | 0.44831800 | 0.43229000 | -6.07387200 |
| C | -0.70782400 | -0.21723100 | -6.56048900 |
| H | -1.24152700 | -0.92895000 | -5.91518700 |
| C | -1.15681000 | 0.03879100 | -7.86299300 |
| H | -2.05222800 | -0.47402800 | -8.24544900 |
| C | -0.46022700 | 0.94747900 | -8.68129300 |
| H | -0.81545800 | 1.14848100 | -9.70359300 |
| C | 0.69191500 | 1.59540600 | -8.20052300 |
| H | 1.23792400 | 2.30164200 | -8.84403600 |
| C | 1.15207600 | 1.34039400 | -6.89985800 |
| H | 2.05725300 | 1.84657900 | -6.53114700 |

C -0.29285900 2.56059500 3.26927500
C -0.61111300 2.96014700 1.81818600
H 0.29643500 2.88496600 1.18464400
H -0.98424200 4.00210800 1.77031400
H -1.39713500 2.30580500 1.38245600
C 0.82761100 3.45545000 3.82593300
H 1.08241200 3.17692800 4.86820400
H 0.50059400 4.51408500 3.84226900
H 1.74599200 3.38470200 3.20998800
C -1.55934500 2.66703800 4.14277200
H -2.36229500 2.01019800 3.75197700
H -1.93900500 3.70863100 4.15823200
H -1.34589700 2.36724100 5.18800900
C 0.80877100 -2.61318200 3.72098700
C 2.14333200 -2.78069600 2.96569300
H 2.94697100 -2.15751800 3.40258200
H 2.02813700 -2.49428100 1.89716100
H 2.47663800 -3.83753600 2.99121100
C 0.98469400 -3.02120300 5.19490600
H 1.34428200 -4.06690800 5.26267200
H 0.02932200 -2.94735000 5.74979500
H 1.73299300 -2.38101400 5.70523100
C -0.27793700 -3.47699600 3.05783400
H 0.01439500 -4.54546600 3.06235500
H -0.42661000 -3.17985900 1.99531800
H -1.24840400 -3.37441100 3.58213300
C 2.73676500 0.34328400 4.25525800
C 3.30186300 0.85592300 3.06624600
H 2.63849100 1.26716100 2.28944300
C 4.69329100 0.85014200 2.89517600
H 5.13495800 1.25819800 1.97328000
C 5.52493700 0.33515100 3.90681500
H 6.61749300 0.33345300 3.77265200
C 4.96545400 -0.16796500 5.09529400
H 5.61782800 -0.56307400 5.88864600
C 3.57334100 -0.16571700 5.27459600
H 3.14158400 -0.56213200 6.20646700
C 0.44831800 0.43229000 6.07387200
C 1.15207600 1.34039400 6.89985800
H 2.05725300 1.84657900 6.53114700
C 0.69191500 1.59540600 8.20052300
H 1.23792400 2.30164200 8.84403600
C -0.46022700 0.94747900 8.68129300
H -0.81545800 1.14848100 9.70359300

C -1.15681000 0.03879100 7.86299300
H -2.05222800 -0.47402800 8.24544900
C -0.70782400 -0.21723100 6.56048900
H -1.24152700 -0.92895000 5.91518700
Bi 1.14196900 -0.43080800 0.00000000

**5** (in quartet state)

E = -7480.644573 au

2 4
Ge 2.07281600 0.30613600 -0.65832600
Ge -2.03701900 0.44089800 0.68193700
P 4.23491900 -0.80390300 0.47385800
P -4.33616000 -0.82608200 0.16602500
O -0.16170800 2.46862000 -0.33564800
N 3.78915900 -0.45034300 -1.10669900
N 2.83731600 -0.09353200 1.06782000
N -3.26251900 0.07093900 -0.77803900
N -3.44991200 -0.52575300 1.57018900
C 2.15739300 2.25476300 -0.83395000
C 3.41566700 2.84900500 -1.05829400
H 4.29398500 2.21087200 -1.23455900
C 3.55170100 4.24466500 -1.02812300
H 4.53570500 4.70630800 -1.19895300
C 2.43085400 5.05429000 -0.77959100
H 2.55301600 6.14634100 -0.76221800
C 1.15492000 4.49789000 -0.56496600
C 1.05298300 3.09090400 -0.59316200
C -0.12073000 5.33170200 -0.38892300
C 0.17543900 6.74581600 0.13625900
H 0.67738100 6.72208500 1.12423000
H -0.75829300 7.33338000 0.23000100
H 0.81789600 7.30355700 -0.57211600
C -0.81964000 5.42990600 -1.77847300
H -0.16145400 5.95128600 -2.50262000
H -1.76985100 5.99482800 -1.69103800
H -1.05094600 4.42479700 -2.18554600
C -1.08533900 3.15139100 0.44203700
C -1.06004600 4.55582800 0.54126300
C -1.99110400 5.14768400 1.41771600
H -1.99576200 6.23983800 1.54195100
C -2.92798000 4.37691500 2.13021100

H -3.63994700 4.87130700 2.80815900
C -2.97074700 2.98331600 1.96247300
H -3.71483600 2.37903600 2.50260600
C -2.04814900 2.36757400 1.09797900
C -3.54264600 0.80929700 -2.04449000
C -4.34719300 -0.09001300 -2.99898500
H -4.52639400 0.43806900 -3.95641700
H -5.34014400 -0.34554600 -2.57904000
H -3.80537800 -1.03096200 -3.21958900
C -2.18771100 1.15052600 -2.68607800
H -1.63782900 0.22396500 -2.95755300
H -1.54820400 1.74637000 -2.00502200
H -2.34111400 1.73821700 -3.61256500
C -4.31469100 2.11134100 -1.73959600
H -4.48481200 2.68470000 -2.67292900
H -3.74451100 2.75172000 -1.03745700
H -5.30270600 1.89637200 -1.28830300
C -3.31229900 -1.35059800 2.80101800
C -4.71212800 -1.64414900 3.37018800
H -5.26089100 -0.70766300 3.59089200
H -4.62845800 -2.22840200 4.30791200
H -5.31756000 -2.24388700 2.66094200
C -2.55822500 -2.66241400 2.50224200
H -3.13039900 -3.31611300 1.81615000
H -2.37450200 -3.22904700 3.43710300
H -1.57635900 -2.44349100 2.03270600
C -2.50379200 -0.49424800 3.79048500
H -1.50401700 -0.24707300 3.36976900
H -2.33874600 -1.04226800 4.73890000
H -3.02763400 0.45443700 4.02162600
C -4.43610600 -2.55087600 -0.35728600
C -5.47085800 -3.40022500 0.09854400
H -6.29238600 -3.00327500 0.71418300
C -5.44754900 -4.76248600 -0.23779800
H -6.25225800 -5.42529200 0.11457600
C -4.40210000 -5.27833900 -1.02512900
H -4.39181900 -6.34698400 -1.28897100
C -3.37526800 -4.43194500 -1.48329800
H -2.56518800 -4.83621200 -2.10938200
C -3.38964700 -3.07088600 -1.15054300
H -2.59632600 -2.39265100 -1.50183300
C -6.00830600 -0.15723700 0.29918400
C -6.23887800 0.89331700 1.21600900
H -5.42108000 1.23080800 1.86788500

| | | | |
|---|---|---|---|
| C | -7.50439600 | 1.49097200 | 1.28317200 |
| H | -7.68847600 | 2.30553800 | 1.99987700 |
| C | -8.53898800 | 1.04710600 | 0.43840400 |
| H | -9.53237500 | 1.51806000 | 0.49387100 |
| C | -8.31074400 | 0.00112700 | -0.47374200 |
| H | -9.12233800 | -0.34683500 | -1.13043000 |
| C | -7.04730000 | -0.60466400 | -0.54937700 |
| H | -6.87560200 | -1.42259400 | -1.26529000 |
| C | 4.48758900 | -0.62953900 | -2.41082500 |
| C | 3.61114200 | 0.04913500 | -3.47855100 |
| H | 2.60237900 | -0.41802400 | -3.51888700 |
| H | 4.06663100 | -0.06909000 | -4.48113700 |
| H | 3.49224700 | 1.13381700 | -3.28415600 |
| C | 4.63795500 | -2.12558800 | -2.74281900 |
| H | 5.23610800 | -2.65491200 | -1.97409700 |
| H | 5.15051600 | -2.25596600 | -3.71690700 |
| H | 3.64473900 | -2.61556700 | -2.81019500 |
| C | 5.87705200 | 0.03854200 | -2.37895000 |
| H | 5.80926500 | 1.10574500 | -2.08829600 |
| H | 6.34815600 | -0.01644400 | -3.38034400 |
| H | 6.55547500 | -0.46522200 | -1.66382400 |
| C | 2.17339900 | -0.22090500 | 2.38586000 |
| C | 1.32552700 | -1.50831500 | 2.43680300 |
| H | 1.95267900 | -2.41114700 | 2.29918400 |
| H | 0.56171200 | -1.49431500 | 1.62799900 |
| H | 0.79500500 | -1.60002700 | 3.40619800 |
| C | 3.22976700 | -0.23663100 | 3.50614700 |
| H | 2.73258000 | -0.29871900 | 4.49437400 |
| H | 3.85117400 | 0.67950100 | 3.48775100 |
| H | 3.89738700 | -1.11703700 | 3.41846800 |
| C | 1.26962600 | 1.01420600 | 2.53499400 |
| H | 0.73503900 | 1.00047400 | 3.50548100 |
| H | 0.50210100 | 1.03507100 | 1.73095700 |
| H | 1.85634100 | 1.95171600 | 2.46665000 |
| C | 4.36496800 | -2.56721000 | 0.86039900 |
| C | 3.52669700 | -3.46186400 | 0.15643600 |
| H | 2.91236300 | -3.09052700 | -0.67841900 |
| C | 3.47885800 | -4.81312200 | 0.52171900 |
| H | 2.83226400 | -5.50878500 | -0.03447400 |
| C | 4.26292500 | -5.28085100 | 1.59339800 |
| H | 4.22906700 | -6.34419400 | 1.87606700 |
| C | 5.08983100 | -4.39324000 | 2.30358000 |
| H | 5.69919200 | -4.75804900 | 3.14423000 |
| C | 5.14218300 | -3.03677000 | 1.94446300 |

H 5.78213500 -2.34739400 2.51510600
C 5.72835500 0.05238200 1.01280400
C 7.00439600 -0.55303200 0.94438800
H 7.11164300 -1.60506400 0.63882400
C 8.14630800 0.20138500 1.25369000
H 9.14077700 -0.26723600 1.20326300
C 8.02057000 1.55271300 1.62400900
H 8.91974300 2.13908400 1.86776000
C 6.75129100 2.15750200 1.68288000
H 6.65656800 3.21508300 1.97254100
C 5.60354200 1.41322600 1.37560700
H 4.60584900 1.87647300 1.41176900
Bi 0.30650200 -1.54351800 -1.56948600

**6 (doublet state)**

**E = -7481.122262 au**

0 2
Ge -0.42079000 -0.37087400 2.08453900
Ge -0.42079000 -0.37087400 -2.08453900
P 0.89251900 0.29893900 4.37050500
P 0.89251900 0.29893900 -4.37050500
O -2.59620000 0.20579800 0.00000000
N 0.10163700 1.16406100 3.17825600
N 0.33348600 -1.15789300 3.70864600
N 0.10163700 1.16406100 -3.17825600
N 0.33348600 -1.15789300 -3.70864600
C -2.37552500 -0.51888800 2.25229900
C -2.98384400 -0.95595200 3.44542800
H -2.34183900 -1.23385600 4.29451300
C -4.38003600 -1.06572600 3.53748400
H -4.84490700 -1.41905000 4.47113100
C -5.19102200 -0.73036500 2.43720900
H -6.28374900 -0.81805700 2.52453100
C -4.62076500 -0.27934400 1.23142200
C -3.21442400 -0.19454500 1.17491700
C -5.40923700 0.18647500 0.00000000
C -6.85206900 -0.33906000 0.00000000
H -6.88375600 -1.44740200 0.00000000
H -7.40341400 0.02732400 -0.88869000
H -7.40341400 0.02732400 0.88869000
C -5.42880700 1.74339900 0.00000000

H -5.95165100 2.12143200 0.90279400
H -5.95165100 2.12143200 -0.90279400
H -4.39977200 2.15455500 0.00000000
C -3.21442400 -0.19454500 -1.17491700
C -4.62076500 -0.27934400 -1.23142200
C -5.19102200 -0.73036500 -2.43720900
H -6.28374900 -0.81805700 -2.52453100
C -4.38003600 -1.06572600 -3.53748400
H -4.84490700 -1.41905000 -4.47113100
C -2.98384400 -0.95595200 -3.44542800
H -2.34183900 -1.23385600 -4.29451300
C -2.37552500 -0.51888800 -2.25229900
C -0.38159800 2.55671700 -3.18307900
C 0.69124600 3.48652900 -3.78324000
H 0.35768700 4.54378800 -3.73376500
H 0.87733400 3.24485600 -4.84950000
H 1.64894500 3.39305200 -3.23313300
C -0.63136200 2.94048900 -1.71495000
H 0.30155600 2.84058700 -1.12613200
H -1.39037500 2.27601200 -1.25223600
H -0.99770400 3.98451500 -1.63644000
C -1.69386200 2.66796900 -3.99000100
H -2.09027700 3.70505500 -3.96665000
H -2.46273700 1.98862500 -3.57000400
H -1.52902100 2.38438400 -5.04901300
C 0.87951500 -2.52049200 -3.91738200
C 1.01651400 -2.78711000 -5.42877800
H 0.04385300 -2.66653600 -5.94584800
H 1.38454100 -3.81862500 -5.60565700
H 1.73883900 -2.08671600 -5.89639500
C 2.24975700 -2.71094600 -3.23037900
H 3.00797700 -2.01264800 -3.63377000
H 2.62002800 -3.74681800 -3.37873100
H 2.16255600 -2.53845300 -2.13693300
C -0.13975400 -3.50228400 -3.30880300
H -0.25348700 -3.31889900 -2.21920100
H 0.19995000 -4.54943600 -3.44572100
H -1.13370200 -3.38658700 -3.78586600
C 2.67975200 0.51946500 -4.24634000
C 3.58197900 0.15128600 -5.27692000
H 3.19781400 -0.16797200 -6.25803000
C 4.96313200 0.17015300 -5.04432500
H 5.65488600 -0.11471200 -5.85271700
C 5.46837400 0.53329200 -3.77778800

| | | | |
|---|---|---|---|
| H | 6.55446100 | 0.53692300 | -3.59711400 |
| C | 4.57687800 | 0.88593600 | -2.74744400 |
| H | 4.96120400 | 1.16415600 | -1.75334500 |
| C | 3.19423400 | 0.88869400 | -2.97475500 |
| H | 2.49307400 | 1.15754600 | -2.16790900 |
| C | 0.39178500 | 0.58458600 | -6.06895300 |
| C | -0.83544000 | 0.03012800 | -6.52765400 |
| H | -1.40180000 | -0.63686300 | -5.86346800 |
| C | -1.31769200 | 0.33442000 | -7.80489600 |
| H | -2.26901500 | -0.10532000 | -8.14417400 |
| C | -0.59478700 | 1.19389200 | -8.65701300 |
| H | -0.97642300 | 1.42973600 | -9.66212700 |
| C | 0.61871800 | 1.75681700 | -8.20641000 |
| H | 1.18540000 | 2.43933800 | -8.85936400 |
| C | 1.10574800 | 1.46728100 | -6.92690500 |
| H | 2.03894700 | 1.93548500 | -6.57932800 |
| C | -0.38159800 | 2.55671700 | 3.18307900 |
| C | -0.63136200 | 2.94048900 | 1.71495000 |
| H | 0.30155600 | 2.84058700 | 1.12613200 |
| H | -0.99770400 | 3.98451500 | 1.63644000 |
| H | -1.39037500 | 2.27601200 | 1.25223600 |
| C | 0.69124600 | 3.48652900 | 3.78324000 |
| H | 0.87733400 | 3.24485600 | 4.84950000 |
| H | 0.35768700 | 4.54378800 | 3.73376500 |
| H | 1.64894500 | 3.39305200 | 3.23313300 |
| C | -1.69386200 | 2.66796900 | 3.99000100 |
| H | -2.46273700 | 1.98862500 | 3.57000400 |
| H | -2.09027700 | 3.70505500 | 3.96665000 |
| H | -1.52902100 | 2.38438400 | 5.04901300 |
| C | 0.87951500 | -2.52049200 | 3.91738200 |
| C | 2.24975700 | -2.71094600 | 3.23037900 |
| H | 3.00797700 | -2.01264800 | 3.63377000 |
| H | 2.16255600 | -2.53845300 | 2.13693300 |
| H | 2.62002800 | -3.74681800 | 3.37873100 |
| C | 1.01651400 | -2.78711000 | 5.42877800 |
| H | 1.38454100 | -3.81862500 | 5.60565700 |
| H | 0.04385300 | -2.66653600 | 5.94584800 |
| H | 1.73883900 | -2.08671600 | 5.89639500 |
| C | -0.13975400 | -3.50228400 | 3.30880300 |
| H | 0.19995000 | -4.54943600 | 3.44572100 |
| H | -0.25348700 | -3.31889900 | 2.21920100 |
| H | -1.13370200 | -3.38658700 | 3.78586600 |
| C | 2.67975200 | 0.51946500 | 4.24634000 |
| C | 3.19423400 | 0.88869400 | 2.97475500 |

H 2.49307400 1.15754600 2.16790900
C 4.57687800 0.88593600 2.74744400
H 4.96120400 1.16415600 1.75334500
C 5.46837400 0.53329200 3.77778800
H 6.55446100 0.53692300 3.59711400
C 4.96313200 0.17015300 5.04432500
H 5.65488600 -0.11471200 5.85271700
C 3.58197900 0.15128600 5.27692000
H 3.19781400 -0.16797200 6.25803000
C 0.39178500 0.58458600 6.06895300
C 1.10574800 1.46728100 6.92690500
H 2.03894700 1.93548500 6.57932800
C 0.61871800 1.75681700 8.20641000
H 1.18540000 2.43933800 8.85936400
C -0.59478700 1.19389200 8.65701300
H -0.97642300 1.42973600 9.66212700
C -1.31769200 0.33442000 7.80489600
H -2.26901500 -0.10532000 8.14417400
C -0.83544000 0.03012800 6.52765400
H -1.40180000 -0.63686300 5.86346800
Bi 1.17312000 -0.83307900 0.00000000

**6 (quartet state)**

**E = -7481.075511 au**

0 4
Ge 2.16868400 0.38517400 -0.66456000
Ge -1.81076700 0.23150600 0.52700400
P 4.28203800 -0.95306600 0.39950400
P -4.33198600 -0.79856400 0.12576600
O -0.04872100 2.51372900 -0.45693500
N 3.82893200 -0.46135900 -1.15953700
N 2.95915500 -0.10609200 1.03322400
N -3.31143000 0.11221700 -0.82790300
N -3.33956500 -0.64633200 1.46949400
C 2.30899200 2.34170300 -0.72783500
C 3.57096100 2.96975300 -0.76478500
H 4.47477600 2.35394000 -0.88117700
C 3.68073000 4.36031400 -0.61158100
H 4.67216200 4.83899400 -0.63438400
C 2.52839000 5.14365900 -0.41633900
H 2.62976000 6.23131100 -0.29073700
C 1.24927300 4.55457600 -0.38115500

C 1.17393900 3.15471000 -0.54252400
C -0.06548700 5.33030200 -0.22842900
C 0.14963200 6.72335700 0.38174200
H 0.60694900 6.66200000 1.38997200
H -0.81258700 7.26625400 0.46622400
H 0.80561200 7.34011100 -0.26426000
C -0.70534300 5.48202800 -1.63993100
H -0.03827900 6.06817200 -2.30561800
H -1.68400500 5.99957300 -1.56434700
H -0.87611300 4.49190800 -2.10757800
C -1.00068600 3.07396500 0.39120600
C -1.00506100 4.46351000 0.62056300
C -1.94522800 4.95700300 1.54780500
H -1.97892100 6.03332100 1.77083900
C -2.85034900 4.08921600 2.18633800
H -3.57321200 4.49389300 2.91249200
C -2.84610500 2.71671700 1.88629800
H -3.56332300 2.03804800 2.37455400
C -1.92167300 2.18478200 0.96649200
C -3.67857800 0.96062900 -1.98421000
C -4.58722000 0.16909000 -2.94713400
H -4.81682100 0.77412500 -3.84870000
H -5.55271600 -0.08994000 -2.46800900
H -4.09681500 -0.77106200 -3.27044600
C -2.38793900 1.34722100 -2.72896100
H -1.88190900 0.44712800 -3.13820500
H -1.67192600 1.86788100 -2.06267000
H -2.62306400 2.02320900 -3.57674600
C -4.39791200 2.24821000 -1.51992000
H -4.64897200 2.89379900 -2.38772200
H -3.75264500 2.82409100 -0.82839900
H -5.33962000 2.00711700 -0.98846900
C -3.17849300 -1.57191300 2.61252300
C -4.55744300 -1.93532200 3.19451900
H -5.12032600 -1.02415500 3.47988000
H -4.44068600 -2.57391300 4.09460900
H -5.16635900 -2.49948100 2.45988700
C -2.42066400 -2.85131100 2.19361100
H -2.99862300 -3.43450900 1.45031500
H -2.22422300 -3.50453800 3.06936000
H -1.44568600 -2.58439700 1.73564700
C -2.35944500 -0.80857600 3.67178100
H -1.37504900 -0.50794300 3.25496600
H -2.17882700 -1.44266900 4.56406500

H -2.89039600 0.11165100 3.98883400
C -4.49707100 -2.48155800 -0.53066600
C -5.50203600 -3.38472000 -0.10388700
H -6.30636900 -3.03840400 0.56345700
C -5.47128400 -4.72280600 -0.51929800
H -6.25914800 -5.41540000 -0.18368900
C -4.43234700 -5.18631800 -1.35313300
H -4.40777800 -6.24041800 -1.67021200
C -3.43021700 -4.29505000 -1.77940000
H -2.61746100 -4.64719300 -2.43418000
C -3.46373600 -2.95250700 -1.37959500
H -2.69150200 -2.24203900 -1.71336000
C -6.00068700 -0.17719400 0.43252500
C -6.17512800 0.81861100 1.42783800
H -5.30665500 1.12486900 2.02808200
C -7.43142700 1.40050700 1.63433400
H -7.55220500 2.17361700 2.40954300
C -8.53793400 0.99935200 0.85962000
H -9.52548700 1.45691800 1.02509800
C -8.37259500 0.01035800 -0.13128100
H -9.23173700 -0.30533600 -0.74398600
C -7.11650700 -0.56994500 -0.35133700
H -6.99574000 -1.32889600 -1.13936300
C 4.57837000 -0.59281100 -2.42661600
C 3.57882000 -0.37124500 -3.57602400
H 2.76889800 -1.12923100 -3.54748700
H 4.09356600 -0.43670600 -4.55681900
H 3.11024700 0.63315700 -3.50119700
C 5.19262200 -2.00253500 -2.54421600
H 5.92234900 -2.18963300 -1.72969700
H 5.72898700 -2.10788700 -3.50978900
H 4.41303700 -2.78690800 -2.48655000
C 5.70727200 0.45949700 -2.51101100
H 5.28727400 1.48465300 -2.51736500
H 6.29864800 0.32928300 -3.44147100
H 6.39634900 0.37081000 -1.64752300
C 2.31073800 -0.26205700 2.35170700
C 1.44181700 -1.53713200 2.40594500
H 2.05342300 -2.44846100 2.25844100
H 0.66877600 -1.50643600 1.60916200
H 0.92012100 -1.62107700 3.38190700
C 3.38741200 -0.32162000 3.45288500
H 2.90750300 -0.39322900 4.45060900
H 4.02969400 0.58087800 3.43443800

| |
|---|
| H 4.03870800 -1.21080500 3.32694800<br>C 1.42493500 0.98095400 2.55547700<br>H 0.93159600 0.95700000 3.54858900<br>H 0.62166600 1.01803300 1.78904800<br>H 2.02242300 1.91103400 2.47307200<br>C 4.19504500 -2.72643700 0.63038400<br>C 3.20120800 -3.43065400 -0.11011100<br>H 2.62190100 -2.89537500 -0.88051900<br>C 2.94876600 -4.78148400 0.15042300<br>H 2.17248700 -5.30744400 -0.42797300<br>C 3.67857800 -5.46816200 1.14146100<br>H 3.48110700 -6.53269700 1.33996800<br>C 4.65908800 -4.77536200 1.88552100<br>H 5.22327600 -5.29850800 2.67387500<br>C 4.90946200 -3.41941200 1.64592200<br>H 5.64742700 -2.88375700 2.26208300<br>C 5.85240300 -0.31551200 0.96651900<br>C 7.06402500 -1.05748200 0.88364100<br>H 7.04560200 -2.10602800 0.54955900<br>C 8.28442300 -0.45137000 1.20215300<br>H 9.21378000 -1.03923400 1.13639900<br>C 8.33438600 0.90606800 1.58781200<br>H 9.29861600 1.37884900 1.82923900<br>C 7.13914000 1.65213000 1.65171500<br>H 7.16732400 2.71297400 1.94805100<br>C 5.91077200 1.05638200 1.34721600<br>H 4.97599900 1.63525800 1.39652600<br>Bi 0.04078900 -1.07798800 -1.39114900 |
| |

**Table S29.** The Cartesian coordinates of the studied complexes **4**, **5** and **6** with $C_s$ symmetry at the BP86-D3(BJ)/def2-TZVPP level.

| |
|---|
| **4**<br><br>**E = -7484.386545 au**<br><br>1 1<br>Ge -0.41995900 -0.34559600 2.06927700<br>Ge -0.41995900 -0.34559600 -2.06927700<br>P 0.86489500 0.26240500 4.33022800<br>P 0.86489500 0.26240500 -4.33022800 |

| | | | |
|---|---|---|---|
| O | -2.57994300 | 0.21297400 | 0.00000000 |
| N | 0.10846900 | 1.16832600 | 3.20050500 |
| N | 0.34492400 | -1.16525000 | 3.68252600 |
| N | 0.10846900 | 1.16832600 | -3.20050500 |
| N | 0.34492400 | -1.16525000 | -3.68252600 |
| C | -2.37025200 | -0.51036800 | 2.24445600 |
| C | -2.96734100 | -0.94807400 | 3.43272400 |
| H | -2.33308700 | -1.21435100 | 4.27788800 |
| C | -4.35427100 | -1.07176700 | 3.52224000 |
| H | -4.81374900 | -1.42615000 | 4.44619700 |
| C | -5.16119200 | -0.74628100 | 2.42616600 |
| H | -6.24318300 | -0.84699500 | 2.51249500 |
| C | -4.60083600 | -0.29168500 | 1.22697300 |
| C | -3.20342300 | -0.19724100 | 1.17194500 |
| C | -5.39665500 | 0.15484700 | 0.00000000 |
| C | -6.82576600 | -0.39760900 | 0.00000000 |
| H | -6.83748400 | -1.49684800 | 0.00000000 |
| H | -7.37736500 | -0.04199400 | -0.88072900 |
| H | -7.37736500 | -0.04199400 | 0.88072900 |
| C | -5.45162600 | 1.70826400 | 0.00000000 |
| H | -5.98188100 | 2.06669100 | 0.89426600 |
| H | -5.98188100 | 2.06669100 | -0.89426600 |
| H | -4.44196100 | 2.14156600 | 0.00000000 |
| C | -3.20342300 | -0.19724100 | -1.17194500 |
| C | -4.60083600 | -0.29168500 | -1.22697300 |
| C | -5.16119200 | -0.74628100 | -2.42616600 |
| H | -6.24318300 | -0.84699500 | -2.51249500 |
| C | -4.35427100 | -1.07176700 | -3.52224000 |
| H | -4.81374900 | -1.42615000 | -4.44619700 |
| C | -2.96734100 | -0.94807400 | -3.43272400 |
| H | -2.33308700 | -1.21435100 | -4.27788800 |
| C | -2.37025200 | -0.51036800 | -2.24445600 |
| C | -0.36970300 | 2.56711700 | -3.20544200 |
| C | 0.72607400 | 3.49674600 | -3.74550800 |
| H | 0.38847400 | 4.54230100 | -3.70551100 |
| H | 0.96135300 | 3.26642300 | -4.79424100 |
| H | 1.64691600 | 3.40473100 | -3.15321000 |
| C | -0.67019700 | 2.92739900 | -1.74590200 |
| H | 0.23042100 | 2.80683900 | -1.12866500 |
| H | -1.45590100 | 2.27829100 | -1.32958700 |
| H | -1.01813800 | 3.96722500 | -1.67152400 |
| C | -1.64739600 | 2.69139200 | -4.05285100 |
| H | -2.04488300 | 3.71611900 | -4.00525800 |
| H | -2.41875100 | 2.00032500 | -3.68500900 |

| | | | |
|---|---|---|---|
| H | -1.44570400 | 2.45183100 | -5.10604900 |
| C | 0.87806600 | -2.54332400 | -3.85271000 |
| C | 0.99285300 | -2.86198400 | -5.34971400 |
| H | 0.01800700 | -2.78048600 | -5.84908700 |
| H | 1.36754200 | -3.88601800 | -5.48764500 |
| H | 1.69580300 | -2.18136900 | -5.85263200 |
| C | 2.24793500 | -2.72153500 | -3.17563500 |
| H | 3.00899800 | -2.06787700 | -3.61990600 |
| H | 2.58765400 | -3.76240300 | -3.27985300 |
| H | 2.17862700 | -2.49832900 | -2.10014500 |
| C | -0.14263700 | -3.48493400 | -3.19883600 |
| H | -0.23632800 | -3.26802300 | -2.12281400 |
| H | 0.18243300 | -4.52953300 | -3.30383200 |
| H | -1.13240500 | -3.37663900 | -3.66385200 |
| C | 2.65805500 | 0.45997200 | -4.27274200 |
| C | 3.49505700 | 0.05728600 | -5.32741000 |
| H | 3.06511700 | -0.27988000 | -6.27184000 |
| C | 4.88080800 | 0.07607400 | -5.16472900 |
| H | 5.52754900 | -0.23718800 | -5.98559900 |
| C | 5.43817700 | 0.49083900 | -3.95047100 |
| H | 6.52216700 | 0.50284700 | -3.82610100 |
| C | 4.60850400 | 0.89288100 | -2.89859300 |
| H | 5.04233000 | 1.21842700 | -1.95186400 |
| C | 3.22311500 | 0.88196600 | -3.05646500 |
| H | 2.56790000 | 1.18571500 | -2.23645500 |
| C | 0.35736400 | 0.55461400 | -6.03589400 |
| C | -0.79742000 | -0.07392400 | -6.52893300 |
| H | -1.31518700 | -0.80239200 | -5.90687700 |
| C | -1.27365200 | 0.23065600 | -7.80262300 |
| H | -2.16885800 | -0.26530100 | -8.18056100 |
| C | -0.60392900 | 1.17012100 | -8.59408100 |
| H | -0.97814200 | 1.40939600 | -9.59063300 |
| C | 0.54648600 | 1.80047900 | -8.11036900 |
| H | 1.07114500 | 2.53174500 | -8.72693300 |
| C | 1.02890600 | 1.49542900 | -6.83701000 |
| H | 1.92689700 | 1.99018900 | -6.46494100 |
| C | -0.36970300 | 2.56711700 | 3.20544200 |
| C | -0.67019700 | 2.92739900 | 1.74590200 |
| H | 0.23042100 | 2.80683900 | 1.12866500 |
| H | -1.01813800 | 3.96722500 | 1.67152400 |
| H | -1.45590100 | 2.27829100 | 1.32958700 |
| C | 0.72607400 | 3.49674600 | 3.74550800 |
| H | 0.96135300 | 3.26642300 | 4.79424100 |
| H | 0.38847400 | 4.54230100 | 3.70551100 |

H 1.64691600 3.40473100 3.15321000
C -1.64739600 2.69139200 4.05285100
H -2.41875100 2.00032500 3.68500900
H -2.04488300 3.71611900 4.00525800
H -1.44570400 2.45183100 5.10604900
C 0.87806600 -2.54332400 3.85271000
C 2.24793500 -2.72153500 3.17563500
H 3.00899800 -2.06787700 3.61990600
H 2.17862700 -2.49832900 2.10014500
H 2.58765400 -3.76240300 3.27985300
C 0.99285300 -2.86198400 5.34971400
H 1.36754200 -3.88601800 5.48764500
H 0.01800700 -2.78048600 5.84908700
H 1.69580300 -2.18136900 5.85263200
C -0.14263700 -3.48493400 3.19883600
H 0.18243300 -4.52953300 3.30383200
H -0.23632800 -3.26802300 2.12281400
H -1.13240500 -3.37663900 3.66385200
C 2.65805500 0.45997200 4.27274200
C 3.22311500 0.88196600 3.05646500
H 2.56790000 1.18571500 2.23645500
C 4.60850400 0.89288100 2.89859300
H 5.04233000 1.21842700 1.95186400
C 5.43817700 0.49083900 3.95047100
H 6.52216700 0.50284700 3.82610100
C 4.88080800 0.07607400 5.16472900
H 5.52754900 -0.23718800 5.98559900
C 3.49505700 0.05728600 5.32741000
H 3.06511700 -0.27988000 6.27184000
C 0.35736400 0.55461400 6.03589400
C 1.02890600 1.49542900 6.83701000
H 1.92689700 1.99018900 6.46494100
C 0.54648600 1.80047900 8.11036900
H 1.07114500 2.53174500 8.72693300
C -0.60392900 1.17012100 8.59408100
H -0.97814200 1.40939600 9.59063300
C -1.27365200 0.23065600 7.80262300
H -2.16885800 -0.26530100 8.18056100
C -0.79742000 -0.07392400 6.52893300
H -1.31518700 -0.80239200 5.90687700
Bi 1.19837300 -0.73240000 0.00000000

**5**

**E = -7484.108712 au**

2 2
Ge -0.51188700 -0.29911200 2.15713400
Ge -0.51188700 -0.29911200 -2.15713400
P 0.92542900 0.17989600 4.33163300
P 0.92542900 0.17989600 -4.33163300
O -2.62728500 0.21362500 0.00000000
N 0.16689700 1.14678000 3.22861600
N 0.32111500 -1.20651400 3.63772200
N 0.16689700 1.14678000 -3.22861600
N 0.32111500 -1.20651400 -3.63772200
C -2.44778000 -0.46109900 2.26783200
C -3.05054600 -0.86069600 3.46863800
H -2.42765900 -1.09381500 4.33148800
C -4.43727300 -0.97388700 3.54849300
H -4.90748200 -1.29631300 4.47809200
C -5.23056000 -0.67285700 2.43571600
H -6.31357500 -0.76189300 2.51778500
C -4.66276200 -0.25037700 1.22826000
C -3.26475000 -0.16796900 1.17479600
C -5.46018100 0.18724500 0.00000000
C -6.88605900 -0.37512700 0.00000000
H -6.89294200 -1.47424100 0.00000000
H -7.44131900 -0.02125300 -0.87851500
H -7.44131900 -0.02125300 0.87851500
C -5.52393000 1.74111600 0.00000000
H -6.05774500 2.09550000 0.89318900
H -6.05774500 2.09550000 -0.89318900
H -4.51780400 2.18335900 0.00000000
C -3.26475000 -0.16796900 -1.17479600
C -4.66276200 -0.25037700 -1.22826000
C -5.23056000 -0.67285700 -2.43571600
H -6.31357500 -0.76189300 -2.51778500
C -4.43727300 -0.97388700 -3.54849300
H -4.90748200 -1.29631300 -4.47809200
C -3.05054600 -0.86069600 -3.46863800
H -2.42765900 -1.09381500 -4.33148800
C -2.44778000 -0.46109900 -2.26783200
C -0.28767500 2.55935800 -3.28648600
C 0.83656100 3.44618200 -3.83529900
H 0.51633100 4.49713600 -3.84270700
H 1.08809000 3.17394600 -4.86982300

| | | | |
|---|---|---|---|
| H | 1.74429100 | 3.36513000 | -3.22180500 |
| C | -0.60936100 | 2.96274000 | -1.84221200 |
| H | 0.28598900 | 2.87757900 | -1.20854100 |
| H | -1.40148300 | 2.32505700 | -1.41654700 |
| H | -0.96492100 | 4.00116900 | -1.80201200 |
| C | -1.54616900 | 2.67536700 | -4.16176900 |
| H | -1.91548600 | 3.71074700 | -4.16867000 |
| H | -2.34563600 | 2.02487200 | -3.78002900 |
| H | -1.33033300 | 2.38425700 | -5.19868000 |
| C | 0.81335900 | -2.61052500 | -3.68553100 |
| C | 0.98640100 | -3.03954100 | -5.14893500 |
| H | 0.03925500 | -2.96434500 | -5.69885800 |
| H | 1.33119000 | -4.08142100 | -5.19640900 |
| H | 1.73558200 | -2.41985500 | -5.66235500 |
| C | 2.14285600 | -2.77300500 | -2.93042600 |
| H | 2.94259700 | -2.16745000 | -3.37463900 |
| H | 2.46204100 | -3.82463800 | -2.94187300 |
| H | 2.02891200 | -2.47165600 | -1.87578900 |
| C | -0.27016100 | -3.46211600 | -3.01298800 |
| H | -0.41234300 | -3.15668600 | -1.96156700 |
| H | 0.02052700 | -4.52126700 | -3.00975900 |
| H | -1.23305500 | -3.36400900 | -3.53231500 |
| C | 2.71551100 | 0.32737000 | -4.24273100 |
| C | 3.54025200 | -0.18901900 | -5.25798200 |
| H | 3.10202900 | -0.58510200 | -6.17514100 |
| C | 4.92569200 | -0.19740200 | -5.09207100 |
| H | 5.56346300 | -0.59787900 | -5.88100800 |
| C | 5.49440100 | 0.30989400 | -3.91921800 |
| H | 6.57822800 | 0.30529200 | -3.79535300 |
| C | 4.67763700 | 0.83412600 | -2.91107200 |
| H | 5.12476100 | 1.24607600 | -2.00528600 |
| C | 3.29290300 | 0.84353900 | -3.07003800 |
| H | 2.64727700 | 1.26326300 | -2.29634300 |
| C | 0.45227000 | 0.40912100 | -6.04575000 |
| C | -0.69010200 | -0.24033700 | -6.54360200 |
| H | -1.22114500 | -0.95156900 | -5.91244900 |
| C | -1.12629700 | 0.01242000 | -7.84238800 |
| H | -2.00725300 | -0.50069200 | -8.23001300 |
| C | -0.43134700 | 0.92069800 | -8.64897900 |
| H | -0.77513200 | 1.11902600 | -9.66510900 |
| C | 0.70723500 | 1.56941300 | -8.15987400 |
| H | 1.25152000 | 2.27194000 | -8.79209200 |
| C | 1.15407400 | 1.31499600 | -6.86339300 |
| H | 2.04710900 | 1.81735200 | -6.48936500 |

C -0.28767500 2.55935800 3.28648600
C -0.60936100 2.96274000 1.84221200
H 0.28598900 2.87757900 1.20854100
H -0.96492100 4.00116900 1.80201200
H -1.40148300 2.32505700 1.41654700
C 0.83656100 3.44618200 3.83529900
H 1.08809000 3.17394600 4.86982300
H 0.51633100 4.49713600 3.84270700
H 1.74429100 3.36513000 3.22180500
C -1.54616900 2.67536700 4.16176900
H -2.34563600 2.02487200 3.78002900
H -1.91548600 3.71074700 4.16867000
H -1.33033300 2.38425700 5.19868000
C 0.81335900 -2.61052500 3.68553100
C 2.14285600 -2.77300500 2.93042600
H 2.94259700 -2.16745000 3.37463900
H 2.02891200 -2.47165600 1.87578900
H 2.46204100 -3.82463800 2.94187300
C 0.98640100 -3.03954100 5.14893500
H 1.33119000 -4.08142100 5.19640900
H 0.03925500 -2.96434500 5.69885800
H 1.73558200 -2.41985500 5.66235500
C -0.27016100 -3.46211600 3.01298800
H 0.02052700 -4.52126700 3.00975900
H -0.41234300 -3.15668600 1.96156700
H -1.23305500 -3.36400900 3.53231500
C 2.71551100 0.32737000 4.24273100
C 3.29290300 0.84353900 3.07003800
H 2.64727700 1.26326300 2.29634300
C 4.67763700 0.83412600 2.91107200
H 5.12476100 1.24607600 2.00528600
C 5.49440100 0.30989400 3.91921800
H 6.57822800 0.30529200 3.79535300
C 4.92569200 -0.19740200 5.09207100
H 5.56346300 -0.59787900 5.88100800
C 3.54025200 -0.18901900 5.25798200
H 3.10202900 -0.58510200 6.17514100
C 0.45227000 0.40912100 6.04575000
C 1.15407400 1.31499600 6.86339300
H 2.04710900 1.81735200 6.48936500
C 0.70723500 1.56941300 8.15987400
H 1.25152000 2.27194000 8.79209200
C -0.43134700 0.92069800 8.64897900
H -0.77513200 1.11902600 9.66510900

C -1.12629700 0.01242000 7.84238800
H -2.00725300 -0.50069200 8.23001300
C -0.69010200 -0.24033700 6.54360200
H -1.22114500 -0.95156900 5.91244900
Bi 1.13366400 -0.38540600 0.00000000

**6**

**E = -7484.516997 au**

0 2
Ge -0.42388100 -0.35909800 2.08242900
Ge -0.42388100 -0.35909800 -2.08242900
P 0.88340900 0.28672800 4.34907000
P 0.88340900 0.28672800 -4.34907000
O -2.59045500 0.21329100 0.00000000
N 0.10053100 1.16060400 3.19261600
N 0.33673100 -1.15033600 3.69889700
N 0.10053100 1.16060400 -3.19261600
N 0.33673100 -1.15033600 -3.69889700
C -2.37904900 -0.50753900 2.24673900
C -2.98376900 -0.94144500 3.43371400
H -2.35026300 -1.21338400 4.27811700
C -4.37152600 -1.05789900 3.52355500
H -4.83184100 -1.40811600 4.44926900
C -5.17766100 -0.73046300 2.42719800
H -6.26059800 -0.82443800 2.51235200
C -4.61088200 -0.28152700 1.22794200
C -3.21319200 -0.19305400 1.17332900
C -5.40140700 0.17139500 0.00000000
C -6.83572400 -0.36738400 0.00000000
H -6.85572100 -1.46667900 0.00000000
H -7.38322500 -0.00773900 -0.88212500
H -7.38322500 -0.00773900 0.88212500
C -5.44394100 1.72492900 0.00000000
H -5.96976300 2.08848300 0.89542200
H -5.96976300 2.08848300 -0.89542200
H -4.42971500 2.14695200 0.00000000
C -3.21319200 -0.19305400 -1.17332900
C -4.61088200 -0.28152700 -1.22794200
C -5.17766100 -0.73046300 -2.42719800
H -6.26059800 -0.82443800 -2.51235200
C -4.37152600 -1.05789900 -3.52355500
H -4.83184100 -1.40811600 -4.44926900
C -2.98376900 -0.94144500 -3.43371400

H -2.35026300 -1.21338400 -4.27811700
C -2.37904900 -0.50753900 -2.24673900
C -0.35865400 2.56014700 -3.19228200
C 0.72734600 3.47505700 -3.77950100
H 0.40611400 4.52605000 -3.72434000
H 0.91500400 3.23757600 -4.83656200
H 1.67056600 3.36727900 -3.22592500
C -0.61076800 2.94342100 -1.72929800
H 0.30658500 2.81841400 -1.13811400
H -1.38750200 2.30516300 -1.28131400
H -0.94512600 3.98871700 -1.65596500
C -1.66071400 2.69455000 -4.00288100
H -2.03998000 3.72754800 -3.96427800
H -2.43132700 2.02318500 -3.59824400
H -1.49253300 2.42673100 -5.05534900
C 0.87262000 -2.51736800 -3.90071100
C 1.00896400 -2.79319600 -5.40576800
H 0.04544000 -2.66800600 -5.91871200
H 1.36243100 -3.82207600 -5.56879300
H 1.73378100 -2.10966900 -5.87183800
C 2.23590500 -2.71653400 -3.21321500
H 2.99532400 -2.03733900 -3.62077400
H 2.58570000 -3.75063900 -3.35445900
H 2.15055100 -2.53533400 -2.13089300
C -0.15090500 -3.48685700 -3.29195500
H -0.26065300 -3.30339900 -2.21156100
H 0.18062300 -4.52670100 -3.42928400
H -1.13500900 -3.36484700 -3.76634600
C 2.66278600 0.49433000 -4.23774000
C 3.54991100 0.12789800 -5.27168900
H 3.15648500 -0.18551300 -6.23991500
C 4.92589200 0.13993500 -5.05702800
H 5.60065100 -0.14465100 -5.86662800
C 5.44444300 0.49751400 -3.80296200
H 6.52277200 0.49641900 -3.63592000
C 4.57075500 0.85021700 -2.76817600
H 4.96409800 1.12350500 -1.78711700
C 3.19352100 0.85833000 -2.97960500
H 2.51217500 1.12825100 -2.16926700
C 0.39602900 0.57509800 -6.04034700
C -0.82263600 0.02998300 -6.51034500
H -1.39116100 -0.63394300 -5.86101000
C -1.29280900 0.33694000 -7.78286700
H -2.23453000 -0.09539600 -8.12701800

C -0.56545600 1.19205400 -8.62245500
H -0.93569000 1.43011600 -9.62055300
C 0.64008900 1.74637500 -8.16349800
H 1.20894400 2.42314700 -8.80406300
C 1.11349400 1.45207600 -6.88874700
H 2.03745300 1.91164500 -6.53599900
C -0.35865400 2.56014700 3.19228200
C -0.61076800 2.94342100 1.72929800
H 0.30658500 2.81841400 1.13811400
H -0.94512600 3.98871700 1.65596500
H -1.38750200 2.30516300 1.28131400
C 0.72734600 3.47505700 3.77950100
H 0.91500400 3.23757600 4.83656200
H 0.40611400 4.52605000 3.72434000
H 1.67056600 3.36727900 3.22592500
C -1.66071400 2.69455000 4.00288100
H -2.43132700 2.02318500 3.59824400
H -2.03998000 3.72754800 3.96427800
H -1.49253300 2.42673100 5.05534900
C 0.87262000 -2.51736800 3.90071100
C 2.23590500 -2.71653400 3.21321500
H 2.99532400 -2.03733900 3.62077400
H 2.15055100 -2.53533400 2.13089300
H 2.58570000 -3.75063900 3.35445900
C 1.00896400 -2.79319600 5.40576800
H 1.36243100 -3.82207600 5.56879300
H 0.04544000 -2.66800600 5.91871200
H 1.73378100 -2.10966900 5.87183800
C -0.15090500 -3.48685700 3.29195500
H 0.18062300 -4.52670100 3.42928400
H -0.26065300 -3.30339900 2.21156100
H -1.13500900 -3.36484700 3.76634600
C 2.66278600 0.49433000 4.23774000
C 3.19352100 0.85833000 2.97960500
H 2.51217500 1.12825100 2.16926700
C 4.57075500 0.85021700 2.76817600
H 4.96409800 1.12350500 1.78711700
C 5.44444300 0.49751400 3.80296200
H 6.52277200 0.49641900 3.63592000
C 4.92589200 0.13993500 5.05702800
H 5.60065100 -0.14465100 5.86662800
C 3.54991100 0.12789800 5.27168900
H 3.15648500 -0.18551300 6.23991500
C 0.39602900 0.57509800 6.04034700

C 1.11349400 1.45207600 6.88874700
H 2.03745300 1.91164500 6.53599900
C 0.64008900 1.74637500 8.16349800
H 1.20894400 2.42314700 8.80406300
C -0.56545600 1.19205400 8.62245500
H -0.93569000 1.43011600 9.62055300
C -1.29280900 0.33694000 7.78286700
H -2.23453000 -0.09539600 8.12701800
C -0.82263600 0.02998300 6.51034500
H -1.39116100 -0.63394300 5.86101000
Bi 1.15980900 -0.80821900 0.00000000

# References


(1) Rufanov, K. A.; Pruß, N. K.; Sundermeyer, J. Simple Entry into *N*-tert-Butyl-Iminophosphonamide Rare-Earth Metal Alkyl and Chlorido Complexes. *Dalton Trans.* **2016**, *45*, 1525–1538.

(2) Chávez, I.; Alvarez-Carena, A.; Molins, E.; Roig, A.; Maniukiewicz, W.; Arancibia, A.; Arancibia, V.; Brand, H.; Manríquez, J. M. Selective Oxidants for Organometallic Compounds Containing a Stabilising Anion of Highly Reactive Cations:(3, 5 (CF3) 2C6H3) 4B−) Cp2Fe+ and (3, 5 (CF3) 2C6H3) 4B−) Cp* 2Fe+. *J. Organomet. Chem.* **2000**, *601*, 126–132.

(3) Sheldrick, G. Crystal Structure Refinement with SHELXL. *Acta Crystallogr. Sect. C* **2015**, *71*, 3–8.

(4) Dolomanov, O. V; Bourhis, L. J.; Gildea, R. J.; Howard, J. A. K.; Puschmann, H. OLEX2: A Complete Structure Solution, Refinement and Analysis Program. *J. Appl. Crystallogr.* **2009**, *42*, 339–341.

(5) Bain, G. A.; Berry, J. F. Diamagnetic Corrections and Pascal's Constants. *J. Chem. Educ.* **2008**, *85*, 532.

(6) Chilton, N. F.; Anderson, R. P.; Turner, L. D.; Soncini, A.; Murray, K. S. PHI: A Powerful New Program for the Analysis of Anisotropic Monomeric and Exchange-coupled Polynuclear D-and F-block Complexes. *J. Comput. Chem.* **2013**, *34*, 1164–1175.

(7) Neese, F.; Wiley, J. The ORCA Program System. *Wiley Interdiscip. Rev. Comput. Mol. Sci.* **2012**, *2*, 73–78.

(8) Neese, F. Prediction of Electron Paramagnetic Resonance g Values Using Coupled Perturbed Hartree–Fock and Kohn–Sham Theory. *J. Chem. Phys.* **2001**, *115*, 11080–11096.

(9) Neese, F. Efficient and Accurate Approximations to the Molecular Spin-Orbit Coupling Operator and Their Use in Molecular g-Tensor Calculations. *J. Chem. Phys.* **2005**, *122*.

(10) Weigend, F.; Ahlrichs, R. Balanced Basis Sets of Split Valence, Triple Zeta Valence and Quadruple Zeta Valence Quality for H to Rn: Design and Assessment of Accuracy. *Phys. Chem. Chem. Phys.* **2005**, *7*, 3297–3305.

(11) Perdew, J. P. Phys Rev B 33: 8822. *Erratum Phys Rev B* **1986**, *34*, 7406.

(12) Becke, A. D. Density-Functional Exchange-Energy Approximation with Correct Asymptotic Behavior. *Phys. Rev. A* **1988**, *38*, 3098.

(13) Weigend, F.; Ahlrichs, R. Balanced Basis Sets of Split Valence, Triple Zeta Valence and Quadruple Zeta Valence Quality for H to Rn: Design and Assessment of Accuracy. *Phys. Chem. Chem. Phys.* **2005**, *7*, 3297–3305.

(14) Weigend, F. Accurate Coulomb-Fitting Basis Sets for H to Rn. *Phys. Chem. Chem. Phys.* **2006**, *8*, 1057–1065.

(15) Grimme, S.; Antony, J.; Ehrlich, S.; Krieg, H. A Consistent and Accurate Ab Initio Parametrization of Density Functional Dispersion Correction (DFT-D) for the 94 Elements H-Pu. *J. Chem. Phys.* **2010**, *132*.

(16) Frisch, M. J.; Trucks, G. W.; Schlegel, H. B.; Scuseria, G. E.; Robb, M. a.; Cheeseman, J. R.; Scalmani, G.; Barone, V.; Petersson, G. a.; Nakatsuji, H.; Li, X.; Caricato, M.; Marenich, a. V.; Bloino, J.; Janesko, B. G.; Gomperts, R.; Mennucci, B.; Hratchian, H. P.; Ortiz, J. V.; Izmaylov, a. F.; Sonnenberg, J. L.; Williams; Ding, F.; Lipparini, F.; Egidi, F.; Goings, J.; Peng, B.; Petrone, A.; Henderson, T.; Ranasinghe, D.; Zakrzewski, V. G.; Gao, J.; Rega, N.; Zheng, G.; Liang, W.; Hada, M.; Ehara, M.; Toyota, K.; Fukuda, R.; Hasegawa, J.; Ishida, M.; Nakajima, T.; Honda, Y.; Kitao, O.; Nakai, H.; Vreven, T.; Throssell, K.; Montgomery Jr., J. a.; Peralta, J. E.; Ogliaro, F.; Bearpark, M. J.; Heyd, J. J.; Brothers, E. N.; Kudin, K. N.; Staroverov, V. N.; Keith, T. a.; Kobayashi, R.; Normand, J.; Raghavachari, K.; Rendell, a. P.; Burant, J. C.; Iyengar, S. S.; Tomasi, J.; Cossi, M.; Millam, J. M.; Klene, M.; Adamo, C.; Cammi, R.; Ochterski, J. W.; Martin, R. L.; Morokuma, K.; Farkas, O.; Foresman, J. B.; Fox, D. J. G16_C01. 2016, p Gaussian 16, Revision C.01, Gaussian, Inc., Wallin.

(17) Stoll, S.; Schweiger, A. EasySpin, a Comprehensive Software Package for Spectral Simulation and Analysis in EPR. *J. Magn. Reson.* **2006**, *178*, 42–55.

(18) Reed, A. E.; Weinstock, R. B.; Weinhold, F. Natural Population Analysis. *J. Chem. Phys.* **1985**, *83*, 735–746.

(19) Glendening, E. D.; Landis, C. R.; Weinhold, F. NBO 7.0: New Vistas in Localized and Delocalized Chemical Bonding Theory. *J. Comput. Chem.* **2019**, *40*, 2234–2241.

(20) R. F. W. Bader. Atoms in Molecules: A Quantum Theory, Oxford University Press. **1990**, 438.

(21) Lu, T.; Chen, F. Multiwfn: A Multifunctional Wavefunction Analyzer. *J. Comput. Chem.* **2012**, *33*, 580–592.

(22) Ziegler, T.; Rauk, A. On the Calculation of Bonding Energies by the Hartree Fock Slater Method: I. The Transition State Method. *Theor. Chim. Acta* **1977**, *46*, 1–10.

(23) Mitoraj, M.; Michalak, A. Donor-Acceptor Properties of Ligands from the Natural Orbitals for Chemical Valence. *Organometallics* **2007**, *26*, 6576–6580.

(24) Te Velde, G. t; Bickelhaupt, F. M.; Baerends, E. J.; Fonseca Guerra, C.; van Gisbergen, S. J. A.; Snijders, J. G.; Ziegler, T. Chemistry with ADF. *J. Comput. Chem.* **2001**, *22*, 931–967.

(25) Frenking, G.; Matthias Bickelhaupt, F. The EDA Perspective of Chemical Bonding. In *The Chemical Bond*; 2014; pp 121–157.

(26) Zhao, L.; von Hopffgarten, M.; Andrada, D. M.; Frenking, G. Energy Decomposition Analysis. *Wiley Interdiscip. Rev. Comput. Mol. Sci.* **2018**, *8*, e1345.

(27) Zhao, L.; Pan, S.; Holzmann, N.; Schwerdtfeger, P.; Frenking, G. Chemical Bonding and Bonding Models of Main-Group Compounds. *Chem. Rev.* **2019**, *119*, 8781–8845.

(28) Takahashi, S.; Sekiguchi, J.; Ishii, A.; Nakata, N.; Takahashi, S.; Sekiguchi, J.; Ishii, A.; Nakata, N. An Iminophosphonamido-Chlorosilylene as a Strong σ-Donating NHSi Ligand: Synthesis and Coordination Chemistry. *Angew. Chem. Int. Ed.* **2021**, *60*, 4055–4059.
